# Public Interest and Accessibility of Telehealth in Japan: Retrospective Analysis Using Google Trends and National Surveillance

**DOI:** 10.2196/36525

**Published:** 2022-09-14

**Authors:** Takuya Kinoshita, Takehiro Matsumoto, Naota Taura, Tetsuya Usui, Nemu Matsuya, Mayumi Nishiguchi, Hozumi Horita, Kazuhiko Nakao

**Affiliations:** 1 Department of Medical Informatics Nagasaki University Nagasaki Japan; 2 Department of Medical Informatics Nagasaki University Hospital Nagasaki Japan; 3 Department of Laboratory Medicine Nagasaki University Hospital Nagasaki Japan; 4 Department of Neurology National Hospital Organization Nagasaki Kawatana Medical Center Kawatana Japan

**Keywords:** COVID-19, telehealth, telemedicine, public interest, mobile app, correlation, infodemiology, infoveillance, surveillance, Google Trends

## Abstract

**Background:**

Recently, the use of telehealth for patient treatment under the COVID-19 pandemic has gained interest around the world. As a result, many infodemiology and infoveillance studies using web-based sources such as Google Trends were reported, focusing on the first wave of the COVID-19 pandemic. Although public interest in telehealth has increased in many countries during this time, the long-term interest has remained unknown among people living in Japan. Moreover, various mobile telehealth apps have become available for remote areas in the COVID-19 era, but the accessibility of these apps in epidemic versus nonepidemic regions is unknown.

**Objective:**

We aimed to investigate the public interest in telehealth during the first pandemic wave and after the wave in the first part of this study, and the accessibility of medical institutions using telehealth in the epidemic and nonepidemic regions, in the second part.

**Methods:**

We examined and compared the first wave and after the wave with regards to severe cases, number of deaths, relative search volume (RSV) of telehealth and COVID-19, and the correlation between RSV and COVID-19 cases, using open sources such as Google Trends and the Japanese Ministry of Health, Labour and Welfare (JMHLW) data. The weekly mean and the week-over-week change rates of RSV and COVID-19 cases were used to examine the correlation coefficients. In the second part, the prevalence of COVID-19 cases, severe cases, number of deaths, and the telehealth accessibility rate were compared between epidemic regions and nonepidemic regions, using the JMHLW data. We also examined the regional correlation between telehealth accessibility and the prevalence of COVID-19 cases.

**Results:**

Among the 83 weeks with 5 pandemic waves, the overall mean for the RSV of telehealth and COVID-19 was 11.3 (95% CI 8.0-14.6) and 30.7 (95% CI 27.2-34.2), respectively. The proportion of severe cases (26.54% vs 18.16%; *P*<.001), deaths (5.33% vs 0.99%; *P*<.001), RSV of telehealth (mean 33.1, 95% CI 16.2-50.0 vs mean 7.3, 95% CI 6.7-8.0; *P*<.001), and RSV of COVID-19 (mean 52.1, 95% CI 38.3-65.9 vs mean 26.3, 95% CI 24.4-29.2; *P*<.001) was significantly higher in the first wave compared to after the wave. In the correlation analysis, the public interest in telehealth was 0.899 in the first wave and –0.300 overall. In Japan, the accessibility of telehealth using mobile apps was significantly higher in epidemic regions compared to nonepidemic regions in both hospitals (3.8% vs 2.0%; *P*=.004) and general clinics (5.2% vs 3.1%; *P*<.001). In the regional correlation analysis, telehealth accessibility using mobile apps was 0.497 in hospitals and 0.629 in general clinics.

**Conclusions:**

Although there was no long-term correlation between the public interest in telehealth and COVID-19, there was a regional correlation between mobile telehealth app accessibility in Japan, especially for general clinics. We also revealed that epidemic regions had higher mobile telehealth app accessibility. Further studies about the actual use of telehealth and its effect after the COVID-19 pandemic are necessary.

## Introduction

Due to the global spread of COVID-19, the government of Japan announced a state of emergency for self-restraint [1]. In the meantime, the World Health Organization recommended telemedicine for treating patients with critical illnesses [2]. Correspondingly, all first visits using telephone or video conferencing were covered temporarily in Japan. Although first visits using telehealth were not covered until then, revisits using telephone had increased after the Tohoku Earthquake in 2011 [3]. Recently, various telehealth apps in mobile devices (or mobile apps) became available in Japan for patients in remote areas. These apps are convenient for patients who have difficulties visiting hospitals, and they attracted the attention of medical practitioners and the public after the emergence of COVID-19 [4]. Along with the demand for telehealth, studies investigating public interest in telehealth increased with the spread of COVID-19 [5,6]. Recently, studies in the fields of infodemiology and infoveillance using web-based sources such as Google Trends are becoming popular for health policy and assessment [7]. Google Trends contain a massive quantity of real-time search interests used for alternative surveillance and health care policy decisions by investigating their correlation [6]. However, most of these studies investigate only the first wave of COVID-19, and therefore, after the first pandemic wave remains unknown [5,6]. There is a study investigating the search term for an alternative surveillance in Japan, using other search engines [8]; however, there is no study focusing on public interest in telehealth in Japan using Google Trends.

In terms of the accessibility of telehealth, many medical institutions started telehealth after COVID-19. In the United States, the accessibility of telehealth increased after the COVID-19 pandemic [9], but the accessibility of medical institutions using telehealth, especially mobile apps, is unknown. The correlation between telehealth accessibility rate and COVID-19 cases may be in line with the hypothesis that epidemic regions have higher accessibility rates. However, we do not know about this correlation across regions. In the first part of this study, we aimed to investigate the long-term correlation between public interest in telehealth and COVID-19 and compared the differences between the first pandemic wave and after the wave. In the second part, we investigated the accessibility of medical institutions using telehealth in the epidemic and nonepidemic regions in Japan.

## Methods

### Data Sources

We used various types of data in this study. For investigating the correlation between public interest in telehealth and COVID-19, we used Google Trends’ relative search volume (RSV). RSV is an index of 0-100 representing public interest regarding a specific topic in a certain time and region and with a certain search term. High RSV indicates that many users are searching for a certain term at that point in time in the observed period.

For data about COVID-19, we used nationwide open data from the Japanese Ministry of Health, Labour and Welfare (JMHLW). COVID-19 data include routinely collected daily or cumulative confirmed number of cases, polymerase chain reaction tests, hospitalized cases, severe cases, and deaths from 47 prefectures of Japan; the data were obtained in October 2021. For telehealth accessibility data, a list of telehealth-implemented medical institution names, addresses, telephone numbers, websites, and departments were collected by each prefecture and reported to the government; the data were obtained in October 2020.

To investigate the accessibility of telehealth mobile apps, we used company data from medical institutions listed on the company website. The mobile apps observed were “Clinics,” “Curon,” “YaDoc,” “Carada/Lunaluna,” and “Pocket Doctor.” These companies had an open list of contract medical institutions in each prefecture on their website. The data were obtained in October 2020. For the denominator used for calculating the proportion of accessible institutions and the prevalence of COVID-19, registered medical institutions and the population in each prefecture from a yearly statistical report called E-STAT were used. The latest annual data about medical institutions were for 2019, and the population data were for 2020, both published in September 2020 and December 2021. The data were obtained in October 2020 and December 2021, respectively.

### Ethical Considerations

The data used in the study were all open data without personal information. Therefore, informed consent was not obtained. The data from JMHLW were also open source. Therefore, no ethical approval was necessary.

### Study Design and Setting

This study had 2 parts. First, we examined the relationship between COVID-19 cases and the RSV of telehealth and COVID-19. The keywords for COVID-19 and telehealth were “corona” and “online-shinryou” in Japanese. These keywords had the largest search volume compared with other potential search terms (Multimedia Appendix 1). The observed period was 83 weeks (March 1, 2020, to October 2, 2021), with 5 pandemic waves. Both the COVID-19 cases and RSVs were averaged weekly. For comparison, the proportion of severe cases, deaths, and the mean RSV of telehealth and COVID-19 were compared between the first pandemic wave (week 1-13) and after it (week 14-83). The correlation between RSV and COVID-19 cases and the week-over-week change rate were also investigated. Telehealth accessibility between epidemic and nonepidemic regions was then investigated. Tokyo, Kanagawa, Saitama, Chiba, Osaka, Fukuoka, Hokkaido, Ibaraki, Ishikawa, Gifu, Aichi, and Kyoto were selected as epidemic regions (Multimedia Appendix 2). In these prefectures, a state of emergency was declared in the first wave of the COVID-19 pandemic. We included all the institutions from the observed data (Multimedia Appendix 3). Invalid samples such as the ones with missing names, addresses, or domains were excluded. Duplicated data were also excluded from the study. The data were reviewed by 2 researchers and double-checked for compliance. To investigate the regional correlations between COVID-19 cases and telehealth accessibility, we first compared the mean prevalence of COVID-19 cases, severe cases, and deaths; secondly, the mean telehealth accessibility rate of telephone and mobile apps was examined between epidemic and nonepidemic regions. Additionally, for mobile apps, we investigated the regional correlation between the proportion of telehealth-accessible medical institutions and COVID-19 prevalence in each prefecture.

### Statistical Analysis

We assumed the data had no normality. Therefore, the chi-square test for comparing proportions, Mann-Whitney *U* test for comparing means, and Spearman rank-order correlation test for correlation analysis were applied. A *P* value of .05 was considered statistically significant. All analyses were conducted using SPSS software (version 26; IBM Corp).

## Results

### Characteristics and RSV

**Figure 1:**
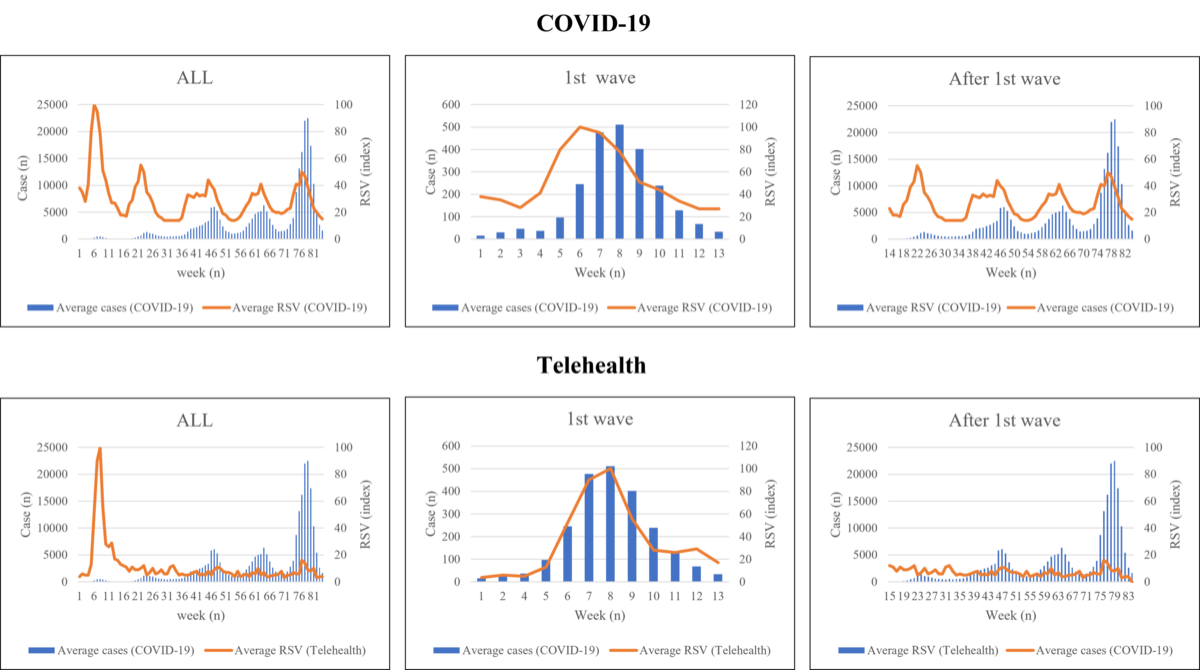
Public interest and mean weekly COVID-19 cases. RSV: relative search volume.

**Figure 2:**
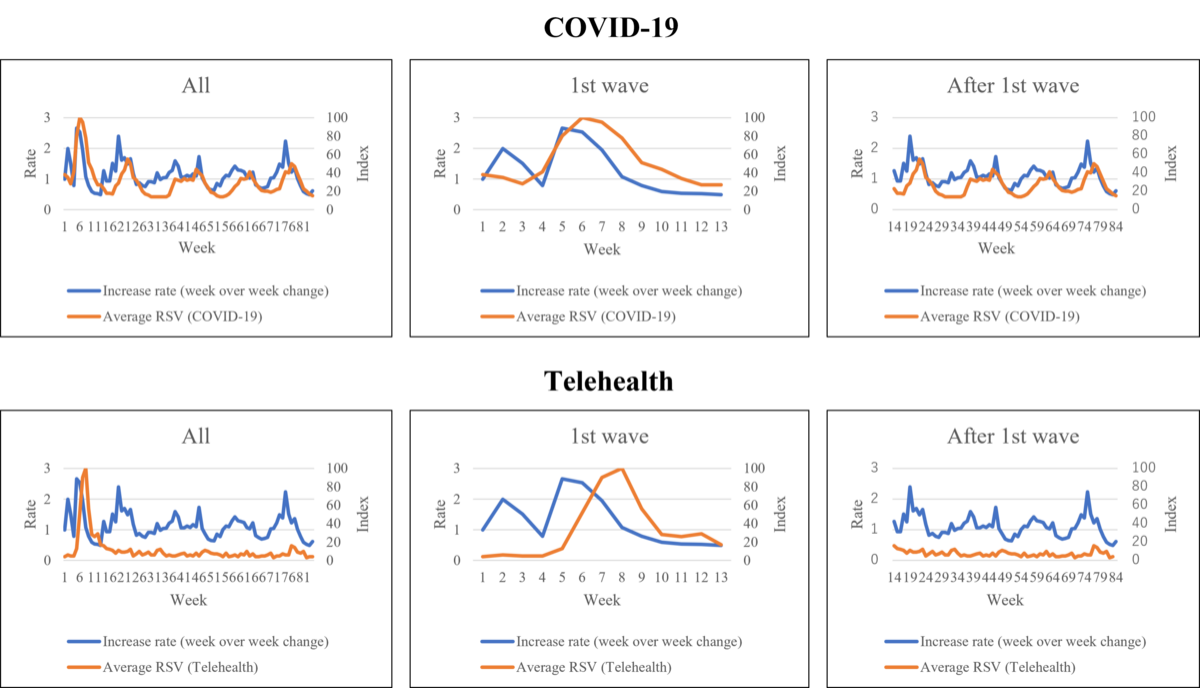
Public interest and week-over-week increase rate of COVID-19 cases. RSV: relative search volume.

In the observed 83 weeks with 5 pandemic waves, there were 1,707,581 COVID-19 cases overall: 16,693 in the first pandemic wave and 1,690,888 after the first pandemic wave. The proportion of severe cases (26.54% vs 18.16%; *P*<.001) and deaths (5.33% vs 0.99%; *P*<.001) were significantly higher in the first pandemic wave compared to after the wave (Table 1). The Japanese public interest in telehealth and COVID-19 was the highest in the first wave, and it decreased with time (Figure 1). In terms of increase rate, the highest increase rate was observed in the 5th week (Figure 2). The overall mean RSV of telehealth and COVID-19 was 11.3 (95% CI 8.0-14.6) and 30.7 (95% CI 27.2-34.2), respectively. The mean RSV of telehealth in the first wave was significantly higher compared to the mean RSV of telehealth after the first wave (33.1 vs 7.3; *P<*.001), and this also was the case in the mean RSV of COVID-19 (52.1 vs 26.8; *P<*.001).

**Table 1:** Basic characteristics of COVID-19 and relative search volume (RSV) in the first wave and after the first wave.

Characteristics		Overall (n=1,707,581)	First wave (n=16,693)	After the first wave (n=1,690,888)	*P* value
**COVID-19 characteristics, n (%)**					
	Severe cases	311,562 (18.24)	4417 (26.54)	307,145 (18.16)	<.001
	Deaths	17,709 (1.03)	891 (5.33)	16,818 (0.99)	<.001
**RSV, mean (95% CI)**					
	Telehealth (search term “online-shinryou”)	11.3 (8.0-14.7)	33.1 (16.2-50.0)	7.3 (6.7-8.0)	<.001
	COVID-19 (search term “corona”)	30.7 (27.2-34.3)	52.1 (38.3-65.9)	26.8 (24.4-29.2)	<.001
**Correlation coefficient^a^ for telehealth RSV, r**					
	Mean weekly cases	–0.300	–0.899	–0.208	N/A^b^
	Change rate	0.054	0.005	0.137	N/A
**Correlation coefficient for** **COVID-19 RSV, r**					
	Mean weekly cases	0.152	0.657	0.536	N/A
	Change rate	0.428	0.679	0.516	N/A

^a^Chi-square test was applied for comparing two proportions and Mann-Whitney *U* test was applied for comparing two means. Spearman rank-order correlation coefficient was applied for public interest of telehealth and COVID-19. *P* value was set at a significant level of .05.

^b^N/A: not applicable.

### Correlations Between the First COVID-19 Pandemic Wave and After the Wave

The correlation coefficient between COVID-19 cases and the RSV of COVID-19 was 0.152, but for the RSV of telehealth, it was –0.300 overall (Table 1). When categorizing the period into the first wave and after, the correlation coefficient between the weekly COVID-19 cases and the RSV of telehealth was –0.899, but that between the cases and the RSV of COVID-19 was 0.657 in the first pandemic wave. After the first wave, the correlation between the weekly cases of COVID-19 and the RSV of telehealth was –0.208, but that between the cases and the RSV of COVID-19 was 0.536. When adjusting to week-over-week change rate, the correlation between COVID-19 cases and the RSV of COVID-19 was –0.428, but that between the cases and the RSV of telehealth was 0.054. Focusing only on the first wave, the correlation between the weekly case of COVID-19 and the RSV of COVID-19 was 0.679, but that between the cases and the RSV of telehealth was 0.005. With regard to change rate, after the first wave, the correlation between the weekly cases of COVID-19 and the RSV of COVID-19 was 0.516, but that between the cases and the RSV of telehealth was 0.137.

### Accessibility Rate of Telehealth and Regional Correlations

**Figure 3:**
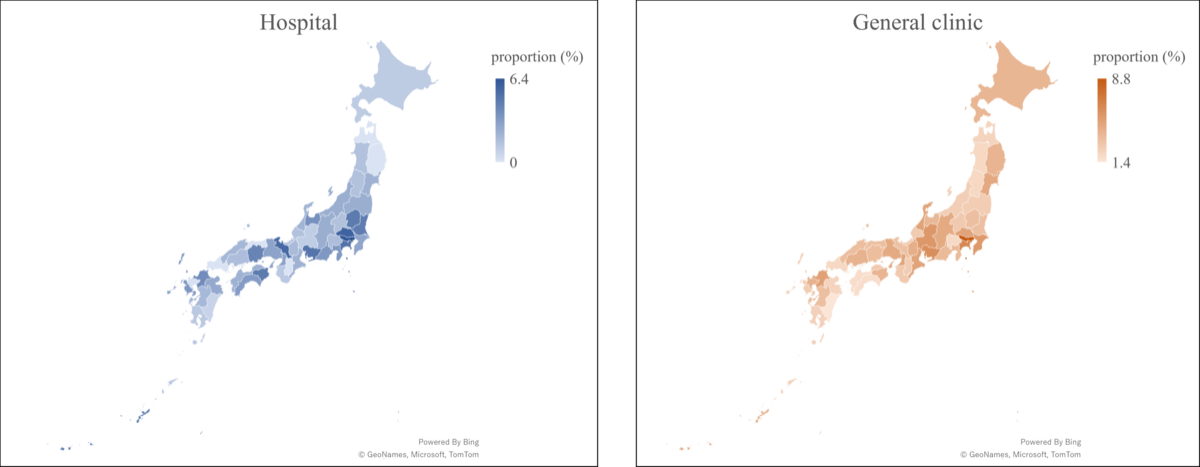
Accessibility rate of medical institution using mobile telehealth apps (in each prefecture).

**Figure 4:**
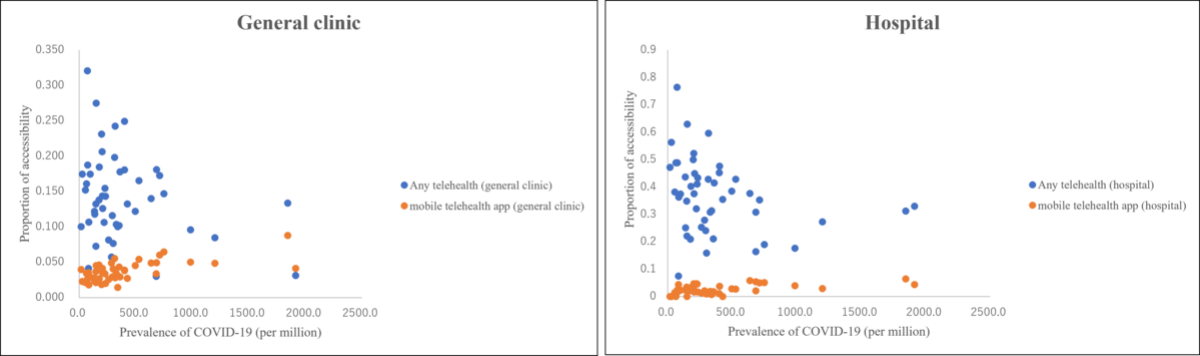
Correlation charts of telehealth accessibility.

The highest telehealth accessibility was observed in Tokyo with 6.4% in hospitals and 8.8% in general clinics (Figure 3; Multimedia Appendix 4). As shown in Table 2, the accessibility rate of mobile apps had a significant difference between epidemic and nonepidemic regions in both hospitals (3.8% vs 2%; *P=*.004) and general clinics (5.2% vs 3.1%; *P<*.001), but no significant difference was obtained for all telehealth modes in hospitals (32.8% vs 38.2%; *P=*.24) and general clinics (13.9% vs 14.3%; *P*=.79). In terms of the correlation between mobile app accessibility and the COVID-19 prevalence in each prefecture, the correlation coefficient was 0.497 in hospitals and 0.629 in general clinics (Figure 4).

**Table 2:** Comparison of basic characteristics of COVID-19 and telehealth accessibility in epidemic and nonepidemic regions.

Characteristics			Total regions (N=47)		Epidemic regions (n=13)		Nonepidemic regions (n=34)		*P* value	
**COVID-19/100,000 people (n), mean (95% CI)**										
	Cases	897.2 (709.6-1084.7)		1495 (1183.1-1806.9)		658.1 (478.6-837.6)		<.001		
	Severe cases	170.5 (119.0-221.9)		277.3 (160.4-394.2)		127.7 (76.4-179.1)		.006		
	Deaths	9.1 (7.0-11.1)		16.9 (12.5-21.3)		6 (4.7-7.2)		<.001		
**Accessibility of all telehealth (%), mean (95% CI)**										
	Hospital	36.7 (32.8-40.7)		32.8 (26.9-38.6)		38.2 (33.3-43.2)		.24		
	General clinic	14.2 (12.5-16.0)		13.9 (11.2-16.6)		14.3 (12.1-16.6)		.79		
**Accessibility of mobile apps (%), mean (95% CI)**										
	Hospital	2.5 (2.0-3.0)		3.8 (2.7-4.8)		2 (1.5-2.5)		.004		
	General clinic	3.6 (3.3-4.1)		5.2 (4.3-6.0)		3.1 (2.8-3.4)		<.001		
**Regional correlation between mobile app accessibility and COVID-19 prevalence, r**										
	Hospital	0.497^a^		0.457^a^		0.496^a^				
	General clinic	0.629^a^		0.566^a^		0.627^a^				

^a^Prefectures declared to be in a state of emergency in the first wave were categorized as epidemic regions (Tokyo, Kanagawa, Saitama, Chiba, Osaka, Hyogo, Fukuoka, Hokkaido, Ibaraki, Ishikawa, Gifu, Aichi, and Kyoto). Student *t* test was applied to compare two independent means for cases of COVID-19, severe cases, and deaths in epidemic and nonepidemic regions. Mann-Whitney *U* Test was applied to compare two independent means for mobile app accessibility rate in hospitals and general clinics. Spearman rank-order correlation was applied for regional correlation of COVID-19 prevalence and the proportion of telehealth availability in each prefecture. *P* value was set at a significant level of .05.

## Discussion

### Principal Findings

In Japan, the public interest in telehealth and COVID-19 cases had a weak negative correlation overall. When adjusting the weekly cases of COVID-19 to week-over-week change rate, the RSV of telehealth did not correlate. Regarding the accessibility of mobile telehealth apps, epidemic regions had significantly higher accessibility rates compared to nonepidemic regions. There was also a positive regional correlation in general clinics. To our knowledge, this study was the first study to investigate the public interest in telehealth, the accessibility of mobile telehealth apps, and the regional correlation between app accessibility and the COVID-19 prevalence in Japan.

### Comparison With Prior Work

In this study, the public interest in telehealth had a weak negative correlation with COVID-19 cases overall, but they were highly correlated in the first wave. This result was similar to previous studies [5,6]. When we adjusted to week-over-week change rate, the correlation coefficient was higher overall for the RSV of COVID-19. Therefore, the public interest may depend on how rapidly the COVID-19 cases increase rather than the weekly cases. Many other possible reasons might have influenced public interest. Considering the Japanese political background, the government announced to reimburse initial visits via telephones and videoconferencing from April 13, 2020. They also reported a nationwide list of telehealth-accessible medical institutions on April 25, 2020. These announcements by the government through the media caused interest among the health care providers and the public. Additionally, a decrease in the proportion of severe cases and deaths after the first wave might have affected public interest. A study reported that social behavior such as fear could be affected by the uncertainty of the severity and mortality rate [10]. Therefore, with a decrease in the risk of severe illness or death, the restrained patients might have revisited the hospital directly rather than via telehealth. However, the actual use of telehealth or the causation is unclear. Therefore, studies focusing on such aspects need to be conducted in the future.

By looking at previous studies, the accessibility of telehealth increased after the COVID-19 in the United States [11]. In Japan, telehealth accessibility for clinical use was about 1% in small hospitals and general clinics before COVID-19 [3]. This was because treating patients who lived in rural areas was the main purpose of telehealth before COVID-19 [12]. Although the accessibility rate was much higher than previous telehealth use rate in Japan [3], the accessibility rate of mobile apps is still limited (2.5% in hospitals and 3.6% in general clinics). Another study done in Japan showed that the use of telehealth increased among younger people from August to September 2020, compared to April 2020 [13]. Although the study showed an increase in telehealth use, it was a web-based survey, and the observed period was short. Therefore, future studies considering the actual telehealth use after COVID-19 using claims data are necessary.

There was a regional correlation between telehealth accessibility using mobile apps and the COVID-19 cases in Japan, especially for general clinics. This suggests that general clinics using mobile apps for telehealth are more accessible in areas where people must self-restrain under the governmental order. We suspect that general clinics were more likely to apply telehealth to continue medical care under the circumstances. A previous study supports our findings that telemedicine use was higher among those living in urban areas compared to rural areas [13]. Since most of the epidemic regions cover the urban areas of Japan, we believe that similar results were obtained in this study. However, a weak negative regional correlation was observed using all telehealth methods. Since mobile apps are more costly in terms of implementation and management compared to telephones, they may have already been adopted in most medical institutions. In future studies, a comparison of actual telehealth use and the clinical effect of telephones and mobile apps should be investigated.

### Limitations

Several limitations need to be considered in this study. First of all, causation was not investigated in this study. A regression approach should be applied to define the causal relationship. Second, the use of Google Trends does not reflect the entire public interest. However, approximately 70% of all searches are conducted via Google, and it is the most used search engine in Japan [14]. Moreover, the internet use among Japanese people varies by age, and the older population are more likely to have less accessibility to the internet. Therefore, the public interest may not have reflected the actual Japanese population. Furthermore, the keywords chosen in this study may not be appropriate. We believe that the terms “corona” and “online-shinryou” were appropriate based on previous research and the RSV from Google Trend (Multimedia Appendix 1) [8]. Further studies comparing potential keywords are necessary to define the most appropriate search terms. Third, the groups chosen for comparisons such as the periods of the epidemic waves and the prefectures as epidemic regions might be unsuitable. However, we believe that separating the period as first wave and after the first wave was suitable for understanding the differences. Fourth, infectious disease and public interest are said to have lagged in searches among the general population or in reporting confirmed cases for surveillance [15]. However, this study averaged COVID-19 cases by week instead of daily cases. Therefore, the lags have a limited effect on the results. In addition, there is a time lag for COVID-19 infection, onset, admission, and death [16], and the decision for admission differs by hospital. These aspects were not investigated in this study since we believe that they do not have an immediate effect on public interest. Furthermore, the definition of severe cases may be different between prefectures and JMHLW, since some prefectures did not count patients in the intensive care unit without extracorporeal membrane oxygenation or ventilator. Therefore, the comparison may not be robust. Fifth, we could not investigate the user characteristics of Google Trends or telehealth accessibility. Since the data only show the RSV and the absence of telehealth implementation, further study using real-world data is needed. Finally, the accessibility of mobile telehealth apps through company data does not cover all mobile app use. There are more companies supplying such services that are used by medical institutions. However, we searched the major companies and believe that the results were comprehensive enough to understand the accessibility. Moreover, quite a few medical institutions applied free communication apps such as “Line,” “Zoom,” and “Skype.” These apps are easier to use but have security issues such as protecting personal information [17]. Since those apps were not telehealth apps, we included them in all telehealth groups. We believe that the accessibility rate of mobile apps did not affect the results.

### Conclusions

Although there was no positive long-term correlation between public interest in telehealth and COVID-19, there was a regional correlation between mobile telehealth app accessibility and COVID-19 prevalence in Japan, especially for general clinics. We also revealed that epidemic regions had higher mobile telehealth app accessibility. Further studies about the actual use of telehealth and its effect after the COVID-19 pandemic are necessary.

## Appendix

Multimedia Appendix 1 Google Trends Search term (keywords).

Multimedia Appendix 2 Telehealth accessibility in each prefecture of Japan.

Multimedia Appendix 3 Data sample of telehealth accessibility (Tokyo).

Multimedia Appendix 4 Endemic region (prefectures).

## Abbreviations

JMHLW: Japanese Ministry of Health, Labour and Welfare
RSV: relative search volume
