# Supplementary material for: Public Interest and Accessibility of Telehealth in Japan: Retrospective Analysis Using Google Trends and National Surveillance
Source: JMIR Form Res. 2022 Sep 14;6(9):e36525. doi: 10.2196/36525 (PMC9520390; doi:10.2196/36525)
Supplement: Multimedia Appendix 2 [file formative_v6i9e36525_app2.docx]

| **Mobile telemedicine app introduced medical institutions** | | | | |
| --- | --- | --- | --- | --- |
|  | All Hospitals (n=109,068) | | |  |
|  | Hospitals |  | General Clinics |  |
|  | (n=8,540) |  | (n=100,528) | P-Value |
| *Prefecture n, %* |  |  |  |  |
| Hokkaido | 6/552 (1.1) |  | 132/3397 (3.9) | 0.001 |
| Aomori | 0/94 (0.0) |  | 20/877 (2.3) | 0.001 |
| Iwate | 0/91 (0.0) |  | 35/879 (4.0) | 0.001 |
| Miyagi | 3/138 (2.2) |  | 77/1671 (4.6) | 0.001 |
| Akita | 1/68 (1.5) |  | 17/802 (2.1) | 0.001 |
| Yamagata | 1/68 (1.5) |  | 19/919 (2.1) | 0.001 |
| Fukushima | 3/126 (2.4) |  | 37/1346 (2.7) | 0.001 |
| Ibaraki | 8/173 (4.6) |  | 59/1749 (3.4) | 0.001 |
| Tochigi | 5/106 (4.7) |  | 50/1460 (3.4) | 0.001 |
| Gunma | 2/130 (1.5) |  | 45/1551 (2.9) | 0.001 |
| Saitama | 20/342 (5.8) |  | 214/4378 (4.9) | 0.58 |
| Chiba | 8/289 (2.8) |  | 207/3818 (5.4) | 0.053 |
| Tokyo | 41/638 (6.4) |  | 1206/13707 (8.8) | 0.01 |
| Kanagawa | 17/336 (5.1) |  | 439/6820 (6.4) | 0.20 |
| Niigata | 3/127 (2.4) |  | 44/1671 (2.6) | 0.82 |
| Toyama | 4/107 (3.7) |  | 29/762 (3.8) | 0.96 |
| Ishikawa | 2/94 (2.1) |  | 43/872 (4.9) | 0.20 |
| Fukui | 1/67 (1.5) |  | 16/573 (2.8) | 0.52 |
| Yamanashi | 1/60 (1.7) |  | 14/698 (2.0) | 0.84 |
| Nagano | 3/127 (2.4) |  | 71/1574 (4.5) | 0.23 |
| Gifu | 1/98 (1.0) |  | 88/1587 (5.5) | 0.04 |
| Shizuoka | 6/175 (3.4) |  | 101/2732 (3.7) | 0.77 |
| Aichi | 16/323 (5.0) |  | 328/5455 (6.0) | 0.30 |
| Mie | 2/93 (2.2) |  | 74/1519 (4.9) | 0.17 |
| Shiga | 1/57 (1.8) |  | 47/1091 (4.3) | 0.29 |
| Kyoto | 9/165 (5.5) |  | 82/2451 (3.3) | 0.18 |
| Osaka | 15/513 (2.9) |  | 412/8533 (4.8) | 0.02 |
| Hyogo | 10/348 (2.9) |  | 233/5125 (4.5) | 0.11 |
| Nara | 0/79 (0.0) |  | 33/1215 (2.7) | 0.14 |
| Wakayama | 1/83 (1.2) |  | 30/1025 (2.9) | 0.36 |
| Tottori | 0/43 (0.0) |  | 17/497 (3.4) | 0.21 |
| Shimane | 1/49 (2.0) |  | 25/715 (3.5) | 0.54 |
| Okayama | 7/161 (4.3) |  | 58/1650 (3.5) | 0.70 |
| Hiroshima | 4/237 (1.7) |  | 106/2563 (4.1) | 0.053 |
| Yamaguchi | 0/145 (0.0) |  | 26/1240 (2.1) | 0.08 |
| Tokushima | 5/107 (4.7) |  | 29/727 (4.0) | 0.77 |
| Kagawa | 2/88 (2.3) |  | 24/825 (2.9) | 0.68 |
| Ehime | 3/135 (2.2) |  | 22/1226 (1.8) | 0.77 |
| Kochi | 4/124 (3.2) |  | 10/549 (1.8) | 0.33 |
| Fukuoka | 18/459 (3.9) |  | 237/4713 (5.0) | 0.21 |
| Saga | 1/101 (0.1) |  | 28/691 (4.1) | 0.10 |
| Nagasaki | 5/149 (3.4) |  | 37/1371 (2.7) | 0.68 |
| Kumamoto | 4/211 (1.9) |  | 50/1469 (3.4) | 0.24 |
| Oita | 4/155 (2.6) |  | 22/949 (2.3) | 0.84 |
| Miyazaki | 1/137 (0.7) |  | 13/899 (1.4) | 0.47 |
| Kagoshima | 3/241 (1.2) |  | 35/1374 (2.5) | 0.18 |
| Okinawa | 4/91 (4.4) |  | 37/901 (4.1) | 0.96 |
