# Supplementary material for: Public Interest and Accessibility of Telehealth in Japan: Retrospective Analysis Using Google Trends and National Surveillance
Source: JMIR Form Res. 2022 Sep 14;6(9):e36525. doi: 10.2196/36525 (PMC9520390; doi:10.2196/36525)
Supplement: Multimedia Appendix 3 [file formative_v6i9e36525_app3.pdf]

電話や情報通信機器を用いて診療を実施する医療機関の一覧（東京都）

	基本情報					事務連絡に基づく対応について				
	施設名	郵便番号	住所（都道府県から記載）	電話番号	ウェブサイトURL	初診の電話等を用いた診療の実施の有無	再診の電話等を用いた診療の実施の有無	対応診療科	担当医師名	対面診療が必要と判断した場合に連携する医療機関名（複数ある場合は複数、住所も併せて記載）
1	秋葉原あつたアレルギー呼吸器内科クリニック	101-0033	東京都千代田区神田岩本町１番地５<br>清水ビル６階	03-3253-4159	https://atsuta-clinic.jp		○	呼吸器内科／アレルギー科／内科	熱田　了	三井記念病院（東京都千代田区神田和泉町1）<br>日本大学病院（東京都千代田区神田駿河台1-6）<br>杏雲堂病院（東京都千代田区神田駿河台1-8）<br>東京医科歯科大学医学部附属病院（東京都文京区湯島1-5-45）<br>順天堂大学医学部附属病院（東京都文京区本郷2-1-1）
2	秋葉原スキンクリニック	101-0021	東京都千代田区外神田四丁目６番７号<br>カンダエイトビル２階　３階	03-3256-1212	https://www.akihabara-skin.com/		○	皮膚科	堀内　祐紀／矢田　佳子／明石玲／関　詠姿／西山　有希子／向久保　寿恵／吉田　珠英／上松　ふみ／吉田昇子／川上　倫	左記施設
3	秋葉原ＤＥＭ内科クリニック	101-0025	東京都千代田区神田佐久間町三丁目２８番地<br>星野ビル２階	03-5846-9876	www.akibademclinic.jp		○	内科／糖尿病内分泌内科／代謝内科	内村　功／諸星政治	左記施設<br>東京医科歯科大学医学部附属病院（東京都文京区湯島1-5-45）<br>三井記念病院（東京都千代田区神田和泉町1）<br>三楽病院（東京都千代田区神田駿河台2-5）
4	秋葉原メンタルクリニック	101-0027	東京都千代田区神田平河町４番地<br>渡辺ビル６階	03-5833-6852	https://www.akihabara-mental-clinic2019.com/		○	精神科	安藤　智道	左記施設
5	浅井医院	102-0083	東京都千代田区麹町三丁目７番地２４<br>東急ドエルグラフィオ麹町１０１	03-3261-0453	www.asai-iin.com		○	皮膚科／内科／外科	戸田　素子	東京逓信病院（東京都千代田区富士見2-14-23）
6	あたまと体のヘルスケアクリニック神田	101-0041	東京都千代田区神田須田町一丁目１０番地４２<br>エスペランサ神田須田町２階	03-3254-0056	http://atamato-karada.com	○	○	脳神経外科／内分泌内科	池田　秀敏	総合南東北病院（福島県郡山市八山田7-115）
7	アルツクリニック東京	100-0005	東京都千代田区丸の内一丁目７番１２号<br>サピアタワー７階	03-3212-0003			○	メンタルクリニック（精神科）／内科／神経内科	新井　平伊／出雲　博子	
8	淡路町眼科	101-0041	東京都千代田区神田須田町1-2-7　淡路町駅前ビル2F	03-3526-6086	https://www.awajichoganka.com/		○	眼科	由井　あかり	
9	飯田橋駅前さくら坂クリニック	102-0071	東京都千代田区富士見二丁目１０番２号<br>飯田橋グラン・ブルーム　サクラテラス３階	03-3239-1112	https://iidabashi-hifuka.com		○	皮膚科	小谷　和弘	

電話や情報通信機器を用いて診療を実施する医療機関の一覧（東京都）

	基本情報					事務連絡に基づく対応について				
	施設名	郵便番号	住所（都道府県から記載）	電話番号	ウェブサイトURL	初診の電話等を用いた診療の実施の有無	再診の電話等を用いた診療の実施の有無	対応診療科	担当医師名	対面診療が必要と判断した場合に連携する医療機関名（複数ある場合は複数、住所も併せて記載）
10	飯田橋東口内科心療内科診療所	102-0072	東京都千代田区飯田橋四丁目９番９号<br>第７田中ビル８階	03-6260-9863	http://www.iidabashi-shinryounaika.jp		○	内科／心療内科	下平　智史	東京逓信病院（東京都千代田区富士見2-14-23）<br>三井記念病院三井記念病院（東京都千代田区神田和泉町1）<br>東京新宿メディカルセンター
11	飯田橋皮膚科スキンクリニック	102-0072	東京都千代田区飯田橋飯田橋四丁目２番２号<br>宮下ビル１Ｆ	03-3234-0140	https://iskc.jp		○	皮膚科	河野　志穂美	
12	飯田橋レディースクリニック	102-0072	東京都千代田区飯田橋三丁目１０番１０号<br>ガーデンエアータワー２階	03-3556-8333	http://www.iidabashi-ladies-clinic.com		○	産婦人科	岡野　浩哉	
13	市ヶ谷駅前診療所	102-0076	東京都千代田区五番町2<br>横山ビル1階	03-3234-5021	https://ichigaya-shinryosyo.com/	○	○	内科／皮膚科	泉田　秀輝	東京逓信病院（東京都千代田区富士見2-14-23）
14	一般財団法人健康医学協会附属霞が関ビル診療所	100-6003	東京都千代田区霞が関霞が関三丁目２番５号<br>霞が関ビル３階	03-3581-6031			○	内科	鈴木　順一／今井　道代／二宮栄司／田辺　紀子	虎の門病院
15	一般財団法人健康医学協会附属東都クリニック	102-0094	東京都千代田区紀尾井町4-1<br>ホテルニューオータニガーデンタワー２階	03-3239-0301	https://www.kenkoigaku.or.jp		○	[illegible]内科／消化器内科／循環器内科／糖尿病内科／呼吸器内科／心療内科／外科／乳腺外科／婦人科／眼科／耳鼻咽喉科／泌尿器[illegible]	[illegible]（院長）／佐原由華子（副院長）<br>【内科】四方千裕／尾仲章男／上野由起子／岡芙久子／白鳥敬子／遠藤康夫／泉二登志子／鈴木順一／小西敏郎／勝木美[illegible]	
16	一般財団法人全日本労働福祉協会　九段クリニック	102-0073	東京都千代田区九段北1-9-5<br>朝日九段マンション１階１号室	03-3222-0071			○	内科／婦人科	西原　浩憲／上田医師／木村緑	
17	一般財団法人日本がん知識普及協会付属有楽町電気ビルクリニック	100-0006	東京都千代田区有楽町一丁目７番１号<br>有楽町電気ビル北館１０階	03-3213-0091<br>（代）			○	内科	川島　隆二	
18	医療法人財団小畑会浜田病院	101-0062	東京都千代田区神田駿河台二丁目５番地	03-5280-1166	obatakai.or.jp		○	産婦人科／小児科／不妊科／乳腺外科	小畑　清一郎／知念　香奈／清水　俊明／合阪幸三／平田　哲也／平池　修／土屋　富士子／小田　美規／原田　美由紀	

電話や情報通信機器を用いて診療を実施する医療機関の一覧（東京都）

	基本情報					事務連絡に基づく対応について				
	施設名	郵便番号	住所（都道府県から記載）	電話番号	ウェブサイトURL	初診の電話等を用いた診療の実施の有無	再診の電話等を用いた診療の実施の有無	対応診療科	担当医師名	対面診療が必要と判断した場合に連携する医療機関名（複数ある場合は複数、住所も併せて記載）
19	医療法人財団駿愛会駿河台診療所	101-0062	東京都千代田区神田駿河台二丁目１番地４５ ニュー駿河台ビル５階	03-3219-1121	http://www.sunaikai.jp	○	○	内科	塩澤　宏和	杏雲堂病院（東京都千代田区神田駿河台1-8）<br>三楽病院（東京都千代田区神田駿河台2-5）<br>日本大学病院（東京都千代田区神田駿河台1-6）<br>東海大学医学部付属東京病院（東京都渋谷区代々木1-2-5）
20	医療法人財団日睡会御茶ノ水呼吸ケアクリニック	101-0062	東京都千代田区神田駿河台2-8 瀬川ビルディング３Ｆ	03-3292-3838	https://.sas-care.jp		○	呼吸器内科（当院CPAP処方患者の定期受診に限る）	村田　朗	
21	医療法人財団福音医療会神田キリスト教診療所	101-0053	東京都千代田区神田美土代町７番地４ 東英美土代ビル５階・６階	03-3294-0808	www.fukuin.net		○	内科／消化器内科／循環器内科／泌尿器科／皮膚科	小西　弘一	
22	医療法人財団 神尾記念病院	101-0063	東京都千代田区神田淡路町2丁目25番地	03-3253-3351	http://www.kamio.org/		○	耳鼻咽喉科 皮膚科	各主治医	
23	医療法人財団医親会海上ビル診療所	100-0005	東京都千代田区丸の内一丁目２番１号 東京海上日動ビルディング新館３階	03-3212-7690	http://www.kbclinic.or.jp/		○	内科／消化器内科／循環器内科／整形外科／皮膚科／婦人科／眼科／泌尿器科／呼吸器内科／リウマチ科／代謝内科	各担当医師	
24	医療法人社団アネモス会荻本医院	101-0047	東京都千代田区内神田二丁目７番１４号 山手ビル３号館４Ｆ	03-3255-4730	www.ogimoto.com		○	精神科／内科／心療内科	荻本　芳信	その都度、患者と話し合い、ご本人が納得した医療機関に診療情報提供を認め御高診をお願いする
25	医療法人社団お茶会　お茶の水循環器内科	101-0051	東京都千代田区神田神保町一丁目４番地 神保町１－４ビル８階	03-5577-6513	ochanomizunaik.com		○	循環器内科	五十嵐　健祐	左記施設
26	医療法人社団ひのき会証クリニック神田	101-0052	東京都千代田区神田小川町三丁目８番地 中北ビル２階	03-3292-7701	http://www.akashi-clinic.com		○	漢方内科／内科／漢方消化器内科／漢方精神科	伊藤　隆／岡本英輝／檜山　幸孝／横山　浩一	
27	医療法人社団益生会神保町代謝クリニック	101-0051	東京都千代田区神田神保町二丁目９番地１ 神田神保町メディカルモール５階	03-5210-3412	http://www1.tcn-catv.ne.jp/taisha/		○	内科（糖尿病）	益子　茂	東京逓信病院（東京都千代田区富士見2-14-23）

電話や情報通信機器を用いて診療を実施する医療機関の一覧（東京都）

	基本情報					事務連絡に基づく対応について				
	施設名	郵便番号	住所（都道府県から記載）	電話番号	ウェブサイトURL	初診の電話等を用いた診療の実施の有無	再診の電話等を用いた診療の実施の有無	対応診療科	担当医師名	対面診療が必要と判断した場合に連携する医療機関名（複数ある場合は複数、住所も併せて記載）
28	医療法人社団遠藤クリニック	101-0054	東京都千代田区神田錦町一丁目８番地１階	03-3295-1013	www.endo.or.jp	○	○	内科	遠藤　素夫	
29	医療法人社団響中澤プレスセンタークリニック	100-0011	東京都千代田区内幸町2-2-1<br>日本プレスセンタービル地下１階	03-3500-1101	https://nakazawa-pcc.net		○	外科／（内科）	中澤　英樹	虎の門病院
30	医療法人社団慶洋会ケイアイ飯田橋クリニック	102-0072	東京都千代田区飯田橋三丁目１０番１０号<br>ガーデンエアータワー２階	03-3239-2777	kiclinic.jp/kiic	○	○	内科／整形外科	杉村　香織（内科）／海苔　聡（整形外科）	ＪＣＨＯ東京新宿メディカルセンター
31	医療法人社団互酬会水道橋東口クリニック	101-0061	東京都千代田区神田三崎町一丁目３番１２号<br>水道橋ビル９階	03-3292-1270	https://www.suidobashi-e-cl.jp		○	内科／老年内科	辻　彼南雄	三井記念病院（東京都千代田区神田和泉町1）<br>東京逓信病院（東京都千代田区富士見2-14-23）<br>三楽病院（東京都千代田区神田駿河台2-5）<br>九段坂病院（東京都千代田区九段南1-6-12）<br>杏雲堂病院（東京都千代田区神田駿河台1-8）
32	医療法人社団公颯会　東商ビル診療所	100-0005	東京都千代田区丸の内三丁目２番２号<br>丸の内二重橋ビルディング東京商工会議所ビル地下１階	03-3283-7781	https://www.tosho-clinic.com/		○	耳鼻咽喉科／内科	杉村　浩美／久武　朋子／上田晴美	
33	医療法人社団高典会飯田橋クリニック	102-0072	東京都千代田区飯田橋四丁目６番９号<br>ＳＴビル２階	03-3222-6461		○	○	皮膚科／形成外科／アレルギー科	高橋　典大	東京逓信病院（東京都千代田区富士見2-14-23）<br>九段坂病院（東京都千代田区九段南1-6-12）<br>帝京大学医学部附属病院（東京都板橋区加賀2-11-1）
34	医療法人社団榊原厚生会榊原サピアタワークリニック	100-0005	東京都千代田区丸の内一丁目７番１２号<br>サピアタワー７階	03-5288-0011	http://sapiatower-clinic.jp		○	内科／循環器内科／消化器内科／糖尿病代謝科	北原　公一／鎌西　亮子／関根百合子／秋本恵子／阿部　麻希子	
35	医療法人社団桜メディスン有楽町桜クリニック	100-0006	東京都千代田区有楽町一丁目７番１号<br>有楽町電気ビル南館７階７５８区	03-6914-5333			○	精神科／心療内科	神山　昭男	左記施設
36	医療法人社団山岡クリニック	102-0083	東京都千代田区麹町麹町四丁目３番地<br>麹町富士ビル２階	03-3264-5160	yamaoka-clinic.com	○	○	内科／小児科／皮膚科	鈴木　淳子／今田　義夫／山岡周平／平山　真帆	東京逓信病院（東京都千代田区富士見2-14-23）<br>九段坂病院（東京都千代田区九段南1-6-12）

電話や情報通信機器を用いて診療を実施する医療機関の一覧（東京都）

	基本情報					事務連絡に基づく対応について				
	施設名	郵便番号	住所（都道府県から記載）	電話番号	ウェブサイトURL	初診の電話等を用いた診療の実施の有無	再診の電話等を用いた診療の実施の有無	対応診療科	担当医師名	対面診療が必要と判断した場合に連携する医療機関名（複数ある場合は複数、住所も併せて記載）
37	医療法人社団慈鴻会麹町内科	102-0083	東京都千代田区麹町二丁目２番地３１ 麹町サンライズビル２０１号室	03-5276-2082	koujimachinaika.com		○	内科／皮膚科	河野　英雄	東京医科歯科大学医学部附属病院（東京都文京区湯島1-5-45）<br>国立国際医療研究センター（東京都新宿区戸山1-21-1）<br>九段坂病院（東京都千代田区九段南1-6-12）<br>医療法人社団あんしん会四谷メディカルキューブ（東京都千代田区二番町7-7）
38	医療法人社団授生会杉村レディースクリニック	102-0076	東京都千代田区五番町２番地 横山ビルＢ１階	03-3264-8686	https://www.ivf-baby.org	○	○	婦人科	赤星　晃一／杉村　和男	
39	医療法人社団順成会　いずみレディスクリニック	101-0025	東京都千代田区神田佐久間町二丁目１８番地５ アークビル６階	03-3862-7733	izumi-ladies.jp	○	○	婦人科	柿木　成子	三井記念病院（東京都千代田区神田和泉町1）<br>三楽病院（東京都千代田区神田駿河台2-5）
40	医療法人社団仁成守会 秋元医院	101-0042	東京都千代田区神田東松下町１９番地 秋元ビル１階２階	03-5297-5888	http://www.akimotoiiw.jp/		○	内科／泌尿器科	秋元　達雄／秋元　成太／小山利香	
41	医療法人社団晴美会麹町リバース今井整形外科クリニック	102-0083	東京都千代田区麹町四丁目２番地１２ クーアハウスビル１階・２階	03-3261-0700	http://kojimachi-rebirth.imai-seikei.jp/		○	整形外科／漢方科	今井　大	
42	医療法人社団清湘会聖橋クリニック	101-0063	東京都千代田区神田淡路町2-105 ワテラスアネックス５階	03-5298-8612			○	内科／糖尿病内科	下田　研二	
43	医療法人社団西端耳鼻咽喉科	100-0006	東京都千代田区有楽町2-10-1 東京交通会館３階	03-3214-9047	http://www.nishihata-ent.or.jp	○	○	耳鼻咽喉科／アレルギー科	西端　慎一	東京逓信病院（東京都千代田区富士見2-14-23）
44	医療法人社団千禮会千代田漢方内科クリニック	101-0052	東京都千代田区平河町一丁目７番１１号　第二大盛丸平河町ビル２階	03-5357-1575		○	○	心療内科／アレルギー科／皮膚科／婦人科／漢方内科／泌尿器科	信川　益明／團　茂樹	日本大学病院（千代田区神田駿河台1-6）
45	医療法人社団爽和会お茶の水駿河台クリニック	101-0062	東京都千代田区神田駿河台2-1-47 廣瀬お茶の水ビル１・２階	03-5280-9122	www.gazo.or.jp		○	内科／消化器内科	中元　和也／杉本　貴史／小林　敏	

電話や情報通信機器を用いて診療を実施する医療機関の一覧（東京都）

	基本情報					事務連絡に基づく対応について				
	施設名	郵便番号	住所（都道府県から記載）	電話番号	ウェブサイトURL	初診の電話等を用いた診療の実施の有無	再診の電話等を用いた診療の実施の有無	対応診療科	担当医師名	対面診療が必要と判断した場合に連携する医療機関名（複数ある場合は複数、住所も併せて記載）
46	医療法人社団多久美会みきクリニック市ヶ谷	102-0074	東京都千代田区九段南四丁目３番７号翠ビルＢ１Ｆ	03-3234-5421	http://www.miki-beauty.com	○	○	皮膚科／形成外科	多久嶋　美紀／菱沼　茂之	東京逓信病院（東京都千代田区富士見2-14-23）<br>杏林大学医学部附属病院（東京都三鷹市新川6-20-2）
47	医療法人社団大政クリニック	102-0083	東京都千代田区麹町四丁目３番地麹町富士ビル２階	03-3556-7875			○	胃腸科／肛門科	大政　良二	
48	医療法人社団中興会内外クリニック	100-0006	東京都千代田区有楽町０番地銀座インズ３	03-3562-5971		○	○	内科	中村　治道／奥脇　純子／石松隆子	患者地元の病院<br>聖路加国際病院（東京都中央区明石町9-1）<br>東京逓信病院（東京都千代田区富士見2-14-23）<br>三井記念病院（東京都千代田区神田和泉町1）
49	医療法人社団八橋会飯田橋耳鼻咽喉科医院	102-0071	東京都千代田区富士見二丁目１１番１０号　レオ飯田橋ビル６階	03-3230-1916	iidabashijibika.com	○	○	耳鼻咽喉科	八幡　則子／和多田　有紀子	東京逓信病院（東京都千代田区富士見2-14-23）<br>JCHO東京新宿メディカルセンター（東京都新宿区津久戸町5-1）<br>日本大学病院（東京都千代田区神田駿河台1-6）
50	医療法人社団碧桜秋葉原駅クリニック	101-0025	東京都千代田区神田佐久間町二丁目１番地大原ビル４Ｆ	03-5835-2860	www.ekic.jp		○	内科	大和田　潔／佐々木　欧	
51	医療法人社団芳雅会　内神田皮膚科	101-0047	東京都千代田区内神田2-13-7石原ビル１階	03-6260-9515	http://kanda-hifuka.jp		○	皮膚科	白井　明／森悦子／遠藤　幸紀／白砂　智紹	三井記念病院（東京都千代田区神田和泉町1）<br>東京逓信病院（東京都千代田区富士見2-14-23）<br>東京大学医学部附属病院
52	医療法人社団<br>丸の内クリニック	100-0005	東京都千代田区丸の内１－６－２<br>新丸の内センタービルディング４Ｆ	03-5223-8822	http://marunouchi-c.org/		○	内科<br>皮膚科<br>婦人科	石川 隆、松田梨恵<br>金子 健彦<br>熊谷 万紀子、<br>神津 円	
53	医療法人社団裕健会神田クリニック	101-0047	東京都千代田区内神田二丁目４番１号<br>神田メディカルビル地下１階、２～８階	03-3252-0621	https://www.kanda.or.jp		○	内科／循環器内科／消化器内科／糖尿病内科	和田　理恵／馬渕　浩輔／小黒雅子／酒井　伸／宮本　正章／吉田　任子／時田　美和	
54	医療法人社団雄仁会　メディカルケア大手町	100-0004	東京都千代田区大手町二丁目２番１号<br>新大手町ビル地下１階	03-6262-1666	www.medcare-tora.com/otemachi/		○	精神科／心療内科	五十嵐　良雄／森田　哲也／長尾　博司	メディカルケア虎ノ門（東京都港区虎ノ門1-16-16　虎ノ門一丁目MGビル3階・4階）

電話や情報通信機器を用いて診療を実施する医療機関の一覧（東京都）

	基本情報					事務連絡に基づく対応について				
	施設名	郵便番号	住所（都道府県から記載）	電話番号	ウェブサイトURL	初診の電話等を用いた診療の実施の有無	再診の電話等を用いた診療の実施の有無	対応診療科	担当医師名	対面診療が必要と判断した場合に連携する医療機関名（複数ある場合は複数、住所も併せて記載）
55	医療法人社団樵会九段ごみぶちクリニック	102-0073	東京都千代田区九段北二丁目３番１号<br>九段増田ビル２階	03-3263-0161	http://gomibuchi-clinic.com		○	精神科／心療内科	五味渕　隆志	適宜
56	医療法人社団誠和堂丸山医院	101-0061	東京都千代田区神田三崎町二丁目２０番１号<br>第二石川ビル５階	03-3261-9679	https://www.maruyama-clinic.or.jp		○	耳鼻咽喉科／気管食道科／アレルギー科	丸山　毅	
57	医療法人社団滝医院	102-0074	東京都千代田区九段南四丁目３番１号	03-3264-3101	http://www.nt.pial.jp/taki-iin/	○	○	内科／小児科／糖尿病内科	滝　正彦／滝雅史／滝　ゆうこ	グローバルヘルスケアクリニック（東京都千代田区麹町3-12　麹町MSビル1階）<br>東京逓信病院（東京都千代田区富士見2-14-23）<br>日本大学病院（東京都千代田区神田駿河台1-6）
58	医療法人社団糖正会飯田橋メディカルクリニック	102-0072	東京都千代田区飯田橋三丁目４番１０号<br>飯田橋エルシスビル２Ｆ	03-3237-7633	https://iidabashimedical.com		○	糖尿病内科	丹羽　正孝	
59	医療法人社団みすまるのさと会アイ・ローズクリニック	100-0013	東京都千代田区霞が関一丁目４番１号<br>日土地ビル１階	03-3503-3900			○	眼科	安達　京	
60	医療法人聖光園細野診療所東京診療所	100-0006	東京都千代田区有楽町有楽町一丁目２番１４号<br>紫ビル４階	03-5251-3637	www.hosonokanpo.com		○	内科／漢方	細野　孝郎	
61	医療法人社団恒正会<br>そねクリニック丸の内	100-0005	東京都千代田区丸の内一丁目8番2号 鉄鋼ビルディング地下1階	03-6212-3888	https://www.soneclinic-marunouchi.com/		○	内科	種市　春仁	
62	医療法人社団　縁風会<br>ユアクリニックお茶の水	101-0062	東京都千代田区神田駿河台2-4<br>日健ビル3F	03-3259-1190	https://yourclinic.jp	○	○	内科<br>皮膚科	國廣　崇<br>井上　眞璃子<br>宮内　隆政<br>杉山　美紀子	東京医科歯科大学医学部附属病院(文京区湯島1-5-45)<br>日本大学病院(千代田区神田駿河台1-6)
63	医療法人社団　縁風会<br>ユアクリニック秋葉原	101-0021	東京都千代田区外神田4-9-2<br>千住ビル4F	03-3256-1180	https://yourclinicakb.jp	○	○	内科<br>小児科	杉原　桂<br>宮内　隆政<br>松野　由以<br>渡邉　太郎<br>安原　務	日本大学病院(千代田区神田駿河台1-6)<br>東京逓信病院（千代田区富士見2-14-23）

電話や情報通信機器を用いて診療を実施する医療機関の一覧（東京都）

	基本情報					事務連絡に基づく対応について				
	施設名	郵便番号	住所（都道府県から記載）	電話番号	ウェブサイトURL	初診の電話等を用いた診療の実施の有無	再診の電話等を用いた診療の実施の有無	対応診療科	担当医師名	対面診療が必要と判断した場合に連携する医療機関名（複数ある場合は複数、住所も併せて記載）
64	医療法人社団慶翔会神田須田町アイクリニック	101-0041	東京都千代田区神田須田町1-3-1<br>須田町ビル２階	03-5207-2882	http://kanda-eye.com	○	○	眼科	吉野　麻未	両国眼科クリニック（東京都墨田区両国4-33-12　グラン・アルブル両国1F)<br>医療法人社団慶翔会飯田橋眼科クリニック（東京都千代田区飯田橋三丁目１０番１０号　ガーデンエアータワー２階）
65	医療法人社団青十字会日比谷国際クリニック	100-0011	東京都千代田区内幸町二丁目２番３号<br>日比谷国際ビル地下１階	03-3503-3430	www.hibiyakokusai.or.jp	○	○	内科	馬島／福田／櫻岡	
66	医療法人社団青十字会日比谷国際クリニック法務省共済組合診療所	100-0013	東京都千代田区霞が関一丁目１番１号<br>中央合同庁舎第６号館Ａ棟１７階	03-3592-4055			○	内科	鈴木　直美	
67	医療法人社団正誠会林医院	101-0021	東京都千代田区外神田三丁目７番１号<br>１階・２階	03-3253-7436	https://hayashi-clinic.com	○	○	内科／消化器科	林　久太佳	三井記念病院（東京都千代田区神田和泉町1）<br>東京医科歯科大学医学部附属病院（東京都文京区湯島1-5-45）
68	医療法人社団医新会　神田医新クリニック	101-0032	東京都千代田区岩本町2-2-13<br>1～7階	03-5833-3240	https://www.ishin-kai.or.jp		○	泌尿器科／内科	藤原　博通	
69	医療法人社団知慎会ＪＴＫクリニック	102-0083	東京都千代田区麹町4-1-5<br>麹町志村ビル２階	03-6261-6386	https://www.jtkclinic.com	○	○	内科	小笠原　均	
70	医療法人社団滉志会瀬田クリニック東京	101-0062	東京都千代田区神田駿河台2-1-45<br>ニュー駿河台ビル３階	03-5280-0086	hhtp://www.j-immunother.com	○	○	内科	瀧本　理修／後藤　重則／神垣隆	
71	医療法人社団渡邊内科	100-0004	東京都千代田区大手町一丁目７番２号<br>東京サンケイビル地下１階	03-3245-2800	www.drwatanabe.com	○	○	呼吸器内科／循環器内科／消化器内科	渡邊　龍彦	東京逓信病院（東京都千代田区富士見2-14-23）
72	イワサキクリニック東京	101-0044	東京都千代田区鍛冶町二丁目９番５号<br>東園ビル３階	03-3256-0055	http://www.soujikai.jp/clinic/iwasaki-tokyo.html		○	アレルギー科	岩崎　純夫	

電話や情報通信機器を用いて診療を実施する医療機関の一覧（東京都）

	基本情報					事務連絡に基づく対応について				
	施設名	郵便番号	住所（都道府県から記載）	電話番号	ウェブサイトURL	初診の電話等を用いた診療の実施の有無	再診の電話等を用いた診療の実施の有無	対応診療科	担当医師名	対面診療が必要と判断した場合に連携する医療機関名（複数ある場合は複数、住所も併せて記載）
73	ＨＤＣアトラスクリニック	102-0082	東京都千代田区一番町５番地３<br>アトラスビル１階	03-3234-6060		○	○	糖尿病内科／内科	鈴木　吉彦	慶應義塾大学病院
74	エムアンドエムスキンケアクリニック	101-0051	東京都千代田区神田神保町二丁目９番地<br>神田神保町メディカルモール３階	03-3556-8866			○	皮膚科／形成外科	鈴木　みね子／今野　みどり／萩原　ゆかり	
75	エムズクリニック九段下	101-0051	東京都千代田区神田神保町三丁目５番地<br>ニュー徳栄ビル８階	03-3239-5857	http://www.ms-clinic.com		○	精神科／心療内科	藤田　充明	
76	大島眼科医院	102-0074	東京都千代田区九段南四丁目７番１３号<br>市ヶ谷安田ビル２階	03-3261-4132	http://www.oshima-eye.com		○	眼科	大島　剛	
77	大手町フィナンシャルシティ西山クリニック	100-0004	東京都千代田区大手町大手町一丁目９番４号<br>大手町フィナンシャルシティノースタワー４階	03-3270-2986	kouko-clinic-nishiyama		○	内科／循環器科	西山　信一郎	虎の門病院（東京都港区虎ノ門2-2-2）<br>聖路加国際病院（東京都中央区明石町9-1）<br>東京逓信病院（東京都千代田区富士見2-14-23）
78	大手町プレイス内科	100-0004	東京都千代田区大手町二丁目３番１号<br>大手町プレイスウエストタワー地下１階　Ｂ１０９区画、Ｂ１１０区画	03-6262-5622	http://otemachi-place-naika.clinic/		○	内科／糖尿病	田嶼　尚子	
79	大森胃腸科	102-0092	東京都千代田区隼町2-15	03-3234-6226	oomori-i.jp		○	内科	大森　格	東京逓信病院（東京都千代田区富士見2-14-23）
80	お茶の水血管外科クリニック	101-0062	東京都千代田区神田駿河台二丁目１番地<br>ヒルクレスト御茶ノ水５階	03-5281-4103	https://www.kekkangeka.com		○	血管外科	廣川　雅之／栗原　伸久／伴祐子	三楽病院（東京都千代田区神田駿河台2-5）
81	貝坂クリニック	102-0093	東京都千代田区平河町一丁目４番１２号<br>平河町センタービル１０階３号	03-5213-6710		○	○	内科	髙野　学美／髙野　義人	東京逓信病院（東京都千代田区富士見2-14-23）

電話や情報通信機器を用いて診療を実施する医療機関の一覧（東京都）

	基本情報					事務連絡に基づく対応について				
	施設名	郵便番号	住所（都道府県から記載）	電話番号	ウェブサイトURL	初診の電話等を用いた診療の実施の有無	再診の電話等を用いた診療の実施の有無	対応診療科	担当医師名	対面診療が必要と判断した場合に連携する医療機関名（複数ある場合は複数、住所も併せて記載）
82	加賀耳鼻咽喉科クリニック	101-0051	東京都千代田区神田神保町一丁目３１番地１ 加賀耳鼻科ビル１階２階	03-3295-3387	http://www.kaga.min.gr.jp		○	耳鼻咽喉科	相田　瑞恵	
83	霞が関アーバンクリニック	100-0013	東京都千代田区霞が関一丁目４番１号 日土地ビル１階	03-5157-3911	https://www.urban-clinic.jp/		○	内科／リウマチ科／膠原病内科／整形外科／アレルギー科／精神科／脳神経内科／皮膚科／呼吸器内科	西岡　久寿樹／中島　正男／臼井　千恵／西岡健弥／高柳　広／森本　幾夫／友利　新／石原陽子／津田　淳子／平井　利明	
84	株式会社日立製作所本社秋葉原ダイビル診療所	101-8608	東京都千代田区外神田一丁目１８番１３号秋葉原ダイビル１７F	03-4564-3423		○	○	内科	秋山義之、大友夏子	
85	株式会社日立製作所本社丸の内センタービル診療所	100-8220	東京都千代田区丸の内一丁目６番１号丸の内センタービルディング１２F	03-4235-4244		○	○	内科	秋山義之、大友夏子	
86	河内クリニック	101-0063	東京都千代田区神田淡路町一丁目９番地１ ニューお茶の水ビル３０１	03-3252-8225			○	精神科／神経科	河内　清	左記施設
87	神田医院	101-0044	東京都千代田区鍛冶町1-8-6 神田ＫＳビル２Ｆ	03-3252-8896	www.kanda-iin.com	○	○	内科／循環器内科／精神科／心療内科	里井　重仁（初診対応・再診内科）／里井孝光（再診のみ内科・循内）／石井　元康（再診のみ　精・心）	三井記念病院（東京都千代田区神田和泉町1）
88	神田ウィメンズクリニック	101-0044	東京都千代田区鍛冶町二丁目８番６号 メディカルプライム神田６階	03-6206-0065	https://kandawomens.com	○	○	生殖医療婦人科／ 婦人科	清水　真弓	
89	神田駅東口クリニック	101-0044	東京都千代田区鍛冶町二丁目８番６号 メディカルプライム神田２階	03-6271-7916	https://kanda.clinic	○	○	内科	馬渕　浩輔／吉田　任子	
90	神田北口診療所	101-0047	東京都千代田区内神田三丁目２０番３号 小鍛冶ビル４階	050-5840-6565	https://kandakita.clinic/	○	○	内科	内山　武史／岡田　恭江／福田貴規／安井　梢子	

電話や情報通信機器を用いて診療を実施する医療機関の一覧（東京都）

	基本情報					事務連絡に基づく対応について				
	施設名	郵便番号	住所（都道府県から記載）	電話番号	ウェブサイトURL	初診の電話等を用いた診療の実施の有無	再診の電話等を用いた診療の実施の有無	対応診療科	担当医師名	対面診療が必要と判断した場合に連携する医療機関名（複数ある場合は複数、住所も併せて記載）
91	神田すこやかクリニック	101-0041	東京都千代田区神田須田町1-30-1-104	03-6206-0153	http://www.kanda-sukoyaka.com	○	○	内科<br>皮膚科・アレルギー科	橋本　亮	国立国際医療研究センター病院（新宿区戸山1-21-1）
92	神田ホリスティックひふ科	101-0054	東京都千代田区神田錦町二丁目１番地５<br>マストライフ神田錦町１階	03-3294-1112		○	○	皮膚科	若松　順子	東京医科大学病院（東京都新宿区西新宿7-6-1）
93	紀尾井町内科	102-0094	東京都千代田区紀尾井町３番１号<br>ＫＫＤビル１階	03-6265-6500	https://kioichonaika.jp	○	○	内科／循環器科	市村　有紀子	
94	九段こころのクリニック	102-0073	東京都千代田区九段北一丁目１番５号<br>第二中央ビル３階	03-6256-8397	kudan-kokoro.clinic/	○	○	心療内科／精神科	後藤　牧子	順天堂大学医学部附属病院（東京都文京区本郷2-1-1）
95	九段下さくら形成外科・皮膚科	102-0073	東京都千代田区九段北一丁目３番１号<br>日宝九段下ビル５階	03-3222-8341	https://sakura-skin.jp	○	○	皮膚科／形成外科／小児皮膚科／美容皮膚科	木下　佳保里	九段坂病院（東京都千代田区九段南1-6-12）<br>東京逓信病院（東京都千代田区富士見2-14-23）
96	九段下駅前ココクリニック	102-0073	東京都千代田区九段北一丁目２番１号<br>九段中央ビル３階	03-5212-5551	http://kudanshita.clinic	○	○	内科／循環器内科／内分泌・代謝内科	石井　聡	
97	九段下駅前まめクリニック	102-0073	東京都千代田区九段北一丁目３番２号<br>大橋ビル３階	03-6256-9273	https://mame-clinic.jp	○	○	内科	石川　雅俊	三井記念病院（東京都千代田区神田和泉町1）<br>東京逓信病院（東京都千代田区富士見2-14-23）<br>日本大学病院（東京都千代田区神田駿河台1-6）<br>順天堂大学医学部付属順天医院<br>三楽病院（東京都千代田区神田駿河台2-5）<br>JCHO東京新宿メディカルセンター<br>東京大学医学部付属病院<br>虎の門病院
98	クリニックフォア飯田橋	102-0071	東京都千代田区富士見二丁目７番１号<br>飯田橋プラーノ１階	03-4579-9486	https://www.clinicfor.life/iidabashi/	○	○	内科／皮膚科／アレルギー科	渡辺　絵梨沙	済生会中央病院（東京都港区三田1-4-17）<br>東京逓信病院（東京都千代田区富士見2-14-23）<br>虎の門病院（東京都港区虎ノ門2-2-2）<br>東京医科大学病院（東京都新宿区西新宿7-6-1）
99	クリニックフォア有楽町	100-0006	東京都千代田区有楽町二丁目７番１号<br>有楽町駅前ビルディングイトシアプラザ地下１階	03-6269-9061	www.clinicfor.life/yurakucho/	○	○	内科／皮膚科／アレルギー科	永田　明久	済生会中央病院（東京都港区三田1-4-17）<br>虎の門病院（東京都港区虎ノ門2-2-2）<br>東京慈恵会医科大学附属病院（東京都港区西新橋3-19-18）

電話や情報通信機器を用いて診療を実施する医療機関の一覧（東京都）

	基本情報					事務連絡に基づく対応について				
	施設名	郵便番号	住所（都道府県から記載）	電話番号	ウェブサイトURL	初診の電話等を用いた診療の実施の有無	再診の電話等を用いた診療の実施の有無	対応診療科	担当医師名	対面診療が必要と判断した場合に連携する医療機関名（複数ある場合は複数、住所も併せて記載）
100	グローバルヘルスケアクリニック	102-0083	東京都千代田区麹町麹町三丁目１２番地 麹町ＭＳビル１階	03-3222-8832	https://ghc.tokyo	○	○	内科／小児科／アレルギー科	水野　泰孝	
101	ケイアイ秋葉原クリニック	101-0023	東京都千代田区神田松永町１１AT第一ビル	03-3255-8755	http://kiclinic.jp/kiac/	○	○	内科	石原　雅巳	三井記念病院（東京都千代田区神田和泉町1番地）
102	公益財団法人佐々木研究所附属 杏雲堂病院	101-0062	東京都千代田区神田駿河台1-8	03-3292-2051	https://www.kyoundo-hospital.jp/		○	内科/外科/婦人科/整形外科/乳腺外科/循環器科/消肝内科/腫瘍内科/呼吸器科/皮膚科/泌尿器科/放射線科	各医師	日本大学病院（東京都千代田区神田駿河台1-6）<br>日本大学医学部附属板橋病院（東京都板橋区大谷口上町30-1）<br>東京医科歯科大学医学部附属病院(東京都文京区湯島1-5-45)<br>東京大学医学部附属病院(東京都文京区本郷7-3-1)<br>東京警察病院(東京都中野区中野4-22-1)
103	公益財団法人神経研究所附属睡眠呼吸障害クリニック	101-0061	東京都千代田区三崎町二丁目１８番１１号 堀内三崎町ビル２階	03-3556-9181	http://www.suiminclinic.com		○	呼吸器内科／精神科	福原　俊明／大賀　栄次郎／伊藤　永喜／松本容子／結城　将明／中島　大輝／梶村　尚史／福田　健介／河野　史歩／由井	
104	麹町眼科	102-0094	東京都千代田区紀尾井町３番１９号 紀尾井町コートビル２０１	03-3222-0041			○	眼科／内科	新本　和英	国立国際医療研究センター（東京都新宿区戸山1-21-1）<br>JCHO東京新宿メディカルセンター（東京都新宿区津久戸町5-1）
105	麹町消化器・内視鏡クリニック	102-0083	東京都千代田区麹町四丁目６番地８ ダイニチ麹町ビル１階	03-5215-1114	https://kojimachi-gc.com	○	○	消化器内科／内視鏡内科／肛門外科／内科	内田　寛	東京逓信病院（東京都千代田区富士見2-14-23）<br>九段坂病院（東京都千代田区九段南1-6-12）
106	麹町皮ふ科・形成外科クリニック	102-0093	東京都千代田区平河町1-4-5平和第一ビル地下１階	03-6261-2458	https://kojihifu.com/	○	○	皮膚科	苅部　淳<br>坂本　淳	
107	ココロモクリニック神田	101-0044	東京都千代田区鍛冶町二丁目８番６号 メディカルプライム神田５階	03-3254-5560	kokoromo.jp	○	○	精神科／心療内科	山下　佑介	
108	国家公務員共済組合連合会　九段坂病院	102-0074	東京都千代田区九段南1-6-12	03-3262-9191	http://www.kudanzaka.com/		○	内科　外科　整形外科　皮膚科　泌尿器科　婦人科　耳鼻咽喉科　眼科	当日担当医師	自院にて対応

電話や情報通信機器を用いて診療を実施する医療機関の一覧（東京都）

	基本情報					事務連絡に基づく対応について				
	施設名	郵便番号	住所（都道府県から記載）	電話番号	ウェブサイトURL	初診の電話等を用いた診療の実施の有無	再診の電話等を用いた診療の実施の有無	対応診療科	担当医師名	対面診療が必要と判断した場合に連携する医療機関名（複数ある場合は複数、住所も併せて記載）
109	こばなわ神田整形外科	101-0044	東京都千代田区鍛冶町二丁目２番９号<br>第二登栄ビル６階	03-5297-2462	kandaseikei.com		○		小塙　幸司	
110	さくらの木クリニック秋葉原	101-0033	東京都千代田区神田岩本町1番地<br>清水ビル２階	03-3255-3960	http://www.sakuranoki-akihabara.com/		○	精神科	倉持　穣	東京医科歯科大学医学部附属病院（東京都文京区湯島1-5-45）<br>三井記念病院（東京都千代田区神田和泉町1）
111	佐々木内科クリニック	102-0083	東京都千代田区麹町四丁目４番地６<br>麹町四丁目小倉ビル２階	03-3264-3635	http://www.sasaki-m-clinic.com	○	○	内科／小児科	佐々木　俊治	東京逓信病院（東京都千代田区富士見2-14-23）<br>順天堂医院（東京都文京区本郷3-1-3）<br>日本大学病院（東京都千代田区神田駿河台1-6）
112	山王メディカルクリニック	100-0014	東京都千代田区永田町二丁目１２番８号<br>永田町ＳＲビル１階	03-3539-3012	www.s-med.jp	○	○	皮膚科	宮地　百子	北里研究所病院（東京都港区白金5-9-1）<br>済生会中央病院（東京都港区三田1-4-17）<br>慶應義塾大学病院（東京都新宿区信濃町35）
113	島田内科	101-0047	東京都千代田区内神田二丁目１１番１号<br>島田ビル９階	03-3252-0121		○	○	内科	島田　英世	
114	社会福祉法人　三井記念病院	101-8643	東京都千代田区神田和泉町１番地	03-3862-9111	https://www.mitsuihosp.or.jp		○	全診療科（内．呼内．消内．循内．小．神内．精．外．消外．呼外．整．脳外．産婦．眼．耳咽．泌．皮．麻．歯．歯外．	全医師	
115	常喜医院	102-0085	東京都千代田区六番町７番地１<br>番町グロリアビル１階	03-5226-7557	joki-clinic.jp	○	○	内科／皮膚科	常喜　眞理	
116	神保町駅前皮膚科	101-0051	東京都千代田区神田神保町1-7-12<br>巌松堂ビル６階	03-5577-3877	https://jimbocho-hifu.com		○	皮膚科／アレルギー科	中村　明博／後藤　あかね	東京逓信病院（東京都千代田区富士見2-14-23）
117	神保町整形外科	101-0051	東京都千代田区神田神保町一丁目２９番地<br>すずらんビル２階３階４階	03-5577-7997	https://www.jimbocho-seikei.com	○	○	整形外科	板倉　剛	慶應義塾大学病院（東京都新宿区信濃町35）<br>聖路加国際病院（東京都中央区明石町9-1）<br>日本大学病院（東京都千代田区神田駿河台1-6）

電話や情報通信機器を用いて診療を実施する医療機関の一覧（東京都）

	基本情報					事務連絡に基づく対応について				
	施設名	郵便番号	住所（都道府県から記載）	電話番号	ウェブサイトURL	初診の電話等を用いた診療の実施の有無	再診の電話等を用いた診療の実施の有無	対応診療科	担当医師名	対面診療が必要と判断した場合に連携する医療機関名（複数ある場合は複数、住所も併せて記載）
118	神保町たねもと眼科	101-0054	東京都千代田区神田錦町三丁目１８番地１０ 浜田ビル１階	03-5577-2913			○	眼科	種元　桂子	左記施設
119	水道橋メディカルクリニック	101-0065	東京都千代田区西神田一丁目４番１１号 サンポウ水道橋ビル301号	03-3518-6127			○	内科	砂山　聡	順天堂医院（東京都文京区本郷3-1-3）
120	駿河台こころのクリニック	101-0062	東京都千代田区神田駿河台三丁目２番地６ Ａ・Ｐｌａｚａ御茶の水ビル７階	03-3257-5678	http://surugadai-kokoro.com		○	心療内科／精神科	宮武　良輔	
121	聖堂前クリニック	101-0052	東京都千代田区神田小川町3-1-10 メディカビル６階	050-3734-5574	https://selidomae.com		○	精神科	木村　智城	
122	聖路加国際病院附属クリニック聖路加メディローカス	100-0004	東京都千代田区大手町1-9-7 大手町フィナンシャルシティサウスタワー２階	03-3527-9520	http://medilocus.luke.ac.jp		○	内科／呼吸器内科／循環器内科／消化器内科／婦人科	本多　一文／藤井　さと子／田草川　正弘／酒見　智子／岡田瑠理子	
123	瀬木診療所	101-0032	東京都千代田区岩本町一丁目７番１号 瀬木ビル３階	03-3866-7313		○	○	内科／皮膚科	瀬木　邦久	
124	雪月花メディカルクリニック秋葉原診療所	101-0021	東京都千代田区外神田6-14-2 サカイ末広ビル７階Ｂ室	03-5812-5301	https://snow-moon-flower.jp		○	内科／皮膚科／アレルギー科	大道　雅文	
125	雪月花メディカルクリニック秋葉原中央通診療所	101-0041	東京都千代田区神田須田町１－７－８　秋葉原シグマビル１階	03-3525-8025	https://akbcore.jp	○	○	内科／皮膚科／アレルギー科	齋藤　哲史	
126	Sowaka women's health clinic	102-0074	東京都千代田区九段南3-7-8	03-6910-0753	https://sowaka-cl.com	○	○	婦人科	竹元　葉	

電話や情報通信機器を用いて診療を実施する医療機関の一覧（東京都）

	基本情報					事務連絡に基づく対応について				
	施設名	郵便番号	住所（都道府県から記載）	電話番号	ウェブサイトURL	初診の電話等を用いた診療の実施の有無	再診の電話等を用いた診療の実施の有無	対応診療科	担当医師名	対面診療が必要と判断した場合に連携する医療機関名（複数ある場合は複数、住所も併せて記載）
127	竹橋クリニック	101-0054	東京都千代田区神田錦町3-17<br>北の丸ビル１階	03-3295-5525	http://takebashiclinic.jimdo.com		○	内科	徳永　昌子	東京逓信病院（東京都千代田区富士見2-14-23）<br>九段坂病院（東京都千代田区九段南1-6-12）<br>聖路加国際病院<br>東京慈恵会医科大学附属病院
128	帝国クリニック	100-0011	東京都千代田区内幸町一丁目１番１号<br>帝国ホテル本館4階	03-3503-8681		○	○	内科	岩本　耕太郎	東京逓信病院（東京都千代田区富士見2-14-23）<br>虎の門病院（東京都港区虎ノ門2-2-2）<br>聖路加国際病院<br>慈恵医大病院<br>日赤医療センター
129	東京キャンサークリニック	102-0072	東京都千代田区飯田橋一丁目３番２号<br>曙杉館ビル９階	03-6380-8031	https://tokyocancerclinic.jp/	○	○	内科	阿部　博幸／笹田　亜麻子	一般財団法人全日本労働福祉協会　九段クリニック（東京都千代田区九段北1-9-5　朝日九段マンション１階１号室）
130	東京クリニック	100-0004	東京都千代田区大手町二丁目２番１号<br>新大手町ビル１階・地下１階・地下２階	03-3516-7151			○	内科／ペインクリニック内科／小児科／神経内科／呼吸器内科／消化器内科／形成外科／美容外科／皮膚科／美容皮膚科／外		
131	東京シーサイドクリニック	102-0093	東京都千代田区平河町2-14-7 YUKEN平河町ビル3F	03-6272-6616	http://www.ts-clinic.jp/	○	○	内科/循環器内科	中川敬一	東京逓信病院（千代田区富士見2-14-23）<br>東京高輪病院（港区高輪3-10-11）
132	東京歯科大学　水道橋病院	101-0061	東京都千代田区三崎町２－９－１８	03-5275-1856	http://www.tdc.ac.jp/sh/tabid/233/Default.aspx	○<br>（ただし、内科のみ）	○	眼科／内科	【眼科】<br>太田　友香／西島　有衣<br>【内科】<br>山岸　由幸	左記施設<br>受診不可であれば、近医に紹介状送付
133	東京都信用金庫健康保険組合診療所	101-8312	東京都千代田区神田駿河台二丁目９番地２<br>信用金庫健保会館２～４階	03-5280-0561	https://www.shinkinkenpo.or.jp		○	内科	藤原　啓二	
134	東京ビジネスクリニック　グランスタ丸の内	100-0005	東京都千代田区丸の内一丁目９番１号<br>ＪＲ東日本東京駅構内地下１階	03-6259-1605		○	○	内科／外科／皮膚科／小児科	内藤　祥	聖路加国際病院（東京都中央区明石町9-1）<br>虎の門病院（東京都港区虎ノ門2-2-2）<br>慶應義塾大学病院（東京都新宿区信濃町35）<br>三井記念病院（東京都千代田区神田和泉町1）<br>東京医科歯科大学医学部附属病院（東京都文京区湯島1-5-45）
135	東京ビジネスクリニック　八重洲北口	100-0005	東京都千代田区丸の内一丁目８番２号<br>鉄鋼ビルディング地下１階	03-6268-0079	businessclinic.tokyo	○	○	内科／皮膚科／小児科／外科	小野　顕人／内藤　祥	聖路加国際病院（東京都中央区明石町9-1）<br>慶應義塾大学病院（東京都新宿区信濃町35）<br>東京医科歯科大学医学部附属病院（東京都文京区湯島1-5-45）<br>虎の門病院（東京都港区虎ノ門2-2-2）<br>三井記念病院（東京都千代田区神田和泉町1）

電話や情報通信機器を用いて診療を実施する医療機関の一覧（東京都）

	基本情報					事務連絡に基づく対応について				
	施設名	郵便番号	住所（都道府県から記載）	電話番号	ウェブサイトURL	初診の電話等を用いた診療の実施の有無	再診の電話等を用いた診療の実施の有無	対応診療科	担当医師名	対面診療が必要と判断した場合に連携する医療機関名（複数ある場合は複数、住所も併せて記載）
136	東京ビジネスパーソンズクリニック	101-0044	東京都千代田区鍛冶町一丁目８番３号 神田９１ビル１階	03-6206-4484	https://tokyo-bp-clinic.com		○	内科／皮膚科	伊藤　諭	東京逓信病院（東京都千代田区富士見2-14-23）
137	中田クリニック	100-0011	東京都千代田区内幸町二丁目２番１号 日本プレスセンタービルＢ１	03-5511-7770			○	呼吸器科	中田　紘一郎	東京逓信病院（東京都千代田区富士見2-14-23）<br>虎の門病院<br>聖路加国際病院
138	日本医科大学呼吸ケアクリニック	102-0074	東京都千代田区九段南四丁目７番１５号 ＪＰＲ市ヶ谷ビル８階	03-5276-2325			○	呼吸器内科	日野　光紀／谷内　七三子／宮永　晃彦／吉川　明子	日本医科大学付属病院
139	日本歯科大学附属病院	102-8158	東京都千代田区富士見２－３－１６	03-3261-5511	http://dent-hosp.ndu.ac.jp/nduhosp/		○	内科<br>外科	全医師	
140	二宮内科クリニック	101-0044	東京都千代田区鍛冶町一丁目９番１号 第二大久保ビル３階	03-3254-5007	http://ninomiya-cl.jp	○	○	内科／リウマチ科／糖尿病内科／アレルギー科	髙崎　千穂／山中　健次郎	
141	原プレスセンタークリニック	100-0011	東京都千代田区内幸町二丁目２番１号 日本プレスセンタービルＢ１	03-3595-2961	https://hara-pcc.net		○	内科／腎臓内科	原　茂子	虎の門病院
142	はりまメンタルクリニック	101-0052	東京都千代田区神田小川町三丁目２４番地１ カスタリアお茶の水１０２号室	03-5281-4800	www.harimamental.com		○	精神科	針間　克己	
143	パレスクリニック	100-0003	東京都千代田区一ツ橋1-1-1 パレスサイドビル１階	03-3213-2855	http://palaceclinic99.wixsite.com/gairai	○	○	耳鼻咽喉科／皮膚科／眼科	春山　喜一	順天堂大学医学部付属順天堂医院
144	半蔵門のびすここどもクリニック	102-0082	東京都千代田区一番町４番地１６ プルミエール一番町　１階	03-6261-7540	https://nobisuko.jp	○	○	小児科／児童精神科	河嶌　美穂／河嶌　譲	

電話や情報通信機器を用いて診療を実施する医療機関の一覧（東京都）

	基本情報					事務連絡に基づく対応について				
	施設名	郵便番号	住所（都道府県から記載）	電話番号	ウェブサイトURL	初診の電話等を用いた診療の実施の有無	再診の電話等を用いた診療の実施の有無	対応診療科	担当医師名	対面診療が必要と判断した場合に連携する医療機関名（複数ある場合は複数、住所も併せて記載）
145	日比谷ガーデンクリニック	100-0006	東京都千代田区有楽町１－５－１ 日比谷マリンビル地下２階	03-6206-1121	https://www.hibiya-garden-clinic.com	○	○	心療内科／精神科	髙橋　栄	左記施設
146	広川眼科	101-0044	東京都千代田区鍛冶町2-2-9 第二登栄ビル４階	03-3256-3839			○	眼科	広川　正泰	
147	ホテルニューオータニ診療所	102-0094	東京都千代田区紀尾井町４番１号 ホテルニューオータニ本館地下１階	03-3221-4154			○	内科	山本　純子	東京逓信病院（東京都千代田区富士見2-14-23）<br>東京慈恵会医科大学附属病院（東京都港区西新橋3-19-18）<br>九段坂病院（東京都千代田区九段南1-6-12）<br>心臓血管研究所付属病院（東京都港区西麻布3-2-19）
148	まなメンタルクリニック新御茶ノ水	101-0063	東京都千代田区神田淡路町1-11-8 淡路町ＵＫビル３階	03-3526-2613	https://manaclinic.jp		○	心療内科／精神科	市川　弓紀子	
149	丸ノ内なかごみクリニック	100-0005	東京都千代田区丸の内二丁目２番１号 岸本ビル２階	03-6551-2011	marunouchi-nakagomi.com		○	内科／脳神経外科	中込　忠好	東京逓信病院（東京都千代田区富士見2-14-23）
150	丸の内の森レディースクリニック	100-8509	東京都千代田区丸の内一丁目５番１号 新丸の内ビルディング９階	03-6206-3900			○	産科／婦人科	宋　美玄	
151	三木内科クリニック	101-0047	東京都千代田区内神田3-14-8　ニシザワビル１Ｆ	03-5298-4120			○	内科	三木　治	
152	明神下青木皮膚科	101-0021	東京都千代田区外神田2-3-6 ＫＩＴＡＨＡＲＡビル２階	03-3251-3666			○	皮膚科	青木　見佳子	
153	森山眼科医院	101-0021	東京都千代田区外神田三丁目１３番１２号	03-3251-3938			○	眼科	森山　知英郎	

電話や情報通信機器を用いて診療を実施する医療機関の一覧（東京都）

	基本情報					事務連絡に基づく対応について				
	施設名	郵便番号	住所（都道府県から記載）	電話番号	ウェブサイトURL	初診の電話等を用いた診療の実施の有無	再診の電話等を用いた診療の実施の有無	対応診療科	担当医師名	対面診療が必要と判断した場合に連携する医療機関名（複数ある場合は複数、住所も併せて記載）
154	柳下医院	102-0093	東京都千代田区平河町一丁目７番１４号<br>１階	03-3261-1261			○	内科／循環器内科	柳下　芳樹	左記施設
155	やじまクリニック	102-0072	東京都千代田区飯田橋一丁目８番９号<br>ニューシティーハイツ飯田橋２０１	03-3261-7853	http://www.yajima-cl.net	○	○	内科	矢島　俊巳	患者に合わせて紹介
156	山本亜希メンタルクリニック	102-0073	東京都千代田区九段北一丁目３番２号<br>大橋ビル６階	03-3230-0002	http://www.yamamotoaki.jp		○	精神科／心療内科	山本　亜希	東京逓信病院（東京都千代田区富士見2-14-23）<br>だいだいクリニック（東京都千代田区九段南1-6-17　千代田会館１階区画Ｂ）
157	有楽町睡眠・呼吸器内科クリニック	100-0006	東京都千代田区有楽町1－7－1<br>有楽町電気ビル　北館5階	03-5221-8088	www.yurakucho-sleep.jp	○	○	内科／呼吸器内科	永岡賢一／赤星俊樹／遠藤大介／佐藤誠／横江琢也／関山忠孝／楠裕司／渡部良雄／岡本真一／佐藤春奈／河原朋子	東京逓信病院（東京都千代田区富士見2－14－23）
158	有楽町ビル駅前眼科	100-0006	東京都千代田区有楽町一丁目１０番１号<br>有楽町ビルヂング４Ｆ	03-3213-2028	http://www.yurakucho-ganka.jp		○	眼科	深澤　あゆみ	
159	有楽町ビル婦人科クリニック	100-0006	東京都千代田区有楽町一丁目１０番１号<br>有楽町ビル４階４１４区	03-3214-2568			○	産婦人科	岡宮　育世	
160	洋洋クリニック	102-0071	東京都千代田区富士見2-10-2<br>飯田橋グラン・ブルーム３階	03-6272-3318	http://youyouclinic.g1.xrea.com	○	○	内科／小児科	白川　洋一	
161	リーレクリニック大手町	100-0004	東京都千代田区大手町一丁目３番２号<br>大手町カンファレンスセンター　ショップ＆レストラン　地下１階ＳＢ１２４区	03-5224-6672	lireclinic.com	○	○	内科	片山　泰輔	慶應義塾大学病院<br>東京医科歯科大学医学部付属病院
162	六番町耳鼻咽喉科	102-0085	東京都千代田区六番町３番地１<br>協和ビル３階	03-3222-4133			○	耳鼻咽喉科	湯浅　貴文	東京逓信病院（東京都千代田区富士見2-14-23）

電話や情報通信機器を用いて診療を実施する医療機関の一覧（東京都）

	基本情報					事務連絡に基づく対応について				
	施設名	郵便番号	住所（都道府県から記載）	電話番号	ウェブサイトURL	初診の電話等を用いた診療の実施の有無	再診の電話等を用いた診療の実施の有無	対応診療科	担当医師名	対面診療が必要と判断した場合に連携する医療機関名（複数ある場合は複数、住所も併せて記載）
163	医療法人社団　久響会<br>あいクリニック神田	101-0047	東京都千代田区内神田3-14-8<br>ニシザワビル5階	03-3253-2100	http://www.i-clinic.or.jp		○	精神科<br>心療内科	鬼頭諭<br>西松能子<br>野村俊明<br>齊藤卓弥<br>太田直子<br>角田ひとみ<br>金吉晴<br>岩崎康孝	
164	山王クリニック	100-6125	東京都千代田区永田町2-11-1<br>山王パークタワー25階	03-3580-5001	https://www.sannoclinic.jp/	○	○	内科、精神科、皮膚科、循環器科	鈴木　努	
165	富士見スキンクリニック飯田橋	102-0071	東京都千代田区富士見2-11-10-B1F	03-6261-7366	https://www.fujimiskin.com/	○	○	皮膚科<br>美容皮膚科<br>アレルギー科	小渕　英里<br>吉原　有希<br>仲　沙耶香	当院
166	お茶の水耳鼻咽喉科・アレルギー科	101-0062	東京都千代田区神田駿河台2-10-6VORT御茶ノ水2階	03-5843-9226	https://ochanomizu-ent.com/	○	○	耳鼻咽喉科<br>アレルギー科	吉田　千栄子	
167	出版健康保険組合診療所	101-0062	東京都千代田区神田駿河台1-7	03-3292-5092	https://www.phia.or.jp		○	内科	松岡　美佳	
168	有明こどもクリニック勝どき院	104-0054	東京都中央区勝どき3-5-5AIP勝どき駅前ビル3階	03-3533-7281	https://child-clinic.or.jp	○	○	小児科　内科	中本　祐樹	
169	（医）慶友 慶友銀座ｸﾘﾆｯｸ	104-0045	東京都中央区築地1-13-11　高橋ビル2F	03-3542-3387	https://www.ginzaclinic.com/	○	○	耳鼻咽喉科内科	大場俊彦 全勤務医	東京都済生会中央病院（東京都港区三田1-4-17）
170	医）八丁堀医院	104-0032	東京都中央区八丁堀２－２０－８　八丁堀綜通ビル９Ｆ	03-3551-8860	https://www.hatchobori-iin.com	○	○	内科	里井　豊	聖路加国際病院（東京都中央区）
171	医療法人オーク会<br>オーク銀座レディースクリニック	104-0061	東京都中央区銀座2-6-12 Okura House7階	0120-009-345	https://www.oakclinic-group.com/	○	○	産科・婦人科	全医師	

電話や情報通信機器を用いて診療を実施する医療機関の一覧（東京都）

	基本情報					事務連絡に基づく対応について				
	施設名	郵便番号	住所（都道府県から記載）	電話番号	ウェブサイトURL	初診の電話等を用いた診療の実施の有無	再診の電話等を用いた診療の実施の有無	対応診療科	担当医師名	対面診療が必要と判断した場合に連携する医療機関名（複数ある場合は複数、住所も併せて記載）
172	医療法人社団医成会<br>銀座さわだ内科クリニック	104-0061	東京都中央区銀座5-9-15銀座清月堂ビル4階	03-3572-4898	http://iseikai-med.or.jp/	○	○	内科<br>消化器内科	澤田　秀雄<br>澤田　貴志	
173	医療法人社団健育会<br>石川島記念病院	104-0051	東京都中央区佃二丁目5番2号	03-3532-3201	https://ishikawajima.gr.jp/		○	内科<br>整形外科	全医師	
174	医療法人社団頌栄会　上田診療所	103-0027	東京都中央区日本橋2-1-10柳屋ビル地下1回	03-3271-2775	https://www.ueda.com		○	内科<br>小児科	上田　晃<br>志村　右子	
175	医療法人社団スリープクリニック<br>スリープクリニック銀座	104-0061	東京都中央区銀座3-8-10銀座朝日ビル8階	03-554-7700	https://www.sleepmedicine-tokyo.com/		○	精神科<br>心療内科	当日の担当医	
176	医療法人社団　玉寄クリニック	103-0013	東京都中央区日本橋人形町1-12-11リガーレ日本橋人形町105号	03-3661-5555	https://tamayose-cl.jp	○	○	内科、糖尿病内科、小児科	玉寄兼治<br>玉寄皓大	
177	医療法人社団千秋双葉会<br>日本橋茅場町耳鼻咽喉科	103-0025	東京都中央区日本橋茅場町2-8-10碧山ビル3階	03-3669-2085	http://www.nihonbashi-ent.com/sp/		○	耳鼻咽喉科	植草康浩	日本大学病院（〒101-8309 東京都千代田区神田駿河台1-6）三井記念病院（〒101-8643 東京都千代田区神田和泉町1番地）
178	医療法人社団進興会<br>東京ダイヤビルクリニック	104-0033	東京都中央区新川1-28-25<br>東京ダイヤビルディング3号館1階	03-3551-7737	https://www.daiya-clinic.jp/	○	○	内科<br>消化器内科<br>呼吸器科<br>循環器科<br>禁煙治療<br>婦人科<br>男性専門治療	川村　洋<br>細井広子、野田泰弘、山田康嗣、川村　洋<br>渡邉賀代<br>陣内恵美子<br>川村　洋<br>田續綾野	日本大学病院(〒101-8309 東京都千代田区神田駿河台1-6)<br>聖路加国際病院(101-8560東京都中央区明石町9-1)<br>(公財)心臓血管研究所付属病院(106-0031東京都港区西麻布3-2-19)<br>浜松町ハマサイトクリニック(〒105-0022 東京都港区海岸1-2-20 汐留ビルディング2F)<br>国立がん研究センター中央病院(104-0045東京都中央区
179	医療法人社団桃仁日本橋レディースクリニック	103-0022	東京都中央区日本橋室町1-5-2　8階	03-3516-3150	http://www.nl-clinic.jp		○	胃腸科<br>肛門科	野澤真木子	
180	医療法人社団　平郁会<br>日本橋かきがら町<br>クリニック	103-0014	東京都中央区日本橋蛎殻町1丁目10-4 宮田ビル2階	03-5651-1361	http://www.heiikukai.com/clinic/kakigara/	○	○	内科<br>小児科	鴈野　上田<br>深井	

電話や情報通信機器を用いて診療を実施する医療機関の一覧（東京都）

	基本情報					事務連絡に基づく対応について				
	施設名	郵便番号	住所（都道府県から記載）	電話番号	ウェブサイトURL	初診の電話等を用いた診療の実施の有無	再診の電話等を用いた診療の実施の有無	対応診療科	担当医師名	対面診療が必要と判断した場合に連携する医療機関名（複数ある場合は複数、住所も併せて記載）
181	医療法人社団墨水会浜町センタービルクリニック	103-0007	東京都中央区日本橋浜町2-31-1	03-3664-6858	http://hamacho-cc.or.jp/		○	内科<br>外科<br>消化器科<br>循環器科<br>呼吸器科<br>糖尿病科<br>脳神経科	窪田徳光<br>木村暢孝	
182	医療法人社団　わたなべこどもクリニック	104-0052	東京都中央区月島1－8－1アイ・マークタワー202	03-5548-2511	http://www.watanabe-kodomo.jp		○	小児科	渡邉浩志	
183	医）リゾーム慶和会ザ東京タワーズクリニック	104-0054	東京都中央区勝どき6-3-2-102	03-3534-9002	http://www.thetowers.jp	○	○	内科	矢野芳和<br>矢野宏行	
184	ＥＡＳＥ女性のクリニック	104-042	東京都中央区入船1-1-24　5Ｆ	03-3206-1112	https://ease-clinic.jp	○	○	産科<br>婦人科<br>女性内科	丸山 真理子	
185	Wi clinic　銀座院	104-0061	東京都中央区銀座8－9－16長崎センタービル９階	0120-684-998	https://wi-clinic.com/	○	○	美容皮膚科・皮膚科	秋田　護	東京美容医療クリニック（東京都豊島区南池袋1－23－11池袋ブロンズビル１０階
186	AGクリニック銀座	104-0061	東京都中央区銀座4-10-1 HOLON-GINZA 4階	13-6278-8112	https://ginza-ginginclinic.com	○	○	泌尿器科<br>皮膚科	乙供　太郎	東京腎泌尿器センター大和病院（東京都板橋区本町36-3）聖路加国際病院（東京都中央区明石町9-1）
187	おりたメンタルクリニック	103-0011	東京都中央区日本橋大伝馬町13-8　メディカルプライム日本橋小伝馬町8F	03-5614-0756	https://orita-mental.com/	○	○	心療内科　精神科	織田　宗太郎	
188	勝どきウィメンズクリニック	104-0054	東京都中央区勝どき2-10-4宮野海運ビル４Ｆ	03-3536-7723	http://www.kachidoki-women.com/	○	○	産婦人科	松葉　悠子	
189	勝どき小平眼科	104-0054	東京都中央区勝どき1-8-1-213	03-5547-8010	http://www.kachidoki-ganka.com		○	眼科	小平奈利	

電話や情報通信機器を用いて診療を実施する医療機関の一覧（東京都）

	基本情報					事務連絡に基づく対応について				
	施設名	郵便番号	住所（都道府県から記載）	電話番号	ウェブサイトURL	初診の電話等を用いた診療の実施の有無	再診の電話等を用いた診療の実施の有無	対応診療科	担当医師名	対面診療が必要と判断した場合に連携する医療機関名（複数ある場合は複数、住所も併せて記載）
190	勝どき脳神経外科	104-0054	東京都中央区勝どき3-5-5　AIP勝どき駅前ビル３Ｆ	03-6204-9911	http://kachidokineurosurgery.jp		○	脳神経外科	藤塚光幸	
191	神谷町スキンケアクリニック	105-0001	東京都港区虎ノ門５－１３－７虎ノ門Ａ＆Ｋ－ＩＰビル２Ｆ	03-5777-1102	http://kamiyacho-skincare.jp/	○	○	皮膚科	太田　美佳	
192	亀田京橋クリニック	104-0031	東京都中央区京橋三丁目1番1号<br>東京スクエアガーデン4階・6階	0570-018-000	http://www.kameda-kyobashi.com		○	内科	金子　教宏<br>郭　友輝<br>難波　雄亮<br>吉澤　瑛子	亀田総合病院（千葉県鴨川市東町929）<br>亀田クリニック（千葉県鴨川市東町1344）
193	茅場町パークサイドクリニック	103-0025	東京都中央区日本橋茅場町2-2-1東石ビル１階	03-3666-2212	https://www.dr-sakurai.com/		○	内科	櫻井達也	
194	銀座スキンクリニック	104-0061	東京都中央区銀座３－３－１４銀座グランディアビル3階	0120-4103-53	https://www.ginzaskin.com	○	○	皮膚科<br>美容皮膚科	坪内利江子	
195	銀座すずらん通りレディスクリニック	104-0061	東京都中央区銀座6-9-7近畿建物銀座ビル8階	03-3569-7711	www.huninsho.com		○	産婦人科	小野美央子	
196	銀座並木通りクリニック	104-0061	東京都中央区銀座4-2-2　第1弥生ビル7F	03-3562-7773	https://www.ginzanamiki-clinic.com/	○	○	内科<br>脳神経外科	三好立<br>丸山隆志	
197	銀座ハートクリニック	03-5148-3921	東京都中央区銀座８－１６－５銀座中央ビル１Ｆ	03-5148-3921	https://ginza-heart.com		○	内科　循環器内科　心臓血管外科　小児科	永瀬裕三	聖路加国際病院（東京都中央区明石町９－１
198	銀座富士クリニック	104-0061	東京都中央区銀座４－１１－２　丸正ビル２階	03-3542-8371	http://ginzafuji.com/		○	内科	田所　賢也	

電話や情報通信機器を用いて診療を実施する医療機関の一覧（東京都）

	基本情報					事務連絡に基づく対応について				
	施設名	郵便番号	住所（都道府県から記載）	電話番号	ウェブサイトURL	初診の電話等を用いた診療の実施の有無	再診の電話等を用いた診療の実施の有無	対応診療科	担当医師名	対面診療が必要と判断した場合に連携する医療機関名（複数ある場合は複数、住所も併せて記載）
199	銀座よしえクリニック銀座二丁目院	104-0061	東京都中央区銀座2-5-11 V88ビルディング5階	0120-398-885	https://www.ginzabiyou.com/	○	○	皮膚科<br>美容皮膚科	担当医	
200	銀座よしえクリニック銀座本院	104-0061	東京都中央区銀座6-9-8銀座UKビル4F	0120-398-885	https://www.ginzabiyou.com/	○	○	皮膚科<br>美容皮膚科	担当医	
201	銀座レンガ通りクリニック	104-0061	東京都中央区銀座1－6－16銀座1616ビル４階	03-3535-2280	http://ginza-rengadori.com/	○	○	心療内科<br>精神科<br>内科	臼井　幸治	
202	公益財団法人<br>朝日生命成人病研究所附属医院	103-0002	東京都中央区日本橋馬喰町2-2-6朝日生命須長ビル	03-3639-5501	https://asahi-life.or.jp		○	糖尿病内科・循環器内科・消化器内科	各医師	
203	公益財団法人<br>早期胃癌検診協会附属茅場町クリニック	103-0025	東京都中央区日本橋茅場町2-6-12	03-3668-6800	http://www.soiken.or.jp		○	内科<br>消化器内科	各医師	
204	呼吸ケアクリニック東京	104-0031	東京都中央区京橋1-12-5　京橋YSビル1階	03-6263-2143	https://www.rcc-icr.com/		○	呼吸器内科<br>内科<br>アレルギー科	木田厚瑞・茂木孝・平松久弥子・森井恵子	聖路加国際病院（東京都中央区明石町9-1）
205	国立がん研究センター中央病院	104-0045	東京都中央区築地５－１－１	03-3542-2511	https://www.ncc.go.jp/jp/ncch/index.html		○	概ね全診療科	特定していない	
206	小坂こども元気クリニック	104-0052	東京都中央区月島3-30-3ベルウッドビル2～4階	03-5547-1191	http://e-kosaka.jp/	○	○	小児科　アレルギー科　内科	小坂　和輝	聖路加国際病院（東京都中央区明石町9-1）
207	さいとう内科・循環器クリニック	103-0011	東京都中央区日本橋大伝馬町13-8-3F	03-3639-3110	https://saito-heart.com/	○	○	内科<br>循環器内科<br>糖尿病内科	齋藤　幹	

電話や情報通信機器を用いて診療を実施する医療機関の一覧（東京都）

	基本情報					事務連絡に基づく対応について				
	施設名	郵便番号	住所（都道府県から記載）	電話番号	ウェブサイトURL	初診の電話等を用いた診療の実施の有無	再診の電話等を用いた診療の実施の有無	対応診療科	担当医師名	対面診療が必要と判断した場合に連携する医療機関名<br>（複数ある場合は複数、住所も併せて記載）
208	白倉内科診療所	104-0061	東京都中央区銀座 4-13-18 医療ビル1階	03-3543-4361	https://www.hankeikai.com/		○	内科	吉田　範子	
209	シロノクリニック銀座	104-0061	東京都中央区銀座2-2-2<br>ヒューリック西銀座ビル4F	0120-99-7474	https://www.shirono-ginza.net/	○	○	美容皮膚科	徳永　真理<br>笠井　美貴子	
210	豊海診療所	104-0055	東京都中央区豊海町2-2-113	03-3533-6751		○	○	内科	矢野芳和	
211	水天宮前いきいきクリニック内科･呼吸器内科	151-0053	東京都中央区日本橋蛎殻町1-29-6 水天宮前東急ビル2階	03-3665-1159	http://iki-iki.jp		○	内科<br>呼吸器内科	宮本　優美	
212	鈴木整形外科	104-0042	東京中央区入船1-2-8-310	03-3553-5967	http://suzuki-seke.byoinnavi.jp/pc/	○	○	整形外科	鈴木　秀和	
213	聖路加国際病院	104-8560	東京都中央区明石町9-1	03-3541-5151	http://hospital.luke.ac.jp/		○	呼吸器外科、呼吸器内科、感染症科、皮膚科、神経内科、脳神経外科、神経血管内治療科、整形外科、アレルギー・膠原病	各医師	
214	月島シーサイドクリニック	104-0051	東京都中央区佃2-20-5<br>月島医療ステーションB1F,1F	03-6228-2234	https://tsukishima.clinic/	○	○	内科	保阪　政樹	聖路加国際病院（東京都中央区明石町9-1）<br>昭和大学江東豊洲病院（東京都江東区豊洲5-1-38）
215	たいようクリニック八重洲院	104-0028	東京都中央区八重洲2丁目6-21 三徳八重洲ビル4階	03-6265-1667	https://home.sun-cl.jp/	○	○	泌尿器科	野口　真康	
216	高橋医院	104-0032	東京都中央区八丁堀３－２６－８　高橋ビル１階	03-3551-5955	https://hatchobori.jp/		○	内科	高橋友乃<br>高橋宏樹	聖路加国際病院　（東京都中央区明石町9-1）

電話や情報通信機器を用いて診療を実施する医療機関の一覧（東京都）

	基本情報					事務連絡に基づく対応について				
	施設名	郵便番号	住所（都道府県から記載）	電話番号	ウェブサイトURL	初診の電話等を用いた診療の実施の有無	再診の電話等を用いた診療の実施の有無	対応診療科	担当医師名	対面診療が必要と判断した場合に連携する医療機関名（複数ある場合は複数、住所も併せて記載）
217	武田クリニック東銀座診療所	104-0045	東京都中央区築地1-12-22　コンワビル4階	03-3542-4881	https://higagin.jp/	○	○	糖尿病内科	遠藤　温子<br>山本　貴子	
218	対馬ルリ子<br>女性ライフクリニック銀座	104-0061	東京都中央区銀座2-6-5　銀座トレシャス７F	03-3538-0270	http://w-wellness.com/ginza/	○	○	婦人科	対馬ルリ子	
219	東京心臓血管・内科クリニック	103-0013	東京都中央区日本橋人形町1-7-7 笠原ビルディング3階	03-5641-1177	https://shinzo-kekkan.clinic/	○	○	循環器内科<br>内科	柴山　謙太郎<br>堀内　優	
220	東京ひざ関節症クリニック<br>銀座院	104-0061	東京都中央区銀座5-3-12 壹番館ビル 7F	0120-013-712	https://www.knee-joint.net/clinic/	○	○	整形外科	輿石 暁<br>八代 忍	
221	内科総合クリニック人形町	103-0013	東京都中央区日本橋人形町1丁目６－１０　ユニコム人形町ビル1階	03-5843-6714	https://ningyocho-cl.com/	○	○	内科	藤田　英理	三井記念病院（東京都千代田区神田和泉町1番地）<br>聖路加国際病院（東京都中央区明石町９－１）<br>東京大学医学部付属病院（東京都文京区本郷７－３－１）<br>メデイカルスキャニング
222	中込眼科	104-0033	東京都中央区新川２－２２－６SJIビル５階	03-5542-5267	https://nakagomiganka.com		○	眼科	中込　豊	
223	日本橋えがわクリニック	103-0028	東京都中央区八重洲1-1-3寿ビル2階	03-5204-0311	https://nihonbashi-egawacl.com/	○	○	内科<br>整形外科	穎川一忠<br>二見一美<br>二見一平<br>根岸道子	聖路加国際病院（東京都中央区明石町9-1）<br>慈恵会医科大学病院（東京都港区西新橋3-19-18）<br>順天堂医院（東京都文京区本郷3-1-3）
224	日本橋カワマタ内科クリニック	103-0006	東京都中央区日本橋富沢町10-18-1階	03-3669-2626	kawamata-cl.com/	○	○	内科<br>循環器科<br>糖尿病	川俣博文	
225	日本橋・重田クリニック	103-0022	東京都中央区日本橋室町４-２-１３－５F	03-3241-4045			○	内科<br>胃腸科<br>消化器科<br>皮膚科<br>泌尿器科<br>肛門科	重田　　博	

電話や情報通信機器を用いて診療を実施する医療機関の一覧（東京都）

	基本情報					事務連絡に基づく対応について				
	施設名	郵便番号	住所（都道府県から記載）	電話番号	ウェブサイトURL	初診の電話等を用いた診療の実施の有無	再診の電話等を用いた診療の実施の有無	対応診療科	担当医師名	対面診療が必要と判断した場合に連携する医療機関名（複数ある場合は複数、住所も併せて記載）
226	日本橋内科・アレルギー科クリニック	103-0022	東京都中央区日本橋室町2-4-3日本橋室町野村ﾋﾞﾙ7階	03-3274-4159	http://www.nohonbashi-allergy.jp		○	内科<br>アレルギー科<br>呼吸器内科	竹内聡美	聖路加国際病院（東京都中央区明石町9-1）<br>三井記念病院（東京都千代田区神田和泉町1）
227	日本橋浜町耳鼻咽喉科	103-0007	東京都中央区日本橋浜町3-25-10 日本橋浜町ポストビル3F	03-6661-6088	http://hamacho-ent.com	○	○	耳鼻咽喉科	許　芳行	
228	日本橋馬喰町クリニック	103-0002	東京都中央区日本橋馬喰町1-6-1<br>JU SKY TOWER 日本橋 3F	03-5843-6275	http://www.nb-clinic.jp/		○	内科<br>糖尿病内科	中西　静佳	
229	日本橋室町三井タワー ミッドタウンクリニック	103-0022	東京都中央区日本橋室町3-2-1日本橋室町三井タワー７階	03-3231-2071	https://www.mtc-nihonbashi.jp	○		内科	畑　啓介	三井記念病院　（東京都千代田区神田和泉町1）
230	日本橋むらやまクリニック	103-0004	東京都中央区東日本橋1-1-4	03-3851-2473	https://www.junseikai-nmc.or.jp/	○	○	内科<br>整形外科<br>麻酔科	村山　清之<br>土居　真太郎	
231	人形町こどもクリニック	103-0014	東京都中央区日本橋蛎殻町1-9-1 アポテックビル１階	03-3669-2828	https://tsubasa-cello-trimboli.com/	○	○	小児科	田中　翼	当院に通院できる範囲での診療に限ります
232	人形町メンタルクリニック	103-0013	東京都中央区日本橋人形町1-1-21人形町ビル３F	03-5614-7087	http://www.cocoro-support.com/		○	精神科<br>心療内科	勝　久寿	
233	晴海3丁目クリニック	104-0053	東京都中央区晴海3-10-1　1F	03-3536-8633	https://www.harumi3-cl.jp/	○	○	内科　消化器内科　胃腸内科<br>内視鏡内科	平野　直樹	昭和大学江東豊洲病院　聖路加国際病院
234	晴海トリトン　夢未来クリニック	104-0053	東京都中央区晴海1-8-16晴海トリトンスクエア３F	03-3536-1361	https://yumemirai-clinic.net/	○	○	内科、アレルギー科	望月　さやか	聖路加国際病院<br>昭和大学江東豊洲病院

電話や情報通信機器を用いて診療を実施する医療機関の一覧（東京都）

	基本情報					事務連絡に基づく対応について				
	施設名	郵便番号	住所（都道府県から記載）	電話番号	ウェブサイトURL	初診の電話等を用いた診療の実施の有無	再診の電話等を用いた診療の実施の有無	対応診療科	担当医師名	対面診療が必要と判断した場合に連携する医療機関名（複数ある場合は複数、住所も併せて記載）
235	久松町まつうらクリニック	103-0005	東京都中央区日本橋久松町10-8　2F	03-6661-0024	https://www.matsuura-cl.net	○	○	内科　外科	松浦　裕史	
236	プライベートケアクリニック東京　東京院	103-0027	東京都中央区日本橋2-2-2マルヒロ日本橋ビル4F	03-5255-6611	https://pcct.jp/	○	○	性感染症内科	吉田　直人<br>三須　恵太<br>山本　藍<br>小堀　善友	
237	ヘルス・マネジメント・クリニック	104-0028	東京都中央区八重洲２－１１－２　城辺橋ビル１階	03-6271-8971	http://hmclinic-tokyo.org	○	○	内科<br>小児科	行松　伸成	
238	みらいクリニック	103-0001	東京都中央区日本橋小伝馬町９－６－３<br>松村ビル３階	03-3663-3366	https://mirai-clinic.org	○	○	内科	酒井　文彦	
239	人形町まつだ耳鼻咽喉科	103-0013	東京都中央区日本橋人形町2-2-3アライヴ人形町２Ｆ	03-6264-9762	http://ningyocho-matsuda-ent.com	○	○	耳鼻咽喉科	松田雄大	三井記念病院
240	みんなの内科クリニック<br>人形町・水天宮	103-0013	東京都中央区日本橋人形町2-2-3アライヴ人形町６階	03-3668-2200	https://minnano-naika.com	○	○	内科<br>腎臓内科	谷津　圭介	
241	八重洲大島眼科	104-0028	東京都中央区八重洲2-1八重洲地下街	03-6262-3996	http://www.yaesuoshima.com/		○	眼科	岡島　修	
242	八重洲形成外科・美容皮膚科	103-0027	東京都中央区日本橋3丁目４-14八重洲Ｎ３ビル4階	03-6265-1981	http://yaesukeisei.com/	○	○	形成外科・皮膚科	原かや	
243	八重洲街診療所	103-0027	東京都中央区日本橋2-2-20<br>日本橋仲通りビル3階	03-3271-1828	http://www.yaesugai.msn.gr.jp/		○	内科<br>泌尿器科	藤沢　民	昭和大学江東豊洲病院（東京都江東区豊洲5-1-38）<br>聖路加国際病院（東京都中央区明石町9-1）

電話や情報通信機器を用いて診療を実施する医療機関の一覧（東京都）

	基本情報					事務連絡に基づく対応について				
	施設名	郵便番号	住所（都道府県から記載）	電話番号	ウェブサイトURL	初診の電話等を用いた診療の実施の有無	再診の電話等を用いた診療の実施の有無	対応診療科	担当医師名	対面診療が必要と判断した場合に連携する医療機関名（複数ある場合は複数、住所も併せて記載）
244	吉井医院	104-0041	東京都中央区新富1-5-14	03-3551-2032	https://www.yoshii-iin.or.jp		○	内科	吉井　治<br>西田　友哉<br>福井　由希子	聖路加国際病院（東京都中央区明石町9-1）
245	リバーシティクリニック東京	104-0051	東京都中央区佃1-11-8　ピアウエストスクエア2F	03-6219-7771	https://rivercity-clinic.jp/		○	内科<br>消化器内科<br>婦人科	南川　里抄<br>栗田　昌裕	
246	リバーシティこどもクリニック	104-0051	東京都中央区佃二丁目１番－６　リバーシティＭスクエア　１Ｆ	03-5548-2323	http://rc-kodomo.jp/		○	小児科	設楽利二	聖路加国際病院
247	リバーシティすずき整形外科	102-0051	東京都中央区佃1-11-8ピアウエストスクエア2階264	03-3534-5678	http://suzuki-seikei.tokyo		○	整形外科<br>リハビリテーション科<br>リウマチ科	鈴木秀彦	
248	市川医院	104-0061	東京都中央区銀座２－１１－１９銀座市川ビル	03-3541-7202			○	内科<br>皮膚科<br>消化器科	市川　尚一	
249	銀座まいにちクリニック	104-0061	東京都中央区銀座４－１３－１１銀座M&Sビル4F	03-6260-6001	https://mainichiclinic.jp/	○	○	内科、泌尿器科、皮膚科	山本悠太	
250	東京アスボクリニック	104-0031	東京都中央区京橋2-2-1　京橋エドグラン24F	03-6665-0518	https://www.asbo.or.jp/	○	○	内科<br>整形外科<br>婦人科	板倉弘重<br>永江玄太<br>手取屋岳夫<br>神谷貫之	
251	東京トータル美容クリニック	104-0061	東京都中央区銀座8-10-2 ルアンビル8F	03-3573-7878	https://tokyo-tbc.jp/inquiry/	○		美容外科・皮膚科・形成外科	久保田　全	
252	銀座セントラルクリニック	104-0061	東京都中央区銀座1-15-4　銀座1丁目ビル5階	03-5579-5995	gcclinic.jp		○	消化器内科<br>肛門内科<br>内科	鈴木　康夫<br>年森　明子	昭和大学江東豊洲病院(東京都江東区豊洲5-1-38)

電話や情報通信機器を用いて診療を実施する医療機関の一覧（東京都）

	基本情報					事務連絡に基づく対応について				
	施設名	郵便番号	住所（都道府県から記載）	電話番号	ウェブサイトURL	初診の電話等を用いた診療の実施の有無	再診の電話等を用いた診療の実施の有無	対応診療科	担当医師名	対面診療が必要と判断した場合に連携する医療機関名（複数ある場合は複数、住所も併せて記載）
253	アイビークリニック虎ノ門	105-0001	東京都港区虎ノ門1-8-8	03-5501-1511	http://www.ivy-clinic.biz/	○	○	内科<br>外科<br>消化器科	畝村　さゆみ<br>畝村　泰樹	
254	あおいクリニック	105-0003	東京都港区西新橋3-19-12　メディコ西新橋ビル4階	03-6453-0039	https://aoiclinic.jp/	○	○	糖尿病内科<br>内科	森武美帆	
255	あおぞらクリニック新橋院	105-0004	東京都港区新橋2-16-1ニュー新橋ビル3階339	03-3506-8880	https://www.aozoracl.com/	○	○	性感染症内科	内田千秋	
256	あおば皮フ科クリニック	106-0045	東京都港区麻布十番1-5-8ヴェスタビル2F	03-6447-1290	https://aoba.clinic	○	○	皮膚科<br>内科	菊地さやか<br>川田裕人	済生会中央病院<br>（東京都港区三田1-4-17）
257	青山・表参道<br>睡眠ストレスクリニック	107-0062	東京都港区南青山5-1-22　青山ライズスクエア3階	03-6427-6062	https://omotesando-sleep.com		○	心療内科<br>精神科	中村 真樹	
258	青山メンタルクリニック	107-0062	東京都港区南青山3丁目１８－２１サンコーノビル４F	03-5772-9200	https://www.aoyama-mc.com/	○	○	心療内科<br>精神科	中村　稔<br>安宅　勝弘<br>杉原　玄一	
259	赤坂おだやかクリニック	107-6302	東京都港区赤坂5-3-1　赤坂BIZタワー2階	03-6234-511	http://www.junreikai.com	○	○	内科<br>循環器内科<br>呼吸器科	西澤　寛人<br>井上　宏一<br>林　光恵<br>伊藤　栄喜	
260	赤坂ファミリークリニック	107-0052	東京都港区赤坂2-15-15　赤坂プラザビル3階	03-5562-8825	http://www.afc.tokyo	○	○	小児科<br>内科	伊藤明子、<br>半下石美佐子、<br>安藤恵美子	
261	赤坂見附前田病院	107-0051	東京都港区元赤坂1-1-5	03-3408-1136	http://www.maeda-hospital.or.jp/		○	外科<br>内科<br>整形外科	前田　泉<br>岩郷　俊幸	

電話や情報通信機器を用いて診療を実施する医療機関の一覧（東京都）

	基本情報					事務連絡に基づく対応について				
	施設名	郵便番号	住所（都道府県から記載）	電話番号	ウェブサイトURL	初診の電話等を用いた診療の実施の有無	再診の電話等を用いた診療の実施の有無	対応診療科	担当医師名	対面診療が必要と判断した場合に連携する医療機関名（複数ある場合は複数、住所も併せて記載）
262	あべ耳鼻咽喉科クリニック	106-0032	東京都港区六本木6-1-20六本木電気ビル2F	03-3401-3387	http://www.abe-ent.jp	○	○	耳鼻咽喉科	阿部悦子<br>倉田奈都子	
263	アマラクリニック表参道	107-0062	東京都港区南青山3-10-38-101	03-6438-9869	http://www.amaraclinic.com/	○	○	精神科	松薗理英子	
264	有明こどもクリニック田町芝浦院	108-0023	東京都港区芝浦3-2-28-1階	03-6722-6300	https://child-clinic.or.jp	○	○	内科　耳鼻咽喉科<br>小児科	蛯名　彩<br>杉山　隆輔	
265	泉ウィメンズクリニック	107-0062	東京都港区南青山５－４－３南青山泉ビル３階	03-3400-1213	https://izumi-womens.jp/		○	婦人科	泉　康史	
266	医療法人財団玉川会<br>エム・オー・エー高輪クリニック	108-0074	東京都港区高輪4-8-10　MOA会館1階	03-5421-0021	https://itp.ne.jp/info/139940521116720110/	○	○	内科<br>心療内科	鈴木清志、畠山和幸、蒲原聖可、加藤眞三	
267	医療法人社団IHL<br>品川イーストワンメディカルクリニック	108-0075	東京都港区港南2-16-1　品川イーストワンタワー3F	03-6718-2898		○	○	内科	板倉　弘重<br>内山　明好<br>大西　真<br>白石　隆吉	NTT東日本関東病院（東京都品川区東五反田5-9-22）
268	医療法人社団スリープクリニック<br>スリープクリニック青山	107-0062	東京都港区南青山4-2-4ｼｬﾄｰ青山第3 101号室	03-5786-9700	https://www.sleepmedicine-tokyo.com/		○	精神科<br>心療内科	当日の担当医	
269	医療法人社団ケイセイ会<br>パークサイドクリニック	105-0011	東京都港区芝公園２－６－８<br>日本女子会館ビル２F	03-3434-7800	http://parkside-clinic.jp/		○	内科・外科<br>消化器科<br>糖尿病内科<br>循環器科	大原　成官<br>浅岡　弘	
270	医療法人財団順和会山王病院	107-0052	東京都港区赤坂8-10-16	03-3402-3151	https://www.sannoclc.or.jp/hospital/	○	○	内科	小田原　雅人（糖尿病内科）<br>石川　卓也（糖尿病内科）<br>竹中　恒夫（腎臓内科）<br>清水　伸幸（消化器センター）	山王病院の各診療科

電話や情報通信機器を用いて診療を実施する医療機関の一覧（東京都）

	基本情報					事務連絡に基づく対応について				
	施設名	郵便番号	住所（都道府県から記載）	電話番号	ウェブサイトURL	初診の電話等を用いた診療の実施の有無	再診の電話等を用いた診療の実施の有無	対応診療科	担当医師名	対面診療が必要と判断した場合に連携する医療機関名（複数ある場合は複数、住所も併せて記載）
271	医療法人社団松柏会　藤田クリニック	107-0062	東京都港区南青山2-22-19　三和青山ビル8階	03-5772-7381	http://www.fujita-clinic.jp/index.html		○	内科<br>小児科	藤田耕一郎	
272	医療法人社団勝優会 たまちホームクリニック	108-0073	東京都港区三田3-1-4 Net.1三田ビル1階	03-6435-2331	http://www.shoyuukai.jp/tamachi/	○	○	内科・消化器科・泌尿器科	菰池 信彦	
273	医療法人社団<br>PrimePartners<br>新橋ファーストクリニック	105-0004	東京都港区新橋3-14-5　S'TEC3ビル　3階	03-5777-5065	https://first-clinic.jp/	○	○	泌尿器科(自由診療)<br>皮膚科(自由診療)	市村　明	
274	医療法人社団癒合会<br>高輪和合クリニック	108-0074	東京都港区高輪4-22-10　小川商事ビル２階	03-5447-8521	https://www.takanawa-clinic.com/	○	○	内科	陰山　康成	
275	インターナショナルヘルスケアクリニック	105-0004	東京都港区新橋2-10-5末吉ビル３階	03-3501-1330	https://www.ihc-clinic.jp/	○	○	内科	鷲尾美香	NTT東日本関東 〒141-0022 東京都品川区東五反田５丁目９−２２<br>虎の門病院　〒105-8470東京都港区虎ノ門2丁目2-2<br>都立広尾病院　〒150-0013 東京都渋谷区恵比寿２丁目３４−１０<br>けいゆう病院（〒220-8521 神奈川県横浜市西区みなとみらい３丁目７−３
276	大村ビル皮フ科	105-0004	東京都港区新橋2－5－6大村ビル2階	03-3503-2278	http://hifukaiin.at-ninja.jp/index.html		○	皮膚科	加曽利潤一郎	
277	おかだスマイルクリニック	105-0013	東京都港区浜松町2-3-1日本生命浜松町クレアタワー4F	03-6809-1725	https://okada-smile.com/	○	○	内科　外科<br>胃腸内科	岡田　慶吾	
278	岡野内科診療所	105-0004	東京都港区新橋1-18-14三洋堂本館ビル8階	03-3502-8060		○	○	内科<br>胃腸科<br>肛門科	岡野喜久夫	
279	お台場クリニック	135-0092	東京都港区台場1－5－7－105	03-5531-0731	http://www7b.biglobe.ne.jp/~odaiba-clinic/		○	内科、小児科外科、皮膚科	櫻井貞夫、櫻井美佐	

電話や情報通信機器を用いて診療を実施する医療機関の一覧（東京都）

	基本情報					事務連絡に基づく対応について				
	施設名	郵便番号	住所（都道府県から記載）	電話番号	ウェブサイトURL	初診の電話等を用いた診療の実施の有無	再診の電話等を用いた診療の実施の有無	対応診療科	担当医師名	対面診療が必要と判断した場合に連携する医療機関名（複数ある場合は複数、住所も併せて記載）
280	ガーデンクリニック広尾	107-0062	東京都港区南青山7-14-7-2F	03-6427-9198	www.gardenclinic-hiroo.com	○	○	皮膚科<br>アレルギー科	キョンチンファイ	
281	学校法人北里研究所　北里大学東洋医学総合研究所	108-8642	東京都港区白金5-9-1	03-3444-6161	https://www.kitasato-u.ac.jp/toui-ken/	○	○	漢方科	小田口浩<br>鈴木邦彦<br>星野卓之<br>森裕紀子<br>川鍋伊晃<br>石毛達也<br>伊東秀憲	北里大学北里研究所病院（東京都港区白金5-9-1）
282	眼科スターアイクリニック	107-0052	東京都港区赤坂2-11-15　第二堀内ビル４階	03-3505-9981	http://www.starseye.com/		○	眼科	スターン美千代<br>堀越　淑子	
283	銀座総合美容クリニック	105-0004	東京都港区新橋1-9-5 新橋M-SQUARE Bright 4F・5F	0120-972-335	https://www.gincli.jp/	○	○	自由診療<br>(AGA)	正木 健太郎<br>須貝 歩	
284	クリニックフォア新橋	105-0004	東京都港区新橋１丁目４-番5号 ヒューリックG10ビル 9F	03-4579-9029	https://www.clinicfor.life/shimbashi/	○	○	内科<br>皮膚科<br>アレルギー科<br>美容皮膚<br>生活習慣病	金子和真<br>圓山尚	
285	クリニックフォア田町	108-0023	東京都港区芝浦３丁目１-３２ なぎさテラス 2F／4F	03-4579-8189	https://www.clinicfor.life/tamachi/	○	○	内科<br>皮膚科<br>アレルギー科<br>生活習慣病<br>専門外来（甲状腺・いびき）	村丘寛和<br>金子和真	
286	国際医療福祉大学三田病院	108-8329	東京都港区三田1-4-3	03-3451-8121	http://mita.iuhw.ac.jp/		○	内科、[illegible]臓内科、糖尿病内科、内分泌内科、血液内科、呼吸器内科、呼吸器外科、消化器内科、消化器外科、循環器内科、脳神経内[illegible]	各担当医	
287	国家公務員共済組合連合会<br>虎の門病院	105-8470	東京都港区虎ノ門2-2-2	03-3588-1111	https://www.toranomon.gr.jp/		○	全診療科		
288	サニーガーデンこどもクリニック	106-0045	東京都港区麻布十番2-18-8-6F	03-6722-6623	https://sunnygardencl.com/	○	○	小児科	首里　京子	

電話や情報通信機器を用いて診療を実施する医療機関の一覧（東京都）

	基本情報					事務連絡に基づく対応について				
	施設名	郵便番号	住所（都道府県から記載）	電話番号	ウェブサイトURL	初診の電話等を用いた診療の実施の有無	再診の電話等を用いた診療の実施の有無	対応診療科	担当医師名	対面診療が必要と判断した場合に連携する医療機関名（複数ある場合は複数、住所も併せて記載）
289	しおどめ眼科	105-0021	東京都港区東新橋1-8-2カレッタ汐留B1F	03-6228-5821	www.shiodomeganka.jp		○	眼科<br>内科	岩間　真由美	東京慈恵会医科大学付属病院
290	芝浦クリニック	108-0023	東京都港区芝浦3-16-1　中野興産ビル1階	03-5765-5264	http://shibaura-clinic.net		○	内科皮膚科整形外科	宇治原	
291	品川イーストクリニック	108-6102	東京都港区港南2-15-2 品川インターシティB棟2階	03-5783-5521	e-clinic.gr.jp	○	○	内科<br>眼科	本郷/二宮/中谷	
292	品川胃腸肛門内視鏡クリニック	108-0074	東京都港区高輪3-8-17　メディカルプライム高輪2階	03-3447-6337	https://www.gutclinic.jp/	○	○	消化器内科<br>内視鏡内科	望月　暁	NTT東日本関東病院(東京都品川区東五反田５丁目９−２２)<br>済生会中央病院(東京都港区三田1-4-17)
293	湘南美容クリニック新橋銀座口院	105-0004	東京都港区新橋1-12-9 新橋プレイス９階	0120-546695	https://www.s-b-c.net/clinic/branch/shinbashi/	○	○	美容皮膚科	竹田啓介<br>中山昌大<br>内田　悠記<br>富田さくら<br>大貝　明日香	湘南美容クリニック各院
294	湘南内科クリニック六本木院	106-0032	東京都港区六本木6-1-24 ラピロス六本木8階	0120-228-622	https://www.sbc-medicalcare-roppongi.com/	○	○	美容皮膚科・美容内科・内科・脂質代謝内科	御園生 佳奈子	湘南美容クリニック各院
295	湘南美容クリニック六本木院	106-0032	東京都港区六本木6-1-24 ラピロス六本木8階	0120-490089	https://www.s-b-c.net/clinic/branch/roppongi/	○	○	美容皮膚科	生水幹憲<br>小泉　彰郎<br>辻　大成	湘南美容クリニック各院
296	しろかねたかなわクリニック	108-0072	東京都港区白金1-13-11	03-6721-7561	https://shirokanetakanawa.jp		○	精神科<br>心療内科	木村元紀<br>松島英介<br>市田典子	
297	新田町ビル診療所	108-0014	東京都港区芝5-34-6-2F	03-3451-2619			○	内科 皮膚科	坪田　淳　雅子	

電話や情報通信機器を用いて診療を実施する医療機関の一覧（東京都）

	基本情報					事務連絡に基づく対応について				
	施設名	郵便番号	住所（都道府県から記載）	電話番号	ウェブサイトURL	初診の電話等を用いた診療の実施の有無	再診の電話等を用いた診療の実施の有無	対応診療科	担当医師名	対面診療が必要と判断した場合に連携する医療機関名（複数ある場合は複数、住所も併せて記載）
298	しんばし内科・脳神経クリニック	105-0004	東京都港区新橋1-17-2、ダイワロイネットホテル新橋地下1階	03-3507-6066	http://shimbashiclinic.jp	○	○	内科・脳神経科	星　明彦	
299	心療内科・神経科赤坂クリニック	107-0052	東京都港区赤坂3-9-18　B.I.C赤坂ビル6F	03-5575-8198	http://fuanclinic.com/akasaka/		○	心療内科<br>精神科<br>小児神経科	貝谷久宣<br>以下16名	
300	菅原クリニック東京脳ドック	107-6102	東京都港区赤坂5-2-20　赤坂パークビル2階	03-5573-8822	https://tokyo-noudock.jp/	○	○	内科<br>脳神経外科	菅原　道仁<br>伊藤　たえ	
301	高輪台レディースクリニック	108-0074	東京都港区高輪３－８－１７　TK高輪ビル3階	03-3441-1377	http://takanawadai-lc.com		○	産科・婦人科	瀧澤　慎<br>瀧澤　芳子	
302	田町メンタルクリニック	108-0014	東京都港区芝5-34-6新田町ビル2階	03-6809-4132	http://www.tamachi-mental.com/		○	精神科<br>心療内科	網野　賀一郎<br>丸山　志織	
303	東京シティクリニック三田	108-0014	東京都港区芝4-5-8　池藤ビル2F	03-5439-5566	https://www.tokyocityclinic-mita.com/	○	○	小児科<br>アレルギー科<br>内科	世間瀬　基樹	
304	東京慈恵会医科大学附属病院	105-8471	東京都港区西新橋3-19-18	03-3433-1111	https://www.hosp.jikei.ac.jp/		○<br>慢性疾患を有する再診患者が対象で医師が必要と判断した場合	全診療科	各医師	
305	東京都済生会中央病院	108-0073	東京都港区三田1-4-17	03-3451-8211	http://www.saichu.jp		○	全診療科	全担当医師	
306	東京ミッドタウンクリニック	107-6206	東京都港区赤坂9-7-1 ミッドタウン・タワー6F	03-5413-0080	https://www.tokyomidtown-mc.jp/	○	○	内科	各担当医師	東京医大・国際医療福祉大学三田病院・日本医大・癌研有明・日赤医療センター・NTT東日本病院

電話や情報通信機器を用いて診療を実施する医療機関の一覧（東京都）

	基本情報					事務連絡に基づく対応について				
	施設名	郵便番号	住所（都道府県から記載）	電話番号	ウェブサイトURL	初診の電話等を用いた診療の実施の有無	再診の電話等を用いた診療の実施の有無	対応診療科	担当医師名	対面診療が必要と判断した場合に連携する医療機関名（複数ある場合は複数、住所も併せて記載）
307	ながさき内科医院	03-3435-5707	東京都港区浜松町2－3－1　日本生命クレアタワー4階	03-3435-5707	https://www.nagasakinaikaiin.com/	○	○	内科	長崎綾子	東京都済生会中央病院　東京慈恵会医科大学付属病院　東京都立広尾病院
308	にしや耳鼻咽喉科クリニック	108-0075	東京都港区港南2-16-7 品川Vタワーメディカルゾーン２F	03-5715-0248	http://www.ent248.com/index.php	○	○	耳鼻咽喉科	西谷　全弘	
309	ピンクリボンブレストケアクリニック表参道	107-0061	東京都港区北青山3-6-19　バイナリー北青山2階	03-3407-7373	http://pinkribbon-breastcare.com/	○	○	乳腺外科<br>産婦人科	島田　菜穂子<br>宮城　由美<br>田村　宜子<br>石山　めぐみ<br>谷川　輝美<br>尾身　牧子	
310	古川橋病院	106-8581	東京都港区南麻布2-10-21	03-3453-5011	http://www.zai-kouseikai.or.jp		○	内科<br>整形外科		
311	ホロン鳥居坂クリニック耳鼻咽喉科アレルギー科	106-0045	東京都港区麻布十番1-5-8ｳﾞｪｽﾀﾋﾞﾙ4F	03-5770-8733	https://holon-cl.com	○	○	耳鼻咽喉科<br>アレルギー科	仲田　拡人	ホロン鳥居坂クリニック耳鼻咽喉科アレルギー科（東京都港区麻布十番1-5-8ヴェスタビル４Ｆ）
312	みなと芝クリニック	105-0014	東京都港区芝2-12-1 2F	070-1313-0555	http://www.minatoshiba-cl.com/	○	○	内科	川本　徹	
313	南青山TOKUNAKAクリニック	107-0062	東京都港区南青山3-16-1南青山巴ビル3階4階	03-6812-9480	https://tokunaka-clinic.com	○	○	皮膚科形成外科	徳中亮平	
314	南青山みみのクリニック	107-0062	東京都港区南青山3－3－16　ハレアカラ南青山１F	03-5772-6450	http://www.yamakawa-clinic.com	○	○	耳鼻咽喉科	山川　卓也	順天堂大学病院（東京都文京区本郷3-1-1）
315	森野クリニック	105-0004	東京都港区新橋1-18-19キムラヤ大塚ビル７F	03-3591-2200	https://morino-clinic.com	○	○	脳神経外科<br>内科<br>外科	森野　道晴	

電話や情報通信機器を用いて診療を実施する医療機関の一覧（東京都）

	基本情報					事務連絡に基づく対応について				
	施設名	郵便番号	住所（都道府県から記載）	電話番号	ウェブサイトURL	初診の電話等を用いた診療の実施の有無	再診の電話等を用いた診療の実施の有無	対応診療科	担当医師名	対面診療が必要と判断した場合に連携する医療機関名（複数ある場合は複数、住所も併せて記載）
316	やべ耳鼻咽喉科表参道	107-0062	東京都港区南青山５－１０－１９－５F	03-3409-3387	http://www.yabe-3387.com	○	○	耳鼻咽喉科<br>アレルギー科	矢部多加夫	
317	六本木レディースクリニック	106-0032	東京都港区六本木7-15-17 ユニ六本木ビル3階	0120-853-999	https://www.sbc-ladies.com/	○	○	婦人科	山本　篤	湘南美容クリニック各院
318	浅田レディース品川クリニック	108-0075	東京都港区港南２-３-１３ 品川フロントビル3F	03-3472-2203	https://ivf-asada.jp/		○	婦人科	吉岡　陽子	
319	医療法人社団ハクナマタタ ぽれぽれクリニック	108-0074	東京都港区高輪1-5-21-4階	03-5422-7626	https://polepole-clinic.com	○	○	小児科・内科	三浦　麻子	愛育病院（東京都港区芝浦1-16-10）
320	みちとせクリニック	107-0062	東京都港区南青山三丁目8-13 1F-S	03-6434-9320	https://michitoseclinic.jp/	○	○	漢方内科<br>漢方小児科	堀田　広満	
321	内科　外科・優里医院	106-0047	東京都港区南麻布4-11-30　南麻布渋谷ビル7階	03-6450-3500			○	内科<br>循環器内科<br>外科	高橋　路子<br>大作　昌義<br>山田　好則	北里大学北里研究所病院（東京都港区白金5-9-1）
322	アイランド耳鼻咽喉科	163-1301	東京都新宿区西新宿6-5-1、<br>新宿アイランドタワー東館1階	03-5323-0252	http://aisyukai.com	○	○	耳鼻咽喉科	高野　愛弓	
323	あおぞらクリニック新宿院	160-0022	東京都新宿区新宿3-18-1しんじゅく一色ビル6階	03-3353-8800	https://www.aozoracl.com/shinjyuku	○	○	性感染症内科	福地裕三	あおぞらクリニック新橋院(東京都港区新橋2-16-1ニュー新橋ビル3階339)
324	あけぼの診療所	160-0001	東京都新宿区 片町2-3 菱和ビル	03-6457-7237	https://www.akebonoclinic.net/	○	○	内科<br>循環器内科<br>腎臓内科<br>皮膚科<br>形成外科<br>リハビリテーション科	下山祐人	

電話や情報通信機器を用いて診療を実施する医療機関の一覧（東京都）

	基本情報					事務連絡に基づく対応について				
	施設名	郵便番号	住所（都道府県から記載）	電話番号	ウェブサイトURL	初診の電話等を用いた診療の実施の有無	再診の電話等を用いた診療の実施の有無	対応診療科	担当医師名	対面診療が必要と判断した場合に連携する医療機関名（複数ある場合は複数、住所も併せて記載）
325	曙ホームクリニック	162-0054	東京都新宿区河田町3-8 THE DENS曙橋204	03-5357-7656	http://akebono-home.jp	○	○	脳神経内科<br>内科	余郷　麻希子	
326	阿蘇医院	160-0008	東京都新宿区四谷三栄町1-5	03-3351-3224	www.aso.iin.com/		○	内科	阿蘇　大輔	
327	あべメディカルクリニック	162-0811	東京都新宿区水道町4-29宝ビル1階	03-6228-1783	https://abemc.jp/		○	内科皮膚科	阿部航<br>阿部郁子	
328	アルバ・メンタルクリニック	160-0022	東京都新宿区新宿3-32-5日原ビル7階	03-5360-7119	http://www.alba-mental.jp		○	精神科<br>心療内科	海老澤佐知江	
329	飯田橋中村クリニック	162-0822	東京都新宿区下宮比町3-2　日本精鉱ビル2F	03-5225-7119			○	内科<br>泌尿器科	中村剛<br>蔵本美与子	
330	いしい内科・外科クリニック	160-0023	東京都新宿区西新宿7-1-10 守矢ビル5F	03-5937-3361	https://ishii-cl.net/	○	○	内科<br>外科	石井　雄二	
331	磯貝クリニック	169-0051	東京都新宿区西早稲田2-4-8	03-3232-1776		○	○	内科	磯貝祐貴子	区内医療機関
332	一般社団法人　日本家族計画協会市谷クリニック	162-0843	東京都新宿区市谷田町1-10保健会館新館2階	03-3235-2694	http://www.jfpa-clinic.org/		○	婦人科	北村　邦夫	
333	いなげ眼科	169-0075	東京都新宿区高田馬場1-21-10豊電ビル3階いなげ眼科	03-6380-2668	http://inage-ganka.com/		○	眼科<br>小児眼科	稲毛佐知子	

電話や情報通信機器を用いて診療を実施する医療機関の一覧（東京都）

	基本情報					事務連絡に基づく対応について				
	施設名	郵便番号	住所（都道府県から記載）	電話番号	ウェブサイトURL	初診の電話等を用いた診療の実施の有無	再診の電話等を用いた診療の実施の有無	対応診療科	担当医師名	対面診療が必要と判断した場合に連携する医療機関名（複数ある場合は複数、住所も併せて記載）
334	いやま眼科	162-0811	東京都新宿区水道町4-13 三晃ビル3F クリニックステーション江戸川橋	03-5946-8188	https://www.iyama-eyeclinic.com/	○	○	眼科	井山　千草	
335	医療法人財団東京勤労者医療会　おおくぼ戸山診療所	162-0072	東京都新宿区大久保2-8-2	03-5272-2585	http://www.tokyo-kinikai.com/02jigyosyo/jigyosyo_02.html		○	内科	梅津　仁	
336	医療法人社団あけぼの会　山本医院	161-0033	東京都新宿区下落合４-23-21	161-0033	http://www.yamamotoiin.org/		○	皮膚科<br>泌尿器科	山本史郎	聖母病院（東京都新宿区中落合2-5-1）<br>JCHO東京山手メディカルセンター（東京都新宿区百人町3-22-1）
337	医療法人社団石川記念会　新宿石川クリニック	160-0023	東京都新宿区西新宿1-7-1<br>松岡セントラルビル6・7階	03-3340-1751	https://ishikawa-hp.com/shinjuku/		○	糖尿内科<br>泌尿器科<br>移植外来<br>内分泌科<br>腎臓内科<br>循環器科	三浦順子<br>哲翁たまき<br>佐中真由実<br>丸山聡子<br>尾形真紀子<br>勝盛弘三<br>三木誠<br>小山一郎	
338	医療法人社団医進会小田クリニック	169-0072	東京都新宿区大久保1-11-3大東ビル2階	03-5273-0770	https://www.ishinkai-mc.net		○	内科	髙崎医師	国立国際医療研究センター病院<br>国立国際医療研究センター病院
339	医療法人社団慶洋会　ケイアイクリニック	160-0004	東京都新宿区四谷1-20-23ケイアイメディカルビル	03-5269-2111	http://kiclinic.jp/kic/kenshin.html	○	○	内科/整形外科/婦人科/泌尿器科	黒瀬　嚴・石原雅巳・齋藤　英胤・篠澤　妙子・吉田　えり/河合　桃太郎・佐々木正・岸田　俊一・高橋　慶行	慶應義塾大学病院（東京都新宿区信濃町35）<br>国際医療福祉大学三田病院（東京都港区三田1-4-3　）<br>国立国際医療研究センター（東京都新宿区戸山1-21-1）
340	医療法人社団慶潤会ＫＤＤＩビルクリニック	163-8003	東京都新宿区西新宿　2-3-2　KDDIビル1階	03-6386-0123	http://www.keijunkai-kddi.jp		○	内科	林　潤一<br>岩橋尚子<br>中村美波子<br>五十嵐悠一<br>五十嵐慶子<br>加藤真由子	
341	医療法人社団慶潤会　新都心十二社クリニック	160-0023	東京都新宿区西新宿5-5-1-203<br>ｻﾞ・ﾊﾟｰｸﾊｳｽ西新宿ﾀﾜｰ60	03-3377-3033	http://www.keijunkai.or.jp		○	内科	久米雅彦	
342	医療法人社団広恵会　春山記念病院	169-0073	東京都新宿区百人町1-24-5	03-3363-1661	https://www.haruyama-hosp.com		○	内科<br>外科	清川 隆<br>山田 省一	

電話や情報通信機器を用いて診療を実施する医療機関の一覧（東京都）

	基本情報					事務連絡に基づく対応について				
	施設名	郵便番号	住所（都道府県から記載）	電話番号	ウェブサイトURL	初診の電話等を用いた診療の実施の有無	再診の電話等を用いた診療の実施の有無	対応診療科	担当医師名	対面診療が必要と判断した場合に連携する医療機関名（複数ある場合は複数、住所も併せて記載）
343	医療法人社団恒正会<br>そねクリニック新宿	160-0022	東京都新宿区新宿三丁目32番8号 新宿中央ロビル3階	03-3356-3888	https://www.soneclinic.com/		○	内科	曽根　正好	
344	医療法人社団玄英会<br>新宿ウエストクリニック	160-0023	東京都新宿区西新宿7-9-7<br>丸山ビル5F	03-5330-4079	https://www.westcl.com/	○	○	形成外科、美容外科、泌尿器科、皮膚科	入江武志	
345	医療法人社団順正会<br>ヒロオカクリニック	160-0022	東京都新宿区新宿2丁目5番12号　３Ｆ	03-3225-1666	http://www.h-cl.org/	○	○	内科	田原　稔<br>弘岡　泰正<br>弘岡　順子	
346	医療法人社団尚高会　西新宿きさらぎクリニック	160-0023	東京都新宿区西新宿6-6-2　新宿国際ビルディング1階	03-3344-0529	http://kisa-cl.com/		○	消化器内科　内視鏡内科　内科	石川 尚之<br>福澤 麻理	
347	医療法人社団新友会　プラザ３０階クリニック	160-0023	東京都新宿区西新宿2-2-1京王プラザホテル本館30階	03-5323-4330			○	内科<br>婦人科<br>眼科<br>皮膚科	髙橋　義德	
348	医療法人社団清慈会<br>牛込パーククリニック	162-0053	東京都新宿区原町1-18	03-6380-3387	https://ushigomepark-cl.com/	○	○	内科<br>・皮膚科<br>・歯科	猪瀬　悠理<br>/宮坂　厚弘<br>他各担当医師	国立国際医療研究センター（東京都新宿区戸山1-21-1）<br>東京慈恵医科大学付属病院（東京都港区西新橋3-19-18）<br>東京女子医科大学病院（東京都新宿区河田町8-1）
349	医療法人先進会<br>先進会眼科 東京	163-1335	東京都新宿区西新宿6-5-1<br>新宿アイランドタワー35F	03-5990-5212	https://senshinkai-clinic.jp/	○	○	眼科	カムラン　典子<br>比嘉　利沙子<br>石山　善三	
350	医療法人社団菱秀会<br>金内メディカルクリニック	160-0023	東京都新宿区西新宿7-5-25-2階	080-4166-9341	http://www.kmc.or.jp	○	○	内科	永積惇<br>木内麻里<br>金柿久里子	大久保病院（東京都新宿区歌舞伎町2-44-1
351	医療法人社団竹誠会　宗像クリニック	161-0033	東京都新宿区下落合1-6-1	03-3227-2510			○	内科	宗像　昭夫	

電話や情報通信機器を用いて診療を実施する医療機関の一覧（東京都）

	基本情報					事務連絡に基づく対応について				
	施設名	郵便番号	住所（都道府県から記載）	電話番号	ウェブサイトURL	初診の電話等を用いた診療の実施の有無	再診の電話等を用いた診療の実施の有無	対応診療科	担当医師名	対面診療が必要と判断した場合に連携する医療機関名（複数ある場合は複数、住所も併せて記載）
352	医療法人双葉会　新大久保　文化通り診療所	169-0073	東京都新宿区百人町2丁目5-8　科研ビル1F	03-5937-0610	http:www.shinookubo-bunkadori.clinic/		○	内科	畑田　康政	JCHO東京山手メディカルセンター
353	えどがわ橋内科・内視鏡クリニック	162-0811	東京都新宿区水道町4-13三晃ビル3階	03-5228-0750	https://edogawabashi-naishikyo.com/	○	○	内科<br>消化器内科	上條信也	
354	大内クリニックおおくぼ	169-0073	東京都新宿区百人町2-11-24　染矢グロービル3階	03-6279-2277	http://www.oouchihp.net/ookubo/		○	精神科	舟木　栄一	
355	太田医院	160-0008	東京都新宿区四谷三栄町9-6	03-5366-3213		○	○	内科	太田　正敏	
356	落合パークサイドクリニック	161-0034	東京都新宿区上落合1-1-4鈴木ビル4F	03-5338-4976	http://parkside-clinic.com	○	○	内科	外尾正俊	新宿区内の医療機関
357	オペラシティクリニック	163-1047	東京都新宿区西新宿3-20-2　東京オペラシティタワー7階	03-5353-7100	https://operacity-clinic.com/		○	内科	室賀一宏	
358	かおり内科クリニック	163-1090	東京都新宿区西新宿3-7-1　新宿パークタワーB1F	03-5321-6677	https://www.kaoriclinic.jp/	○	○	内科<br>リウマチ科	阿部　香織	
359	神楽坂医院	162-0825	東京都新宿区神楽坂3-6　近藤ビル１階	03-3269-7267	http://www.kagurazaka-iin.com	○	○	内科　整形外科<br>麻酔科	安部　通	
360	神楽坂こころのクリニック	162-0825	東京都新宿区神楽坂６－４８　ＴＯＭＯＳビル５階	03-6280-8407	http://www.kagurazaka-mc-com		○	心療内科<br>精神科	鈴木　英朗	

電話や情報通信機器を用いて診療を実施する医療機関の一覧（東京都）

	基本情報					事務連絡に基づく対応について				
	施設名	郵便番号	住所（都道府県から記載）	電話番号	ウェブサイトURL	初診の電話等を用いた診療の実施の有無	再診の電話等を用いた診療の実施の有無	対応診療科	担当医師名	対面診療が必要と判断した場合に連携する医療機関名（複数ある場合は複数、住所も併せて記載）
361	神楽坂内科<br>脳神経内科ｸﾘﾆｯｸ	162-0825	東京都新宿区神楽坂6-42神楽坂喜多川ビル1階	03-6280-7582	https://kagurazakanaika.wixsite.com/kagurazakaclinic	○	○	内科<br>脳神経内科	院長　坂本　智子	JCHO東京新宿メディカルセンター（東京都新宿区）<br>国立国際医療研究センター（東京都新宿区）
362	神楽坂みなみの眼科	162-0825	東京都新宿区神楽坂5-21-1長谷川ビル2階	03-6280-7690	http://www.minamino-ganka.com		○	眼科	南野　麻美	
363	久木田医院	161-0031	東京都新宿区西落合1-9-8	03-3952-5550	http://kukita-clinic.com/	○<br>受診歴のある方のみ	○	耳鼻咽喉科	久木田　尚仁	
364	栗原隆ウェルネスクリニック	1620044	東京都新宿区喜久井町4-1新宿印刷会館4階	03-6457-6572	https://www.dr-taka.jp/	○	○	内科	栗原隆	
365	慶應義塾大学病院	160-8582	東京都新宿区信濃町３５	03-3353-1211	http://www.hosp.keio.ac.jp/		○	外来診療を行う診療科	各診療科担当医	
366	国立国際医療研究センター病院	162-8655	東京都新宿区戸山1-21-1	03-3202-7181	http://www.hosp.ncgm.go.jp/index.html		○	呼吸器内科、消化器内科（消化管）、消化器内科（肝臓）、循環器内科、腎臓内科、神経内科、小児科、精神科、心療内科、糖尿病内分泌代謝科、膠原病科、血液内科、[illegible]	[illegible]一、柳瀬幹雄、廣井透雄、日ノ下文彦、新井憲俊、七野浩之、加藤温、菊地裕絵、梶尾裕、金子礼志、半下石明、山田和彦、清松知充、竹村信行、北川大、清[illegible]	
367	コンフォガーデンクリニック	162-0054	東京都新宿区河田町3-2	03-3357-0086	http://www.shokoukai.com/	○	○	内科<br>小児科	関口　聡子<br>及川　信哉	国立国際医療研究センター（東京都新宿区戸山1丁目21-1）
368	紫藤クリニック	169-0075	東京都新宿区高田馬場1-29-21みかどビル2階	03-3232-1622	https://www.shido-clinic.com/		○	精神科	紫藤　昌彦	
369	耳鼻咽喉科ヒロ医院	169-0075	東京都新宿区高田馬場3-2-5ＡＮビル7階	03-3369-3033	http://jibika-hiroiin.com		○	耳鼻咽喉科	宮坂宏恵	

電話や情報通信機器を用いて診療を実施する医療機関の一覧（東京都）

	基本情報					事務連絡に基づく対応について				
	施設名	郵便番号	住所（都道府県から記載）	電話番号	ウェブサイトURL	初診の電話等を用いた診療の実施の有無	再診の電話等を用いた診療の実施の有無	対応診療科	担当医師名	対面診療が必要と判断した場合に連携する医療機関名（複数ある場合は複数、住所も併せて記載）
370	耳鼻いんこう科<br>山西クリニック	162-0041	東京都新宿区早稲田鶴巻町518第一石川ビル2Ｆ	03-5155-0822	http://www.yamanishiclinic.jp/	○	○	耳鼻咽喉科	山西敏朗	国立国際医療研究センター（東京都新宿区戸山1-21-1）
371	湘南AGAクリニック西新宿院	160-0023	東京都新宿区西新宿7-21-3　西新宿大京ビル7階	0120-541-488	https://www.sbc-aga.jp/clinic/branch/agaoguard/?fd_bridge_id=emsrY1ltd1VJL3AyNEpSRmR2TXNYQT09LS1GallVeDYzeU9ndmd6d0p3R1l5TUJ3PT0%3D--		○	AGA<br>（自由診療）	曽我　俊介<br>阿部　吉伸<br>光川　泰弘<br>谷治　春香	湘南美容クリニック各院
372	湘南美容クリニック新宿本院	163-1324	東京都新宿区西新宿6-5-1　アイランドタワー24階	0120-548-940	https://www.s-b-c.net/clinic/branch/shinjuku/	○	○	美容皮膚科	西川　礼華<br>宮里　翔子<br>山本　彩<br>林　篤志<br>山本　しおり<br>完山　依里子<br>藤瀬　恵里香<br>太田　草子	
373	新宿ＮＳビルクリニック	163-0804	東京都新宿区西新宿２-４-１<br>新宿ＮＳビル４階	03-3343-3511	http://www.sakakibarakouseikai.com/ns/		○	内科	磯野　椋棒<br>小口　小船井<br>柴田　菊池　東<br>大武　橋本<br>市川　小原<br>田中　髙木	東京女子医科大学病院(東京都新宿区河和田町8-1)<br>大久保病院(東京都新宿区歌舞伎町2-44-1)<br>東海大学医学部付属東京病院(東京都渋谷区代々木1-2-5)<br>東京医大病院(東京都新宿区西新宿6-7-1)
374	新宿OP廣瀬クリニック	162-0054	東京都新宿区河田町７－６　新宿OPビル	03-6380-1280	hirose-clinic.net	○	○	精神科/心療内科	廣瀬久益/廣瀬圭太/廣瀬能華	廣瀬クリニック（茨城県水戸市見川町２３５２－３）
375	新宿オークタワークリニック	163-6002	東京都新宿区西新宿6-8-1 住友不動産新宿オークタワー2階	03-5381-0551	http://oaktowerclinic.com	○	○	内科<br>循環器内科<br>呼吸器内科<br>消化器内科<br>外科	田中　鶴人	東京大学医学部附属病院(東京都文京区本郷6-8-1)<br>東京逓信病院(東京都千代田区富士見2-14-23)<br>東京医科大学病院(東京都新宿区西新宿6-7-1)<br>JR東京総合病院(東京都渋谷区代々木)<br>大久保病院(東京都新宿区歌舞伎町2-44-1)
376	新小川町クリニック	162-0814	東京都新宿区新小川町6-40　入交ビル3階	03-3235-5265	https://www.hospita.jp/detail/934/		○	内科	早川和志	
377	新宿 睡眠・呼吸器内科クリニック	163-1312	東京都新宿区西新宿6-5-1　新宿アイランドタワー12階	03-5909-5588	http://www.shinjuku-sleep.jp		○	内科<br>呼吸器内科	遠藤大介	
378	新宿センタービル遠藤眼科	163-0055	東京都新宿区西新宿1-25-1新宿センタービル5F	03-3344-4146		○	○	眼科	遠藤耀子<br>山本美香(金曜)	新宿区内の総合病院

電話や情報通信機器を用いて診療を実施する医療機関の一覧（東京都）

	基本情報					事務連絡に基づく対応について				
	施設名	郵便番号	住所（都道府県から記載）	電話番号	ウェブサイトURL	初診の電話等を用いた診療の実施の有無	再診の電話等を用いた診療の実施の有無	対応診療科	担当医師名	対面診療が必要と判断した場合に連携する医療機関名（複数ある場合は複数、住所も併せて記載）
379	新宿センタービルクリニック	163-0605	東京都新宿区西新宿1-25-1　新宿センタービル5階	03-3346-1151	http://www.center-clinic.jp/		○	内科	平塚　伸	
380	新宿トミヒサクロス クリニック	162-0067	東京都新宿区富久町17-2 富久クロス1F	03-3353-2111	https://www.tomihisa-clinic.jp/	○	○	内科<br>小児科	三浦　崇幣	東京医科大学病院（東京都新宿区西新宿６－７－１）国立国際医療研究センター（東京都新宿区戸山1-21-1）大久保病院（東京都新宿区歌舞伎町2-44-1）
381	新宿東口眼科医院	160-0022	東京都新宿区新宿3-25-1　ヒューリック新宿ビル9階	03-5363-0507	http://www.shec.jp/		○	眼科	新川　恭浩<br>長谷川　二三代	東京医科大学病院（東京都新宿区西新宿6-7-1）<br>国立国際医療研究センター（東京都新宿区戸山1-21-1）
382	新宿東口クリニック	160-0022	東京都新宿区新宿3-24-1　新宿M-SQUARE10F	03-5366-3389	http://www.shinjukuc.com/		○	内科<br>皮膚科	山中　晃	
383	新宿ホームクリニック	160-0014	東京都新宿区内藤町1	03-6273-2109	https://shinjuku-home-clinic.com/	○	○	内科<br>整形外科	名倉　義人<br>菊池　亮	さくらクリニック（東京都世田谷区桜新町1-14ー22ー101）<br>レッツメディカルガーデンクリニック（東京都葛飾区細田5-15-6 アポロサンズビル1階）<br>九段下駅前まめクリニック（東京都千代田区九段北1丁目3-2 大橋ビル3階）<br>池袋東口まめクリニック（東京都豊島区東池袋１丁目４２－１２ ステーションサイド ビル３Ｆ）
384	新宿三井ビルクリニック	163-0404	東京都新宿区西新宿2-1-1 新宿三井ビル4階	03-3344-3311	https://www.sakakibarakouseikai.com/mitsui/		○	内科<br>呼吸器科	足立<br>菅原<br>斉藤<br>高倉<br>塩塚<br>丸山<br>柴田<br>菊池	東京女子医科大学病院（東京都新宿区河和田町8-1）<br>榊原記念病院（東京都府中市朝日町3-16-1）<br>国立国際研究医療センター（東京都新宿区戸山町1-21-1）
385	新宿南口皮膚科	160-0023	東京都新宿区西新宿1-18-7-3F	03-3343-4103	https://www.hifuka.com/		○	皮膚科	乃木田　辰俊	
386	助川クリニック	169-0075	東京都新宿区高田馬場1-5-10	03-3209-3333	https://sukegawa-clinic.com/	○	○	整形外科<br>内科<br>皮膚科	助川卓行<br>助川卓也<br>助川敦子<br>早稲田のぞみ	
387	住友内科クリニック	160-0007	東京都新宿区荒木町4-4森初ビル2階	03-5362-3288	http://sumitomo-clinic.jp/		○	内科	住友　高	国立国際医療研究センター病院（新宿区戸山1-21-1）

電話や情報通信機器を用いて診療を実施する医療機関の一覧（東京都）

	基本情報					事務連絡に基づく対応について				
	施設名	郵便番号	住所（都道府県から記載）	電話番号	ウェブサイトURL	初診の電話等を用いた診療の実施の有無	再診の電話等を用いた診療の実施の有無	対応診療科	担当医師名	対面診療が必要と判断した場合に連携する医療機関名（複数ある場合は複数、住所も併せて記載）
388	聖母病院	161-8521	東京都新宿区中落合2-5-1	03-3951-1111	http://www.seibokai.or.jp		○	内科/皮膚科/耳鼻咽喉科/外科/乳腺外科	担当医師	
389	高田馬場アイクリニック	169-0075	東京都新宿区高田馬場2-18-11 稲門ビル 2F	03-6457-6661	http://www.baba-eyeclinic.com/	○	○	眼科	荒木　寿子	東京医科大学病院<br>（〒160-0023 東京都新宿区西新宿６丁目７−１）<br>日本大学医学部附属板橋病院<br>（〒173-8610 東京都板橋区大谷口上町３０−１）
390	高橋医院	169-0074	東京都新宿区北新宿3-21-8	03-3371-8064		○	○	内科	高橋秀和	国立国際医療研究センター病院（東京都新宿区戸山1-21-1）
391	田中内科医院	160-0008	東京都新宿区四谷三栄町4-11 YSビル1階	03-3351-1815		○	○	内科<br>小児科	鹿井干城<br>樫田光夫<br>田中由利子	
392	たまきクリニック	160-0023	東京都新宿区西新宿1-12-12　河西ビル2階	03-3348-5888			○	心療内科<br>精神科	玉置　元	
393	寺尾クリニカ	169-0073	東京都新宿区百人町3-25-5グランドヒルズA	03-5338-9955	terao.clinica@brown.plala.or.jp	○	○	内科<br>心療内科アレルギー科<br>呼吸器科	寺尾　一郎	
394	東京女子医科大学東洋医学研究所クリニック	162-8666	東京都新宿区河田町8番1号	03-6709-9021	http://www.twmu.ac.jp/IOM/index.html		○	漢方内科	各医師	
395	東京都保健医療公社<br>大久保病院	160-8488	東京都新宿区歌舞伎町2-44-1	03-5273-7711	http://www.ohkubohospital.jp		○	概ね全診療科	各医師	
396	東京保健生活協同組合<br>江戸川橋診療所	162-0802	東京都新宿区改代町33番	03-3269-7855	http://www//thoken.or.Jp/edoshin//		○	内科	柴原　昭典	

電話や情報通信機器を用いて診療を実施する医療機関の一覧（東京都）

	基本情報					事務連絡に基づく対応について				
	施設名	郵便番号	住所（都道府県から記載）	電話番号	ウェブサイトURL	初診の電話等を用いた診療の実施の有無	再診の電話等を用いた診療の実施の有無	対応診療科	担当医師名	対面診療が必要と判断した場合に連携する医療機関名（複数ある場合は複数、住所も併せて記載）
397	富久クロス眼科	162-0067	東京都新宿区富久町17-2富久クロス1F	03-3351-1131	https://tomihisa-eye.jp/		○	眼科	早水　扶公子	
398	富久こどもハート・アレルギークリニック	162-0067	東京都新宿区富久町17－2	03-5362-1810	https://kodomo-heart.com/tomi/	○	○	小児科<br>アレルギー科<br>小児循環器内科	五十嵐　岳宏	国立国際医療研究センター（東京都新宿区戸山1-21-1）
399	なでしこ女性診療所	169-0075	東京都新宿区高田馬場1-1-1　メトロシティ西早稲田2F	03-6233-8228	https://nadeshiko-women.com/		○	婦人科<br>皮膚科	宮田智子	国立国際医療研究センター<br>東京山手メディカルセンター<br>東京女子医大病院<br>東大病院
400	西新宿コンシェリアクリニック	160-0023	東京都新宿区西新宿6-20-7コンシェリア西新宿タワーズウエスト2階	03-5323-8112	http://www.concieria-clinic.com		○	精神科	金子宏明	東京医大病院（東京都新宿区西新宿6-7-1）
401	西新宿さいとう眼科	160-0023	東京都新宿区西新宿1-5-11新宿三葉ビル7階	03-3343-8282	http://www.hospita.jp		○	眼科	齋藤　伸行　齊藤　康子　柴垣　三季	
402	西早稲田眼科	169-0075	東京都新宿区高田馬場1-1-1メトロシティ西早稲田2F	03-6380-2466	https://wwec.jp		○	眼科	寺井　和都	
403	丹羽クリニック	169-0074	東京都新宿区北新宿1-8-17ヒジカタビル2Ｆ	03-3368-7587		○	○	内科<br>アレルギー科<br>消化器内科	丹羽　正幸	東京山手メディカルセンター（東京都新宿区百人町3-22-1）<br>大久保病院（東京都新宿区歌舞伎町2-44-1）
404	抜弁天クリニック	162-0055	東京都新宿区余丁町8-7抜弁天ビル1階	03-5919-1522	http://www.nukebenten-clinic.com	○	○	内科<br>小児科	菊池　智津	国立国際医療研究センター（東京都新宿区戸山1-21-1）
405	ふたばクリニック	160-0017	東京都新宿区左門町20　四谷メディカルビル2階	03-3351-4133	http://www.futaba-ent.jp/	○	○	耳鼻咽喉科<br>小児科（再診のみ）<br>泌尿器科（再診のみ）	橋口一弘　綾美咲　入江　啓	慶応病院（東京都新宿区）<br>北里研究所病院（東京都港区）

電話や情報通信機器を用いて診療を実施する医療機関の一覧（東京都）

	基本情報					事務連絡に基づく対応について				
	施設名	郵便番号	住所（都道府県から記載）	電話番号	ウェブサイトURL	初診の電話等を用いた診療の実施の有無	再診の電話等を用いた診療の実施の有無	対応診療科	担当医師名	対面診療が必要と判断した場合に連携する医療機関名（複数ある場合は複数、住所も併せて記載）
406	光眼科医院	169-0073	東京都新宿区百人町2－5－8　科研ビル203	03-3360-2240		○	○	眼科	坊内　薫	
407	プライベートケアクリニック東京　新宿院	160-0032	東京都新宿区西新宿7-10-7加賀谷ビル3F	03-5337-6611	https://pcct.jp/	○	○	性感染症内科	鈴木　哲也<br>宮里　悠佑<br>中本　貴人<br>三須　恵太	
408	プライベートクリニック高田馬場	169-0075	東京都新宿区高田馬場1-28-4-4F	03-6908-8515	private-clinic.jp	○	○	内科・性感染症内科・美容皮膚科	鏡原裕一・梯勇希	
409	ほそかわ内科クリニック	161-0035	東京都新宿区中井2-20-7	03-3952-4181	http://hosokawa - naika - clinic.blogspot.jp/	○	○	内科	細川芳文	大久保病院（東京都新宿区歌舞伎町2-44-1）<br>聖母病院（東京都新宿区中落合２-5-1）
410	まさご眼科	164-0004	東京都新宿区四谷1-3　高増屋ビル4F	03-3350-3681	https://yotsuya.ganka.jp		○	眼科	真砂　めぐみ	
411	丸尾眼科	165-0025	東京都新宿区高田馬場３－３－１　ユニオン駅前ビル３階	03-3361-0015	http://www.maruoganka.com	○	○	眼科	丸尾敏之	
412	三越診療所	160-0023	東京都新宿区西新宿1-24-1エステック情報ビル	03-3348-5791	http://www.mhwf.or.jp/clinic/	○	○	内科	水野　杏一<br>山下　毅	国立国際医療研究センター病院（東京都新宿区戸山1-21-1）<br>日本医科大学付属病院（東京都文京区千駄木1-1-5）
413	ミラザ新宿つるかめクリニック	160-0022	東京都新宿区新宿3-36-10ミラザ新宿7階	03-6300-0063	https://mirrazatsurukamekai.jp/index.html		○	内科<br>消化器内科<br>糖尿病内科<br>婦人科	福島啓太<br>早瀬行治<br>杉原一明<br>加藤順子<br>福田達也<br>田嶋わか奈<br>佐野靖子	
414	目白ブライトクリニック	161-0033	東京都新宿区下落合3－21－3Mコート目白1Ｆ	03-3565-5788	mejiro-br.com	○	○	内科<br>外科<br>肛門科<br>消化器科	正岡　直子	

電話や情報通信機器を用いて診療を実施する医療機関の一覧（東京都）

	基本情報					事務連絡に基づく対応について				
	施設名	郵便番号	住所（都道府県から記載）	電話番号	ウェブサイトURL	初診の電話等を用いた診療の実施の有無	再診の電話等を用いた診療の実施の有無	対応診療科	担当医師名	対面診療が必要と判断した場合に連携する医療機関名（複数ある場合は複数、住所も併せて記載）
415	目白よしみクリニック	161-0033	東京都新宿区下落合3-12-23 豊ビル1F	03-5988-7667	http://yoshimi-clinic.com/		○	内科	金井 淑美	
416	もりた眼科小児科	160-0004	東京都新宿区四谷１－６－１－205B　コモレ四谷	03-6380-5363	https://www.morita-eye.com/	○	○	小児科	森田　玲子	
417	もりのぶ小児科	162-0812	東京都新宿区西五軒町8-10	03-6280-7158	https://www.nobu-kids.jp/	○	○	小児科	森　伸生<br>栗田 富美子<br>五十川 真歩	国立国際医療センター病院（東京都新宿区戸山1-21-1）
418	薬王寺アイクリニック	162-0063	東京都新宿区市谷薬王寺町70アルス市ヶ谷薬王寺1F	03-5227-7230	http://www.yakuoji-ic.jp/		○	眼科	高橋　眞理子	国立国際医療研究センター病院（東京都新宿区戸山1-21-1）<br>JCHO東京新宿メディカルセンター（東京都新宿区津久戸町5-1）
419	四谷クリニック	160-0004	東京都新宿区四谷1-2-30第二中村ビル３F	03-3226-9028		○	○	内科	福岡　稔晃	東京新宿メディカルセンター
420	四谷･血管クリニック	160-0004	東京都新宿区四谷1-8-14　四谷1丁目ビル2階	03-6380-6252	https://www.yotsuya-kekkanclinic.com/	○	○	内科<br>血管外科	保坂　純郎	
421	若松河田クリニック	162-0054	東京都新宿区河田町10-7-201	03-6457-8351	http://wakamatsukawadac.sakura.ne.jp/clinic/		○	内科<br>小児科	松岡瑠美子	東京女子医科大学病院（東京都新宿区河田町８－１）<br>国立国際医療研究センター（東京都新宿区戸山1-21-1）
422	若松町整形外科	162-0056	東京都新宿区若松町１０－１　日生ビル５Ｆ・６Ｆ	03-3207-5566			○	整形外科	清水　恒偉	
423	早稲田クリニック	162-0045	東京都新宿区馬場下町10早稲田レストハイツ2F	03-3208-2007	http://www.waseda-clinic.com	○	○	内科	小西　洋之	

電話や情報通信機器を用いて診療を実施する医療機関の一覧（東京都）

	基本情報					事務連絡に基づく対応について				
	施設名	郵便番号	住所（都道府県から記載）	電話番号	ウェブサイトURL	初診の電話等を用いた診療の実施の有無	再診の電話等を用いた診療の実施の有無	対応診療科	担当医師名	対面診療が必要と判断した場合に連携する医療機関名（複数ある場合は複数、住所も併せて記載）
424	早稲田大学保健センター	162-0041	東京都新宿区早稲田鶴巻町５３８－２１	03-3202-0580			○<br>（本学教職員または学生のうち定期受診者のみ）	内科<br>精神科	堀　正士<br>ほか20名	
425	早稲田メンタルクリニック	162-0042	東京都新宿区早稲田町69-4ウエステールワセダ5階	03-6233-9538	https://wasedamental.com/	○	○	精神科	益田　裕介	
426	はらまちクリニック	162-0053	東京都新宿区原町二丁目43番地	03-3341-8536			○	小児科	山川　陽子	
427	加藤レディスクリニック	160-0023	東京都新宿区西新宿7-20-3<br>ウエストゲート新宿ビル	03-3366-3777	https://www.towako-kato.com/		○	産婦人科	藤田　裕<br>佐藤　團<br>伊藤　正典	
428	マルファン・大動脈・ハートクリニック<br>青見循環器外科・内科	162-0052	東京都新宿区河田町７－１	03-3208-9340	https://www.marfan-aomi.jp	○	○	循環器外科<br>循環器内科	青見茂之	各県の大学病院や心臓外科のある病院
429	余丁町皮フ科	162-0055	東京都新宿区余丁町８－１６<br>ネオメディトピア３階	03-5312-1211	https://www.yochomachi-hifuka.com/	○	○	皮膚科	岩下　賢一	
430	あいそめクリニック	113-0022	東京都文京区千駄木3-40-17 CASA VERDE 2F	03-6314-5944	https://yanesen-kampo.jp	○		漢方内科	中西　幸子	
431	赤池耳鼻咽喉科	112-0011	東京都文京区千石４－４０－２	03-5976-3355	https://www.akaike-jibika.com<br>…	○	○	耳鼻咽喉科	赤池　洋	
432	いいの耳鼻咽喉科	113-0022	東京都文京区千駄木3-48-6	03-3828-3387	https://www.iino-jibika.com/	○	○	耳鼻咽喉科<br>アレルギー科	飯野　孝	

電話や情報通信機器を用いて診療を実施する医療機関の一覧（東京都）

	基本情報					事務連絡に基づく対応について				
	施設名	郵便番号	住所（都道府県から記載）	電話番号	ウェブサイトURL	初診の電話等を用いた診療の実施の有無	再診の電話等を用いた診療の実施の有無	対応診療科	担当医師名	対面診療が必要と判断した場合に連携する医療機関名（複数ある場合は複数、住所も併せて記載）
433	いいもりこころの診療所	113-0024	東京都文京区西片1-3-18	050-3734-5396	https://www.psycocoro.org/		○	精神科	飯森眞喜雄	
434	猪狩医院	113-0031	東京都文京区根津1-16-8-201	03-3822-4735		○	○	内科	山道博	
435	石原医院	112-0006	東京都文京区小日向1-6-6	03-3941-8526		○	○	内科・小児科	藤原　陽子<br>藤原　圭	JCHO東京新宿メディカルセンター<br>都立大塚病院<br>順天堂大学附属順天堂病院
436	一般財団法人近藤記念医学財団近藤記念医学財団<br>富坂診療所	113-0033	東京都文京区本郷1丁目３３－９	03-3814-2662	http://www.tomisaka.or.jp/		○	一般内科	岡本　保　若林香　小山　雄広<br>若井　布由子<br>柏木　佑介	
437	一般財団法人慈愛会<br>慈愛病院	113-0033	東京都文京区本郷６－１２－５	03-3812-7360	http://www.jiaibyoin.or.jp/		○	内科<br>皮膚科	吉田　勝俊<br>小林　中<br>山道　紀子<br>松本　美緒	日本医科大学付属病院（東京都文京区千駄木1-1-5 ）
438	医療法人社団同友会<br>春日クリニック	112-0002	東京都文京区小石川1-12-16ＴＧビル	03-3813-0080	http://www.do-yukai.com		○	内科	当日の担当医	
439	医療法人社団心緑会<br>小石川メンタルクリニック	112-0012	東京都文京区大塚3-6-5　白井ビル1～4F	03-3943-5858	http://www.koishikawa-mentalcl.com		○	精神科<br>心療内科	白井　麻理	
440	医療法人社団　静晴会<br>山田胃腸科外科医院	113-0031	東京都文京区根津1－16－10	03-3821-3381	http://www.ymc.or.jp		○	内科	中村秀紀 渡邉綾 佐々木隆 林高則 井原涼子<br>吉田俊太郎<br>梅本朋幸　ほか	
441	医療法人社団静美会<br>駒込かせだクリニック	113-0022	東京都文京区本駒込4-19-16<br>タウンハイム本駒込1F	03-5832-5665	http://www.kaseda-clinic.com/		○	内科<br>脳神経内科<br>呼吸器内科<br>アレルギー科	加勢田 美恵子<br>加勢田 靜<br>加勢田 幸子<br>岸田 修二	

電話や情報通信機器を用いて診療を実施する医療機関の一覧（東京都）

	基本情報					事務連絡に基づく対応について				
	施設名	郵便番号	住所（都道府県から記載）	電話番号	ウェブサイトURL	初診の電話等を用いた診療の実施の有無	再診の電話等を用いた診療の実施の有無	対応診療科	担当医師名	対面診療が必要と判断した場合に連携する医療機関名（複数ある場合は複数、住所も併せて記載）
442	(医)千秋双葉会　茗荷谷耳鼻咽喉科・アレルギー科	112-0002	東京都文京区小石川5-5-2バンビビル2階	03-3944-8711	http://myogadani-ent.com/	○	○	耳鼻咽喉科	長谷川　かおり	
443	医療法人社団　藤翔会加藤内科胃腸科クリニック	113-0021	東京都文京区本駒込6-1-21コロナ社第三ビル一階	03-5319-1470	http//www.katoh-climic.jp		○	内科<br>小児科<br>胃腸内科<br>消化器内科	加藤　裕昭	寺田医院（文京区西方）
444	医療法人社団　中野小児科内科	113-0023	東京都文京区白山1-33-23ベルメゾン文京白山1F	03-5805-8551	https://www.nakano-shounikanaika.com/		○	内科<br>小児科	中野圭介　中野起久恵	
445	医療法人社団森谷会森谷医院	113-0022	東京都文京区千駄木５－４３－１１	03-3821-0128	http://www.moritani-mc.com	○	○	内科<br>小児科	森谷茂樹	
446	医療法人緑寿会　小林クリニック	112-0002	東京都文京区小石川3-16-19	03-5689-2025	https://www.kobayashiclinic-koishikawa.com/		○	皮膚科<br>内科	小林寿美子<br>畑佳納子	
447	医療法人社団和幸会　上田医院	112-0013	東京都文京区音羽1丁目20-15	03-3943-0268	https://ueta-medical-clinic.jimdofree.com/		○	内科循環器科	上田　浩	東京新宿メディカルセンター(東京都新宿区津久戸町5-1)
448	うすだクリニック	113-0033	東京都文京区本郷4-36-5	03-5840-8250	http://www001.upp.so-net.ne.jp/usuda-c/		○	内科<br>小児科	薄田康広	
449	江戸川橋胃腸肛門クリニック	112-0014	東京都文京区関口1-19-6弥助ビル５F	03-3269-2020	https://www.edb-ichou.com/		○	胃腸科肛門科	森　康治	
450	江戸川橋さくらクリニック	112-0014	東京都文京区関口1-1-6	03-5227-3433	http://edogawabashi-sakura-clinic.com/	○	○	内科<br>消化器内科	千葉　悦子	国立国際医療研究センター<br>東京新宿メディカルセンター

電話や情報通信機器を用いて診療を実施する医療機関の一覧（東京都）

	基本情報					事務連絡に基づく対応について				
	施設名	郵便番号	住所（都道府県から記載）	電話番号	ウェブサイトURL	初診の電話等を用いた診療の実施の有無	再診の電話等を用いた診療の実施の有無	対応診療科	担当医師名	対面診療が必要と判断した場合に連携する医療機関名（複数ある場合は複数、住所も併せて記載）
451	えなのさとクリニック	112-0012	東京都文京区大塚3-1-10-206	03-5976-3065	https://www.enanosato.com/	○	○	内科、小児科、心療内科、皮膚科	福島幸江、山本享子	
452	大塚診療所	113-0034	東京都文京区湯島３－３１－６	03-3831-2294	http://www.ohtsukaclinic.com/	○	○	内科<br>小児科<br>アレルギー科	大塚　宜一	
453	御茶ノ水聖橋クリニック	113-0034	東京都文京区湯島1-9-15	03-5689-6811	https://www.i-seitoukai.or.jp/	○	○	内科・皮膚科・循環器科	林さなえ・橋本知加・林同文・今井靖・門前幸志郎・高木通乃・迫田智子	
454	春日駅前あべファミリークリニック	112-0002	東京都文京区小石川2-1-12<br>小石川トーセイビル6F	03-3830-0395	https://www.bunkyo-naika.com/	○	○	内科<br>脳神経外科<br>整形外科	阿部　琢巳	
455	春日眼科	112-0002	東京都文京区小石川1-13-12	03-3815-0177	http://www.kasugaeye.com	○	○	眼科	松井洋法	
456	きたなかこども成長クリニック	113-0033	東京都文京区本郷2-37-6　稲毛屋ビル4階	03-3817-0976	https://www.kitanaka-kidsgrowth.com/	○	○	小児科	北中幸子	
457	下司婦人科クリニック	113-0031	東京都文京区根津2-14-11 Tツインズビル4階	03-5832-4122	https://www.geshi-clinic.com/		○	産婦人<br>内科	下司有美	
458	小石川循環器内科	112-0002	東京都文京区小石川2-24-14	03-6801-8450	http://www/koishikawa-clinic.com	○	○	内科	鈴木　宏昌	順天堂医院（東京都文京区本郷2-1-1）
459	小石川柳町クリニック	112-0002	東京都文京区小石川1-13-9-103　クリオ文京小石川	03-5805-3749	https://www.koishikawa-cl.com	○	○	小児科　内科	近藤　千里<br>近藤　光子	都立大塚病院（〒170-8476 東京都豊島区南大塚２丁目８-1）

電話や情報通信機器を用いて診療を実施する医療機関の一覧（東京都）

	基本情報					事務連絡に基づく対応について				
	施設名	郵便番号	住所（都道府県から記載）	電話番号	ウェブサイトURL	初診の電話等を用いた診療の実施の有無	再診の電話等を用いた診療の実施の有無	対応診療科	担当医師名	対面診療が必要と判断した場合に連携する医療機関名（複数ある場合は複数、住所も併せて記載）
460	後楽園ウィメンズクリニック	113-0033	東京都文京区本郷１－３３－８ハウス本郷ビル4階	03-5689-5500	https://korakuen-womens.jp		○	産婦人科	佐々木　明香	
461	こひなた眼科	112-0006	東京都文京区小日向2-10-3	03-3944-5566	http://www.kohinata-eyeclinic.com		○	眼科<br>小児眼科	藤巻　拓郎	順天堂医院（東京都文京区本郷3-1-3）
462	こまごめ緑陰診療所	113-0021	東京都文京区本駒込5-19-2 小林ビルデンス2F	03-3943-5525	http://www.ryokuin.jp/		○	心療内科<br>精神科	福田博文<br>福田陽明	
463	坂巻クリニック	112-0005	東京都文京区水道2-14-7	03-3941-6074		○	○	内科	坂巻　壽	JCHO東京新宿メディカルセンター
464	しおざわクリニック	112-0006	東京都文京区小日向4-4-3-103	03-3945-3711	http://www//shiozwa-clinic.com	○	○	内科<br>泌尿器科<br>皮膚科<br>形成外科	塩澤　真司	順天堂医院(東京都文京区本郷3丁目1-3)<br>都立大塚病院(東京都豊島区南大塚2丁目8-1)<br>JCHO東京新宿メディカルセンター(東京都新宿区津久戸町5-1)<br>東京逓信病院（東京都千代田区富士見2丁目14-23）
465	順天堂大学医学部附属順天堂医院	113-8431	東京都文京区本郷3-1-3	03-3813-3111	https://www.juntendo.ac.jp/hospital/	○	○	初診：一部診療科<br>再診：全診療科	各医師	
466	心陽クリニック	113-0033	東京都文京区本郷２－２９－３　UK'sビル３F	03-6801-6160	http://www.shinyo.pro/contents/services/clinic/	○	○	内科	石田陽子	
467	水道橋ひふ科クリニック	113-0033	東京都文京区本郷1-14-4 AU南陽堂ビル1階	03-5684-4112	https://suido-hihu.com/	○	○	皮膚科	神島輪	
468	すずき医院	112-0015	東京都文京区目白台１－２３－７－１０１	03-3941-1590	http://www1.odn.ne.jp/suzuki-iin	○	○	内科<br>小児科	和智　行夫	

電話や情報通信機器を用いて診療を実施する医療機関の一覧（東京都）

	基本情報					事務連絡に基づく対応について				
	施設名	郵便番号	住所（都道府県から記載）	電話番号	ウェブサイトURL	初診の電話等を用いた診療の実施の有無	再診の電話等を用いた診療の実施の有無	対応診療科	担当医師名	対面診療が必要と判断した場合に連携する医療機関名（複数ある場合は複数、住所も併せて記載）
469	鈴木医院	113-0021	東京都文京区本駒込２－２８－３５	03-3941-6615			○	内科	鈴木弘之	
470	須田整形外科クリニック	112-0004	東京都文京区後楽2-23-15	03-3811-0881		○	○	整形外科<br>内科	須田　均	JCHO東京新宿メディカルセンター（新宿区津久戸町5-1）<br>順天堂大学医学部附属順天堂医院（文京区本郷3-1-3）
471	千駄木みしま眼科	113-0031	東京都文京区根津２－３７－４グランドメゾン根津1階	03-5834-7559	https://mishimaganka.com/	○	○	眼科	三嶋明香	
472	タツノ内科・循環器科	113-0033	東京都文京区本郷5-1-16　B1F	03-5800-0203	http://www.tatsuno-clinic.com		○	内科<br>循環器科	龍野　勝彦	
473	団子坂くつろぎクリニック	113-0022	東京都文京区千駄木5-21-12カサデコリーナ1階	03-5834-7702	http://www.kojikai.com	○	○	内科<br>アレルギー科	髙野夏希<br>岡本友喜<br>野呂林太郎<br>加藤泰裕	
474	千晶こどもクリニック	112-0002	東京都文京区小石川1-24-3	03-3868-0703	http://chiaki-childrenclinic.com	○	○	小児科	中井　千晶	
475	寺田医院	113-0024	東京都文京区西片1-2-3	03-3812-1821		○	○	内科　循環器科<br>皮膚科	弓　幸史	加藤内科胃腸科（千石）八千代診療所（白山）他基幹病院
476	寺本内科歯科クリニック	113-0033	東京都文京区本郷５－２５－１３	03-5684-0818	termoto-mdc.com		○	内科	寺本民生<br>脇裕則<br>若栗稔子	帝京大学病院（東京都板橋区）<br>東大病院（東京都文京区）
477	伝通院クリニック	112-0006	東京都文京区小日向４丁目６番１８号ステラメゾン若荷谷201	03-6912-1565	https://dentsuin.jp/	○	○	内科<br>小児科<br>耳鼻咽喉科	善積秀幸	

電話や情報通信機器を用いて診療を実施する医療機関の一覧（東京都）

	基本情報					事務連絡に基づく対応について				
	施設名	郵便番号	住所（都道府県から記載）	電話番号	ウェブサイトURL	初診の電話等を用いた診療の実施の有無	再診の電話等を用いた診療の実施の有無	対応診療科	担当医師名	対面診療が必要と判断した場合に連携する医療機関名（複数ある場合は複数、住所も併せて記載）
478	東京医科歯科大学医学部附属病院	113-8519	東京都文京区湯島1-5-45	03-3813-6111	http://www.tmd.ac.jp/medhospital/topics/tel/index.html		○	[illegible]ウマチ内科、糖尿病・内分泌・代謝内科、腎臓内科、総合診療科、消化器内科、循環器内科、呼吸器内科、緩和ケア科、食道外科、胃外科、大腸・肛門外科、消化器化学療法外科、乳腺外科、小児外科[illegible]	各科において対応	東京北医療センター（東京都北区赤羽4-17-56）小児科<br>練馬光が丘病院（東京都練馬区光が丘2-11-1）小児科<br>武蔵野赤十字病院（東京都武蔵野市境南町1-26-1）小児科<br>土浦協同病院（茨城県土浦市おおつ野4-4-1）小児科<br>JAとりで総合医療センター（茨城県取手市本郷2-1-1）小児科<br>川口市立医療センター（埼玉県川口市西新井宿180）小[illegible]
479	東京健生病院	112-0012	東京都文京区大塚4-3-8	03-3944-6111	http://www.thoken.or.jp/kensei/		○	内科<br>外科<br>整形外科<br>皮膚科<br>腎臓病<br>消化器<br>糖尿病<br>循環器	根岸京田、山崎広樹、加藤冠、岩下直樹、佐藤研路、小金丸千景、加藤貴、高岡和彦、松本剛、泉智子、谷口真知子、吉井[illegible]	大泉生協病院
480	東京大学医学部附属病院	113-8655	東京都文京区本郷7-3-1	03-3815-5411	https://www.h.u-tokyo.ac.jp/		○	かかりつけ診療科のみ	主治医	
481	東京都立駒込病院	113-8677	東京都文京区本駒込三丁目18番22号	03-3823-2101	http://www.cick.jp/		○	全科	各担当医	
482	東京保健生活協同組合セツルメント菊坂診療所	112-0002	東京都文京区小石川1-24-3	03-3812-0016	http://www.http://thoken.or.jp/kikusaka		○	内科	伊藤恵子<br>福間由美子	
483	東都文京病院	113-0034	東京都文京区湯島三丁目5番7号	03-3831-2181	https://www.tohtobunkyo-hp.com/		○	内科・心療内科・泌尿器・外科・整形外科・眼科・産婦人科・小児科・皮膚科・耳鼻咽喉科	[illegible]藤博紀、十菱大介、藤田恵、齊藤美奈子、野中英彰、新井政代、平尾龍彦、井上玲子、矢嶋由紀、建石綾子、石井聡、三浦雅臣、山口聡子、常吉秀男、河野史歩、小原啓子、山川仁子、山下和子、日下[illegible]	
484	内藤クリニック	113-0022	東京都文京区千駄木４－１４－１０－２F	03-5685-2455			○	精神科	内藤　志朗	
485	中村クリニック	112-0002	東京都文京区小石川3-27-6コスモ小石川103	03-3818-7677	clinic@nakamura.or.jp	○	○	消化器<br>外科<br>内科	中村宏	
486	似鳥クリニック	113-0031	東京都文京区根津1-19-11	03-3827-1181	https://www.nitadori.com/	○	○	呼吸器内科<br>循環器内科<br>小児科<br>脳神経外科	似鳥純一<br>服部和紀	東京大学医学部附属病院（東京都文京区本郷7-3-1）<br>三井記念病院（東京都千代田区神田和泉町１）

電話や情報通信機器を用いて診療を実施する医療機関の一覧（東京都）

	基本情報					事務連絡に基づく対応について				
	施設名	郵便番号	住所（都道府県から記載）	電話番号	ウェブサイトURL	初診の電話等を用いた診療の実施の有無	再診の電話等を用いた診療の実施の有無	対応診療科	担当医師名	対面診療が必要と判断した場合に連携する医療機関名（複数ある場合は複数、住所も併せて記載）
487	にわ医院	113-0022	東京都文京区千駄木4-8-12	03-3824-0482		○	○	内科<br>小児科	丹羽　明	
488	白山レディースクリニック	112-0001	東京都文京区白山5-36-9 白山麻の実ビル9F	03-5689-3070	https://hakusanladies.com/	○	○	婦人科	中村 久基	
489	はりまざかクリニック<br>耳鼻咽喉科･アレルギー科	112-0012	東京都文京区大塚1-5-18<br>大伴ビル9階A号	03-6304-1510	https://harimazaka.clinic	○	○	耳鼻咽喉科<br>小児耳鼻咽喉科<br>アレルギー科	三輪　正人	
490	日原内科クリニック	113-0022	東京都文京区千駄木2-13-1-２F	03-3822-6262	http://hihara-naika.byoinnavi.jp/pc/		○	内科<br>小児科	日原義文	
491	ふくだ皮ふ科	112-0002	東京都文京区小石川3-35-11	03-3868-2229	https://fukuda-hifu.jp	○	○	皮膚科	福田理永	
492	藤原クリニック	113-0022	東京都文京区千駄木3-2-7リエス千駄木1階地下1階	03-3821-3767	https://fujiwara-cl.jp/	○	○	内科<br>呼吸器内科<br>循環器内科<br>心療内科<br>精神科	藤原直之	藤原クリニック
493	文京動坂診療所	113-0022	東京都文京区千駄木4-15-3	03-3822-9680	http://www.bunkyo-dozaka.dr-clinic.jp		○	内科	五藤忠、岩澤邦明、明城正博、砂金秀章	
494	文京内科・循環器クリニック	113-0021	東京都文京区本駒込2-10-3-4F	03-6304-1567	https://www.bunkyo-cl.jp/	○	○	内科<br>循環器内科<br>糖尿病内科	藤原聡	日本医科大学附属病院(東京都文京区千駄木1-1-5)
495	保坂こどもクリニック	112-0001	東京都文京区白山5-27-12	03-3946-0641	http://hosaka-kodomo.com	○	○	小児科	保坂　篤人	

電話や情報通信機器を用いて診療を実施する医療機関の一覧（東京都）

	基本情報					事務連絡に基づく対応について				
	施設名	郵便番号	住所（都道府県から記載）	電話番号	ウェブサイトURL	初診の電話等を用いた診療の実施の有無	再診の電話等を用いた診療の実施の有無	対応診療科	担当医師名	対面診療が必要と判断した場合に連携する医療機関名（複数ある場合は複数、住所も併せて記載）
496	細部医院	113-0031	東京都文京区根津１－１－１９－２階	03-3822-6858	www.hosobeclinic.jp		○	泌尿器科	細部 高英	日本医科大学付属病院(文京区千駄木1-1-5)
497	細部小児科クリニック	113-0031	東京都文京区根津2-14-11 Tツインビル2階	03-6303-3162	http://www.hosobe-kodomo.com/	○	○	小児科	細部千晴	東大病院（文京区本郷７－３－１）<br>日医大（文京区千駄木１－１－５）<br>順天堂病院（文京区本郷３－１－３）<br>医科歯病院（文京区湯島１－５－４５）
498	本郷耳鼻咽喉科クリニック	113-0033	東京都文京区本郷4-2-8フローラビルディング２階	03-5689-4133	http://www.hongoent.com/	○	○	耳鼻咽喉科、アレルギー科	木村美和子	
499	本郷台皮膚科	113-0033	東京都文京区本郷５－１－１５　安野ビル３F	03-3812-4112	http://www.tcda.jp/hongoudai/		○	皮膚科	上田　純嗣	
500	本駒込耳鼻咽喉科	113-0021	東京都文京区本駒込3-17-2 木村ビル2階	03-3827-1187	https://www.honkomagome-jibi.com/	○	○	耳鼻咽喉科<br>小児耳鼻咽喉科	吉越　彬	
501	松本医院	113-0021	東京都文京区本駒込５－４１－１１　青木ビル1階	03-5832-9518	matsumoto-iinn.com		○<br>電話またはFAXのみ対応	内科<br>小児科<br>皮膚科<br>アレルギー科	松本　正隆	
502	三浦内科クリニック	112-0015	東京都文京区目白台2-12-5	03-5319-3321	http://www.miuranaikabunkyo.sakura.ne.jp/		○	内科	三浦　誠司	
503	光永クリニック	112-0011	東京都文京区千石４－４－１	03-3943-0270	http://www.mitsunagaclinic.com/		○	内科<br>小児科	光永　文<br>光永　眞人<br>光永　眞之	
504	茗荷谷キッズクリニック	112-0012	東京都文京区大塚3-1-6ラ・トゥール小石川B1F	03-5976-3077	http://myogadani-kids.com/	○	○	小児科<br>アレルギー科	有馬慶太郎	都立大塚病院（東京都豊島区南大塚2丁目8番1号）

電話や情報通信機器を用いて診療を実施する医療機関の一覧（東京都）

	基本情報					事務連絡に基づく対応について				
	施設名	郵便番号	住所（都道府県から記載）	電話番号	ウェブサイトURL	初診の電話等を用いた診療の実施の有無	再診の電話等を用いた診療の実施の有無	対応診療科	担当医師名	対面診療が必要と判断した場合に連携する医療機関名（複数ある場合は複数、住所も併せて記載）
505	茗荷谷なかむらクリニック	112-0006	東京都文京区小日向4-5-16　ツインヒルズ茗荷谷1階	03-3945-6161	http://myogadani-cl.jp/	○	○	内科	中村　陽一	
506	茗荷谷乳腺クリニック	112-0012	東京都文京区大塚1-5-18大伴ビル４階	03-5981-8288	https://breast-cl.com	○	○	乳腺外科	氷室　貴規	
507	茗荷谷レディースクリニック	112-0002	東京都文京区小石川5-3-4-3階	03-3830-0495	https://myogadani-ladies.com/		○	産婦人科	河合有希	
508	みらいメディカルクリニック茗荷谷	112-0012	東京都文京区大塚1-4-15-202	03-3943-0123	http://www.mirai-mc.com	○	○	内科<br>小児科	濱口　玲央	
509	三輪医院	112-0011	東京都文京区千石4-26-8	03-5976-9933	https://miwaclinic.jimdofree.com/	○	○	内科	三輪一彦	
510	むらい内科・循環器クリニック	113-0022	東京都文京区千駄木３－４３－３ATK千駄木ビル1階	03-3822-8010	http://murai-clinic.com/	○	○	内科<br>循環内科	村井　綱児	
511	めぐみクリニック	112-0012	東京都文京区大塚3-1-6-3A	03-5981-8990	http://megumi-cl.com		○	精神科	皆川　恵子	
512	森こどもクリニック	112-0002	東京都文京区5-40-21	03-5803-7887		○	○	小児科<br>アレルギー科	森　蘭子	
513	八千代診療所	113-0001	東京都文京区白山1-5-8	03-3811-4519		○	○	内科・精神科・小児科	井上博和	

電話や情報通信機器を用いて診療を実施する医療機関の一覧（東京都）

	基本情報					事務連絡に基づく対応について				
	施設名	郵便番号	住所（都道府県から記載）	電話番号	ウェブサイトURL	初診の電話等を用いた診療の実施の有無	再診の電話等を用いた診療の実施の有無	対応診療科	担当医師名	対面診療が必要と判断した場合に連携する医療機関名（複数ある場合は複数、住所も併せて記載）
514	ゆき医院	106-0031	東京都文京区本郷5-26-2　東大赤門前ビル3階	03-5840-8660	http://www.yuki-mcl.jp/	○	○	内科	大田由己子	東京大学附属病院（東京都文京区本郷7-3-1）<br>駒込病院(東京都文京区本駒込3-18-22)<br>日本医科大学（東京都文京区千駄木1-1-5）
515	ようようインターナショナルクリニック	112-0003	東京都文京区春日2-11-8-304	03-3946-5886<br>090-8375-2601	https://yyic.jp/index.html	○	○	内科	楊　陽<br>陳　莉薇	1.　聖路加国際病院（東京都中央区明石町9-1）<br>2.　医療法人医新会　医新クリニック（東京都板橋区大山町23-1）<br>3.　医療法人菅沼会　腎内科クリニック世田谷（東京都世田谷区南烏山4-21-14）<br>4.　医療法人瑛会　東京ネクスト内科・透析クリニック（東京都荒川区西日暮里2丁目22-1）<br>5　横浜市立大学附属 市民総合医療センター（横浜市南 [illegible]
516	良田眼科	211-0002	東京都文京区小石川4-21-2　ハイツ小石川２F	03-3816-1300	yoshida-ganka-bunkyoku.com	○	○	眼科	良田　夕里子	
517	りくぎえん皮膚科	113-0021	東京都文京区本駒込6-2-1　栄泉堂ビル301	03-6912-0812	http://rikugien-hifuka.com/		○	皮膚科	二神　綾子	日本医科大学付属病院
518	わたなべ皮膚科	130-0001	東京都文京区白山1-33-16　パークコートプレイスビル４F	03-3830-8080	watanabe-skin.jp	○	○	皮膚科	渡邉荘子	
519	谷口医院	113-0021	東京都文京区本駒込2-8-11	03-5395-7760	http://www.taniguchi-iin.com/	○	○	内科	谷口　善郎	
520	浅草胃腸肛門クリニック	111-0033	東京都台東区花川戸1-2-4　7F	03-3845-7800	https://asakusaichoukoumon.com/		○	大腸・肛門外科、胃腸内科	佐藤兼俊、原聖佳　他	
521	あさくさ橋心臓と血管のクリニック	111-0053	東京都台東区浅草橋1-18-9 山上ビル2階	03-6240-9286	https://acvc.jp/	○	○	心臓内科<br>血管内科<br>血管外科	髙橋保裕	
522	いりやキッズクリニック	110-0004	東京都台東区下谷3-11-12	03-5808-0415	http://iriyakids.com/		○	小児科<br>アレルギー科	浅野　由美子	

電話や情報通信機器を用いて診療を実施する医療機関の一覧（東京都）

	基本情報					事務連絡に基づく対応について				
	施設名	郵便番号	住所（都道府県から記載）	電話番号	ウェブサイトURL	初診の電話等を用いた診療の実施の有無	再診の電話等を用いた診療の実施の有無	対応診療科	担当医師名	対面診療が必要と判断した場合に連携する医療機関名（複数ある場合は複数、住所も併せて記載）
523	医療法人社団一順会田村胃腸科外科	110-0012	東京都台東区竜泉３－４２－１１	03-3874-9981		○	○	内科	田村順二	
524	医療法人社団永明会　一宮メンタルクリニック	110-0005	東京都台東区上野6-1-1小西本店ビル4F	03-5817-4824	https://i-mc.jp	○	○	精神科<br>心療内科	各主治医	
525	医療法人社団尾泉会<br>つちやファミリークリニック	110-0013	東京都台東区入谷2-25-2-1F	03-3873-1375	http://tsuchiya-family-clinic.com/	○	○	内科<br>小児科<br>皮膚科	土屋　晶義<br>土屋　佳奈	永寿総合病院（東京都台東区東上野2-23-16）<br>台東区立台東病院（東京都台東区千束3-20-5）<br>浅草寺病院（東京都台東区浅草2-30-17）
526	医療法人社団菊和会<br>谷中クリニック	110-0001	東京都台東区谷中3-13-10	03-3823-1261		○	○	内科<br>小児科<br>外科・肛門科	竹入　正彦	
527	医療法人社団輝生会<br>たいとう診療所	111-0041	東京都台東区元浅草1-16-17	03-5828-8051	http://www.motoasakusa-reha.com	○	○	内科<br>神経内科<br>リハビリテーション科	斉木　三鈴<br>吉村　菜穂子<br>菊地　サエ子<br>中島　勇二	左院
528	医療法人社団道草会浜田クリニック	110-0015	東京都台東区東上野３－３６－１	03-3832-5426			○	精神科	梶原　徹	
529	医療法人社団ルーチェ会<br>佐々木医院	111-0032	東京都台東区浅草5-33-12	03-3872-6776	http://www.sasakiiin-luce.jjp	○	○	婦人科<br>内科	佐々木　貴子	東京慈恵医科大学柏病院（柏市柏下163-1）
530	上野睡眠クリニック	110-0005	東京都台東区上野７－６－５－９Ｆ	03-5830-5951	http://ueno5951.com		○	内科（睡眠時無呼吸症候群）	古賀　達夫	
531	浦尾医院	110-0003	東京都台東区根岸5-17-6	03-3875-0242	https://www.uraoiin-ioukai.com/	○	○	耳鼻咽喉科	浦尾弥須子<br>浦尾正彦<br>若林健一郎	

電話や情報通信機器を用いて診療を実施する医療機関の一覧（東京都）

	基本情報					事務連絡に基づく対応について				
	施設名	郵便番号	住所（都道府県から記載）	電話番号	ウェブサイトURL	初診の電話等を用いた診療の実施の有無	再診の電話等を用いた診療の実施の有無	対応診療科	担当医師名	対面診療が必要と判断した場合に連携する医療機関名（複数ある場合は複数、住所も併せて記載）
532	永寿総合病院　柳橋分院	111-0052	東京都台東区柳橋2-20-4	03-3851-9375	http://www.yanagibashihp.com	○	○	内科は初再診<br>整形外科は再診のみ	内科初診は米丸、初再診は米丸、和田、藤井/整形外科は全医師	
533	小川眼科診療所	110-0005	東京都台東区上野2-11-6	03-3831-0588	www.ogawa-ganka.jp	○	○	眼科<br>精神科<br>皮膚科	加藤美名子<br>板村論子	
534	北島クリニック	110-0005	東京都台東区上野1-16-16　第２三倉ビル３F	03-5807-6160	http://www.kitajima-cl.com		○	精神科<br>心療内科	北島潤一郎	
535	社会福祉法人　浅草寺病院	111-0032	東京都台東区浅草2丁目30番17号	03-3841-3330	https://www.sensoji-h.or.jp/		○	内科<br>外科<br>整形外科<br>小児科<br>眼科<br>耳鼻咽喉科<br>泌尿器科	黒田　忠英<br>宮木　陽<br>増田　陽子<br>田村　英一郎<br>眼科担当医<br>耳鼻咽喉科担当医<br>泌尿器科担当医	
536	ストレスケア東京上野駅前クリニック	110-0005	東京都台東区上野7-7-7 早稲田ビルヂング6階	03-3842-7730	https://tokyoueno.com/		○	心療内科<br>精神科	細川　大雅<br>緒方　優	東京大学医学部附属病院（東京都文京区本郷7-3-1）
537	ぞうさん内科クリニック	110-0015	東京都台東区東上野2丁目22-5旭ビル1階101号室	03-3837-7775	https://www.zousannaika.com	○	○	内科	森山　賢一	
538	たまおか眼科	110-0005	東京都台東区上野2-7-13-3F	03-5812-3270	tamaoka-eyeclinic.com		○	眼科	玉岡　容子	
539	東京トータルライフクリニック	111-0034	東京都台東区雷門2-6-3ユニカ雷門ビル2階	03-5806-9871	http://tlc.or.jp/	○	○	内科	穴水聡一郎　長屋直樹　藤純一郎　千葉宙門	
540	同善会クリニック	110-0011	東京都台東区三ノ輪2-12-12	03-3801-6180	http://www.dozen-hp.com		○	総合診療科	増田　浩三	

電話や情報通信機器を用いて診療を実施する医療機関の一覧（東京都）

	基本情報					事務連絡に基づく対応について				
	施設名	郵便番号	住所（都道府県から記載）	電話番号	ウェブサイトURL	初診の電話等を用いた診療の実施の有無	再診の電話等を用いた診療の実施の有無	対応診療科	担当医師名	対面診療が必要と判断した場合に連携する医療機関名（複数ある場合は複数、住所も併せて記載）
541	みうら眼科・皮フ科クリニック	110-0012	東京都台東区竜泉2-19-18 クリニックステーション三ノ輪1Ｆ	03-6458-1050	http://miuraclinic-taitoku.jp	○	○	眼科	三浦　克洋	
542	三倉クリニック	110-0005	東京都台東区上野1-16-16第２三倉ビル1F	03-5807-8171	http://www.mikura-clinic.net/	○	○	呼吸器内科<br>循環器内科<br>消化器内科	倉持晋久	三井記念病院（東京都千代田区神田和泉１番地）
543	吉岡整形外科クリニック	111-0053	東京都台東区浅草橋1-10-6 1階	03-5822-5563	http://www.yoshioka-seikeigeka.com		○	整形外科クリニック	吉岡 利孝	
544	吉野眼科クリニック	110-0005	東京都台東区上野1-20-10<br>風月堂本社ビル6階	03-3839-5092	https://www.yoshino-eye-clinic.com/	○	○	眼科	吉野　健一<br>中島　希和子	日本医科大学付属病院（文京区）<br>東京大学医学部附属病院（文京区）<br>慶応義塾大学病院（新宿区）<br>日本大学医学部附属板橋病院（板橋区）
545	医療法人社団ナイズ キャップスクリニック錦糸町	130-0022	東京都墨田区江東橋4-27-14　錦糸町パルコ7F	03-4579-2973	https://www.caps-clinic.jp/kinshicho	○	○	小児科	菅本　健司	
546	吾嬬医院	131-0043	東京都墨田区立花5-26-8	03-3612-3475	azuma-iin.webmedipr.jp		○	内科	揚　志成	
547	医療法人社団草思会<br>クボタクリニック	130-0003	東京都墨田区横川３－２－４	03-3623-2011	https://www.kubocli.jp/yokokawa/		○	精神科<br>心療内科	安達洋一<br>その他主治医	
548	医療法人社団草思会<br>錦糸町クボタクリニック	130-0013	東京都墨田区錦糸３－５－１	03-3623-3031	https://www.kubocli.jp/kinshichou/		○	精神科<br>心療内科	窪田彰<br>その他主治医	
549	医療法人社団中央白報会<br>総合クリニック<br>ドクターランド錦糸町	130-0012	東京都墨田区太平4-1-2　オリナス・モール４F	03-5809-7389	http://dr-land-kinshicho.com/		○	内科	二見　良平	

電話や情報通信機器を用いて診療を実施する医療機関の一覧（東京都）

	基本情報					事務連絡に基づく対応について				
	施設名	郵便番号	住所（都道府県から記載）	電話番号	ウェブサイトURL	初診の電話等を用いた診療の実施の有無	再診の電話等を用いた診療の実施の有無	対応診療科	担当医師名	対面診療が必要と判断した場合に連携する医療機関名（複数ある場合は複数、住所も併せて記載）
550	医療法人社団　立圭会平野医院	131-0043	東京都墨田区立花6-1-14	090-3084-3441	https://rickay.com		○	内科小児科	平野　圭	
551	押上駅前　松浦内科クリニック	131-0045	東京都墨田区押上１－１１－５　菅野ビル１・２階	03-5637-9388	http://www.matsuura-medical-clinic.org/	○	○	内科、呼吸器内科、内分泌代謝内科、循環器内科、消化器内科、耳鼻咽喉科	松浦　宗行<br>鈴木　慎太郎<br>三國　肇子<br>渡部　ちづる<br>近藤　誠太<br>後藤　唯子<br>森　智明<br>藤居　直和	昭和大学江東豊洲病院（東京都江東区）<br>東京曳舟病院（東京都墨田区）<br>あそか病院（東京都江東区）
552	おしあげ耳鼻咽喉科	130-0003	東京都墨田区横川3-11-14　マツマルビル2F	03-3622-2633	http://oshiage.sho-jin.com/	○	○	耳鼻科	山田　哲也	
553	梶原病院	131-0031	東京都墨田区墨田３－３１－１２	03-3614-2255	http://www.kajiwara-hospital.com		○	内科<br>整形	梶原　宗介	東京慈恵会医科大学葛飾医療センター（東京都葛飾区青戸６－４１－２）
554	唐澤医院	130-0023	東京都墨田区立川１－１２－１３	03-3631-2336	http://www.ne.jp/asahi/karasawa/clinic/index.html	○	○	小児科・内科	唐澤　賢祐	
555	木村医院	131-0031	東京都墨田区墨田５－３５－６	03-3618-5501	https://medical-clinic-6705.business.site/	○	○	内科<br>小児科	木村　揚	
556	錦糸町こどもクリニック	130-0013	東京都墨田区錦糸３－１４－９－１階	03-3625-7727	https://kinshicho-kodomo.com		○	小児科	西口　康介	
557	京成曳舟<br>耳鼻科皮フ科クリニック	131-0041	東京都墨田区八広1-15-2	03-6661-8140	https://keiseiclinic.jp/	○	○	耳鼻科<br>皮膚科	久保　伸夫<br>渡邊　恒輔	
558	湘南メディカル記念病院	130-0026	東京都墨田区両国二丁目21番1号	0120-979-097	https://www.sbc-hospital.jp/	○	○	内科	加藤　貴志<br>春山　晋	

電話や情報通信機器を用いて診療を実施する医療機関の一覧（東京都）

	基本情報					事務連絡に基づく対応について				
	施設名	郵便番号	住所（都道府県から記載）	電話番号	ウェブサイトURL	初診の電話等を用いた診療の実施の有無	再診の電話等を用いた診療の実施の有無	対応診療科	担当医師名	対面診療が必要と判断した場合に連携する医療機関名（複数ある場合は複数、住所も併せて記載）
559	隅田川診療所	131-0033	東京都墨田区向島1-24-6	03-3626-5100		○	○	内科　小児科<br>皮膚科	山室　学	
560	積善会医院	131-0032	東京都墨田区東向島６－６－１３	03-3614-3629		○	○	内科、整形外科	吉村　宏<br>木場　ともえ	
561	立花クリニック	131-0043	東京都墨田区立花1-23-3-101	03-3616-8216	https://www.tachibana-cl.or.jp		○	内科	島田　憲明	
562	東京シティクリニック両国	130-0021	東京都墨田区緑1ー14ー4両国TYビル9階	03-5638-3377	https://tokyocityclinic-ryogoku.com/	○	○	内科、乳腺外科	吉井	東京都立墨東病院（〒130-8575　東京都墨田区江東橋4-23-15）<br>杏雲堂病院（〒101-0062　東京都千代田区神田駿河台1-8）
563	とうきょうスカイツリー駅前内科	131-0033	東京都墨田区向島3-33-13　リョービビル４０１号室	03-5809-7660	https://skytree-clinic.jp/	○	○	内科	金子俊之<br>河野晋也	順天堂大学医学部付属順天堂医院（東京都文京区本郷3-1-3）
564	東京都リハビリテーション病院	131-0034	東京都墨田区堤通2-14-1	03-3616-8600	http://www.tokyo-reha.jp/		○	リハビリテーション科<br>整形外科<br>泌尿器科	各医師	
565	東京曳舟病院	131-0032	東京都墨田区東向島2-27-1	03-5655-1120	https://tokyo-hikifune-hp.jp/		○	総合診療科	山本保博	
566	同愛記念病院	130-8587	東京都墨田区横網2-1-11	03-3625-6381	http://www.doai.jp/		○	全科		
567	西大島あたらし眼科	136-0072	東京都江東区大島3-14-17江口ビル２F	03-5858-8861	https://www.atarashi-ganka.com/		○	眼科	新　卓也	江東病院（東京都江東区大島６丁目８−５）<br>墨東病院（東京都墨田区江東橋４丁目２３−１５）

電話や情報通信機器を用いて診療を実施する医療機関の一覧（東京都）

	基本情報					事務連絡に基づく対応について				
	施設名	郵便番号	住所（都道府県から記載）	電話番号	ウェブサイトURL	初診の電話等を用いた診療の実施の有無	再診の電話等を用いた診療の実施の有無	対応診療科	担当医師名	対面診療が必要と判断した場合に連携する医療機関名（複数ある場合は複数、住所も併せて記載）
568	日伸駅前クリニック	130-0022	東京都墨田区江東橋3-10-8錦糸町スクエアビル5階	03-3634-9033	https://hikifune-kids.com		○	内科<br>人工透析	森山　君子	森山記念病院（東京都江戸川区北葛西4-3-1）
569	ひきふねこどもクリニック	131-0046	東京都墨田区京島1-39-1　曳舟西ビューハイツ１F店舗122	03-3618-1111	https://hikifune-kids.com		○	小児科	髙田　昌亮	
570	増田耳鼻咽喉科	131-0032	東京都墨田区東向島6-6-2	03-3611-8733	http://www.masuda-jibika.jp/		○	耳鼻咽喉科	増田　正純<br>阿部　実恵子	
571	宮田内科クリニック	130-0011	東京都墨田区石原3-19-1 早川ビル1階	03-6666-5266	http://www.miyata-naika.jp/		○	内科<br>消化器内科	宮田達也	
572	八広同仁会クリニック	131-0041	東京都墨田区八広6-29-14	03-3619-4471	http://yahirodouzinkai-clinic.com/	○	○	内科小児科泌尿器科	佐々木　美和	同愛記念病院（東京都墨田区横綱2-1-11）
573	両国医院	130-0014	東京都墨田区亀沢2－11－14	03-3624-3366			○	内科<br>小児科	伊能高明・<br>伊能容子	
574	両国駅前内科・透析クリニック	130-0026	東京都墨田区両国2-21-8両国駅前ビル4階	050-5865-5610	https://www.ryogoku-dialysis.jp/	○	○	内科	木村庄吾	
575	両国東口クリニック		東京都墨田区両国3-21-1	130-0026	http://www.higasiguti.jp/		○	リウマチ科<br>内科	大山　博司<br>諸見里　仁<br>藤森　新	
576	ガーデンプラザ石井クリニック	131-0034	東京都墨田区堤通1-19-11	03-3616-1413			○	内科	石井　達男	

電話や情報通信機器を用いて診療を実施する医療機関の一覧（東京都）

	基本情報					事務連絡に基づく対応について				
	施設名	郵便番号	住所（都道府県から記載）	電話番号	ウェブサイトURL	初診の電話等を用いた診療の実施の有無	再診の電話等を用いた診療の実施の有無	対応診療科	担当医師名	対面診療が必要と判断した場合に連携する医療機関名（複数ある場合は複数、住所も併せて記載）
577	赤羽根医院	136-0074	東京都江東区東砂2-1-15	03-3648-3622	https://www.akahaneclinic.com/	○	○	内科<br>小児科	赤羽根　巌	江東病院（東京都江東区大島6-8-5）
578	有明こどもクリニック有明院	135-0063	東京都江東区有明1-4-11-1階	03-3599-0415	https://child-clinic.or.jp	○	○	小児科　内科	小暮　裕之	
579	有明こどもクリニック豊洲院	135-0061	東京都江東区豊洲5-5-25-1F	03-6204-2733	https://clinicclinic.or.jp	○	○	内科小児科アレルギー科	村上典子	昭和大学江東豊洲病院 聖路加国際病院
580	いよりこどもクリニック	135-0062	東京都江東区東雲１-9-10　　イオン東雲ショッピングセンター２階	03-6221-3448	http://www.shujii.com/0362213448/		○	小児科	伊従秀章	
581	医療法人社団大島小児科医院	136-0072	東京都江東区大島8-5-2N&Hビル１，２階	03-5609-7631	http://www.oojimahospital.com/	○	○	小児科、アレルギー科	成高信一	江東病院（東京都江東区大島6-8-5)都立墨東病院
582	医療法人社団　共咲会サワイメディカルクリニック	136-0073	東京都江東区北砂6-27-17	03-3615-0606	http://sawaimedicalclinic.net		○	内科	金田　竜真	
583	医療法人社団弘大会　五の橋こどもクリニック	136-0071	東京都江東区亀戸6-14-3 1F	03-5858-8011	http://www.gonohashi-kodomo.com	○	○	小児科<br>内科	大塚　正弘	東京都立墨東病院（東京都墨田区江東橋4－23－15）
584	医療法人社団祥宏会鈴木医院	136-0076	東京都江東区南砂1-9-9	03-3644-0749	http://www004.upp.so-net.ne.jp/suzuki-clinic/	○	○	内科<br>小児科	鈴木良一	
585	医療法人社団高裕会深川立川病院	135-0011	東京都江東区扇橋2-2-3	03-3645-2101	http://www.tachikawa-hospital.jp	○	○	内科	立川　裕理	

電話や情報通信機器を用いて診療を実施する医療機関の一覧（東京都）

	基本情報					事務連絡に基づく対応について				
	施設名	郵便番号	住所（都道府県から記載）	電話番号	ウェブサイトURL	初診の電話等を用いた診療の実施の有無	再診の電話等を用いた診療の実施の有無	対応診療科	担当医師名	対面診療が必要と判断した場合に連携する医療機関名（複数ある場合は複数、住所も併せて記載）
586	医療法人社団浩成会　なかむら整形外科	135-0005	東京都江東区高橋14-3盛市ビル４階	03-3631-8833	https://www.nakamura-seikeigeka.or.jp/	○	○	整形外科	中村　浩	
587	医療法人社団TIK 大手町さくらクリニックin豊洲	135-0061	東京都江東区豊洲3-2-20　豊洲フロント2F	03-6219-5688	http://oscl.jp/	○	○	内科	西山　寿子	
588	医療法人社団桃医会小野内科診療所	136-0072	東京都江東区大島1－33－15　小野ビル１階	03-3636-5505	https://www.ononaika-cardiogp.com/	○	○	内科、循環器内科	小野　卓哉	
589	医療法人社団<br>ひらの亀戸ひまわり診療所	136-0071	東京都江東区亀戸7-10-1<br>Zビル2F	03-5609-1823	https://www.himawari-clinic.jp/	○	○	内科<br>小児科	毛利　一平	
590	医療法人社団藤崎病院	136-0076	東京都江東区南砂１－２５－１１	03-3648-2111	http://www.fujisaki-hp.com		○	外科<br>内科<br>脳神経外科<br>整形外科<br>泌尿器科	全ての医師	
591	医療法人社団　平生会<br>神原医院	136-0073	東京都江東区北砂7-3-17	03-3645-7351			○	内科<br>小児科	神原　礼文<br>成瀬　勝俊	医療法人社団寿康会<br>寿康会病院
592	永代クリニック	135-0034	東京都江東区永代2-37-22永代クリニックビル1F	03-3641-3055	http://eitai-clinic.com	○	○	内科	金　民日	
593	大島駅前クリニック	136-0072	東京都江東区大島5-10-10-セントラルプラザ大島１階	03-5626-3777	clinic@ojima-ekimae.jp	○	○	内科<br>胃腸内科	近藤　健司	
594	大島耳鼻咽喉科・アレルギー科	136-0072	東京都江東区大島5-10-10　セントラルプラザ大島4F	03-5626-1133	http://www.ojima-ent.com		○	耳鼻咽喉科・アレルギー科	寺田修久　植草康浩	昭和大学江東豊洲病院

電話や情報通信機器を用いて診療を実施する医療機関の一覧（東京都）

	基本情報					事務連絡に基づく対応について				
	施設名	郵便番号	住所（都道府県から記載）	電話番号	ウェブサイトURL	初診の電話等を用いた診療の実施の有無	再診の電話等を用いた診療の実施の有無	対応診療科	担当医師名	対面診療が必要と判断した場合に連携する医療機関名（複数ある場合は複数、住所も併せて記載）
595	医療法人社団<br>おかの小児科	135-0045	東京都江東区古石場2－14－1ウェルタワー深川・医療モール	03-5621-2336	http://home.catv.ne.jp/pp/oknclnc/top.html	○	○	小児科	岡野　周子	
596	オビ内科クリニック	135-0052	東京都江東区潮見2-1-10	03-5665-6770	http://obi-clinic.com		○	内科<br>小児科	小尾　直美	
597	笠井小児クリニック	136-0072	東京都江東区大島9－5－1－103	03-3636-2577		○	○	内科<br>小児科	笠井　秀明	江東病院（東京都江東区大島6－8－5）
598	かしわぎクリニック	135-0047	東京都江東区東雲１－９－２１－１０２	03-3532-6600	https://www.kashiwagi-clinic.jp		○	内科 皮膚科	柏木 明	
599	亀戸キッズクリニック	136-0071	東京都江東区亀戸2-42-5　2F	03-5875-3387	http://www.kameido-kids.com/	○	○	小児科	杉本　佳乃	
600	亀戸水神森クリニック	136-0071	東京都江東区亀戸6－57－20　亀戸東口駅前ビル2階	03-5658-6767	http://www.suijinmori.com	○	○	内科	金光　裕幸	
601	亀戸中央通りクリニック	136-0071	東京都江東区亀戸5-20-23ﾎｰﾌﾟﾊｾｶﾞﾜｰ階	03-5628-6531		○	○	内科	王　国定<br>神戸　正樹	
602	亀戸内科クリニック	136-0071	東京都江東区亀戸2-26-8-風月堂ビル1F	03-5626-5600	http://kameido-naika.com/	○	○	内科	荒木　正	
603	北原診療所	136-0073	東京都江東区北砂5-16-1	03-5632-1991	http://www.ne.jp/asahi/kitahara/clinic/	○	○	内科<br>小児科<br>皮膚科	松元明子	江東病院（東京都江東区大島6-8-5）<br>墨東病院（東京都墨田区江東橋4-23-15）

電話や情報通信機器を用いて診療を実施する医療機関の一覧（東京都）

	基本情報					事務連絡に基づく対応について				
	施設名	郵便番号	住所（都道府県から記載）	電話番号	ウェブサイトURL	初診の電話等を用いた診療の実施の有無	再診の電話等を用いた診療の実施の有無	対応診療科	担当医師名	対面診療が必要と判断した場合に連携する医療機関名（複数ある場合は複数、住所も併せて記載）
604	清澄白河こどもクリニック	135-0022	東京都江東区三好3-1-3	03-5602-9119	http://www.shujii.com/56029119/i/	○	○	小児科	名越　廉<br>名越　涼子	
605	医療法人社団駿圭美会水谷皮フ科クリニック	163-0072	東京都江東区大島1-1-5　VIP大島2階	03-5628-2770	https://mizutani-hifuka.com/	○	○	皮膚科	水谷　治子	
606	協和メディカルクリニック	136-0073	東京都江東区北砂2-15-40	03-3648-4131	https://www.aiiku-med.or.jp/kyowa.html		○	内科	細野 紫麻子	
607	公益財団法人がん研究会有明病院	135-8550	東京都江東区有明3-8-31	03-3520-0111	https://www.jfcr.or.jp/hospital/		○	呼吸器内科<br>消化器内科<br>乳腺内科<br>血液内科<br>腫瘍内科<br>漢方内科<br>精神科<br>皮膚科	各診療科担当医師	
608	小林クリニック	136-0072	東京都江東区大島4-1-6-105	03-3684-0481			○	内科	小林功<br>小林昭夫	
609	小林内科クリニック	135-0011	東京都江東区扇橋2-17-5	03-3699-5884			○	内科<br>呼吸器科<br>循環器科<br>ｱﾚﾙｷﾞｰ科	小林　健嗣	
610	澤井クリニック	135-0061	東京都江東区豊洲5－2－10沢真ビル3F	03-5548-2629	http://www.sawai-cl.com	○	○	内科<br>皮膚科	澤井　まゆみ<br>澤井　真次郎	
611	社会医療法人社団順江会江東病院	136-0072	東京都江東区大島	03-3685-2166	http://www.koto-hospital.or.jp/index.html		○	全科	各担当医	
612	社会福祉法人<br>あそか会あそか病院	135-0002	東京都江東区住吉1-18-1	03-3632-0290	https://hp.asokakai.or.jp/		○	内科<br>耳鼻科	田崎　俊之<br>古川　傑	

電話や情報通信機器を用いて診療を実施する医療機関の一覧（東京都）

	基本情報					事務連絡に基づく対応について				
	施設名	郵便番号	住所（都道府県から記載）	電話番号	ウェブサイトURL	初診の電話等を用いた診療の実施の有無	再診の電話等を用いた診療の実施の有無	対応診療科	担当医師名	対面診療が必要と判断した場合に連携する医療機関名（複数ある場合は複数、住所も併せて記載）
613	寿康会病院	136-0073	東京都江東区北砂2-1-22	03-3645-9151	https://www.jukoukai.or.jp/		○	リハビリ・整形<br>内科<br>小児科<br>消化器外科<br>その他	猪口　雄二<br>井野　威<br>宮本　新次郎<br>山崎　弘貴<br>石津　和洋<br>外来診療担当医	
614	しらかわ耳鼻咽喉科クリニック	135-0021	東京都江東区白河3-4-3-202	03-5245-3387	https://www.shirakawa-ent.com/		○	耳鼻咽喉科	姫野千惠美	順天堂大学附属順天堂医院（文京区本郷3-1-3）
615	砂町銀座はた耳鼻咽喉科	136-0072	東京都江東区北砂5-7-2	03-5634-3387	http://sunamachiginza-ent.com	○	○	耳鼻咽喉科	畠将晃	
616	清湘会記念病院	136-0071	東京都江東区亀戸2-17-24	03-3636-2301	https://www.seishokai.or.jp/		○	内科	氏家　一知	
617	たかすな内科･胃腸内科クリニック	135-0062	東京都江東区東雲1-9-22	03-3536-3004	http://www.takasuna-naika.com/?utm_source=hospita.jp&utm_medium=referral		○	内科<br>胃腸内科	高砂　憲一	
618	たけうちこどもクリニック	135-0061	東京都江東区豊洲4-9-13-132	03-3533-2415	http://www.takeuchi-kc.com		○	小児科	竹内敏雄	
619	タムスファミリークリニック豊洲	135-0061	東京都江東区豊洲2-2-1 アーバンドック ららぽーと豊洲3 4階（豊洲ベイサイドクロスタワーC棟内）	03-3520-8813	https://familyclinic-toyosu.tums.jp/		○	内科　小児科	佐々木　隼人	
620	東京有明医療大学附属クリニック	135-0063	東京都江東区有明2-9-1	03-6703-7003			○	内科<br>整形外科<br>外科	林　洋<br>田中　滋城<br>川嶋　朗<br>鈴木　秀一<br>福林　徹<br>高橋　雅足	
621	東京都立東部療育センター	136-0075	東京都江東区新砂3-3-25	03-5632-8070	https://www.tobu-ryoiku.jp/		○	小児科<br>神経小児科<br>神経内科	岩崎　裕治<br>外　常勤医師	

電話や情報通信機器を用いて診療を実施する医療機関の一覧（東京都）

	基本情報					事務連絡に基づく対応について				
	施設名	郵便番号	住所（都道府県から記載）	電話番号	ウェブサイトURL	初診の電話等を用いた診療の実施の有無	再診の電話等を用いた診療の実施の有無	対応診療科	担当医師名	対面診療が必要と判断した場合に連携する医療機関名（複数ある場合は複数、住所も併せて記載）
622	東陽町駅前クリニック	135-0016	東京都江東区東陽3-27-17 長谷川ビル3階	03-5665-2381	https://toyocho.towakai.com/		○	内科	中田　健一	
623	東陽町すばるクリニック	135-0016	東京都江東区東陽3-27-21 信栄実業本社ビル2階	03-5653-1587	https://toyocho-subaru.towakai.com/		○	耳鼻咽喉科	三橋　正継	
624	豊洲小児科醫院	135-0061	東京都江東区豊洲5-6-29　1F	03-3533-7733	http://www.toyosu-shounika.com	○	○	小児科	染谷　大槻	昭和大学江東豊洲病院（東京都江東区豊洲5ー1ー38）<br>順天堂大学医学部附属順天堂医院（東京都文京区本郷3ー1ー3）<br>東京臨海病院（東京都江戸川区臨海町1ー4ー2）<br>聖路加国際病院（東京都中央区明石町9ー1）
625	とよす内科クリニック	135-0061	東京都江東区豊洲4-2-2豊南堂ビル2階	03-5144-5155	http://toyonai.jp/	○	○	内科・消化器内科・内視鏡内科・肝臓内科	金澤　信彦	
626	豊洲ベイシティクリニック<br>耳鼻咽喉科	135-0061	東京都江東区豊洲2-2-1<br>豊洲ベイサイドクロスタワーC棟4階	03-3534-3387	http://toyosu.sho-jin.com/	○	○	耳鼻科	高野　真吾	
627	豊村医院耳鼻咽喉科	136-0071	東京都江東区亀戸2-28-16	03-5627-8555	https://www.toyomurajibika.com/		○	耳鼻咽喉科	豊村　文将	
628	豊村医院耳鼻咽喉科音声・聴覚メディカルケア	136-0071	東京都江東区亀戸5-15-1　いなきやビル3階	03-5858-8604	https://www.toyomurajibika.com/		○	耳鼻咽喉科	髙瀬　聡一郎	
629	なおやこどもクリニック	136-0074	東京都江東区東砂7-19-13ベルコモン南砂２０１	03-5653-0708	https://www.naokodo.com/	○	○	小児科<br>アレルギー科	坂口直哉	
630	中澤医院	136-0076	東京都江東区南砂７－４－４	03-3644-7670			○	内科<br>小児科<br>循環器内科	新保　悟朗	

電話や情報通信機器を用いて診療を実施する医療機関の一覧（東京都）

	基本情報					事務連絡に基づく対応について				
	施設名	郵便番号	住所（都道府県から記載）	電話番号	ウェブサイトURL	初診の電話等を用いた診療の実施の有無	再診の電話等を用いた診療の実施の有無	対応診療科	担当医師名	対面診療が必要と判断した場合に連携する医療機関名（複数ある場合は複数、住所も併せて記載）
631	ぴやじま内科医院	136-0072	東京都江東区大島5-36-7-2F	03-5628-0571	http://www.clinic-ojima.com	○	○	内科<br>小児科<br>皮膚科<br>泌尿器科	鎌形　博展	
632	平和記念医院	135-0023	東京都江東区平野2-11-5 パシフィック第二門前仲町2F	03-3820-8880	https://heiwa-med.com	○	○	内科<br>皮膚科<br>漢方内科	劉　瑞芹	江東病院　（東京都江東区大島6-8-5)<br>あそか病院　（東京都江東区住吉1-18-1)
633	藤川内科・呼吸器内科クリニック	136-0072	東京都江東区大島4-6-21-西大島ビューハイツ101号室	03-5627-7700	https://www.fujikawa-naika.com		○	内科	藤川　貴浩	
634	MIZENクリニック豊洲	135-0061	東京都江東区豊洲3-2-20 豊洲フロント１階	03-6225-0230	https://www.mizenclinic.jp/	○	○	内科<br>心療内科<br>精神科	田澤 雄基<br>野原 弘義<br>竹林 駿太<br>久志本 公平	昭和大学高等豊洲病院（東京都江東区豊洲５丁目１−３８）
635	正井診療所	135-0012	東京都江東区海辺12-11	03-3644-0943	http://www.masai-s.com/	○	○	内科	正井　博文	
636	みつはたペインクリニック	135-0016	東京都江東区東陽2-4-26　飯田ビル3F	03-5683-1199	https://mituhata-pain.com/		○	ペインクリニック	光畑　裕正	順天堂大学医学部付属順天堂東京江東高齢者医療センター（東京都江東区新砂3-3-20）
637	南砂町駅前<br>おおさわクリニック	136-0075	東京都江東区新砂3-3-53<br>アルカナール南砂2階E	03-6458-7498	https://www.osawaclinic.net/		○	内科<br>胃腸肛門内科<br>外科	大澤　俊也	
638	南砂町おだやかクリニック	136-0075	東京都江東区新砂3-4-31　スナモ4F	03-5633-8751	http://www.junreikai.com	○	○	内科<br>循環器内科<br>呼吸器科	西澤　寛人<br>井上　宏一<br>林　光恵	
639	メンタルオフィス亀戸	136-0071	東京都江東区亀戸6-58-11　亀戸ESビル3F	03-3636-2377	http://www.kameido-mental.com/index.html		○	精神科	佐藤　光雅	

電話や情報通信機器を用いて診療を実施する医療機関の一覧（東京都）

	基本情報					事務連絡に基づく対応について				
	施設名	郵便番号	住所（都道府県から記載）	電話番号	ウェブサイトURL	初診の電話等を用いた診療の実施の有無	再診の電話等を用いた診療の実施の有無	対応診療科	担当医師名	対面診療が必要と判断した場合に連携する医療機関名（複数ある場合は複数、住所も併せて記載）
640	望月内科クリニック	135-0005	東京都江東区高橋13ー2　ヴィラロイヤル森下1F	03-5669-1531	http://www.mochizukinaika.com	○	○	内科<br>呼吸器科<br>循環器科<br>アレルギー科	望月　俊男	
641	森井整形外科	135-0013	東京都江東区千田13-13	03-3649-7000	https://morii-orth.jp/		○	整形外科	森井　太郎	
642	森崎医院	135-0016	東京都江東区東陽4-10-2-1F	03-3645-1493	https://morisaki-iin.com/		○	内科	佐々木　佑	
643	森下駅前クリニック	135-00004	東京都江東区森下1-16-7　太田ビル1階	03-6666-9335	https://www.morishitaekimae.com/		○	内科<br>呼吸器内科<br>小児科	篠塚　成順<br>神坂　京子	
644	もんなか整形外科	135-0044	東京都江東区越中島2-14-10	03-3630-3001	https://monnaka.jp/	○	○	整形外科	佐藤 芳貞	
645	柳沢ファミリークリニック	136-0073	東京都江東区北砂5-14-3-101	03-6458-7818	https://www.yanagisawafc.com	○	○	内科<br>小児科<br>皮膚科	柳澤　明子	
646	六地蔵クリニック	136-0076	東京都江東区南砂2-28-7	03-5690-7651	http://www.rokujizou-clinic.jp		○	脳神経外科<br>整形外科<br>神経内科<br>循環器内科<br>リハビリテーション科	小倉　弘章	
647	医療法人社団同友会　深川クリニック	135-0022	東京都江東区三好2-15-10	03-3630-0003	http://www.do-yukai.com		○	内科	髙谷　純司<br>他当日の担当医	
648	YSこころのクリニック	135-0033	東京都江東区深川1-5-5 佐藤康行真我ビル6階	03-6458-8804	https://shingaclinic.com/	○	○	精神科	宮島　賢也	

電話や情報通信機器を用いて診療を実施する医療機関の一覧（東京都）

	基本情報					事務連絡に基づく対応について				
	施設名	郵便番号	住所（都道府県から記載）	電話番号	ウェブサイトURL	初診の電話等を用いた診療の実施の有無	再診の電話等を用いた診療の実施の有無	対応診療科	担当医師名	対面診療が必要と判断した場合に連携する医療機関名（複数ある場合は複数、住所も併せて記載）
649	豊洲パークシティ皮膚科	135-0061	東京都江東区豊洲2-5-3-102E	03-5547-1221	https://www.toyosu-hifuka.com/		○	皮膚科	萩原　正則	豊洲パークシティ皮膚科
650	あおよこ皮膚科クリニック	140-0004	東京都品川区南品川2-17-24フルカワビル2階	03-5479-6051	http://www.aoyoko-sc.jp/		○	皮膚科	田嶋　徹	
651	阿部病院	141-0022	東京都品川区東五反田1-6-8	03-3447-4777		○	○	内科<br>小児科	木島澄子	
652	いすゞ病院	140-0013	東京都品川区南大井6-21-10	03-3762-9127	http://isuzu-hospital.jp/		○	内科<br>整形外科<br>眼科	芦原毅<br>木村一博<br>保坂美生<br>堀静<br>藤井英<br>姫野良<br>森山沙帆	
653	稲波脊椎・関節病院	140-0002	東京都品川区東品川3-17-5	03-3450-1773	https://www.iwai.com/inanami-sekitsui/	○	○	整形外科	稲波　弘彦<br>髙野　裕一<br>内山　英司<br>湯澤　洋平<br>金子　剛士<br>瀬川　知秀<br>柳澤　和芳<br>小口　史彦	
654	医療法人社団小川医院	142-0053	東京都品川区中延6ー9ー18	337817806	https://www.nakanobu-ogawaiinn.com/	○	○	内科小児科リウマチ科	小川仁史	
655	医療法人社団かなめ会田口クリニック	141-0031	東京都品川区西五反田1-25-5	03-6417-4155	https://taguchi-clinic.jp/		○	内科	田口　和三	
656	医療法人社団恵芳会松脇クリニック品川	141-0001	東京都品川区北品川6-7-29ガーデンシティー品川御殿山1階	03-6721-6870	https://matsuwaki.com		○	耳鼻咽喉科<br>呼吸器内科	松脇由典<br>中島庸也<br>柳清<br>満山知恵子<br>足立哲也<br>中込一之<br>皆川俊介<br>渡邉直昭	
657	医療法人社団孝徳会孝仁クリニック	140-0011	東京都品川区東大井5-14-11-1F	03-3474-5566	https://koujin.or.jp		○	内科（予約による訪問診療）	高久　仁利	

電話や情報通信機器を用いて診療を実施する医療機関の一覧（東京都）

	基本情報					事務連絡に基づく対応について				
	施設名	郵便番号	住所（都道府県から記載）	電話番号	ウェブサイトURL	初診の電話等を用いた診療の実施の有無	再診の電話等を用いた診療の実施の有無	対応診療科	担当医師名	対面診療が必要と判断した場合に連携する医療機関名（複数ある場合は複数、住所も併せて記載）
658	医療法人社団CVIC<br>大井町心臓クリニック	140-0011	東京都品川区東大井5-2-3<br>かんべ土地K-15ビル（おおい元気館）3階	03-6712-9085	https://oimachiclinic.com/		○	循環器内科<br>内科	寺島　正浩<br>伊藤　順子	
659	医療法人社団浄真会<br>仲田クリニック	142-0052	東京都品川区東中延2-9-7	03-5749-5117	http://www.nakada-cl.jp		○	内科<br>泌尿器科<br>皮膚科	仲田浄治郎　仲田佳子	東京品川病院（東京都品川区）
660	医療法人社団千秋双葉会<br>東京アーバンクリニック五反田	141-0022	東京都品川区東五反田5-27-3　第二野村ビル5F	03-3441-6755	http://www.tokyourbanclinic-gotanda.com/	○	○	小児科<br>アレルギー科<br>内科<br>皮膚科	野村　さや香	都立広尾病院（東京都渋谷区）<br>NTT関東病院（東京都品川区）<br>昭和大学病院（東京都品川区）<br>愛育病院（東京都港区）<br>日本赤十字医療センター（東京都渋谷区）など
661	医療法人社団ナイズ<br>キャップスクリニック東品川	140-0002	東京都品川区東品川４丁目５番２２号　オアーゼ品川レジデンス１階	03-4579-9249	https://www.caps-clinic.jp/higashi-shinagawa	○	○	小児科	藤井　泰志	東京品川病院（東京都品川区東大井６丁目３－２２）<br>東京都立広尾病院（東京都渋谷区恵比寿2-34-10）<br>昭和大学病院（東京都品川区旗の台１丁目５－８）
662	医療法人社団 はしだ眼科クリニック	141-0021	東京都品川区上大崎2-16-9アトレ目黒1-4階	03-5791-5430	https://www.hashidaeyeclinic.com/	○	○	眼科	橋田　節子	NTT東日本関東病院（東京都品川区）<br>井上眼科病院（東京都千代田区）
663	医療法人社団柊会島津山眼科	141-0022	東京都品川区東五反田1-5-3	03-5798-2740	http://www.shimazuyama-eye.jp/		○	眼科	酒井達朗	
664	医療法人社団朋英会　舘医院	142-0062	東京都品川区小山2-12-10	03-3782-8088	https://www.tachi-iin.com/556.html		○	内科泌尿器科	舘　英実	
665	医療法人社団　まつばらクリニック	142-0051	東京都品川区平塚3-3-13	03-5751-8501	http://まつばらクリニック.com/		○	内科小児科	松原豊子	
666	医療法人社団明方会　松本クリニック	142-0053	東京都品川区中延4-5-10オーク中延1F	03-3784-9100	https://www.matsumotoclinic-nakanobu.com/	○	○	内科	松本明石、松本真弓、	

電話や情報通信機器を用いて診療を実施する医療機関の一覧（東京都）

	基本情報					事務連絡に基づく対応について				
	施設名	郵便番号	住所（都道府県から記載）	電話番号	ウェブサイトURL	初診の電話等を用いた診療の実施の有無	再診の電話等を用いた診療の実施の有無	対応診療科	担当医師名	対面診療が必要と判断した場合に連携する医療機関名（複数ある場合は複数、住所も併せて記載）
667	医療法人社団めぐみ会めぐみクリニック目黒	141-0021	東京都品川区上大崎2-12-2　ミズホビル2階	03-5422-9877	https://www.megumicl.com/	○	○	内科	小野　直美<br>加藤　正典<br>菊池　俊介<br>須藤　英訓<br>税所　俊光<br>田村　豊	
668	医療法人社団豊佳会青柳医院	142-0042	東京都品川区豊町1ー4ー15第一青柳ビル	03-3788-0801			○	内科<br>外科	青柳豊	
669	医療法人社団柊会大崎眼科クリニック	141-0032	東京都品川区大崎1-6-4新大崎勧業ビル２階	03-5436-4155	http://www.ohsaki-eye.jp/		○	眼科	清水えりか<br>武田義玄	
670	医療法人社団癒合会医道五十三次クリニック	140-0001	東京都品川区北品川1-22-17　ニックハイム北品川109	03-6433-3170	https://www.takanawa-clinic.com/	○	○	内科	陰山　康成<br>/長岡　美妃	
671	NTT東日本関東病院	141-8625	東京都品川区東五反田5-9-22	03-3448-6111	https://www.nmct.ntt-east.co.jp/		○	小児科<br>産婦人科以外の全診療科	外来予定表に準じる	
672	えばらサンクリニック	142-0053	東京都品川区中延2-9-22-1F	03-3786-0012	http://ebara-sun-cl.net/		○	内科<br>小児科<br>皮膚科	北川　浩史	昭和大学病院、NTT東日本関東病院、東京品川病院、荏原病院、など
673	大井町耳鼻咽喉科	140-0011	東京都品川区東大井6-1-5桐木ハイツ202	03-6423-1433	http://ooimachi-jibika.com/	○	○	耳鼻咽喉科	牧野奈緒	
674	大井町とうまクリニック	140-0014	東京都品川大井1-16-2ブリリア大井町ラヴィアンタワー2	03-5718-4080	http://www.oimachi-toma-clin.com	○	○	内科<br>循環器科<br>呼吸器科	當間　弘子	
675	大井町なかじま眼科・内科	140-0014	東京都品川区大井２ー１ー１<br>大井2丁目メディカルセンター1階	03-3785-1255	https://nakajima-ganka-naika.com/	○	○	眼科<br>内科	中島　剛<br>中島　資子	

電話や情報通信機器を用いて診療を実施する医療機関の一覧（東京都）

	基本情報					事務連絡に基づく対応について				
	施設名	郵便番号	住所（都道府県から記載）	電話番号	ウェブサイトURL	初診の電話等を用いた診療の実施の有無	再診の電話等を用いた診療の実施の有無	対応診療科	担当医師名	対面診療が必要と判断した場合に連携する医療機関名（複数ある場合は複数、住所も併せて記載）
676	大崎こどもハート・アレルギークリニック	141-0001	東京都品川区北品川5-8-15北品川ﾎｰﾑｽﾞ１F	03-3440-8211	https://kodomo-heart.com/osaki/	○	○	小児科<br>アレルギー科	加藤　はるか	昭和大学病院（東京都品川区旗の台1-5-8）
677	大崎耳鼻咽喉科	141-6003	東京都品川区大崎2-1-1　Think　Park　Tower　3F	03-5745-0733	https://www.osaki-jibika.net		○	耳鼻咽喉科	八島隆敏<br>加藤智史<br>本田圭司<br>倉田奈都子	
678	大森耳鼻咽喉科	140-0013	東京都品川区南大井6-28-12ﾋｭｰﾘｯｸ大森ﾋﾞﾙ5F	03-6410-8633	http://www.omori-jibika.net		○	耳鼻咽喉科<br>ｱﾚﾙｷﾞｰ科	八島隆敏<br>高橋正時	
679	小澤医院	140-0011	東京都品川区東大井3-1-18	03-3298-0088	https://oimachi-ozawaclinic.com	○	○	小児科<br>内科	小澤　安文	東京品川病院（東京都品川区東大井6-3-22）<br>東邦大学医療センター大森病院(東京都大田区大森西6-11-1)
680	小野内科	142-0063	東京都品川区荏原5-16-19	03-3781-9580	http://www.ononaika.net	○	○	内科<br>小児科	小野竜	
681	柿島医院	140-0014	東京都品川区大井3-21-10	03-3775-5171	http://www.taikou.or.jp		○	内科<br>循環器内科<br>ペインクリニック内科	堀之内八千代<br>堀之内仁美<br>増田尚己<br>中野将孝	
682	かきのき眼科	142-0062	東京都品川区小山6-3-9ウエストヴィレッジビルディング4F	03-6426-4639	http://kakinokiganka.jp/	○	○	眼科	柿木一邦<br>伊藤由紀子	
683	KARADA(からだ)内科クリニック	141-0031	東京都品川区西五反田1-2-8 FUNDES五反田10F	03-3495-0192	①内科ホームページ<br>https://karada-naika.com/<br>②性感染症（性病）ホームページ<br>https://sti-check.com/	○	○	総合内科<br>感染症内科<br>性感染症（性病）<br>渡航外来	佐藤　昭裕	NTT東関東病院（東京都品川区東五反田5-9-22）<br>東京医科大学病院（東京都新宿区西新宿６−７−１）
684	眼科西大井クリニック	140-0015	東京都品川区西大井1-9-6	03-5746-0746	https://nishiooi.com/01clnc.htm	○	○	眼科	徳永　美佳	

電話や情報通信機器を用いて診療を実施する医療機関の一覧（東京都）

	基本情報					事務連絡に基づく対応について				
	施設名	郵便番号	住所（都道府県から記載）	電話番号	ウェブサイトURL	初診の電話等を用いた診療の実施の有無	再診の電話等を用いた診療の実施の有無	対応診療科	担当医師名	対面診療が必要と判断した場合に連携する医療機関名（複数ある場合は複数、住所も併せて記載）
685	きくち眼科	142-0053	東京都品川区中延4-4-8	03-3781-4306	http://www.kikuchinakanobu.com		○	眼科	渡部　通史	
686	銀座よしえクリニック品川院	141-0031	東京都品川区西五反田4-31-17 MYビル2F	0120-398-885	https://www.ginzabiyou.com/	○	○	皮膚科<br>美容皮膚科	担当医	
687	けいひんファミリークリニック	140-0011	東京都品川区東大井2-12-19　MKビル3階	03-5767-7755	https://www.med-kfc.com/	○	○	小児科<br>内科<br>アレルギー科	松本　道祐	
688	五反田なるど耳鼻咽喉科	1410022	東京都品川区東五反田5-28-10第２花谷ビル７階	364325644	https://naruto-orl.com		○	耳鼻咽喉科	鳴戸理佐	
689	五反田泌尿器科内科　すどうクリニック	141-0022	東京都品川区東五反田4-11-6クレスト五反田ビル2階B号	03-6432-5785	http://www.gotanda-minna.com/		○	泌尿器科<br>内科	首藤直樹	NTT東日本関東病院（東京都品川区東五反田5-9-22）
690	御殿山整形外科リハビリクリニック	141-0001	東京都品川区北品川5-4-1　大崎ブライトプラザ2F	03-3446-5108	http://www.gotenyama-seikei.jp		○	整形外科<br>リハビリテーション科	森末　光	
691	こどもの森クリニック	141-0021	東京都品川区上大崎３－３－１自転車総合ビル1階	03-5798-4141	http://kodomonomori-clinic.com		○	小児科	早崎理香	
692	こみね循環器科・内科クリニック	142-0053	東京都品川区中延5-13-16　1階	03-5750-6355	http://komine-cl.com		○	内科	小峰　武明	
693	小山中央診療所	142-0062	東京都品川区小山3-1-10	03-3712-3415	http://www.koyachuo-cl.com	○	○	内科<br>消化器内科	笹川　綾子	

電話や情報通信機器を用いて診療を実施する医療機関の一覧（東京都）

	基本情報					事務連絡に基づく対応について				
	施設名	郵便番号	住所（都道府県から記載）	電話番号	ウェブサイトURL	初診の電話等を用いた診療の実施の有無	再診の電話等を用いた診療の実施の有無	対応診療科	担当医師名	対面診療が必要と判断した場合に連携する医療機関名（複数ある場合は複数、住所も併せて記載）
694	さえきクリニック	142-0041	東京都品川区戸越6－4－3	03-5749-5821	http://saekiclinic.byoinnavi.jp/pc/	○	○	内科<br>小児科	佐伯明子	
695	サンタハウスこどもクリニック	142-0062	東京都品川区小山3-1-2　1階	03-3719-5533	http://www.santahousecodomo.jp/	○	○	小児科<br>アレルギー科<br>内科	山田勝敏	昭和大学病院（東京都品川区旗の台1-5-8）<br>荏原病院（東京都大田区東雪谷4-5-10）<br>都立広尾病院（東京都渋谷区恵比寿2-34-10）<br>日本赤十字社医療センター（東京都渋谷区広尾4-1-22）<br>NTT東日本関東病院（東京都品川区東五反田5-9-22）<br>東京品川病院（東京都品川区東大井6-3-22）<br>東邦大学大森病院（東京都大田区大森西6-11-1）<br>東邦大学大橋病院（東京都目黒区大橋2-22-36）
696	しばざき泌尿器クリニック	142-0062	東京都品川区小山６-4-14	03-5749-1233	http://www.shibazaki-clinic.jp/		○	泌尿器科<br>腎臓内科	柴崎　裕	
697	Jメンタル五反田駅前クリニック	141-0031	東京都品川区西五反田2-5-2五反田東幸ビル7F	03-3491-7776	http://www.j-mental.com/	○	○	精神科<br>心療内科<br>漢方内科	小林城治<br>その他	荏原病院(東京都大田区東雪谷四丁目5-10)<br>晴和病院(東京都新宿区弁天町91)<br>東京都立松沢病院(東京都世田谷区上北沢2-1-1)
698	城南こどもハート・アレルギークリニック	140-0004	東京都品川区南品川2-17-27三井南品川ﾊｲﾂ1階	03-3474-8010	https://kodomo-heart.com/jonan/	○	○	小児科<br>アレルギー科	田村　恵子	昭和大学病院（東京都品川区旗の台1-5-8）
699	昭和大学病院附属東病院	142-0054	東京都品川区西中延2-14-19	03-3784-8000	http://www.showa-u.ac.jp/SUHE/		○<br>外来予約済の患者さんのみ対応可能	糖尿糖代謝内分泌内科<br>脳神経内科<br>精神神経科<br>麻酔科<br>ペインクリニック<br>整形外科	山岸　昌一<br>小野　賢二郎<br>岩波　明<br>大江　克憲<br>稲垣　克記<br>恩田　秀寿<br>安達　太郎	
700	瀬底皮膚科クリニック	140-0011	東京都品川区旗の台5-7-6-108	03-3787-0115	https://www.sesoko-hifuka.com		○	皮膚科	三嶋　絵美	
701	竹内胃腸内科医院	141-0022	東京都品川区東五反田5-27-3第2野村ビル3階	03-5421-7149	https://takeuchi-giclinic.com	○	○	内科<br>消化器内科<br>胃腸内科	竹内義明	NTT東日本関東病院（東京都品川区）<br>昭和大学病院（東京都品川区）<br>東京都品川病院（東京都品川区）
702	たごファミリークリニック	142-0051	東京都品川区平塚2-15-15 丸二ビル 3階	03-3785-5250	http://tago-clinic.in.coocan.jp/		○	内科<br>小児科	田胡秀和	

電話や情報通信機器を用いて診療を実施する医療機関の一覧（東京都）

	基本情報					事務連絡に基づく対応について				
	施設名	郵便番号	住所（都道府県から記載）	電話番号	ウェブサイトURL	初診の電話等を用いた診療の実施の有無	再診の電話等を用いた診療の実施の有無	対応診療科	担当医師名	対面診療が必要と判断した場合に連携する医療機関名（複数ある場合は複数、住所も併せて記載）
703	とごし公園内科クリニック	142-0041	東京都品川区戸越5-11-8新川屋ビル1F	03-3784-2112	https://togoshipark-cl.com/	○	○	内科全般	冨田　英利	
704	戸越泌尿器科内科クリニック	142-0042	東京都品川区豊町1-3-17山崎ビル1階	03-5788-6120	http://togoshi-clinic.com/	○	○	泌尿器科<br>内科	丸山　修	
705	中村医院	142-0053	東京都品川区中延6-4-17	03-3764-0128	http://www.n-cl.jp	○	○	内科　整形　皮膚科	中村　秀紀	東邦大学医療センター大森病院（東京都大田区大森西6-11-1）
706	なるき内科クリニック	142-0043	東京都品川区二葉3-26-6-101	03-5702-2255	https://naruki-naika.com/	○	○	内科	成木　良瑛子	
707	西川医院	142-0064	東京都品川区旗の台4-1-5	03-3781-7825	http://hwm8.spaaqs.ne.jp/nishikawaiin/		○	内科<br>消化器内科<br>小児科	西川順一	
708	野本内科クリニック	142-0062	東京都品川区小山6-3-9-3階	03-5749-3300	http://www.nomotonaika.jp		○	内科	野本日出男	荏原病院（東京都大田区東雪谷4-5-10）<br>NTT東日本関東病院（東京都品川区東五反田5-9-22）<br>昭和大学病院（東京都品川区旗の台1-5-8）<br>東京共済病院（東京都目黒区中目黒2-3-8）
709	旗の台駅東口　いわた眼科	142-0054	東京都品川区西中延2-15-21<br>Ｔ＆Ｙ　YASUDAビル　１Ｆ	03-6426-6155	https://www.iwata.com/	○	○	眼科	岩田　文乃	
710	ハピコワクリニック五反田	141-0022	東京都品川区東五反田1－22－6五反田さくらビル5F	03-3444-8158	https://hapicowa-clinic.jp/	○	○	呼吸器内科・アレルギー科・内科・小児科	岸本　久美子	自院もしくは東京品川病院（東京都品川区東大井6-3-22）
711	はるクリニック西小山	142-0062	東京都品川区小山6-1-1	03-5794-8630	haru-clinic.jp	○	○	内科	飛川浩治	東京品川病院・NTT東日本関東病・院昭和大学病院

電話や情報通信機器を用いて診療を実施する医療機関の一覧（東京都）

	基本情報					事務連絡に基づく対応について				
	施設名	郵便番号	住所（都道府県から記載）	電話番号	ウェブサイトURL	初診の電話等を用いた診療の実施の有無	再診の電話等を用いた診療の実施の有無	対応診療科	担当医師名	対面診療が必要と判断した場合に連携する医療機関名（複数ある場合は複数、住所も併せて記載）
712	東品川クリニック	142-0002	東京都品川区東品川3-18-3　神興ビル3階	03-3472-6684	https://www.hs.-clinic.com	○	○	内科<br>小児科<br>総合診療科	平塚祐介	
713	二葉医院	142-0043	東京都品川区二葉1-7-9	03-3782-2748			○	内科<br>麻酔科	羽尻裕美	
714	穂坂クリニック	142-0062	東京都品川区小山3-24-6	03-3781-1351	https://hosaka-clinic.com	○	○	内科リウマチ科<br>アレルギー科	穂坂　茂	
715	三浦医院	146-0031	東京都品川区西五反田5-10-6-104	03-3492-5225	https://miuraiin.jp/	○	○	内科<br>外科	三浦　和裕	
716	みしま内科	142-0064	東京都品川区旗の台5-8-5	03-6426-7728	http://mishima-naika.com		○	内科	三嶋晃	
717	武蔵小山胃腸内視鏡クリニック	142-0062	東京都品川区小山4-13-13	0120-949-922	http://www.msk-cl.com	○	○	内科	土井　健一	
718	むさしこやま眼科	142-0062	東京都品川区小山3-15-1パークシティ武蔵小山ザモール2階2-H	03-3788-4639	https://www.musashikoyamaganka.jp/	○	○	眼科	熊谷泰雅	かきのき眼科（東京都品川区小山6-3-9ウエストヴィレッジビルディング4F）
719	武蔵小山腎泌尿器クリニック	142-0062	東京都品川区小山4ー3ー12　TK武蔵小山ビル2F	03-6426-2832	http://musako-uro.com/		○	泌尿器科<br>内科<br>腎臓内科	還田　稔	昭和大学病院（東京都品川区旗の台1-5-8）NTT東日本関東病院（東京都品川区東五反田5-9-22）都立荏原病院（東京都大田区東雪谷4-5-10）
720	むらさき乳腺クリニック五反田	141-0031	東京都品川区西五反田2-19-2荒久ビル6F	03-3495-0881	http://www.murasakibreastclinic.com/	○	○	乳腺外科	池田　紫	

電話や情報通信機器を用いて診療を実施する医療機関の一覧（東京都）

	基本情報					事務連絡に基づく対応について				
	施設名	郵便番号	住所（都道府県から記載）	電話番号	ウェブサイトURL	初診の電話等を用いた診療の実施の有無	再診の電話等を用いた診療の実施の有無	対応診療科	担当医師名	対面診療が必要と判断した場合に連携する医療機関名（複数ある場合は複数、住所も併せて記載）
721	医療法人社団めぐみ会めぐみクリニック目黒２	141-0021	東京都品川区上大崎2-12-2　ﾐｽﾞﾎﾋﾞﾙ3F	03-5422-8391	https://www.megumicl.com/2/	○	○	内科	菊地　俊介<br>税所　俊光<br>田村　豊	
722	めぐろ駅東口<br>メンタルクリニック	141-0021	東京都品川区上大崎3ー3ー1 オバタビル5F	03-6277-2188	https://www.mh-mental.jp/		○	心療内科<br>精神科	堺　英彰	
723	目黒みらい内科クリニック	141-0021	東京都品川区上大崎2-13-26　メイプルトップビル3F	03-6721-6672	https://www.meguro-clinic.net/	○	○	内科<br>呼吸器内科<br>糖尿病内科	太田　啓介	
724	もちづき内科クリニック	142-0041	東京都品川区戸越4-9-12	03-6426-2711	https://www.mochizuki-medical.com/	○	○	内科<br>アレルギー科	望月香織	東京品川病院<br>昭和大学付属病院<br>NTT東日本関東病院
725	山内クリニック	142-0062	東京都品川区小山5-17-4、1階	03-3781-2890	www.yamauchicl.com		○	内科	山内健義	
726	山崎眼科	142-0051	東京都品川区平塚2-15-15　1F	03-3782-6666	http://www.pluto.dti.ne.jp/yamazaki-ganka/		○	眼科	山崎　斉	
727	ゆきこどもクリニック	142-0053	東京都品川区中延5-3-8-1F	03-3784-2555	http://yuki-kodomo.com	○	○	小児科・内科・アレルギー科	竹下　由紀子	昭和大学病院（東京都品川区旗の台1-5-8）荏原病院（東京都大田区東雪谷4-5-10）
728	ゆたか診療所	142-0042	東京都品川区豊町4-18-21	03-3781-4723	http://www.yutakashin.jp		○	内科<br>整形外科<br>皮膚科	権守　光夫<br>吉岡　雅之	
729	依田医院	142-0041	東京都品川区戸越6-7-26-201	03-3781-2865			○	内科	依田欣之	

電話や情報通信機器を用いて診療を実施する医療機関の一覧（東京都）

	基本情報					事務連絡に基づく対応について				
	施設名	郵便番号	住所（都道府県から記載）	電話番号	ウェブサイトURL	初診の電話等を用いた診療の実施の有無	再診の電話等を用いた診療の実施の有無	対応診療科	担当医師名	対面診療が必要と判断した場合に連携する医療機関名（複数ある場合は複数、住所も併せて記載）
730	わかばやし眼科	142-0053	東京都品川区中延3-2-18-101	03-6421-5900	https://www.wakabayashi-ganka.jp/		○	眼科	若林俊子	
731	和田外科醫院	142-0051	東京都品川区平塚1-8-18	03-3781-4936			○	整形外科<br>内科	和田信裕　明珠	
732	和田整形外科	142-0041	東京都品川区戸越6−8−20	03-5498-2555	https://www.i-jin.jp/tokyo/09_sinagawa/03_wada_seikei/index.html	○	○	整形外科	和田次郎	昭和大学病院（品川区旗の台1−5−8）<br>NTT東日本関東病院（品川区東五反田5−9−22）
733	東京シティクリニック品川	140-0003	東京都品川区八潮5-10-27<br>パトリア品川別館2F	03-3790-2551	http://tokyocityclinic-shinagawa.com/		○	内科<br>小児科	近藤　敬子	東京品川病院（東京都品川区東大井6-3-22）<br>愛育病院（東京都港区芝浦1-16-10）
734	奥秋整形外科	140-0013	東京都品川区南大井６−２４−９<br>興産大森ビル４階	03-5753-8282	https://www.okuaki-seikei.com		○	整形外科	奥秋　保	
735	KARADA内科クリニック五反田院	1410031	東京都品川区西五反田1-2-8　10F	03-3495-0192	https://karada-naika.com/	○	○	内科	佐藤昭裕	
736	たつのこどもクリニック	140-0014	東京都品川区大井２−２−１１　１F	03-3775-8600		○	○	小児科	田角恭子	
737	たつのシティタワークリニック	140-0014	東京都品川区大井1-31-1　102	03-6303-8737	https://tatsuno-cityclinic.com/	○		小児科<br>小児神経科<br>小児精神科	田角　勝	昭和大学病院（東京都品川区旗の台1-5-8）<br>東邦大学病院（東京都大田区大森6-11-1）
738	みんなのクリニック大井町	140-0004	東京都品川区南品川6-15-5	03-6433-0280	https://oimachi.minnano.clinic/	○	○	内科　小児科<br>皮膚科<br>泌尿器科	年森慎一	東京品川病院<br>（東京都品川区東大井６丁目３−２２）

電話や情報通信機器を用いて診療を実施する医療機関の一覧（東京都）

	基本情報					事務連絡に基づく対応について				
	施設名	郵便番号	住所（都道府県から記載）	電話番号	ウェブサイトURL	初診の電話等を用いた診療の実施の有無	再診の電話等を用いた診療の実施の有無	対応診療科	担当医師名	対面診療が必要と判断した場合に連携する医療機関名（複数ある場合は複数、住所も併せて記載）
739	大崎消化器内科クリニック	141-0001	東京都品川区北品川５－４－１<br>大崎ブライトプラザ２階	03-5791-3388	https://www.osaki-clinic.com/		○	内科	倉岡　賢輔<br>大岩　弘子	NTT東日本関東病院<br>（東京都品川区東五反田5-9-22）
740	みやざきRCクリニック	140-0001	東京都品川区北品川2-23-2-3F	03-5460-1159	https://kita-shinagawa.clinic		○	内科<br>呼吸器内科	宮崎　雅樹	
741	戸越銀座駅近内科クリニック	142-0063	東京都品川区平塚1-7-16-1F	03-5749-1054	http://togoshinaika.com/	○	○	内科	市川　晋也	
742	磯村クリニック	152-0023	東京都目黒区八雲１－１０－１７	03-3723-8841	http://www.is-clinic.com		○	皮膚科・婦人科・漢方内科	磯村知子	
743	伊藤小児科・アレルギー科	152-0034	東京都目黒区緑が丘3-8-8	03-3729-1312		○	○	小児科<br>アレルギー科	伊藤　幸生	
744	医療法人社団一星会<br>目黒通り消化器外科・内科クリニック	153-0064	東京都目黒区下目黒6-1-27,1F	03-3713-1117	https://meguro-ikamera.com/	○	○	内科	川口　大輔	
745	医療法人社団恵俊会　こんどう医院	152-0002	東京都目黒区目黒本町5-27-17-101	03-5768-7317		○	○	内科	近藤俊彦	昭和大学病院（東京都品川区旗の台1-5-8）
746	医療法人社団航洋会<br>目黒通りハートクリニック	152-0004	東京都目黒区鷹番1-10-2-1F	03-6303-3328	http://meguro-heartclinic.com/	○	○	内科<br>小児科	安田　洋	
747	医療法人社団法山会<br>山下診療所自由が丘	152-0035	東京都目黒区自由が丘1-30-3　自由が丘東急ビル7F	03-3724-3811	http://hozankai.com/	○	○	内科・循環器<br>呼吸器・血液<br>消化器・ｱﾚﾙｷﾞｰ	山下・森川<br>軽部・長門<br>三神・中村<br>都築	

電話や情報通信機器を用いて診療を実施する医療機関の一覧（東京都）

	基本情報					事務連絡に基づく対応について				
	施設名	郵便番号	住所（都道府県から記載）	電話番号	ウェブサイトURL	初診の電話等を用いた診療の実施の有無	再診の電話等を用いた診療の実施の有無	対応診療科	担当医師名	対面診療が必要と判断した場合に連携する医療機関名（複数ある場合は複数、住所も併せて記載）
748	医療法人社団めぐみ会 自由が丘メディカルプラザ	152-0035	東京都目黒区自由が丘2-11-16<br>自由が丘日能研ビル2F	03-6421-1080	https://www.jiyugaokamp.com/	○	○	小児科<br>内科	髙嶋能文<br>坂本真保<br>齋藤義弘<br>田村豊<br>山岸征嗣<br>早田輝子<br>小船雅義<br>須藤英訓	
749	医療法人社団めぐみ会<br>自由が丘メディカルプラザ2	152-0035	東京都目黒区自由が丘2-11-16ニューパルビュー3F	03-5731-3565	https://www.jiyugaokamp.com/s/		○	小児科	髙嶋　能文<br>大野　貴美子<br>坂本　真保<br>齋藤　義弘<br>森川　日出男	
750	医療法人社団　順信会<br>目黒整形外科内科	152-0002	東京都目黒区目黒本町6-1-2　友愛ビル1階	03-5725-3677	http://www.meguro-seikei.com		○	整形外科<br>内　　科	鈴木和也（整形）鈴木　仁（内科）	
751	ウェルビーイングクリニック駒沢公園	152-0021	東京都目黒区東が丘2-15-1-102	03-6804-0644	https://clinic.zenplace.co.jp/	○	○	循環器内科/内科	布施　淳	国立病院機構東京医療センター（東京都目黒区東が丘2-5-1）
752	ウォブクリニック中目黒	153-0061	東京都目黒区中目黒1-10-23シティホームズ中目黒　アネックス2F	0120-411-281	https://wove.jp/	○	○	美容皮膚科<br>皮膚科	高瀬　聡子<br>太田　洋美	
753	おおの内科外科クリニック	153-0063	東京都目黒区目黒4-26-5 上田ビル１階	03-6303-3822	https://ono-naikageka.jp/	○	○	内科<br>外科	大野 雅央	東京共済病院（東京都目黒区中目黒2-3-8 ）
754	おおはしさくらクリニック	153-0044	東京都目黒区大橋2-23-1西渋谷ハイウエービル５Ｆ	03-6277-1017			○	内科、糖尿病科	大井　一輝<br>小森　広美	
755	学芸大西口クリニック	152-0004	東京都目黒区鷹番３－７－６NEWWEST3階	03-5773-0615	www.gakugeidai.jp	○	○	内科皮膚科	佐藤克二郎	
756	加藤内科クリニック	153-0051	東京都目黒区上目黒3-1-14	03-3792-8710	http://www.katoh-med.jp/	○	○	内科	加藤　陽一　柴田　祥子　加藤裕佳子	東京共済病院(東京都目黒区中目黒２－３－８)

電話や情報通信機器を用いて診療を実施する医療機関の一覧（東京都）

	基本情報					事務連絡に基づく対応について				
	施設名	郵便番号	住所（都道府県から記載）	電話番号	ウェブサイトURL	初診の電話等を用いた診療の実施の有無	再診の電話等を用いた診療の実施の有無	対応診療科	担当医師名	対面診療が必要と判断した場合に連携する医療機関名（複数ある場合は複数、住所も併せて記載）
757	北神経内科平山記念クリニック	153-0064	東京都目黒区下目黒5-16-16	03-5768-1235	https://www.kitashinkei.com/	○	○	神経内科<br>内科	北　耕平	東京共済病院（目黒区）<br>日本赤十字社医療センター（渋谷区）
758	銀座よしえクリニック都立大院	152-0032	東京都目黒区平町 1-23-14	0120-398-885	https://www.ginzabiyou.com/	○	○	内科<br>皮膚科<br>美容皮膚科	青木　晃<br>廣瀬　嘉恵	
759	五本木クリニック	152-0001	東京都目黒区中央町2-18-14ネオアージュ祐天寺1F	03-5721-7000	gohongi-clinic.com	○	○	内科/泌尿器科	桑満　おさむ	東京医療センター（東京都目黒区東が丘２－５－１）<br>東京共済病院（東京都目黒区中目黒2-3-8　）
760	すけがわクリニック	153-0064	東京都目黒区下目黒3-13-8	03-3760-8642		○	○	内科　小児科<br>循環器科	祐川隆平	
761	洗足駅前クリニック	152-0012	東京都目黒区洗足2-25-19 アソルティ洗足1階	03-3786-1009	https://senzoku-clinic.com	○	○	内科・消化器内科　　皮膚科	齋藤　佑介<br>齋藤　美穂	
762	友成クリニック	153-0063	東京都目黒区目黒4-10-10	03-3715-1181			○	内科	友成　治夫	
763	都立大整形外科クリニック	152-0032	東京都目黒区平町1-26-3　スミール都立大202	03-6404-8550	https://ar-ex.jp/toritsudai		○	整形外科	山田祐子<br>林英俊<br>平田正純<br>清水勇樹	
764	なんば耳鼻咽喉科	152-0012	東京都目黒区洗足２－７－１５－２０１	03-5842-1155	http://namba-clinic.com		○	耳鼻咽喉科	難波　真由美	
765	ふくしま内科こどもクリニック	152-0023	東京都目黒区八雲1－4－17	03-5731-2201	https://fukushima-naika-kodomo-cl.jp/	○	○	内科<br>小児科	福島　優	

電話や情報通信機器を用いて診療を実施する医療機関の一覧（東京都）

	基本情報					事務連絡に基づく対応について				
	施設名	郵便番号	住所（都道府県から記載）	電話番号	ウェブサイトURL	初診の電話等を用いた診療の実施の有無	再診の電話等を用いた診療の実施の有無	対応診療科	担当医師名	対面診療が必要と判断した場合に連携する医療機関名（複数ある場合は複数、住所も併せて記載）
766	福田医院	153-0061	東京都目黒区中目黒3-22-11	03-3712-2868			○	内科 小児科	福田　達弥	
767	ふるかわメディカルクリニック	152-0023	東京都目黒区八雲５－１５－５	03-6459-5539	http://www.furukawa-mc.com		○	内科<br>皮膚科	古川祐介<br>古川直子	
768	ポポロ小児科自由が丘	152-0035	東京都目黒区自由が丘1-24-14	03-5731-8806	http://popolo.tokyo/	○	○	小児科	竹内　邦子	
769	目黒ケイホームクリニック	153-0061	東京都目黒区中目黒4-5-1-エースビル2階	03-5722-5500	www.megurokhome@gmail.com	○	○	内科、呼吸器内科、アレルギー科、循環器内科	安藤克利	
770	もも耳鼻咽喉科	152-0032	東京都目黒区平町1ー27ー8　FPC 平町ビル3階	03-3718-3387			○	耳鼻咽喉科	山崎　ももこ	
771	祐天寺たけまさクリニック	153-0052	東京都目黒区祐天寺２－１５－６－１０１	03-5768-1336	http://www.takemasa-clinic.com	○	○	内科	長　剛正	
772	祐天寺ウィメンズヘルスクリニック	153-0052	東京都目黒区祐天寺２－７－２３　２Ｆ	03-5708-5081	http://www.satoh-womenshealth.com	○	○	産婦人科	佐藤　真之介	
773	よう子　みんなのクリニック	153-0051	東京都目黒区上目黒2-42-11グレイス中目黒1階	03-6412-8188	https://yoko-minnano.clinic/	○	○	内科<br>小児科	眞壁　陽子	
774	吉田医院	152-0003	東京都目黒区碑文谷1-28-11	03-3712-3396	http://yosida-iin.com	○	○	内科神経内科	吉田　裕	厚生中央病院（東京都目黒区三田１－１１－７）<br>東京医療センター（東京都目黒区東が丘２－５－１）<br>荏原病院（東京都大田区東雪谷４－５－１０）

電話や情報通信機器を用いて診療を実施する医療機関の一覧（東京都）

	基本情報					事務連絡に基づく対応について				
	施設名	郵便番号	住所（都道府県から記載）	電話番号	ウェブサイトURL	初診の電話等を用いた診療の実施の有無	再診の電話等を用いた診療の実施の有無	対応診療科	担当医師名	対面診療が必要と判断した場合に連携する医療機関名（複数ある場合は複数、住所も併せて記載）
775	ロコクリニック中目黒	153-0043	東京都目黒区東山1-6-5シティハウス中目黒ステーションコート1階	03-5722-6565	https://loco-clinic.com/	○	○	内科、小児科	嘉村　洋志<br>瀬田　宏哉	東京医療センター（ 東京都目黒区東が丘2-5-1）
776	にっとのクリニック	152-0011	東京都目黒区原町2-1-24	03-5704-4092	https://nittono-clinic.com	○	○	小児科	荻原(谷川)仁美	
777	自由が丘内科クリニック	152-0035	東京都目黒区自由が丘2-8-17グランデ自由が丘1F	03-3723-7888	https://jiyugaoka-naika.clinic	○	○	消化器内科、内科、循環器内科、呼吸器内科、内分泌代謝内科、アレルギー科、肛門内科、泌尿器科、皮膚科など	松原　健朗	三宿病院(東京都目黒区上目黒5丁目33−12)<br>東京医療センター(東京都目黒区東が丘2丁目5−1)昭和大学病院(東京都品川区旗の台1丁目5−8)など
778	あさもとクリニック	144-0052	東京都大田区蒲田3-18-12-102	03-5480-9633	http://asamoto-clinic.com/		○	脳神経外科<br>一般内科<br>アレルギー科	朝元　美利	
779	池上さいとう内科クリニック	146-0082	東京都大田区池上8-23-15	03-3757-3110	https://www.ikegami-saito.com		○	内科<br>脳神経内科	齋藤 悠	昭和大学病院(東京都品川区旗の台1-5-8)<br>東邦大学医療センター大森病院(東京都大田区大森西6-11-1)<br>池上総合病院(東京都大田区池上6-1-19)<br>松井病院(東京都大田区池上2-7-10)<br>荏原病院(東京都大田区東雪谷四丁目5-10)
780	いけがみ耳鼻咽喉科クリニック	146-0082	東京都大田区池上３－３０－１７	03-5700-6733	https://ikegami-jibika.com/		○	耳鼻咽喉科	工藤 睦男	
781	池田耳鼻咽喉科医院	143-0015	東京都大田区大森西3-21-13　2階	03-3761-0792			○	耳鼻咽喉科<br>小児科<br>内科	池田 文	東邦大学医療センター大森病院（東京都大田区大森西6-11-1）
782	いしがき耳鼻咽喉科クリニック	146-0093	東京都大田区矢口1-19-5エスポワール矢口1F	03-3757-3387	https://ishigaki-ent.com/	○	○	耳鼻咽喉科	石垣　高志	
783	伊丹内科医院	145-0065	東京都大田区東雪谷２－１７－１、グランディ雪谷１階	03-3748-0028	https://www.itaminaika.com	○	○	内科	伊丹　滋人	

電話や情報通信機器を用いて診療を実施する医療機関の一覧（東京都）

	基本情報					事務連絡に基づく対応について				
	施設名	郵便番号	住所（都道府県から記載）	電話番号	ウェブサイトURL	初診の電話等を用いた診療の実施の有無	再診の電話等を用いた診療の実施の有無	対応診療科	担当医師名	対面診療が必要と判断した場合に連携する医療機関名（複数ある場合は複数、住所も併せて記載）
784	いまい耳鼻咽喉科	145-0063	東京都大田区南千束3-18-12	03-3726-3387	http://home.a01.itscom.net/imaijibi		○	耳鼻咽喉科	今井容子	
785	医療法人財団　安田病院	143-0016	東京都大田区大森北1-11-18	03-3761-1023	https://www.yasuda-hsp.jp/	○	○	整形外科<br>整形外科<br>整形外科<br>整形外科<br>外　科<br>外　科<br>内　科<br>内　科	明妻　裕孝<br>楠瀬　浩一<br>松尾　亮平<br>浅沼　雄太<br>河野　修三<br>安田　雄一郎<br>小池　牧子<br>袋　瑛子	
786	医療法人社団オーシーエフシー会大川こども＆内科クリニック	146-0095	東京都大田区多摩川1-6-16	03-3758-0920	https://ocfc.jp		○	小児科　内科	大川　洋二	
787	医療法人社団くどうちあき脳神経外科クリニック	143-0016	東京都大田区大森北1-23-10	03-5767-0226	https://kudohchiaki.jp/	○	○	脳神経外科<br>心療内科	工藤　千秋	
788	医療法人社団涓泉会　山王リハビリ・クリニック	145-0065	東京都大田区東雪谷3-4-2	03-5754-2672	https://www.sanno-rc.com/	○	○	リハビリテーション科・整形外科・内科	森　英二/前島早代/安部　佑/森　武男	
789	医療法人社団誠知会　ひだまりクリニック	144-0056	東京都大田区西六郷1-18-11	03-6715-9388	http://www.seichikai.or.jp	○	○	内科	松本　昌和	
790	医療法人社団奏那　ぞうしきこどもクリニック	144-0055	東京都大田区仲六郷二丁目45番地16号	03-3733-2288	https://zoshiki.jp/	○	○	小児科、アレルギー科	植松浩司	東邦大学大森病院（東京都大田区大森西６丁目１１－１）
791	医療法人社団 羽田バス通りクリニック	144-0043	東京都大田区羽田３－３－１３　鈴久ビルマンション1階	03-5735-0808	http://haneda-bus-road-clinic.com/		○	内科、消化器内科、外科、皮膚科	田代　健一	
792	医療法人社団　松井病院	146-0082	東京都大田区池上2-7-10	03-3752-1111	http://www.matsuihsp.or.jp/		○	循環器内科<br>消化器内科<br>呼吸器内科<br>腎臓内科<br>糖尿病内科<br>外科<br>整形外科<br>眼科	橋秀昭、西尾和晃、松井秀夫、宮田一、吉田俊太郎、多田浩子、荒瀬勉、椛澤由博、定方博史、岡良臣、林まりあ、下平和	

電話や情報通信機器を用いて診療を実施する医療機関の一覧（東京都）

	基本情報					事務連絡に基づく対応について				
	施設名	郵便番号	住所（都道府県から記載）	電話番号	ウェブサイトURL	初診の電話等を用いた診療の実施の有無	再診の電話等を用いた診療の実施の有無	対応診療科	担当医師名	対面診療が必要と判断した場合に連携する医療機関名（複数ある場合は複数、住所も併せて記載）
793	医療法人社団癒合会<br>AI和合クリニック	144-0033	東京都大田区東糀谷2-11-18　1階	03-6423-2902	https://www.takanawa-clinic.com/aiwagoclinic/	○	○	内科	長岡　美妃	
794	医療法人社団　祐真会<br>マチノマ大森内科クリニック	143-0015	東京都大田区大森西3-1-38マチノマ大森3階	03-5471-5556	https://machinoma-naika.com/	○	○	内科<br>消化器内科<br>呼吸器内科<br>循環器内科	河合　剛	東邦大学医療センター大森病院（東京都大田区大森西6-11-1）<br>牧田総合病院（東京都大森北1-34-6）
795	医療法人社団緑清会<br>しのはらクリニック	144-0051	東京都大田区西蒲田7-44-6-2F	03-3737-4114	http://shinoharaclinic.com/		○	内科<br>消化器内科	篠原玄夫<br>篠原正夫<br>篠原美絵	
796	岩﨑内科クリニック	146-0095	東京都大田区多摩川1-23-8	03-3759-3771	https://iwasakicl.com		○	内科	武藤　浩<br>武藤　ます江	
797	うつのみや内科クリニック	145-0062	東京都大田区北千束1-44-1リバーレプラザ大岡山1階	03-5726-9807	http://www.utsunomiya-cl.jp/	○	○	内科	宇都宮　幹子	
798	梅屋敷内科クリニック	144-0052	東京都大田区蒲田2-2-13ヴィラはなぞの一階	03-3732-2831	https://umeyashiki-naika.com/	○	○	内科・呼吸器内科・循環器内科	辻川　雄	
799	大田病院附属大森中診療所	143-0014	東京都大田区大森中1-22-2	03-6404-2301	http://www.oomorinakashin.jp/		○	内科	山本　博	
800	大森赤十字病院	143-8527	東京都大田区中央4-30-1	03-3775-3111	http://omori.jrc.or.jp/		○	呼吸器内科他全24科	各診療科医師	
801	大森町駅前内科小児科クリニック	143-0015	東京都大田区大森西3-20-9 1F	03-6423-0428	https://omorimachi.com	○	○	内科、小児科、アレルギー科	柳澤　亮<br>田口　眞寿美	東邦大学大森病院（東京都大田区大森西６丁目１１−１）<br>東京労災病院（東京都大田区大森南４丁目１３−２１）<br>大森赤十字病院（東京都大田区中央４丁目３０−１）

電話や情報通信機器を用いて診療を実施する医療機関の一覧（東京都）

	基本情報					事務連絡に基づく対応について				
	施設名	郵便番号	住所（都道府県から記載）	電話番号	ウェブサイトURL	初診の電話等を用いた診療の実施の有無	再診の電話等を用いた診療の実施の有無	対応診療科	担当医師名	対面診療が必要と判断した場合に連携する医療機関名（複数ある場合は複数、住所も併せて記載）
802	おばら消化器肛門クリニック	146-0093	東京都大田区矢口2-11-23	03-3750-8218	obara-clinic.com	○	○	内科<br>肛門科	小原　邦彦	池上総合病院（東京都大田区池上6-1-19）<br>大森赤十字病院（東京都大田区中央4-30-1）<br>東邦大学大森病院（東京都大田区大森西6-11-1）
803	小原りぼんクリニック	146-0085	東京都大田区久が原2-17-18	03-6410-6110	https://obara-rbn.com/		○	内科	小原　太郎	
804	鎌田医院	144-0033	東京都大田区東糀谷2ー7ー16	03-3741-0662	https://www.hospita.jp/detail/212/announce/16397/		○	内科	濱部　晃<br>松下麻衣子	
805	蒲田いだ耳鼻咽喉科	144-0052	東京都大田区蒲田5-28-7	03-3735-3387	https://kamata-ida-ent.com	○	○	耳鼻咽喉科	井田　裕太郎	
806	蒲田医師会立西六郷診療所	144-0056	東京都大田区西六郷3-1-7	03-6428-6855	https://nishiroku-cl.com/	○	○	内科<br>小児科	大藏美奈子<br>他	
807	蒲田内科クリニック	144-0052	東京都大田区蒲田５－２８－１８　２階	03-6715-7337	http://kamata-cl.jp		○	内科<br>循環器内科	浅見　光一	東京蒲田医療センター(大田区南蒲田２－１９－２)
808	銀座よしえクリニック大岡山院	145-0062	東京都大田区北千束1-45-11	0120-398-885	https://www.ginzabiyou.com/	○	○	皮膚科<br>美容皮膚科	担当医	
809	久が原こどもクリニック	146-0085	東京都大田区久が原5-14-14ライオンズプラザ1F	03-5747-2802		○	○	小児科、内科	森川和洋<br>森川日出男	荏原病院（東京都大田区東雪谷4-5-10）
810	くわはら耳鼻咽喉科クリニック	145-0064	東京都大田区上池台５－３２ー４	03-5754-8733	http://www.kuwahara-jibika.jp/	○	○	耳鼻咽喉科	桑原　大輔	

電話や情報通信機器を用いて診療を実施する医療機関の一覧（東京都）

	基本情報					事務連絡に基づく対応について				
	施設名	郵便番号	住所（都道府県から記載）	電話番号	ウェブサイトURL	初診の電話等を用いた診療の実施の有無	再診の電話等を用いた診療の実施の有無	対応診療科	担当医師名	対面診療が必要と判断した場合に連携する医療機関名（複数ある場合は複数、住所も併せて記載）
811	京浜診療所	144-0046	東京都大田区東六郷1-27-4	03-3734-7288	http://www.keihin-shin.jp/		○	内科	今井　保	
812	糀谷こどもクリニック	144-0034	東京都大田区西糀谷４－２９－１６<br>ステーションツインタワーズ糀谷フロントウエスト１階	03-3745-668	http//www.koujiya-kodomo.com	○	○	小児科	高木　優樹	東邦大学医療センター大森病院（ 東京都大田区大森西６丁目１１−１）
813	眞田クリニック	146-0082	東京都大田区池上４－３０－３	03-3755-1661	http://www.shinkeikai.com/		○	脳神経外科<br>循環器科<br>形成外科	眞田　祥一<br>池内　聡<br>荒川　秀樹<br>安部　開人<br>栗原　邦広	
814	山王耳鼻咽喉科	143-0023	東京都大田区山王2-1-7　神輿大森ビル2階	03-5743-3300	https://sanno-ent-clinic.qwc.jp/	○	○	耳鼻咽喉科	緒方　哲郎	
815	社会医療法人財団仁医会牧田総合病院蒲田分院	144-0051	東京都大田区西蒲田４丁目２２番１号	03-5748-5020	http://www.makita-hosp.or.jp/kamata/		○	内科	越村　勲<br>秋山　敏夫	社会医療法人財団仁医会牧田総合病院（東京都大田区大森北１丁目３４番６号）
816	すずきこどもクリニック	145-0073	東京都大田区北嶺町１２－５－１Ｆ	03-5754-5800	http://suzuki-kids.jp/index.html		○	小児科	鈴木　竜洋	
817	鈴木クリニック	143-0015	東京都大田区大森西5-25-11	03-3765-9400			○	内科<br>小児科	鈴木　和郎	東邦大学医療センター大森病院（東京都大田区大森西6-11-1）
818	鈴木内科医院	143-0023	東京都大田区山王3-29-1	03-3772-1853	www.otaku-suzuki-naika.com/	○	○	内科　老年内科<br>消化器内科	鈴木央	
819	せせらぎクリニック多摩川	145-0071	東京都大田区田園調布１－３３－３田園ホームズ１階	03-5755-5207	https://familymed.seseragic.com		○	内科<br>小児科<br>皮膚科	富塚　太郎	

電話や情報通信機器を用いて診療を実施する医療機関の一覧（東京都）

	基本情報					事務連絡に基づく対応について				
	施設名	郵便番号	住所（都道府県から記載）	電話番号	ウェブサイトURL	初診の電話等を用いた診療の実施の有無	再診の電話等を用いた診療の実施の有無	対応診療科	担当医師名	対面診療が必要と判断した場合に連携する医療機関名（複数ある場合は複数、住所も併せて記載）
820	染川医院	146-0085	東京都大田区久が原5-11-10	03-3751-7254	http://www.somekawa-iin.com/		○	内科<br>心療内科　老年精神科	染川実香	
821	髙野病院	144-0033	東京都大田区東糀谷3-3-24	03-3741-0011	http://www.takanohosp.jp	○<br>当院に受診歴のある患者に対してのみ	○	内科　外科<br>整形外科<br>皮膚科　眼科	髙野　研一郎<br>髙野　恭子<br>髙野　明枝	
822	たけうち皮フ科クリニック	144-0094	東京都大田区蒲田4-10-14 あすとウィズ3階	03-3734-1655	http://www.takeuchiderm.jp/		○<br>診察したことのない症状や疾病は、視診と触診のできない電話再診の対象外	皮膚科	竹内常道	
823	竹内内科小児科医院	145-0072	東京都大田区田園調布本町40-12-201	03-3721-5222	https://www.takeuchi-iin.jp/	○	○	内科 小児科 糖尿病内科 アレルギー科 皮膚科	五藤　良将	
824	立石医院	145-0073	東京都大田区北嶺町３－２７	03-3720-3404	http://www.tateishi-iin.jp		○	内科	立石　淳一	
825	つぼた内科眼科クリニック	144-0055	東京都大田区仲六郷2-44-15	03-3731-7772	https://tsubota.clinic/	○	○	内科<br>眼科	坪田　貴也<br>坪田　恵美	東京蒲田医療センター（東京都大田区南蒲田2-19-2）
826	田園調布長田整形外科	145-0071	東京都大田区田園調布2-41-2NTTビル1階	03-5483-7070	http://www.osada-seikei.com/		○	整形外科内科リハビリテーション科	長田夏哉	東京医療センター、昭和大学病院、ほか
827	田園調布中央病院	145-0071	東京都大田区田園調布2-43-1	03-3721-7121	https://denenchofu-hp.jp/		○	全科	全医師	
828	東急株式会社　東急病院	145-0062	東京都大田区北千束3-27-2	03-3718-3331	http://www.tokyu-hospital.jp/		○	内科、消化器・肝臓内科、腎臓・透析内科、糖尿病内科、循環器内科、呼吸器内科、神経内科、心療内科、外科、消化器外	[illegible]、吉田一明、吉田沙佑美、内山威人、小澁正和、田尻進、上田仁美、松平浩、山中修一郎、田中明彦、小川和彦、友安雅子、宇都宮正範、齊藤惠介、加藤大介、筋野甫、高田道哉、梶原幹生、相良博[illegible]	

電話や情報通信機器を用いて診療を実施する医療機関の一覧（東京都）

	基本情報					事務連絡に基づく対応について				
	施設名	郵便番号	住所（都道府県から記載）	電話番号	ウェブサイトURL	初診の電話等を用いた診療の実施の有無	再診の電話等を用いた診療の実施の有無	対応診療科	担当医師名	対面診療が必要と判断した場合に連携する医療機関名（複数ある場合は複数、住所も併せて記載）
829	東京蒲田病院	144-0051	東京都大田区西蒲田7-10-1	03-3733-0525	https://www.t-kamata-hosp.or.jp/		○	標榜科全て		
830	東京メモリークリニック蒲田	144-0053	東京都大田区蒲田本町2-4-2アクシード蒲田本町1階	03-6715-7873	https://memory-clinic.jp/		○	内科<br>神経内科	園田　康博	
831	東邦大学医療センター大橋病院	153-8515	東京都目黒区大橋2-22-36	03-3468-1251	https://www.ohashi.med.toho-u.ac.jp/		○<br>処方箋FAX対応<br>（診療未実施）	全科	各診療科担当医師	
832	東邦大学医療センター大森病院	143-8541	東京都大田区大森西6-11-1	03-3762-4151	https://www.omori.med.toho-u.ac.jp/		○<br>処方箋ＦＡＸ対応（診療未実施）	泌尿器科、産婦人科、小児科、乳腺外科、皮膚科、呼吸器科、東洋医学科、膠原病科、血液腫瘍科、眼科、整形外科、消化器	各診療科担当医師	
833	とちぎクリニック	143-0023	東京都大田区山王２－１９－８	03-3777-7712<br>070-7489-2324	http://www.tochigiclinic.com	○	○	産婦人科	栃木　明人	
834	中馬込おがわ内科	143-0027	東京都大田区中馬込2-7-10	03-6410-7196	https://www.ogawa-naika.net/	○	○	内科<br>小児科	小川玄洋	
835	西蒲田整形外科	146-0094	東京都大田区東矢口3-2-1-108	03-3735-2117	https://www.nishikamata.jp		○	整形外科	網野　浩	
836	西馬込あくつ耳鼻咽喉科	143-0025	東京都大田区南馬込5-40-1西馬込メディカルビレッジ3階	03-6417-1855	https://www.magojibi.jp/	○	○	耳鼻咽喉科<br>アレルギー科<br>小児科	阿久津　征利	大森赤十字病院（東京都大田区中央4-30-1）<br>荏原病院（東京都大田区東雪谷4-5-10）
837	脳外科西馬込クリニック	143-0026	東京都大田区西馬込2-2-5	03-6303-7070	https://nishi-magome.com/	○	○	脳神経外科	徳永　仁	

電話や情報通信機器を用いて診療を実施する医療機関の一覧（東京都）

	基本情報					事務連絡に基づく対応について				
	施設名	郵便番号	住所（都道府県から記載）	電話番号	ウェブサイトURL	初診の電話等を用いた診療の実施の有無	再診の電話等を用いた診療の実施の有無	対応診療科	担当医師名	対面診療が必要と判断した場合に連携する医療機関名（複数ある場合は複数、住所も併せて記載）
838	萩中ハートフルクリニック	1440047	東京都大田区萩中1-9-16	03-3745-0810	http://www.haginaka-heart.com	○	○	内科	村井　治	
839	原口小児科クリニック	144-0032	東京都大田区北糀谷1-11-8　アルトT1F	03-3742-1517	https://www.haraguchi-clinic.com/		○	小児科<br>アレルギー科	原口　道夫	
840	東馬込しば整形外科	143-0022	東京都大田区東馬込東馬込1-19.-3	03-5718-8811	http://shiba-seikei.jp/		○	整形外科	柴　伸昌	
841	ファミリック診療所	146-0092	東京都大田区下丸子2ー13ー4	03-5741-3717	http://www.familic.com	○	○	内科・小児科・呼吸器内科	山口　久美子	東邦大学大森病院
842	藤川クリニック	146-0083	東京都大田区千鳥３－７－２	03-5732-3200	https://www.fujikawa-cl.jp/		○	内科<br>消化器内科<br>肛門外科<br>外科	藤川　亨	池上総合病院（東京都大田区池上6-1-19）<br>荏原病院（東京都大田区東雪谷4-5-10）<br>大森赤十字病院（東京都大田区中央4-30-1）<br>東邦大学医療センター大森病院（東京都大田区大森西6-11-1）
843	ふじみクリニック	143-0015	東京都大田区大森西3-1-38　マチノマ大森3階	03-5493-5252	https://fujimi-ped.jp/	○	○	小児科<br>アレルギー科<br>腫瘍内科	河本　博<br>村上　直子	
844	平和島医院	143-0016	東京都大田区大森北6-14-10	03-5753-5050		○	○	内科<br>小児科	森本　幸治	
845	ほりクリニック	144-0051	東京都大田区西蒲田7-12-6　1階	03-3731-3108	https://www.horiclinic.org/	○	○	耳鼻咽喉科	堀　雅明	東邦大学医療センター大森病院（〒143-8541 東京都大田区大森西6-11-1）
846	本田英比古クリニック	143-0023	東京都大田区山王3-17-11	03-5718-0077	http://www.honda-hidehiko-clinic.com		○	内科<br>神経内科	本田　英比古	

電話や情報通信機器を用いて診療を実施する医療機関の一覧（東京都）

	基本情報					事務連絡に基づく対応について				
	施設名	郵便番号	住所（都道府県から記載）	電話番号	ウェブサイトURL	初診の電話等を用いた診療の実施の有無	再診の電話等を用いた診療の実施の有無	対応診療科	担当医師名	対面診療が必要と判断した場合に連携する医療機関名（複数ある場合は複数、住所も併せて記載）
847	増田外科	144-0053	東京都大田区蒲田本町1-4-8	03-3732-0877	http://masuda-geka.jp/	○	○	内科<br>外科<br>皮膚科	武田 明芳<br>武田 朋子	池上総合病院（大田区池上6-1-19）<br>大森赤十字病院（大田区中央4 -30-1）<br>東邦大学大森病院（大田区大森西6-11-1）
848	南馬込おかばやし耳鼻咽喉科	143-0025	東京都大田区南馬込6-4-23 ファミネス梅田１F	03-6417-1368	https://www.okabayashi-jibika.com/	○	○	耳鼻咽喉科	岡林　大	昭和大学病院(東京都品川区旗の台1-5-8)<br>東邦大学医療センター大森病院(東京都大田区大森西6-11-1)
849	みやはら内科外科クリニック	144-0055	東京都大田区仲六郷３－５－２	03-6424-9838		○	○	内科<br>外科	宮原　正樹	
850	夫婦坂クリニック	145-0064	東京都大田区上池台4丁目21-7 ウエルシア2階	03-6421-8755	https://meotozaka-clinic.com/	○	○	内科/アレルギー科/皮膚科/泌尿器科		昭和大学病院（東京都品川区旗の台1-5-8）<br>荏原病院（東京都大田区東雪谷四丁目5-10）<br>大森赤十字病院（東京都大田区中央4 丁目30 番1号）
851	目蒲病院	146-0092	東京都大田区下丸子3-23-3	03-3759-8888	http://www.myclinic.ne.jp/mekama/pc/…		○	内科・整形外科	平原・妻鳥	対面診療の対応が不可能な場合があります
852	安田整形外科	144-0052	東京都大田区蒲田4-18-10	03-3734-2761		○	○	整形外科	安田　東<br>金井　ゆりか	
853	やすだ内科クリニック	145-0065	東京都大田区東雪谷2-25-12フローディア雪谷101	03-3726-0660	https://www.yasuda-cl.jp		○	内科	安田　大二郎	
854	横川医院	144-0034	東京都大田区西糀谷4-9-24	03-3741-5567			○	内科<br>小児科	横川　敏男	
855	ヨコヤマクリニック	144-0055	東京都大田区仲六郷1-56-10	03-3733-4771	http://www.clinic-yokoyama.jp		○	内科<br>眼科	横山　真也<br>吉川　典子	

電話や情報通信機器を用いて診療を実施する医療機関の一覧（東京都）

	基本情報					事務連絡に基づく対応について				
	施設名	郵便番号	住所（都道府県から記載）	電話番号	ウェブサイトURL	初診の電話等を用いた診療の実施の有無	再診の電話等を用いた診療の実施の有無	対応診療科	担当医師名	対面診療が必要と判断した場合に連携する医療機関名（複数ある場合は複数、住所も併せて記載）
856	吉田ハートクリニック	144-0051	東京都大田区西蒲田7－9－3－101	03-3731-2302			○	循環器内科<br>内科	吉田　哲	
857	若草クリニック	146-0095	東京都大田区多摩川1-26-21	03-3759-6325		○	○	内科	太田　斉<br>太田　朝子	池上総合病院（東京都大田区池上6-1-19）<br>牧田総合病院（東京都大田区大森北1-34-6）
858	池上仲通りクリニック	146-0082	東京都大田区池上3-32-17-1F	03-5747-6161	http://www.daizem.jp/	○	○	内科	奈良　大	
859	蒲田小児科医院	144-0052	東京都大田区蒲田3－15－18	03-3738-7084		○	○	小児科	高橋　礼子	
860	山縣医院	144-0043	東京都大田区羽田1-6-14	03-3741-1981		○		内科<br>小児科	山縣　法子	
861	東邦大学羽田空港クリニック	144-0041	東京都大田区羽田空港3-4-2第二ターミナル1階	03-6428-8121			○	内科	籾山　浩一<br>大道　泰子	左記施設
862	難波メディカルクリニック	146-0092	東京都大田区下丸子二丁目１３番４号　東京サーハウス共同棟１階	03-5741-3717	https://nanba-medical.com/		○	内科<br>小児科	難波雄亮	都立荏原病院
863	アイリス眼科	157-0066	東京都世田谷区成城6－16－9　リズリ成城一階	03-3483-7373	https://www.iris-ganka.jp/		○	眼科	地場　奈実	
864	あきら内科	158-0097	東京都世田谷区用賀4-3-9	03-3708-6777			○	内科	佐田　昌	関東中央病院（東京都世田谷区上用賀６－２５－１）

電話や情報通信機器を用いて診療を実施する医療機関の一覧（東京都）

	基本情報					事務連絡に基づく対応について				
	施設名	郵便番号	住所（都道府県から記載）	電話番号	ウェブサイトURL	初診の電話等を用いた診療の実施の有無	再診の電話等を用いた診療の実施の有無	対応診療科	担当医師名	対面診療が必要と判断した場合に連携する医療機関名（複数ある場合は複数、住所も併せて記載）
865	浅香耳鼻咽喉科クリニック	158-0093	東京都世田谷区上野毛1丁目19-1　メゾン上野毛1F	03-3703-8733	http：//asaka-ent.com	○	○	耳鼻咽喉科	浅香　大也<br>齋藤　優二<br>横井　佑一郎	東邦大学医療センター大橋病院（東京都目黒区大橋2-22-36）<br>東京共済病院（東京都目黒区中目黒2-3-8）
866	雨宮内科	158-0081	東京都世田谷区深沢6-30-13	03-3701-5920			○	内科	雨宮	東京医療センター
867	アレックス脊椎クリニック	158-0082	東京都世田谷区等々力4-13-1	03-5758-1321	https://ar-ex.jp/spine		○	整形外科	吉原　潔<br>石塚　怜王<br>江口　直	
868	淡島こどもクリニック	155-0032	東京都世田谷区代沢5-18-1　代沢カラバッシュ２F	03-3422-9751	http://www.awashima-cl.jp	○	○	小児科<br>アレルギー科	武隈　孝治<br>野呂　恵子<br>藤谷　しのぶ	
869	医療法人社団こしだ内科クリニック	156-0054	東京都世田谷区桜丘2-27-11ﾚﾌｨｰ/KMビル1階	03-3425-1771	mail@koshida-naika.tokyo	○	○	内科	越田容子	
870	医）修勇会田崎胃腸科内科	158-0081	東京都世田谷区深沢6-2-17-1,2F	03-3703-4133	https://www.tazakiichokanaika.com/		○	内科,胃腸内科,消化器内科,循環器内科,呼吸器内科	田﨑修平	玉川病院（東京都世田谷区）<br>関東中央病院（東京都世田谷区）<br>自衛隊中央病院（東京都世田谷区）<br>東京医療センター（東京都目黒区）
871	AR-Ex尾山台整形外科	158-0082	東京都世田谷区等々力4-13-1	03-5758-3007	https://ar-ex.jp/oyamadai/		○	整形外科	綿貫　誠<br>平田　正純<br>久保　貴敬<br>清水　勇樹<br>大野　達朗<br>森　祐佑<br>内田　宗志<br>平沼　憲治	国立病院機構東京医療センター（東京都目黒区東が丘2-5-1）<br>日本医科大学武蔵小杉病院（神奈川県川崎市中原区小杉町1-396）
872	いなみ内科クリニック	154-0024	東京都世田谷区三軒茶屋１－１３－７<br>三茶JOYビル２F	03-3413-1730	http://inami-naika-clinic.com/		○	内科<br>消化器内科<br>肝臓内科	稲見　真木子<br>川島　志布子	東京医療センター（東京都目黒区東が丘2-5-1）<br>東邦大学医療センター大橋病院(東京都目黒区大橋2-22-36）<br>三宿病院（東京都目黒区上目黒5-33-12）<br>自衛隊中央病院（東京都世田谷区池尻1-2-24）
873	五十子クリニック	156-0052	東京都世田谷区経堂5-3-29	03-3426-0220	https://www.irakoclinic.com	○	○	内科	五十子　大雅	

電話や情報通信機器を用いて診療を実施する医療機関の一覧（東京都）

	基本情報					事務連絡に基づく対応について				
	施設名	郵便番号	住所（都道府県から記載）	電話番号	ウェブサイトURL	初診の電話等を用いた診療の実施の有無	再診の電話等を用いた診療の実施の有無	対応診療科	担当医師名	対面診療が必要と判断した場合に連携する医療機関名（複数ある場合は複数、住所も併せて記載）
874	(医)慈桜会 渡辺クリニック	156-0054	東京都世田谷区桜丘2-5-23	03-5426-8624	https://www.watanabeclin.com		○	内科<br>小児科	渡邉　浩	
875	医療法人社団<br>いしかわキッズクリニック	154-0017	東京都世田谷区世田谷2-6-11	03-5451-3014	http://www.ishikawa-kids.com/	○	○	小児科	石川　央朗	
876	医療法人社団英寿会<br>深沢クリニック	154-0016	東京都世田谷区弦巻2－39－3	03-3426-8253	https://fukazawa-clinic.or.jp	○	○	内科	深沢祐之	
877	医療法人社団家族の木<br>給田ファミリークリニック	157-0064	東京都世田谷区給田3-26-6	03-5315-5511	https://kyuden-clinic.com/	○	○	内科<br>小児科	池亀康夫<br>樫尾明彦	
878	医療法人社団九折会成城<br>木下病院	157-0066	東京都世田谷区成城6-13-20	03-3482-1799	http://www.kinohosp.com/	○	○	婦人科<br>内科<br>小児科	木下勝之<br>木下二宣<br>木下智恵<br>鳥巣弘道<br>齋木美恵<br>山ノ井睦<br>山田由季<br>竹本由美	
879	医療法人社団クレッセント<br>つだ小児科クリニック	154-0017	東京都世田谷区世田谷4－5－8<br>アルス世田谷ネクステージ1F	03-5477-7736	https://www.tsudashonika.com	○	○	小児科	津田正彦	
880	医療法人社団慶永会　きたみ胃･大腸クリニック	156-0067	東京都世田谷区喜多見8-18-10　小泉ビル3F	03-5494-8411	https://kitami-clinic.com		○	消化器内科	西堀英樹，高田厚，渡邉一宏	
881	医療法人社団健央会　健内科クリニック	158-0082	東京都世田谷区等々力4-9-1	03-6809-8171	http://www.ken-cli.jp		○	内科	深井　健一	
882	医療法人社団慈正会<br>副島クリニック	155-0032	東京都世田谷区代沢5-3-8	03-3421-1793		○	○	内科<br>小児科	副島　道正	

電話や情報通信機器を用いて診療を実施する医療機関の一覧（東京都）

	基本情報					事務連絡に基づく対応について				
	施設名	郵便番号	住所（都道府県から記載）	電話番号	ウェブサイトURL	初診の電話等を用いた診療の実施の有無	再診の電話等を用いた診療の実施の有無	対応診療科	担当医師名	対面診療が必要と判断した場合に連携する医療機関名（複数ある場合は複数、住所も併せて記載）
883	医療法人社団周駒会<br>井上外科・内科	154-0024	東京都世田谷区三軒茶屋2-24-17	03-3424-3360	https://www.inoue-geka.net	○	○	内科<br>小児科	井上敬一郎	当院に来院
884	医療法人社団 潤愛会<br>板谷クリニック	158-0083	東京都世田谷区奥沢2-10-12	03-3723-0655	https://www.itaya-cl.jp		○	内科	板谷光慶	
885	医療法人社団翔未会<br>桜新町クリニック	154-0016	東京都世田谷区桜新町1-7-6	03-5706-4321	http://www.shomikai.or.jp/saku/	○	○	内科<br>腎臓内科<br>呼吸器科	堀切　つぐみ<br>外丸　良	
886	医療法人社団頌和会<br>村田医院	154-0021	東京都世田谷区豪徳寺2ー16ー25	03-3425-2612	murata-clinic.net	○	○	内科<br>小児科	村田昌隆	
887	医療法人社団）白木会<br>木下皮フ科	03-3718-9907	東京都世田谷区奥沢5-20-1	03-3718-9907	http://www.kinoshita-clinic.com		○	皮フ科<br>アレルギー科	木下　三和子	
888	医療法人社団仁利会こうらクリニック	154-0021	東京都世田谷区豪徳寺1-23-22	03-3425-2333	https://www.koura-clinic.com	○	○	内科	兒浦利哉	
889	医療法人社団誠清会<br>駒沢皮膚科クリニック	154-0011	東京都世田谷区上馬3-18-11　エルフレア３Ｆ	03-3413-6600	https://komazawahifuka.com/		○	皮膚科	清水　顕	
890	医療法人社団<br>瀬田診療所	158-0093	東京都世田谷区上野毛4-24-15	03-3700-4369	http://seta-med.com/access/index.html	○	○	内科<br>小児科	大橋　美奈子<br>大橋　十也	国立病院機構東京医療センター（東京都目黒区東が丘2-5-1）<br>日産厚生会玉川病院（世田谷区瀬田4-8-1）<br>慈恵医大第三病院
891	医療法人社団創福会<br>ふくろうクリニック等々力	153-0082	東京都世田谷区等々力3-5-2<br>ヒューリック等々力ビル3階	03-5758-3270	http://www.296296.jp	○	○	内科	山口　潔<br>秋好　沢諭<br>片本　行信<br>橋本　昌也<br>吉田　哲彦	

電話や情報通信機器を用いて診療を実施する医療機関の一覧（東京都）

	基本情報					事務連絡に基づく対応について				
	施設名	郵便番号	住所（都道府県から記載）	電話番号	ウェブサイトURL	初診の電話等を用いた診療の実施の有無	再診の電話等を用いた診療の実施の有無	対応診療科	担当医師名	対面診療が必要と判断した場合に連携する医療機関名（複数ある場合は複数、住所も併せて記載）
892	医療法人社団宝池会<br>吉川内科医院	156-0043	東京都世田谷区松原3-28-8	03-3323-0661	https://yoshikawa.or.jp		○	内科	吉川昌男<br>吉川尚男<br>久次米真吾<br>岩崎昌樹	
893	医療法人社団治成会<br>シグマクリニック	154-0002	東京都世田谷区下馬5-41-21	0120-978-922	https://sigma.or.jp/	○	○	内科<br>婦人科<br>甲状腺科<br>乳腺科	田中　弦<br>伊藤　路奈<br>福成　信博<br>上野　貴史	
894	医療法人社団徹友会<br>岸田こどもクリニック	154-0001	東京都世田谷区池尻３－２１－２－２F	03-6450-9655	http://www.kishidakodomo.com/	○	○	小児科	岸田勝	東邦大学医療センター大橋病院<br>（東京都目黒区大橋2-22-36）
895	医療法人社団土筆会<br>桜丘クリニック	156-0054	東京都世田谷区桜丘5-17-4 直井ビル1F	03-5426-2292	https://sakuragaoka-clinic.com/	○	○	内科<br>消化器内科	比嘉　晃二<br>難波　雄亮	関東中央病院（東京都世田谷区上用賀6丁目25-1）
896	医療法人社団なみいろの樹<br>なおこ皮膚科クリニック	158-0097	東京都世田谷区用賀4-9-27　第3福ビル2階	03-6805-7484	http://www.naoko-hifuka.com/	○	○	皮膚科<br>内科<br>小児科	服部　尚子<br>木村　久美子	
897	医療法人社団のびた<br>みくりキッズくりにっく	158-0093	東京都世田谷区上野毛2-22-14-B棟	03-3701-1010	https://micri.jp/	○	○	小児科<br>小児神経科<br>リハビリテーション科	本田<br>岡田<br>大塚	
898	医療法人社団プラタナス<br>桜新町アーバンクリニック	154-0014	東京都世田谷区新町3-21-1<br>さくらウェルガーデン2F	03-3429-1192	http://www.sakura-urban.jp	○	○	内科<br>婦人科<br>心療内科	田中　啓広<br>勝又　聡彦<br>圓井　佐江子<br>野澤　詠子	関東中央病院（東京都世田谷区上用賀６－２５－１）<br>玉川病院（東京都世田谷区瀬田４丁目８－１）<br>用賀アーバンクリニック（東京都世田谷区用賀2-41-17 1F2F）
899	医療法人社団プラタナス<br>松原アーバンクリニック	156-0043	東京都世田谷区松原５－３４－６	03-5355-3388	https://matsubara-urban.jp/	○	○	内科<br>消化器内科	梅田耕名<br>小澤 政成<br>山﨑 励至<br>橘髙 恵<br>佐伯 知行<br>白濱 淳、<br>舟槻 晋吾<br>石川 祐輔	用賀アーバンクリニック<br>桜新町アーバンクリニック
900	医療法人社団プラタナス<br>用賀アーバンクリニック	158-0097	東京都世田谷区用賀2-41-17 1F2F	03-5717-6331	https://yoga-urban.jp/	○	○	内科<br>小児科	野間口　聡<br>田中　勝巳<br>大石　大輔	関東中央病院（東京都世田谷区上用賀６－２５－１）<br>玉川病院（東京都世田谷区瀬田４丁目８－１）<br>桜新町アーバンクリニック（東京都世田谷区新町3-21-1 2F）

電話や情報通信機器を用いて診療を実施する医療機関の一覧（東京都）

	基本情報					事務連絡に基づく対応について				
	施設名	郵便番号	住所（都道府県から記載）	電話番号	ウェブサイトURL	初診の電話等を用いた診療の実施の有無	再診の電話等を用いた診療の実施の有無	対応診療科	担当医師名	対面診療が必要と判断した場合に連携する医療機関名（複数ある場合は複数、住所も併せて記載）
901	医療法人社団恵会<br>二宮こどもクリニック	158-0097	東京都世田谷区用賀4-12-15ビバーチェAB号	03-3709-1300	ninomiya-clinic.jp/		○	内科<br>小児科	二宮　淳一<br>二宮　惠子	
902	医療法人社団ラルゴ　さとう内科・脳神経クリニック	158-0083	東京都世田谷区奥沢2-4-10	03-5726-9995	satoh-nc.jp	○	○	内科<br>小児科<br>神経内科	佐藤　博紀<br>佐藤佳渚子	奥沢病院（東京都世田谷区奥沢2-11-11）<br>昭和大学病院（東京都品川区旗の台1-5-8）<br>荏原病院（東京都大田区東雪谷4-5-10）
903	医療法人社団琉音会<br>用賀耳鼻咽喉科	158-0097	東京都世田谷区用賀2-36-8-105	03-5491-5253	http://ssc.doctorqube.com/ryuonkai/	○	○	耳鼻咽喉科	高橋　幸太郎	
904	医療法人社団リバイブ吉野クリニック	157-0062	東京都世田谷区南烏山5-1-15ﾋｴﾓﾄﾋﾞﾙ102	03-5315-7170		○	○	内科	吉野　博子	
905	医療法人社団 le bois<br>あんどうファミリークリニック	157-0073	東京都世田谷区砧8－21－8　シェーンハイト1F2F	03-5727-5500	http: //www.andyman.jp	○	○	内科<br>小児科	安藤　秀彦	
906	上杉医院	158-0091	東京都世田谷区中町1-19-19	03-5706-0777	www.u-clinic.min.gr.jp		○	内科<br>小児科	上杉　秀永	
907	うたクリニック<br>内科消化器内科	158-0093	東京都世田谷区上野毛1-30-22	03-6382-8111	http://uta-clinic.com/	○	○	内科<br>消化器内科	宇多　慶記	玉川病院（東京都世田谷区瀬田4-8-1）<br>東京医療センター（東京都目黒区東が丘2-5-1）
908	オークラ小児科	158-0082	東京都世田谷区等々力2-33-11	03-3701-5360		○	○	小児科	大倉節子	東京医療センター（東京都目黒区東が丘2-5-1）<br>国立成育医療センター（東京都世田谷区大蔵２丁目１０－１）
909	岡崎内科クリニック	158-0081	東京都世田谷区深沢５－３６－１８	03-5758-3680		○	○	内科	岡崎　怜子	東京医療センター（東京都目黒区東が丘2-5-1）

電話や情報通信機器を用いて診療を実施する医療機関の一覧（東京都）

	基本情報					事務連絡に基づく対応について				
	施設名	郵便番号	住所（都道府県から記載）	電話番号	ウェブサイトURL	初診の電話等を用いた診療の実施の有無	再診の電話等を用いた診療の実施の有無	対応診療科	担当医師名	対面診療が必要と判断した場合に連携する医療機関名（複数ある場合は複数、住所も併せて記載）
910	おくさわキッズクリニック	158-0083	東京都世田谷区奥沢３－３０－１７-1F	03-6425-4970	http://hwm6.gyao.ne.jp/okc330171f/	○	○	小児科<br>アレルギー科	藤井芳実	
911	尾山台かばやま小児科	158-0082	東京都世田谷区等々力4-1-15-2F	03-5758-1147		○	○	小児科	樺山　浩彦	国立成育医療センター（東京都世田谷区大蔵２丁目１０－１）
912	尾山台ゆあさ内科クリニック	158-0082	東京都世田谷区等々力5-5-12-2階	03-3704-1024	http://yuasa-clinic.jp		○	内科	湯浅　幸子<br>湯浅　慎介	
913	かどた内科クリニック	155-0032	東京都世田谷区代沢5-18-1-2F	03-6413-8333	https://kad-cl.com/		○	内科<br>呼吸器内科<br>アレルギー科	門田　篤	
914	上野毛眼科	158-0093	東京都世田谷区上野毛3ー4ー17	03-5752-4111			○	眼科	鎌田絵里子<br>鎌田裕子	
915	うすだ内科クリニック	156-0043	東京都世田谷松原2-29-1　ブランテラス松原201	03-3323-7707	https://usuda-naika.com	○	○	内科<br>脳神経内科	臼田　和弘	
916	上祖師谷かたらいクリニック	157-0065	東京都世田谷区上祖師谷6-7-28　かたらいビル1階	03-3300-6006	http://www.katarai-clinic.jp	○	○	内科	鈴木 淳	
917	唐沢内科医院	158-0081	東京都世田谷区深沢１－１８－９　１F	03-3701-7552	http://www.karasawanaika.jp	○	○	内科<br>小児科	唐澤　達信	国立病院機構東京医療センター（東京都目黒区東が丘２－５－１）
918	かるがもクリニック	156-0054	東京都世田谷区桜丘2ー25ー4	03-5426-2220	https://www.karugamo-cl.jp/	○	○	小児科・内科	宮原　篤	関東中央病院（東京都世田谷区上用賀6-25-1）・成育医療センター（東京都世田谷区大蔵2ー10ー1）

電話や情報通信機器を用いて診療を実施する医療機関の一覧（東京都）

	基本情報					事務連絡に基づく対応について				
	施設名	郵便番号	住所（都道府県から記載）	電話番号	ウェブサイトURL	初診の電話等を用いた診療の実施の有無	再診の電話等を用いた診療の実施の有無	対応診療科	担当医師名	対面診療が必要と判断した場合に連携する医療機関名（複数ある場合は複数、住所も併せて記載）
919	河崎内科クリニック	158-0081	東京都世田谷区深沢2-13-21	03-5707-9520	http://www.newton-doctor.com/doctor/tokyo14/fukasawakawasaki/s01/	○	○	内科	河崎恒久	東京医療センター（東京都目黒区東が丘2-5-1）<br>関東中央病院（東京都世田谷区上用賀6-25-1）<br>奥沢病院（東京都世田谷区奥沢2-11-11）<br>駒沢病院（東京都世田谷区駒沢2-2-15）<br>田園調布中央病院（東京都大田区田園調布2-43-1）<br>日赤医療センター（東京都渋谷区広尾4-1-22）<br>玉川病院（東京都世田谷区瀬田4-8-1）
920	かわまたこどもクリニック	156-0042	東京都世田谷区羽根木2-40-23-1F	03-3321-3077	http://www.kawamata-kodomo.com	○	○	小児科	川真田　光	
921	喜多見こどもクリニック	157-0067	東京都世田谷区喜多見８－１９－１５　森田ビル2階	03-5727-1033	http://www.57271033.com/i/		○	小児科	上田　哲	
922	喜多見やの耳鼻科	157-0067	東京都世田谷区喜多見8丁目１８－１２	03-6411-0322	http://kitamiyanojibika.com/	○	○	耳鼻咽喉科	矢野　裕之	多摩病院<br>慈恵医大第三病院
923	樹のはなクリニック	154-0004	東京都世田谷区太子堂2-26-2　サルース太子橋1階	03-5433-3388	kinohana-clinic.com	○	○	内科	奈良岡　美惠子	東邦大学医療センター大橋病院・関東中央病院
924	経堂こうづき眼科	156-0063	東京都世田谷区経堂２丁目1-３３経堂コルティ2F	03-5799-7276	https://www.kozuki-eyeclinic.com/	○	○	眼科	上月　直之	
925	経堂3丁目クリニック	156-0052	東京都世田谷区経堂3-20-22	03-5799-7027	http://houmonshinryou.com/		○	内科	日吉　晴久	
926	樺クリニック	154-0016	東京都世田谷区弦巻4-2-18　1F	03-5451-7211			○	内科	樺　恵	
927	くろさか皮膚科クリニック	158-0083	東京都世田谷区等々力6-2-1	03-5758-6789	http://www.kurosaka-clinic.com/		○	皮膚科	黒坂　良枝	

電話や情報通信機器を用いて診療を実施する医療機関の一覧（東京都）

	基本情報					事務連絡に基づく対応について				
	施設名	郵便番号	住所（都道府県から記載）	電話番号	ウェブサイトURL	初診の電話等を用いた診療の実施の有無	再診の電話等を用いた診療の実施の有無	対応診療科	担当医師名	対面診療が必要と判断した場合に連携する医療機関名（複数ある場合は複数、住所も併せて記載）
928	こころの診療所三軒茶屋	154-0024	東京都世田谷区三軒茶屋1-38-8 3階	03-6805-5510	https://kokoro-sancha.com/	○	○	心療内科<br>精神科	北條　彩	
929	こじま内科呼吸器科	158-0083	東京都世田谷区奥沢2丁目38番9　自由が丘月瀬ビル3階	03-3725-1159	http://kojimanaika.tokyo/index.html		○	内科<br>呼吸器内科	小島　薫	
930	こだいら泌尿器科	154-0003	東京都世田谷区野沢3-2-8-101	03-3410-2373	www.kodairaurology.jp	○	○	泌尿器科	古平　喜一郎	東京医療センター（東京都目黒区東が丘2-5-1）
931	駒沢風の診療所	154-0011	東京都世田谷区上馬４－４－１０	03-5712-0012		○	○	内科<br>消化器科<br>リウマチ科	斉藤　栄造<br>菊池　真大<br>中里　真宏<br>菊池　美穂	
932	駒沢公園内科クリニック	154-0012	東京都世田谷区駒沢４－１－１ノア駒沢1階	03-5779-6603	https://kp-clinic.com/		○	内科<br>神経内科<br>消化器内科	坂本　剛<br>坂本　夏子	
933	小松内科クリニック	158-0083	東京都世田谷区奥沢５－２５－１１	03-3717-7772	http://www.komatsu-naika.com	○	○	内科<br>小児科	小松　英嗣	
934	さいとうクリニック	154-0014	東京都世田谷区新町2-35-20	03-3425-1200			○	内科	斉藤	玉川病院　世田谷区瀬田4-8-1<br>関東中央病院　世田谷区上用賀6-25-1
935	斎藤小児科内科医院	156-0044	東京都世田谷区赤堤1-39-15	03-3325-1177		○	○	小児科<br>内科	斎藤正敏	関東中央病院（東京都世田谷区）<br>東邦大学医療センター大橋病院（目黒区大橋）<br>柴本内科医院（東京都世田谷区）
936	坂谷クリニック		世田谷区用賀4-10-3 ヒルズII 1F	03-5717-2255		○	○	消化器内科<br>内科	坂谷　新<br>坂谷　敬子	

電話や情報通信機器を用いて診療を実施する医療機関の一覧（東京都）

	基本情報					事務連絡に基づく対応について				
	施設名	郵便番号	住所（都道府県から記載）	電話番号	ウェブサイトURL	初診の電話等を用いた診療の実施の有無	再診の電話等を用いた診療の実施の有無	対応診療科	担当医師名	対面診療が必要と判断した場合に連携する医療機関名（複数ある場合は複数、住所も併せて記載）
937	桜新町こどもクリニック	154-0014	東京都世田谷区新町3-2-1	03-5450-1515	http://www.sakura-kids-clinic.jp/	○	○	内科、小児科、皮膚科、アレルギー科	似鳥　嘉一	東邦大学大橋病院（目黒区大橋2-17-6）東京医療センター（目黒区東が丘2-5-1）成育医療研究センター（世田谷区大蔵2-10-1）
938	さくらキッズくりにっく	154-0015	東京都世田谷区桜新町２－１０－４　ミケア　２Ｆ	03-5451-0016	http//www.sakurakids-clinic.com	○	○	小児科	藤井　明子	国立成育医療研究センター（東京都世田谷区大蔵２丁目１０－１）
939	さくら小児科・内科クリニック	156-0053	東京都世田谷区桜1-67-8 AKビル１F	03-6413-2211	sakura-kids.com	○	○	小児科内科	立山悟志<br>中島夏樹	国立成育衣料研究センター　関東中央病院
940	佐藤診療所	158-0077	東京都世田谷区用賀2-35-4	03-5491-7745	www.sato-shinryojo.com	○	○	内科<br>小児科	佐藤　周三	東京医療センター（東京都目黒区東が丘2-5-1）
941	三軒茶屋駅前おなかクリニック	154-0024	東京都世田谷区三軒茶屋１－３７－２　三茶ビル4階	03-3421-7149	https://sancha-cl.com	○	○	内科　消化器内科　内視鏡内科	三谷　年史	
942	三軒茶屋駅前<br>メンタルクリニック	154-0024	東京都世田谷区三軒茶屋1-37-8<br>ワコーレ三軒茶屋64ビル5階A	03-6804-0126	http://www.sancha-mental.com	○	○	精神科<br>心療内科	服部　栄治	
943	SANCHAこころのクリニック	154-0004	東京都世田谷区太子堂2-14-3シャルムイシクラ4F	03-5431-0258	https://sanchakokoro.com/	○	○	精神科<br>心療内科	西脇　一郎	
944	自衛隊中央病院	154-8532	東京都世田谷区池尻１－２－２４	03-3411-0151	http://www.mod.go.jp/gsdf/chosp/index..html		○	精神科、病理診断科、救急科を除く診療科	左記診療科を除く全ての医師	
945	志賀医院	155-0031	東京都世田谷区北沢2-29-16	03-3460-3552		○	○	内科<br>小児科	志賀　幸雄	東邦大学医療センター大橋病院<br>（東京都目黒区大橋2-17-6）

電話や情報通信機器を用いて診療を実施する医療機関の一覧（東京都）

	基本情報					事務連絡に基づく対応について				
	施設名	郵便番号	住所（都道府県から記載）	電話番号	ウェブサイトURL	初診の電話等を用いた診療の実施の有無	再診の電話等を用いた診療の実施の有無	対応診療科	担当医師名	対面診療が必要と判断した場合に連携する医療機関名（複数ある場合は複数、住所も併せて記載）
946	耳鼻咽喉科弘重クリニック	157-0067	東京都世田谷区喜多見8-19-14 喜多見フォーラム2-4階	03-5494-6565		○	○	耳鼻科	弘重　光一<br>弘重　哉子	東京慈恵会医大第三病院（東京都狛江市和泉本町4-11-1）　関東中央病院（東京都世田谷区上用賀6-25-1）
947	自由が丘南口クリニック	158-0083	東京都世田谷区奥沢6-21-11	03-3718-7429			○	内科<br>皮膚科	稲本元<br>稲本伸子	慶應義塾大学病院<br>独立行政法人国立病院機構東京医療センター<br>北里大学北里研究所病院
948	清水小児科医院	154-0011	東京都世田谷区上馬4-27-7	03-3421-5696		○	○	内科<br>小児科	清水　寛子<br>清水　千鶴<br>清水　浩信	独立行政法人国立病院機構東京医療センター（東京都目黒区東が丘2-5-1）<br>独立行政法人国立成育医療研究センター（東京都世田谷区大蔵2-10-1）
949	シモキタクリニック	155-0031	東京都世田谷区北沢2-17-2	03-3414-5252	http://shimokita-clinic.com/		○	内科	渡邉 友美	
950	下田クリニック	158-0082	東京都世田谷区等々力3-10-3-201	03-6411-6937		○	○	内科	下田泰彦	玉川病院（世田谷区瀬田4-8-1）
951	自由が丘ファミリー皮ふ科	158-0083	東京都世田谷奥沢５－２３－１８メディス自由が丘４階	03-6421-3412	http://jiyugaoka-family-hifuka.com/	○	○	皮膚科	玉城有紀	近隣のクリニック
952	せきぐちクリニック	156-0055	東京都世田谷区船橋1－16－1	03-3429-4181	https://www.sekiguchi-clinic.net	○	○	内科<br>小児科<br>婦人科	関口　茂	
953	せたがや上町脳神経クリニック	154-0012	東京都世田谷区世田谷2-4-2-4F	03-5477-2020	https://setagayakamimachineuro.com/		○	脳神経外科	高見　昌明	
954	世田谷記念病院	158-0092	東京都世田谷区野毛2丁目30－10	03-3703-5100	http://www.setagayahp.jp/	○	○	脳神経外科<br>内科	脳外：村上　秀喜　内科：木村　加奈子、志村　陽子、前田　朝美、横室　浩樹	

電話や情報通信機器を用いて診療を実施する医療機関の一覧（東京都）

	基本情報					事務連絡に基づく対応について				
	施設名	郵便番号	住所（都道府県から記載）	電話番号	ウェブサイトURL	初診の電話等を用いた診療の実施の有無	再診の電話等を用いた診療の実施の有無	対応診療科	担当医師名	対面診療が必要と判断した場合に連携する医療機関名（複数ある場合は複数、住所も併せて記載）
955	世田谷子どもクリニック	154-0017	東京都世田谷区世田谷3-6-14-101	03-5799-1582	https://www.setagayakodomo-clinic.com	○	○	小児科	副田敦裕	
956	世田谷下田総合病院	157-0062	東京都世田谷区南烏山4-9-23	03-3308-5221	http://www.shimodabyouin.jp/	○	○	内科<br>整形外科	下田重人<br>平田剛士<br>中川雅之<br>原田遼	
957	世田谷中央病院	154-0017	東京都世田谷区世田谷1-32-18	03-3420-7111	http://www.setagaya-hp.or.jp		○	内科<br>整形外科<br>外科	米満　祐一<br>小林　俊行<br>吉野　正晃	
958	せたがや内科・消化器クリニック	154-0015	東京都世田谷区桜新町1-40-8桜新町クリニックモール3階	03-3428-1107	https://tokyo-onaka.com	○	○	内科	富沢賢治	虎の門病院（東京都）　虎の門病院分院（神奈川県）
959	せたがや泌尿器腎クリニック	154-0015	東京都世田谷区桜新町1-40-8<br>桜新町クリニックモール２Ｆ	03-3425-1124	setagaya-uro.jp		○	泌尿器科<br>内科	中村圭輔	関東中央病院（東京都世田谷区上用賀6-25-1）
960	世田谷リウマチ<br>膠原病クリニック	156-0052	東京都世田谷区経堂2-4-6	03-6413-4666	http://www.setagayariumachi.com		○	リウマチ科	吉田智彦<br>勝山直興<br>秋山陽一郎	
961	たけおクリニック	154-0004	東京都世田谷区太子堂4-22-7　森住ビル３階	03-5433-3256	https://www.takeo-clinic.com/	○	○	内科	竹尾浩紀<br>田中沙代子<br>堺崇	自衛隊中央病院（東京都世田谷区池尻１－２－２４）
962	テツダ耳鼻咽喉科	158-0081	東京都世田谷区深沢5-23-19　1階	03-5760-3550	https://www.tetsudaent.com/	○	○	耳鼻咽喉科	鐵田　晃久	
963	東京都立松沢病院	156-0057	東京都世田谷区上北沢2-1-1	03-3303-7211	https://www.byouin.metro.tokyo.lg.jp/matsuzawa/		○	精神科、内科、外科、整形外科、形成外科、脳神経外科、リハビリテーション科、麻酔科、眼科、歯科等	外来各担当医	－

電話や情報通信機器を用いて診療を実施する医療機関の一覧（東京都）

	基本情報					事務連絡に基づく対応について				
	施設名	郵便番号	住所（都道府県から記載）	電話番号	ウェブサイトURL	初診の電話等を用いた診療の実施の有無	再診の電話等を用いた診療の実施の有無	対応診療科	担当医師名	対面診療が必要と判断した場合に連携する医療機関名（複数ある場合は複数、住所も併せて記載）
964	医療法人社団 慶永会 徳永整形外科	157-0067	東京都世田谷区喜多見8-18-10 小泉ビル2階	03-5494-7311	https://tokunaga.gr.jp		○	整形外科	徳永　祐二	
965	とも内科クリニック	154-0015	東京都世田谷区桜新町2-10-4ミケアビル２Ｆ	03-5451-7700	http://tomo.clinic	○	○	内科<br>糖尿病内科	加藤　朋子	東京医療センター（東京都目黒区東が丘2-5-1）<br>関東中央病院（東京都世田谷区上用賀6-25-1）<br>東邦大学大橋病院（東京都目黒区大橋2-22-36）<br>日産厚生会玉川病院（東京都世田谷区瀬田4-8-1）
966	ながたクリニック	154-0017	東京都世田谷区世田谷4-1-3世田谷医療COMMUNITY3F	03-5477-1128	http://www.nagataclinic.net/	○	○	内科<br>消化器内科<br>外科	永田　博康	
967	中野医院	1580086	東京都世田谷区尾山台3-7-13	03-3705-2551	http://www.nakano-iin.net/	○	○	産婦人科<br>内科<br>小児科	中野　明	玉川病院(世田谷区瀬田4-8-1)<br>関東中央病院(世田谷区上用賀6-25-1)
968	ニコタマ大腸・肛門クリニック	158-0094	東京都世田谷区玉川３－７－１　新二子玉川ビル１階・２階	03-3700-7777	https://nico-tama.jp		○	肛門外科<br>肛門内科<br>消化器内科	黒田　敏彦	奥沢病院（東京都世田谷区奥沢２丁目１１－１１）<br>田園調布中央病院（東京都大田区田園調布２丁目４３－１）<br>東京医療センター（東京都目黒区東が丘２丁目５－１）<br>東京大学医学部附属病院（東京都文京区本郷７丁目３－１）
969	西島内科クリニック	1570068	東京都世田谷区宇奈根３－１－２１	354942020	http://nishijima-clinic.jp		○	内科<br>小児科	西島敬之郎	
970	仁藤医院	158-0095	東京都世田谷区瀬田4-18-5	03-3700-6216	https://www.nito-iin.com		○	内科<br>皮膚科	仁藤　学<br>仁藤　真佐江	
971	野沢3丁目内科	154-0003	東京都世田谷区野沢3-1-16	03-5779-8255	https://www.nozawa3.jp/	○	○	内科	柳川達郎	
972	パークサイド脳神経外科クリニック	154-0004	東京都世田谷区太子堂1-3-39B1F	03-3414-3300	https://www.parkside-nsc.com		○	脳外科<br>神経内科	近藤　新	

電話や情報通信機器を用いて診療を実施する医療機関の一覧（東京都）

	基本情報					事務連絡に基づく対応について				
	施設名	郵便番号	住所（都道府県から記載）	電話番号	ウェブサイトURL	初診の電話等を用いた診療の実施の有無	再診の電話等を用いた診療の実施の有無	対応診療科	担当医師名	対面診療が必要と判断した場合に連携する医療機関名（複数ある場合は複数、住所も併せて記載）
973	浜本眼科内科	154-0012	東京都世田谷区駒沢4-19-13-201	03-5432-6302	https://www.hamamoto-eyeclinic.com/		○	内科	濱本真	
974	ひかりクリニック	154-0014	東京都世田谷区新町3-22-4	03-5426-7888			○	内科	松本　正廣<br>松本　裕子	関東中央病院（東京都世田谷区上用賀6-25-1）<br>日産厚生会玉川病院（東京都世田谷瀬田4-8-1）
975	ひらくクリニック	157-0065	東京都世田谷区上祖師谷5-18-9	03-3308-0900	http://hiraku-cl.com	○	○	内科	吉田　啓	
976	平山惠子産婦人科小児科クリニック	158-0083	東京都世田谷区奥沢6-3-7-202B	03-5758-3653		○	○	小児科<br>産婦人科	平山　惠子	奥沢病院（東京都世田谷区奥沢2-11-11）
977	ひろみ皮フ科クリニック	156-0051	東京都世田谷区宮坂2ー17ー2英月ビル1階	03-3420-7468	https://hiromi-clinic.jp/	○	○	皮膚科	梅澤裕美	関東中央病院（東京都世田谷区上用賀6-25-1）
978	深沢1丁目クリニック	158-0081	東京都世田谷区深沢1丁目クリニック	03-5758-8310	http://houmonshinryou.com/clinic/jukei-01/		○	内科	安西　光洋<br>鹿野　真実	
979	ふじいこどもクリニック	154-0017	東京都世田谷区世田谷4-1-3<br>世田谷医療COMMUNITY4階	03-5477-3377	http://www.fujii-kids.com/	○	○	小児科	藤井徹	
980	藤沢こどもクリニック	158-0082	東京都世田谷区等々力８－１７－１１	03-5758-3377	http://fcc.dcsv.jp	○	○	小児科	藤沢　康司	藤沢こどもクリニック（自院）
981	二子玉川ガーデン矯正歯科	158-0094	東京都世田谷区玉川3-20-1　名川ビル3階	03-5491-5454	https://www.hanarabi-kirei.com	○	○	矯正歯科	石野　善男	

電話や情報通信機器を用いて診療を実施する医療機関の一覧（東京都）

	基本情報					事務連絡に基づく対応について				
	施設名	郵便番号	住所（都道府県から記載）	電話番号	ウェブサイトURL	初診の電話等を用いた診療の実施の有無	再診の電話等を用いた診療の実施の有無	対応診療科	担当医師名	対面診療が必要と判断した場合に連携する医療機関名<br>（複数ある場合は複数、住所も併せて記載）
982	二子玉川ブレストクリニック	158-0094	東京都世田谷区玉川3-15-1 曽根ビル6階	03-3709-7717	http://www.futakotamagawa-bc.jp		○	乳腺外科・外科	浜口 洋平	
983	ほしの内科・アレルギークリニック	156-0053	東京都世田谷区桜3-2-17DS桜ビル2階	03-5426-6567	https://www.hoshialle.jp/		○	内科<br>小児科	星野　博	
984	牧野皮膚科	158-0081	東京都世田谷区深沢6-35-2	03-3703-5430	home.u05.itscom.net/dermakin/	○	○	皮膚科	牧野寒河江	
985	松尾内科クリニック	158-0097	東京都世田谷区用賀2-10-14 フォレスト446 1F	03-5717-7272	http://www.matsuo-naika.jp		○	内科	松尾　孝俊	関東中央病院（東京都世田谷区）
986	松原診療所	158-0091	東京都世田谷区中町2-37-19	03-3701-1233			○	内科<br>小児科	小池キャサリン<br>小池　満	独立行政法人国立病院機構東京医療センター（〒152-8902東京都目黒区東が丘2-5-1）<br>公立学校共済組合 関東中央病院（〒158-8531 東京都世田谷区上用賀６丁目２５−１）<br>国立成育医療研究センター（〒157-0074 東京都世田谷区大蔵２丁目１０−１）<br>日本赤十字社医療センター（〒150-8935 東京都渋谷区広尾４丁目１−２２）
987	マリア皮フ科クリニック	156-0053	東京都世田谷区桜3丁目2-17　DS桜ビル　4-D	03-5477-1212	https://maria-hifuka.jp/	○	○	皮膚科	平松　正浩	
988	丸山内科医院	158-0082	東京都世田谷区等々力８－６－３	03-3701-6457	http://maruyama-naika.jimdofree.com	○	○	内科	佐藤　明子	玉川病院　東京都世田谷区瀬田４－８－１
989	南烏山クリニック	157-0062	東京都世田谷区南烏山6-7-19グレイスビル１階	03-3309-1818	https://www.minami-k-clinic.com		○	内科	野口　祥代	
990	明大前整形外科クリニック	151-0043	東京都世田谷区松原1-38-25	03-6304-7681	https://ar-ex.jp/meidaimae		○	整形外科	成田哲也<br>久保貴敬<br>石塚怜王	

電話や情報通信機器を用いて診療を実施する医療機関の一覧（東京都）

	基本情報					事務連絡に基づく対応について				
	施設名	郵便番号	住所（都道府県から記載）	電話番号	ウェブサイトURL	初診の電話等を用いた診療の実施の有無	再診の電話等を用いた診療の実施の有無	対応診療科	担当医師名	対面診療が必要と判断した場合に連携する医療機関名（複数ある場合は複数、住所も併せて記載）
991	明大前皮フ科	156-0043	東京都世田谷区松原２－２９－１プランテラス松原202	03-6379-4156	www.meidaimae-hifuka.com/		○	皮膚科	新関寛徳	
992	目黒医院	158-0093	東京都世田谷区上野毛1-4-3	03-3702-5496	http://megurolin.net		○	内科<br>老年内科	目黒　和子	日産玉川病院（世田谷区瀬田4-8-1）<br>東京医療センター（目黒区東が丘2-5-1）<br>関東中央病院（世田谷区上用賀6-25-1）
993	望月眼科クリニック	158-0081	東京都世田谷区奥沢3-28-7	03-5754-4113	https://www.mochizuki-ganka.com		○	眼科	望月　弘嗣	
994	ゆうハートクリニック	155-0031	東京都世田谷区北沢2-1-16 アーバニティ下北沢2階	03-5432-9310	http://youheart.net	○	○	内科<br>循環器内科	鶴見　由起夫	
995	ヨシダ消化器内科クリニック	157-0062	東京都世田谷区南烏山6-6-2	03-5314-5552	http://www5.plala.or.jp/yoshida-cli/	○	○	内科<br>消化器科	吉田　泉	至誠会第二病院
996	若林ゆうクリニック	154-0023	東京都世田谷区若林5-14-6<br>若林ゆうビルディング2階	03-3410-1120	http://www.yu-cl.jp/		○	内科<br>泌尿器科<br>ペイン	五十嵐　裕恵<br>栗栖　寛子<br>五十嵐　一真<br>関島　千尋	
997	そしがや大蔵クリニック	157-0073	東京都世田谷区砧6-30-1 野原ビル1階	03-6411-1535	http://www.soshigayaokura-clinic.jp		○	リウマチ科<br>内科	中山久徳	
998	グレースホームケアクリニック	158-0083	東京都世田谷区奥沢3-35-14<br>島田マンション地下1階	03-6425-8858	http://www.grace-dc.com/st2/gracedc-homecare/	○	○	内科	古田　晃	
999	松村医院	158-0093	東京都世田谷区上野毛３－４－１６	03-3702-8358	https://www.matsumura-iin.com/		○	内科<br>小児科	松村　真司	

電話や情報通信機器を用いて診療を実施する医療機関の一覧（東京都）

	基本情報					事務連絡に基づく対応について				
	施設名	郵便番号	住所（都道府県から記載）	電話番号	ウェブサイトURL	初診の電話等を用いた診療の実施の有無	再診の電話等を用いた診療の実施の有無	対応診療科	担当医師名	対面診療が必要と判断した場合に連携する医療機関名（複数ある場合は複数、住所も併せて記載）
1000	みくに内科眼科クリニック	154-0002	東京都世田谷区下馬1-45-17清水ビル1階	03-3411-3920	https://mikuni.clinic/home		○	内科	三國盛夫	
1001	医療法人社団大南会<br>上野毛あだちクリニック	158-0093	東京都世田谷区上野毛2-7-16　玉屋ビル3F	03-6303-1114	https://www.adachi-clinic.or.jp/	○	○	内科、外科<br>乳腺外科<br>肛門外科	足立　幸博	東京医療センター
1002	ふくろうクリニック自由が丘	158-0083	東京都世田谷区奥沢6-20-23<br>フォーラム自由が丘1階2階	03-3705-3351	http://www.296296.jp/jiyugaoka/	○	○	脳神経内科<br>脳神経外科<br>整形外科	橋本　昌也<br>伊澤真理子	ふくろうクリニック等々力<br>（東京都世田谷区等々力3-5-2）
1003	上田クリニック	158-0083	東京都世田谷区奥沢7-19-9	03-6809-7031	http://www.aoitorikai.com/	○	○	内科皮膚科	堤　直也	
1004	ニコこどもクリニック	158-0094	東京都世田谷区玉川1-15-6-102	03-6431-0205	https://www.nicoco.jp	○	○	小児科<br>小児科	中澤　裕美子<br>内山　芽里	
1005	菅澤医院	158-0093	東京都世田谷区中町4-31-13	03-3701-1740			○	内科<br>小児科	菅澤正明	
1006	GPクリニック自由が丘	158-0083	東京都世田谷区奥沢6-21-12ベルヴェディア自由が丘201	03-6432-1223	http://gp-cl.com/	○	○	内科	・斉藤康洋<br>・安田里加子<br>・安田優<br>・亀井悠一郎	
1007	せたがや下馬クリニック	154-0002	東京都世田谷区下馬3-33-8	03-6413-8471	https://setagaya-shimouma.com/	○	○	消化器内科<br>内科	江藤　哲哉	
1008	やまおかこどもクリニック	1560044	東京都世田谷区赤堤4-20-1　古谷ビル1F	03-5355-5072	http://www.yk-clinic.jimdofree.com	○	○	小児科　内科	山岡光子	

電話や情報通信機器を用いて診療を実施する医療機関の一覧（東京都）

	基本情報					事務連絡に基づく対応について				
	施設名	郵便番号	住所（都道府県から記載）	電話番号	ウェブサイトURL	初診の電話等を用いた診療の実施の有無	再診の電話等を用いた診療の実施の有無	対応診療科	担当医師名	対面診療が必要と判断した場合に連携する医療機関名（複数ある場合は複数、住所も併せて記載）
1009	医療法人財団<br>放友クリニック	150-0042	東京都渋谷区宇田川町7-13　第2共同ビル7階	03-3464-5078			○	内科	石川　道郎<br>荒木　　洋	
1010	医療法人社団恵比寿会<br>淳クリニック	150-0021	東京都渋谷区恵比寿西1-14-2　ツムラビル2F	03-5489-0463	https://ebisukai.server-shared.com	○	○	内科<br>小児科	内藤　淳	日本赤十字社医療センター（東京都渋谷区広尾4-1-22）<br>東京都立広尾病院（東京都渋谷区恵比寿2-34-10）<br>JR東京総合病院（東京都渋谷区代々木2-1-3）<br>東京医科大学病院（東京都新宿区西新宿6-7-1）<br>厚生中央病院（東京都目黒区三田1-11-7）<br>東京共済病院（東京都目黒区中目黒2-3-8）
1011	医療法人社団エヌシー会メディカルクリニック渋谷	150-0002	東京都渋谷区渋谷3-29-17<br>JR東日本ホテルメッツ渋谷2階	03-6418-0024	http://www.nc-shibuya.com		○	内科<br>消化器科	岡野　雄介	東京都立広尾病院（東京都渋谷区恵比寿2-34-10）
1012	医療法人社団慶照会 幡ヶ谷内科クリニック	151-0072	東京都渋谷区幡ヶ谷2-16-8	03-3373-4810	https://hatagaya-hmc.com/		○	内科<br>消化器内科<br>循環器内科	蜂矢　朗彦<br>蜂矢　由美子	JR東京総合病院（東京都渋谷区代々木2-1-3）<br>東京医科大学病院（東京都新宿区西新宿6-7-1）
1013	医療法人社団玄英会<br>渋谷ウエストクリニック	150-0043	東京都渋谷区道玄坂1-5-4<br>照カビル4F	03-6416-1964	https://westclinic.tokyo/	○	○	泌尿器科、皮膚科	中村有吾	
1014	医療法人社団創友会ヒラハタクリニック	150-0002	東京都渋谷区渋谷1-24-6-9F	03-3400-3288	https://www.hirahata-clinic.or.jp/	○	○	内科	平畑光一<br>神山ほなみ<br>積山慧美里	
1015	医療法人社団　DAP<br>北青山Dクリニック	150-0001	東京都渋谷区神宮前3-7-10<br>AKERA（アケラ）ビル地下1階	050-7301-4555	https://www.dsurgery.com/	○	○	内科<br>外科<br>脳神経外科<br>循環器内科<br>呼吸器内科<br>消化器外科<br>消化器内科<br>婦人科	阿保　義久<br>辛　正廣<br>泉　雅文<br>池田　祐一<br>志賀　太郎<br>入江　秀大<br>金沢　考満<br>山下　裕玄	
1016	医療法人社団ナイズ<br>キャップスクリニック代官山T-SITE	150-0033	東京都渋谷区猿楽町17-5 代官山蔦屋書店1号館3F	03-4579-2959	https://www.caps-clinic.jp/daikanyama	○	○	小児科	野呂　惠子	
1017	医療法人社団和会<br>渋谷コアクリニック	150-0002	東京都渋谷区渋谷1-9-8<br>朝日生命宮益坂ビル3階	03-3498-2111	https://www.core-cl.com		○	内科	川井　邦彦<br>増尾　光樹	

電話や情報通信機器を用いて診療を実施する医療機関の一覧（東京都）

	基本情報					事務連絡に基づく対応について				
	施設名	郵便番号	住所（都道府県から記載）	電話番号	ウェブサイトURL	初診の電話等を用いた診療の実施の有無	再診の電話等を用いた診療の実施の有無	対応診療科	担当医師名	対面診療が必要と判断した場合に連携する医療機関名（複数ある場合は複数、住所も併せて記載）
1018	医療法人社団BODHI Dr.KAKUKOスポーツクリニック	150-0033	東京都渋谷区猿楽町9-8-111	03-5784-1101	http://www.dk-sc		○	整形外科	中村　格子<br>阿多　由梨加	
1019	医療法人社団ペルル マーガレットこどもクリニック	151-0053	東京都渋谷区代々木４-３７-１５　おやこ基地シブヤ　３F	03-6276-8657	https://margaret-kodomo.jp	○	○	小児科	田中　純子<br>吉野　ラモナ<br>大瀧　理佐子<br>曽根　尚子	
1020	医療法人道心会 恵比寿内科クリニック	150-0022	東京都渋谷区恵比寿南1-1-1 ヒューマックスビル4F	03-3719-4411	https://www.ebisu-hifuka.jp/		○	内科	菅原　道代	
1021	医療法人道心会 恵比寿皮膚科クリニック	150-0022	東京都渋谷区恵比寿南1-1-1 ヒューマックスビル4F	03-3719-2266	https://www.ebisu-hifuka.jp/medical/index5.html		○	皮膚科	丸山　和道	
1022	医療法人道心会 恵比寿形成外科・美容クリニック	150-0022	東京都渋谷区恵比寿南1-4-15 恵比寿銀座クロスビル4F	03-5725-1115	https://ebisu-keiseibiyou.jp/		○	形成外科<br>美容外科<br>美容皮膚科	西嶌　順子<br>西嶌　暁生	
1023	医療法人社団　鴻鵠会 恵比寿クリニック	150-0013	東京都渋谷区恵比寿4-1-18恵比寿ネオナート２階	03-3440-3131	http://ebisu.clinic.or.jp/	○	○	内科<br>消化器科	宗林　祐史<br>中島　清隆<br>黒堀　ゆう子	
1024	恵比寿ウィメンズクリニック	150-0022	東京都渋谷区恵比寿南1丁目4番15号恵比寿銀座クロスビル５階	03-6452-4276	https://ebisu-womens.jp/	○	○	婦人科	堤 麻衣	
1025	恵比寿門脇ブレストクリニック	150-0013	東京都渋谷区恵比寿1-9-4 ﾒﾃﾞｨｶﾙ恵比寿ﾋﾞﾙ5F	03-5424-1100	http://ebisu-breast-clinic.com/		○	乳腺外科<br>甲状腺外科	門脇　正美	
1026	えびす耳鼻咽喉科	150-0013	東京都渋谷区恵比寿1-9-4メディカル恵比寿ビル	03-3441-1187	http://ebisu-jibika.jp/s/	○	○	耳鼻咽喉科	片山　諭	

電話や情報通信機器を用いて診療を実施する医療機関の一覧（東京都）

	基本情報					事務連絡に基づく対応について				
	施設名	郵便番号	住所（都道府県から記載）	電話番号	ウェブサイトURL	初診の電話等を用いた診療の実施の有無	再診の電話等を用いた診療の実施の有無	対応診療科	担当医師名	対面診療が必要と判断した場合に連携する医療機関名（複数ある場合は複数、住所も併せて記載）
1027	恵比寿脳神経外科・内科クリニック	150-0022	東京都渋谷区恵比寿南1-4-15<br>恵比寿銀座クロスビル 3階	03-3712-1188	https://ebisu-cl.com		○	脳神経外科<br>内科	鈴木　一郎	日赤医療センター（東京都渋谷区広尾4-1-22）<br>東京都立広尾病院（東京都渋谷区恵比寿2-34-10）
1028	恵比寿みかレディースクリニック	150-0022	東京都渋谷区恵比寿南１－１４－１２　ル　ソレイユ３　３Ｆ	03-5734-1654			○	婦人科	田中　美香	
1029	大平医院	150-0031	東京都渋谷区桜丘町23-17 1F	03-3462-1575	https://ohira-clinic.jp	○	○	内科<br>小児科	大平良和<br>田邉真帆	都立広尾病院　日本赤十字医療センターなど
1030	おさめスキンクリニック	150-0021	東京都渋谷区恵比寿西2-17-8　I・T・O代官山202	03-3464-2005	http://www.osame-skinclinic.com/	○	○	皮膚科	納　さつき	同左
1031	MYメディカルクリニック	150-0042	東京都渋谷区宇田川町20-17 NMF渋谷公園通りビル5F	03-4579-9011	https://mymc.jp/	○	○	総合内科<br>消化器内科<br>糖尿病内科	笹倉　渉<br>伊藤　公博	
1032	きかわだクリニック	151-0053	東京都渋谷区代々木1-43-7　SKビル１階	03-3379-2002	https://kikawada-clinic.jp/		○	内科	黄川田　雅之	
1033	金王坂クリニック	150-0002	東京都渋谷区渋谷3-6-18 第4矢木ビル2F	03-5464-1234	https://www.konnozakaclinic.com/	○	○	内科<br>漢方内科<br>感染症科<br>精神科	西大條文一<br>木村好珠	
1034	銀座よしえクリニック表参道院	150-0001	東京都渋谷区神宮前4-9-4 BCG表参道プロパティ3F	0120-398-885	https://www.ginzabiyou.com/	○	○	皮膚科<br>美容皮膚科	担当医	
1035	クオーツメディカルクリニック	150-0002	東京都渋谷区渋谷2丁目10-10<br>徳真会QUARTZTOWER 8F	03-6362-6680	http://www.quartztower.com/medical/		○	皮膚科<br>内科	堀江　千穂	

電話や情報通信機器を用いて診療を実施する医療機関の一覧（東京都）

	基本情報					事務連絡に基づく対応について				
	施設名	郵便番号	住所（都道府県から記載）	電話番号	ウェブサイトURL	初診の電話等を用いた診療の実施の有無	再診の電話等を用いた診療の実施の有無	対応診療科	担当医師名	対面診療が必要と判断した場合に連携する医療機関名（複数ある場合は複数、住所も併せて記載）
1036	小泉クリニック	151-0072	東京都渋谷区幡ヶ谷1-31-6	03-3460-5123	http://www.koizumi.clinic		○	内科	小泉信達	
1037	サン・キタノクリニック	150-0021	東京都渋谷区恵比寿西１－２０－７	03-3461-3317		○	○	内科	北野　裕巳	
1038	参宮橋脊椎外科病院	151-0053	東京都渋谷区代々木3-57-1	03-5308-0511	https://clinics.medley.life/clinics/5c2188b324000004d69b83d2	○	○	整形外科	大堀　靖夫<br>米澤　郁穂<br>網代　泰充<br>吉田　真<br>橋本　敬史	
1039	渋谷駅前おおしま皮膚科	150-0031	東京都渋谷区桜丘町25-18NT渋谷ビル3F	03-3770-3388	https://shibuya-hifuka.jp/	○	○<br>診療日の14：00～16：00	皮膚科<br>小児皮膚科<br>形成外科	大島　昇	東京大学医学部附属病院（東京都文京区本郷7-3-1）
1040	渋谷リーフクリニック	150-0043	東京都渋谷区道玄坂2-23-14　道玄坂225ビル4階	03-5457-1274	http://leaf.clinic/		○	内科<br>胃腸内科<br>泌尿器科	玉山　隆章	
1041	湘南AGAクリニック新宿本院	151-0053	東京都渋谷区代々木2-2-13　新宿TRビル4階	0120-548-911	https://www.sbc-aga.jp/clinic/branch/agashinjuku/?fd_bridge_id=YksyRnVRSHgvWGcvZEtyM1lnVUJSdz09LS13KzZMMVpvOGVaekFlbWhtTG9Xb053PTO%3D--77cf0d149b37f0c285fefb1bf16cb51557813afa		○	美容皮膚科	笠井敬一郎<br>中司 圭<br>荒川 昌彦<br>蛯沢 克己<br>塩川 一郎<br>德永 能隆<br>山本 淳子<br>谷治 春香	湘南AGAクリニック西新宿院（東京都新宿区西新宿7-21-3　西新宿大京ビル7F）<br>湘南AGAクリニック大阪院（大阪府大阪市北区曽根崎新地1-4-12　桜橋プラザビル6F）<br>湘南AGAクリニック仙台院（宮城県仙台市宮城野区榴岡2-2-12　アーバンライフ橋本2F）<br>湘南AGAクリニック福岡院（福岡県福岡市中央区天神3-3-14　ホテルアセント福岡2F）
1042	シロノクリニック恵比寿	150-0012	東京都渋谷区広尾1-1-40恵比寿プライムスクエアプラザ2F	0120-11-4422	https://www.shirono.net/about/clinic/ebisu.html	○	○	美容皮膚科	江馬　潤<br>大内　恵理<br>友利　新	
1043	神宮外苑Woman Life Clinic	150-0001	東京都渋谷区神宮前3-39-5 QizAOYAMA2F	03-6432-9385	https://www.woman-life-clinic.com/	○	○	女性内科(内科・婦人科)	伊沢 博美	日本赤十字社医療センター（東京都渋谷区広尾4-1-22 ）<br>東京都立広尾病院　（東京都渋谷区恵比寿2-34-10）<br>JR 東京総合病院(東京都渋谷区代々木2-1-3)
1044	新宿内科	151-0053	東京都渋谷区代々木2-9-5森本ビル8階	03-6276-6233	https://shinjuku-naika.jp/	○	○	内科<br>消化器科	廣瀬　徳彦<br>絹川　千尋<br>西村　亜希子	JCHO東京山手メディカルセンター<br>（東京都新宿区百人町３丁目２２－１）<br>東京医科大学病院（東京都新宿区西新宿６丁目７－１）

電話や情報通信機器を用いて診療を実施する医療機関の一覧（東京都）

	基本情報					事務連絡に基づく対応について				
	施設名	郵便番号	住所（都道府県から記載）	電話番号	ウェブサイトURL	初診の電話等を用いた診療の実施の有無	再診の電話等を用いた診療の実施の有無	対応診療科	担当医師名	対面診療が必要と判断した場合に連携する医療機関名（複数ある場合は複数、住所も併せて記載）
1045	新宿駅前うわじま皮膚科	151-0053	東京都渋谷区代々木2-6-7セイチビル4F	03-3373-4112	http://uwajima-hifuka.com	○	○	皮膚科<br>形成外科	上嶋　祐太	新宿駅前うわじま皮膚科（東京都渋谷区代々木２－６－７セイチビル４階）
1046	睡眠総合ケアクリニック代々木	151-0053	東京都渋谷区代々木5丁目10番10号SYビル	03-6300-5401	https://www.somnology.com/		○	精神科<br>耳鼻科<br>呼吸器内科<br>神経内科	井上　雄一<br>碓氷　章<br>中山　秀章<br>竹内　暢<br>松澤　重行<br>栁原　万里子<br>ほか	
1047	代官山診療所	150-0033	東京都渋谷区猿楽町24-7代官山プラザ301	03-5728-3165	http://daikanyamashinryojyo.la.coocan.jp/		○	内科<br>脳神経外科<br>外科	植木　泰行	東京都立広尾病院（東京都渋谷区恵比寿2-34-10）<br>東京共済病院（東京都目黒区中目黒2ー3ー8）
1048	代官山内科外科クリニック	150-0033	東京都渋谷区猿楽町21-3	03-6455-0460	http://d-cl.jp	○	○	内科 外科<br>整形外科 皮膚科	深瀬　達	
1049	代官山パークサイドクリニック	150-0034	東京都渋谷区代官山町16-1カスティヨ代官山2F	03-5456-6282	https://www.parksideclinic.jp/	○	○	内科	岡宮　裕	東京共済病院（東京都目黒区中目黒2ー3ー8）
1050	たからぎ医院	150-0011	東京都渋谷区東２－２４－４　荒井ビル４階	070-4293-2232	https://www.takaragi-iin.net/	○	○	小児科	宝樹　真理	
1051	千春皮フ科クリニック広尾院	150-0012	東京都渋谷区広尾5-3-13　Barbizon86 2F	03-3447-1177	https://chiharu-hifuka.com		○	皮膚科<br>形成外科<br>小児皮膚科	渡邊　千春<br>土原　佳与<br>近藤　由佳<br>朝蔭　洋子	
1052	東京都立広尾病院	150-0013	東京都渋谷区恵比寿2-34-10	03-3444-1181	https://www.byouin.metro.tokyo.lg.jp/hiroo/index.html		○	全診療科	各医師	
1053	東京原宿医院	150-0001	東京都渋谷区神宮前1-10-23	03-3402-7474			○	内科	中村　穣	

電話や情報通信機器を用いて診療を実施する医療機関の一覧（東京都）

	基本情報					事務連絡に基づく対応について				
	施設名	郵便番号	住所（都道府県から記載）	電話番号	ウェブサイトURL	初診の電話等を用いた診療の実施の有無	再診の電話等を用いた診療の実施の有無	対応診療科	担当医師名	対面診療が必要と判断した場合に連携する医療機関名（複数ある場合は複数、住所も併せて記載）
1054	道玄坂ふじたクリニック	154-0043	東京都渋谷区道玄坂1-10-19糸井ビル4F	03-5489-5100	http://www.dogenzaka/org		○	児童精神科<br>精神科<br>心療内科	藤田　基<br>藤田　観喜	
1055	内藤小児科内科医院	150-0022	東京都渋谷区恵比寿南2-5-9	03-3713-2526	http://www.naitou-cl.com/		○	内科<br>小児科	内藤章文	
1056	中島医院	150-0046	東京都渋谷区松濤1-8-16	03-346-7501	http://home.k03.itscom.net/nakajima/		○	内科<br>小児科	中島俊一	
1057	ニキ・ハートクリニック	151-0071	東京都渋谷区本町4-16-9　コヅカビル1F	03-6383-3512	https://niki-heartclinic.business.site/	○	○	内科	仁木清美	
1058	はたがや協立診療所	151-0072	東京都渋谷区幡ヶ谷3-9-11	03-5304-0621			○	内科<br>循環器科	園田　久子	
1059	浜内科クリニック	151-0066	東京都渋谷区西原３－２－４　丸幸ビル２階	03-3481-2061	https://hamaclinic.wixsite.com/mysite	○	○	内科	濱　英永	
1060	東肛門科胃腸科クリニック	150-0021	東京都渋谷区恵比寿１－９－７　ITOX3F	03-3280-0021	www.azumaclinic.com/	○	○	内科<br>胃腸科	東　光邦<br>古嶋　薫	
1061	広尾内科クリニック	150-0012	東京都渋谷区広尾5-11-12	03-6277-3916	http://www.hiroomedclinic.com/	○	○	内科	古川　孝美	都立広尾病院　（東京都渋谷区恵比寿2-34-10）<br>日本赤十字社医療センター（東京都渋谷区広尾4-1-22 ）
1062	広尾メンタルクリニック	150-0012	東京都渋谷区広尾5-14-2 広尾KKビル3階	03-6277-3133	https://hiroo-mental.jp/		○	精神科<br>心療内科	熱田　英範	

電話や情報通信機器を用いて診療を実施する医療機関の一覧（東京都）

	基本情報					事務連絡に基づく対応について				
	施設名	郵便番号	住所（都道府県から記載）	電話番号	ウェブサイトURL	初診の電話等を用いた診療の実施の有無	再診の電話等を用いた診療の実施の有無	対応診療科	担当医師名	対面診療が必要と判断した場合に連携する医療機関名（複数ある場合は複数、住所も併せて記載）
1063	フェニックスメディカルクリニック	151-0051	東京都渋谷区千駄ヶ谷３－４１－６	03-3478-3535	http://www.phoenix.gr.jp	○	○	内科、外科、婦人科、眼科	全医師対応可能	
1064	みいクリニック代々木	151-0053	東京都渋谷区代々木2－44－12	03-6276-5385	https://mih-clinic.com/	○	○	内科<br>小児科	宮田　俊男<br>三島　果子<br>三島　千明	
1065	美和メディカルクリニック	150-0001	東京都渋谷区神宮前4－2－17－4A	03-5770-3855	https://www.miwamedical.net		○	内科<br>小児科<br>皮膚科<br>アレルギー科	小林美和子	東京都立広尾病院（東京都渋谷区恵比寿2-34-10）<br>日本赤十字社医療センター（東京都渋谷区広尾4ー1ー22）
1066	Medical Switch in clinic	510-0002	東京都渋谷区渋谷1-9-5　髙橋ﾋﾞﾙ3F	03-5778-3600	http://medicalswitch.com/	○	○	心療内科<br>精神科	小林　由佳	
1067	南新宿クリニック<br>耳鼻科・小児科	151-0053	東京都渋谷区代々木2-11-12南新宿セントラルビル6F	03-3373-4109	http://minamishinjuku-clinic.jp/		○	耳鼻科<br>小児科	木村　暁弘<br>木村　絢子	JR東京総合病院（東京都渋谷区代々木2-1-3）<br>東京医科大学病院（東京都新宿区西新宿6-7-1）
1068	矢澤クリニック渋谷	151-0064	東京都渋谷区上原1-33-11　2F	03-5738-7282	https://www.yazawaclinic.jp/	○	○	内科<br>泌尿器科<br>神経内科<br>循環器内科<br>呼吸器内科<br>消化器内科<br>緩和ケア内科<br>小児科	矢澤　聰<br>田中　恒任	日本赤十字社医療センター（東京都渋谷区広尾4-1-22）<br>東京都立広尾病院（東京都渋谷区恵比寿2-34-10）<br>JR東京総合病院（東京都渋谷区代々木2-1-3）
1069	ゆみのハートクリニック渋谷	150-0031	東京都渋谷区桜ヶ丘25-18NT渋谷ビル2F	03-3461-8838	https://shibuya.yumino-clinic.com/	○	○	内科<br>循環器内科	西原崇創<br>鮫島光博<br>弓野　大	JR総合東京病院（東京都渋谷区代々木2-1-3）<br>都立広尾病院（東京都渋谷区恵比寿２丁目３４－１０）
1070	横山皮フ科クリニック	150-0012	東京都渋谷区広尾5－14－２広尾KKビル５階	03-3440-1225	https://www.yokoyama-skin-clinic.jp/index.html	○	○	皮膚科	横山美保子	
1071	代々木上原駅前内科クリニック	151-0066	東京都渋谷区西原3-7-8フィールド代々木上原2F	03-5790-9880	https://yoyogiuehara-cl.com/	○	○	内科	大田　幹	

電話や情報通信機器を用いて診療を実施する医療機関の一覧（東京都）

	基本情報					事務連絡に基づく対応について				
	施設名	郵便番号	住所（都道府県から記載）	電話番号	ウェブサイトURL	初診の電話等を用いた診療の実施の有無	再診の電話等を用いた診療の実施の有無	対応診療科	担当医師名	対面診療が必要と判断した場合に連携する医療機関名（複数ある場合は複数、住所も併せて記載）
1072	代々木上原皮膚科クリニック	151-0064	東京都渋谷区上原1-17-7 2F	03-6407-2121	http://www.uehara-hifu.jp		○	皮膚科	竹尾　千景<br>臼井　佳恵<br>保母　彩子<br>前　賢一郎	
1073	代々木の森耳鼻咽喉科	151-0063	東京都渋谷区富ヶ谷1-8-3　安達ﾋﾞｼﾞﾈｽﾊﾟｰｸﾋﾞﾙ2F	03-5453-8733	http://www.yoyoginomori-orl.com/japan/main/main.html	○	○	耳鼻咽喉科	森　幸子<br>渡辺　建介	
1074	代々木八幡クリニック	151-0063	東京都渋谷区富ヶ谷1-51-4代々木八幡猪山ビル2F	03-3465-2662	https://yyhmclinic.wixsite.com/yyhmclinic	○		内科<br>整形外科<br>心療内科<br>神経科<br>リハビリテーション科	山本　友佳子	
1075	代々木病院	151-0051	東京都渋谷区千駄ヶ谷一丁目30-7	03-3404-7661	www.tokyo-kinikai.com/yoyogi/		○	内科・外科・整形外科・麻酔科・眼科・泌尿器科・皮膚科・精神科・神経科	全ての医師が対応している	
1076	若樹内科クリニック	150-0043	東京都渋谷区道玄坂1-20-12 MT道玄坂401	03-5935-8013		○	○	内科	安田　浩子	日赤医療センター（東京都渋谷区広尾4-1-22）<br>都立広尾病院（東京都渋谷区恵比寿2-34-10）
1077	恵比寿プラスクリニック	150-0013	東京都渋谷区恵比寿1丁目7ー12　追分ビル6階	03-6277-2211	https://plus-cl.com/ebisu/	○	○	内科<br>皮膚科	松田卓也<br>吉武秀平	
1078	ウェルネストクリニック	150-0043	東京都渋谷区道玄坂2-3-2 大外ビル3階	03-6732-6001	https://wellnest-clinic.jp/	○	○	内科/循環器内科/糖尿病内科/内分泌内科/産婦人科	薬師寺忠幸	
1079	ウェルネストメンタルクリニック	150-0043	東京都渋谷区道玄坂2-3-2 大外ビル3階	03-6732-6002	https://wellnest-clinic.jp/	○	○	心療内科/精神科	井村昌義	
1080	恵比寿こどもクリニック	150-0071	東京都渋谷区恵比寿4-9-13-301	03-3442-2525	http://www.ebisukodomo.jp	○	○	小児科<br>アレルギー科	保科　しほ	日本赤十字医療センター（東京都渋谷区広尾4-1-22）

電話や情報通信機器を用いて診療を実施する医療機関の一覧（東京都）

	基本情報					事務連絡に基づく対応について				
	施設名	郵便番号	住所（都道府県から記載）	電話番号	ウェブサイトURL	初診の電話等を用いた診療の実施の有無	再診の電話等を用いた診療の実施の有無	対応診療科	担当医師名	対面診療が必要と判断した場合に連携する医療機関名（複数ある場合は複数、住所も併せて記載）
1081	アイシークリニック新宿院	151-0053	東京都渋谷区代々木2-5-3 イマス葵ビル2階	03-6276-3870	https://ic-clinic.com	○	○	形成外科	佐藤　昌樹	
1082	東京原宿クリニック	150-0001	東京都渋谷区神宮前6丁目29-3　原宿KYビル10階	03-5962-7570	https://th-clinic.com/	○	○	内科	篠原　岳	
1083	あけのほし内科クリニック	03-3386-6500	東京都中野区中野5-67-4　中野北口ビル2F	03-3386-6500	http://www.akenohoshi-clinic.jp/	○	○	内科	丸山　祥司	
1084	医療法人社団あんず会 いしい内科クリニック	165-0025	東京都中野区沼袋4-31-11 メイゾン丸山1階	03-5345-7667	http://isicl.jp/		○	内科	石井 史	
1085	医療法人社団佐介会 田中クリニック	164-0003	東京都中野区東中野3-17-18	03-5348-2707	https://tanaka-clinic.org	○	○	内科<br>呼吸器内科<br>アレルギー科	田中　佐和子	横畠病院（東京都中野区）<br>東京女子医科大学病院(東京都新宿区)
1086	医療法人社団三峰会宮下医院	164-0014	東京都中野区南台２－２４－１２	03-5385-3050		○	○	内科<br>小児科	宮下宏一	
1087	医療法人社団 浩悠会 田沼内科・小児科医院	164-0012	東京都中野区本町6-23-3	03-3380-2622	http://www.e-doctors-net.com/nakano/tanuma		○	内科<br>小児科	田沼 美昭	
1088	医療法人社団　中村診療所	164-0012	東京都中野区本町5-39-2	03-3381-3797			○	内科	中村　洋一	
1089	医療法人社団友翔会矢野クリニック	164-0001	東京都中野区中野3-28-21キャッスル中野1階	03-5341-3131	http://www.yano-c.jp		○	内科<br>小児科<br>アレルギー科	矢野　貴彦	東京警察病院　(東京都中野区中野4丁目22-1) ・河北総合病院 (東京都杉並区阿佐谷北1-7-3)

電話や情報通信機器を用いて診療を実施する医療機関の一覧（東京都）

	基本情報					事務連絡に基づく対応について				
	施設名	郵便番号	住所（都道府県から記載）	電話番号	ウェブサイトURL	初診の電話等を用いた診療の実施の有無	再診の電話等を用いた診療の実施の有無	対応診療科	担当医師名	対面診療が必要と判断した場合に連携する医療機関名（複数ある場合は複数、住所も併せて記載）
1090	太田整形外科	165-0035	東京都中野区白鷺3-15-10	03-3310-5216		○	○	整形外科	太田　信夫	河北総合病院
1091	小川クリニック	165-0034	東京都中野区大和町2-46-4	03-5327-4664	http://www.ogawaclinic.jp/	○	○	内科リウマチ科	小川純	
1092	小原病院	164-0012	東京都中野区本町3-28-16	03-3372-0311	https://www.obara.or.jp/		○	内科	服部　雅俊<br>大石　　毅	みやびハート&ケアクリニック<br>さくらクリニック
1093	かたやま医院	164-0003	東京都中野区東中野5-23-14	03-3227-0603	http://www.katayama-iin.com/	○	○	内科<br>小児科	方山　栄哲	国立国際医療研究センター病院（東京都新宿区戸山1-21-1）
1094	かみさぎキッズクリニック	165-0031	東京都中野区上鷺宮3-8-14	03-3577-8400	http://www.facebook.com/toshiohya		○	小児科	大谷俊樹	
1095	川島診療所	164-0013	東京都中野区弥生町3-27-11	03-3372-4438	http://www.kenyu-kai.or.jp/jigyousyo/kawasima.html		○	内科	伊藤　洪志	
1096	熊谷医院	165-0032	東京都中野区鷺宮3-32-5	03-3338-1515	https://medikuma.com	○	○	内科<br>放射線科	福島　祥子	
1097	クリニックヨコヤマ	165-0026	東京都中野区新井1-8-8	03-3389-2400	http://www.t3.rim.or.jp/~drtoshiy/	○	○	内科	横山　敏男<br>野上　周一	
1098	健友会　やまと診療所	165-0034	東京都中野区大和町3-3-12	03-3339-5640	http://www.kenyu-kai.or.jp/jigyousyo/yamato.html		○	内科	神谷　寿美子	

電話や情報通信機器を用いて診療を実施する医療機関の一覧（東京都）

	基本情報					事務連絡に基づく対応について				
	施設名	郵便番号	住所（都道府県から記載）	電話番号	ウェブサイトURL	初診の電話等を用いた診療の実施の有無	再診の電話等を用いた診療の実施の有無	対応診療科	担当医師名	対面診療が必要と判断した場合に連携する医療機関名（複数ある場合は複数、住所も併せて記載）
1099	小池小児科医院	165-0033	東京都中野区若宮3-2-10	03-3330-0743	https://koikech3.wixsite.com/rin-site		○	小児科	小池　林太郎<br>保崎　智子<br>吉野　浩<br>松本　居子<br>保崎　明	立正佼成会付属佼成病院（東京都杉並区和田2-25-1）
1100	さわだ医院	165-0025	東京都中野区沼袋4-24-5	03-5318-2526	http://sawada-clinic.or.jp	○	○	内科<br>小児科	佐和田哲也	
1101	耳鼻咽喉科セントラルパーク中野	164-0001	東京都中野区中野4-10-1中野セントラルパークイースト1F	03-5318-4187	https://centralparknakano-ent.com/index.html		○	耳鼻咽喉科	戸田　惠	
1102	社会医療法人社団健友会中野共立病院付属中野共立診療所	164-0001	東京都中野区中野5-45-4	03-3386-7311	http://www.kenyu-kai.or.jp/jigyousyo/nakano_sin.html	○	○	内科<br>外科<br>整形外科<br>皮膚科<br>眼科	梶尾　房枝<br>伊藤　浩一	
1103	新中野FKクリニック	164-0012	東京都中野区本町6-16-12新中野FKビル５F	03-3229-2169	https://fk-clinic.com/		○	精神科/心療内科	藤田憲一	国立研究開発法人　国立精神・神経医療研究センター病院（東京都小平市小川東町４丁目１−１）
1104	新中野内科クリニック	164-0012	東京都中野区本町4-45-9ユニバーサルスクエア新中野4F	03-6382-5270	http://www.shinnakanonaika.jp		○	内科<br>消化器内科	佐々木　克行	
1105	高田皮膚科クリニック	164-0003	東京都中野区東中野2-27-9ﾒﾃﾞｨｶﾙｺｰﾄⅠ-2F	03-3366-5620	https://www.takada-medical.com/index.html	○	○	皮膚科	髙田　和美	
1106	たかねファミリークリニック	165-0021	東京都中野区丸山2-17-13	03-5356-8120	https://www.takane-clinic.jp/	○	○	内科<br>小児科<br>皮膚科	髙根 紘希<br>髙根 歩美	
1107	高橋医院	164-0014	東京都中野区南台3-3-3	03-3381-3238	https://doctorsfile.jp/h/27495/	○	○	内科<br>小児科	高橋康男	

電話や情報通信機器を用いて診療を実施する医療機関の一覧（東京都）

	基本情報					事務連絡に基づく対応について				
	施設名	郵便番号	住所（都道府県から記載）	電話番号	ウェブサイトURL	初診の電話等を用いた診療の実施の有無	再診の電話等を用いた診療の実施の有無	対応診療科	担当医師名	対面診療が必要と判断した場合に連携する医療機関名（複数ある場合は複数、住所も併せて記載）
1108	たけうち内科	165-0027	東京都中野区野方6-8-7岸ビル1階	03-5356-5777	http://t-nogata.com	○	○	内科	鈴木良平<br>竹内友朗	
1109	多田町診療所	164-0014	東京都中野区南台3-33-5	03-3381-3191	http://www.tadamachi.or.jp	○	○	内科　小児科	右近　智雄	
1110	とどろき皮膚科クリニック	164-0001	東京都中野区中野4-2-1　中野サンキビル5F	03-3385-1112	http://www.todoroki-clinic.com	○	○	皮膚科	轟　葉子<br>岸本　恵美	
1111	新渡戸記念中野総合病院	164-8607	東京都中野区中央4-59-16	03-3382-1231	http://www.nakanosogo.or.jp		○<br>※但し処方箋の発行のみ	内科<br>脳神経内科<br>小児科<br>外科<br>整形外科<br>皮膚科<br>泌尿器科<br>婦人科	各科主治医	
1112	医療法人社団みやびみやびハート＆ケアクリニック	164-0011	東京都中野区中央3-35-4ナビウス新中野1階EAST	03-5385-3880	https://miyabi-heart.jp/	○	○	循環器内科	渡邉雅貴	
1113	武藤耳鼻いんこう科医院	164-0003	東京都中野区東中野1-35-21	03-3361-4023	https://mutojibi.jp/	○	○	耳鼻咽喉科	武藤功太郎	
1114	メディカルコート池田耳鼻咽喉科	164-0003	東京都中野区東中野2-27-7メディカルコート1 202号	03-3366-5622	http://www.e-doctors-net.com/nakano/ikeda	○	○	耳鼻咽喉科	池田美智子　窪田麻子	
1115	やじまクリニック	165-0033	東京都中野区若宮２－１－２３	03-3339-1811		○	○	内科<br>小児科	矢島ふみ子	
1116	やまさき内科クリニック	164-0011	東京都中野区中央5-39-13　シャトレソレイユ1階	03-3380-7777	https://yamasaki-nakano.jp/	○	○	内科	山崎　智久<br>山崎　循	新渡戸記念中野総合病院(東京都中野区中央4丁目59-16)<br>東京警察病院(東京都中野区中野4丁目22-1)

電話や情報通信機器を用いて診療を実施する医療機関の一覧（東京都）

	基本情報					事務連絡に基づく対応について				
	施設名	郵便番号	住所（都道府県から記載）	電話番号	ウェブサイトURL	初診の電話等を用いた診療の実施の有無	再診の電話等を用いた診療の実施の有無	対応診療科	担当医師名	対面診療が必要と判断した場合に連携する医療機関名（複数ある場合は複数、住所も併せて記載）
1117	山田医院	164-0001	東京都中野区中野２－１４－１７	03-3384-3555	http://doctorsfile.jp/h/27358/	○	○	産婦人科・小児科・内科・皮膚科	山田正興・窪田園子	
1118	吉田内科クリニック	164-0012	東京都中野区本町6-20-17	03-53028-2050	http://www.y-naika.com		○	内科	吉田慎	
1119	上高田ちば整形外科・小児科	164-0002	東京都中野区上高田1-8-13メディカルプレイス上高田101	03-3228-1555	http://chiba-cl.net/	○	○	内科<br>小児科	千葉　智子	東京警察病院(東京都中野区中野４丁目２２-１）
1120	やはたクリニック	165-0034	東京都中野区大和町１－４４－４	03-5364-5361	https://www.sennenkai.com	○	○	内科	能戸　幸司	
1121	能戸クリニック	165-0034	東京都中野区大和町1-45-12	03-3337-6700	https://www.noto-clinic.jp/	○	○	内科	能戸　保光	
1122	あきやま内科クリニック	168-0082	東京都杉並区久我山3-2-15　A&T　HOF　1F	03-5336-3301	http://www.akiyamanaika-clinic.com	○	○	内科<br>脳神経内科	秋山　尚子	
1123	あおき眼科	166-0002	東京都杉並区高円寺北3-20-18	03-6231-5148	http://aokieye@nifty.com		○	眼科	青木美奈子	河北総合病院（東京都杉並区阿佐ヶ谷北1-7-3）
1124	石井こども・内科循環器科クリニック	166-0014	東京都杉並区松ノ木1-6-21	03-3314-5677	http://www.ishiiclinic.jp/	○		内科<br>小児科	石井健輔<br>石井哲哉	
1125	石沢クリニック	166-0011	東京都杉並区梅里２丁目１－１５　3階	03-5929-1922			○	内科	石沢　和敬	河北総合病院<br>佼成病院<br>荻窪病院

電話や情報通信機器を用いて診療を実施する医療機関の一覧（東京都）

	基本情報					事務連絡に基づく対応について				
	施設名	郵便番号	住所（都道府県から記載）	電話番号	ウェブサイトURL	初診の電話等を用いた診療の実施の有無	再診の電話等を用いた診療の実施の有無	対応診療科	担当医師名	対面診療が必要と判断した場合に連携する医療機関名（複数ある場合は複数、住所も併せて記載）
1126	医療法人社団君真光寺田病院	168-0081	東京都杉並区宮前5-18-16	03-3332-1166		○	○	内科	寺田　光男	河北病院（東京都杉並区阿佐谷北1-7-3）荻窪病院（杉並区今川3-1-24）
1127	医療法人社団こぎつね会ふるはた医院	167-0022	東京都杉並区下井草4―16―2	03-5930-9119		○	○	眼科小児科	古旗茂	
1128	医療法人社団勝榮会いりたに内科クリニック	168-0063	東京都杉並区和泉4-51-6フォンティーヌ杉並1階	03-5305-5788	https://www.iritani.jp/	○	○	内科	入谷栄一<br>山本浩隆<br>大滝美浩<br>塩塚有希子	
1129	医療法人社団千紀会　吉田クリニック	167-0043	東京都杉並区上荻1-18-12　春木家ビル2階	03-5347-7300	http://www.yoshidacl.com/	○	○	内科	吉田　克彦	荻窪病院（東京都杉並区今川3-1-24）
1130	医療法人社団奏友会あんずクリニック	166-0011	東京都杉並区梅里1-7-15　3階	03-5305-3358	http://www.tubomi.co.jp/index.html	○	○	内科<br>心療内科<br>精神科	児島　直樹<br>古田　伸夫	
1131	医療法人社団ちぎら医院	167-0042	東京都杉並区西荻北3-4-4	03-3390-1238	https://www.hospita.jp/detail/145/	○	○	内科<br>小児科	千木良淳	
1132	医療法人社団はなぶさ会しんえい糖健診クリニック	166-0003	東京都杉並区高円寺南4-6-7アンフィニビル3F	03-6383-1870	https://shinei-cl.com		○	内科	永松　信哉	
1133	医療法人社団フロンティアーツ　井草内科クリニック	167-0021	東京都杉並区井草3－17－14サイレンスミヨシ101	03-5311-7077	https://igusa-naika.jp/	○	○	内科	川瀬　直登	
1134	医療法人社団碩和会下平レディスクリニック	166-0003	東京都杉並区高円寺南1－4－12	03-5377-0834	https://www.shimodaira-ladies.com/	○	○	婦人科・産婦人科	中島　由美子	東京女子医科大学病院（東京都新宿区市谷河和田町）

電話や情報通信機器を用いて診療を実施する医療機関の一覧（東京都）

	基本情報					事務連絡に基づく対応について				
	施設名	郵便番号	住所（都道府県から記載）	電話番号	ウェブサイトURL	初診の電話等を用いた診療の実施の有無	再診の電話等を用いた診療の実施の有無	対応診療科	担当医師名	対面診療が必要と判断した場合に連携する医療機関名（複数ある場合は複数、住所も併せて記載）
1135	医療法人　靖和会　林脳神経外科メディカルクリニック	166-0004	東京都杉並区阿佐谷南1-9-2　GOOD地下1階・1階	03-5305-8831	https://hayashi-noushinkei.com/	○	○	内科<br>脳神経外科	林　靖人　森原啓文　井上　剛<br>増渕　充世	河北総合病院（東京都杉並区阿佐谷北1-7-3）
1136	内山クリニック	168-0063	東京都杉並区和泉3丁目6-2 プラムビル1F	03-5355-3535	http://www.uchiyama-cli.com/	○	○	内科<br>小児科	内山　克己	
1137	永福町駅前みんなのクリニック	1680063	東京都杉並区和泉3-4-7	03-3324-1000	eifukucho.minna@gmail.com	○	○	内科　外科<br>小児科 小児外科	奥村　尚威	
1138	おおにし眼科	167-0053	東京都杉並区西荻南2-24-15藤和シティホームズ西荻窪駅前102	03-5941-2450	http://ohnishi-eye.jp		○	眼科	大西智子	荻窪病院
1139	海老沢医院	167-0021	東京都杉並区井草3-24-8	03-3390-1289			○	内科　小児科<br>皮膚科	海老澤俊浩	
1140	大宮の杜小児醫院	168-0064	東京都杉並区永福4-19-3	03-6379-5360		○	○	小児科	近田 照己	河北総合病院（東京都杉並区阿佐谷北1-7-3）
1141	荻窪かえでクリニック	167-0051	東京都杉並区荻窪5-27-5 中島第2ビル3階301号室	03-6279-9894	http://www.ogikubo-kaede-cl.com	○	○	内科・胃腸内科・外科	北山尚也	荻窪病院（東京都杉並区今川3-1-24）
1142	柿田医院	167-0022	東京都杉並区下井草2-23-5	03-3395-3602	http://www.kakita-clinic.com/	○	○	内科<br>小児科	柿田　豊	
1143	久我山レディースクリニック	168-0082	東京都杉並区久我山2-14-8	03-3331-3010	https://www.kugayama-lc.com		○	産婦人科<br>皮膚科	青木　啓光<br>青木　明恵	

電話や情報通信機器を用いて診療を実施する医療機関の一覧（東京都）

	基本情報					事務連絡に基づく対応について				
	施設名	郵便番号	住所（都道府県から記載）	電話番号	ウェブサイトURL	初診の電話等を用いた診療の実施の有無	再診の電話等を用いた診療の実施の有無	対応診療科	担当医師名	対面診療が必要と判断した場合に連携する医療機関名（複数ある場合は複数、住所も併せて記載）
1144	窪田クリニック	166-0002	東京都杉並区高円寺北３－２５－２０	03-6265-5791	http://www.sugi-kubota.com	○	○	内科・外科<br>婦人科	窪田　茂比古<br>下村　浩祐<br>下村　貴子	
1145	木暮クリニック	168-0073	東京都杉並区下高井戸１－４１－６上北沢森ビル１Ｆ	03-3329-3003	http://www.kogure-clinic.com		○	内科	木暮　大嗣	
1146	近藤医院	167-0022	東京都杉並区下井草2-36-24	03-3397-6868		○	○	内科<br>小児科	近藤慶幸　邦夫	
1147	佐々木内科・循環器内科クリニック	166-0016	東京都杉並区成田西2-11-6	03-5335-7121	http://sasaki-clinic.flips.jp		○	内科<br>循環器内科	佐々木　英樹	
1148	佐藤眼科医院	167-0051	東京都杉並区荻窪5-17-20	03-3393-1726			○	眼科	石川実佐子	
1149	耳鼻咽喉科・小児耳鼻咽喉科　宮崎クリニック	168-0063	東京都杉並区和泉３－４－７永福町駅前クリニックモール2階	03-3322-1110	https://miyazaki-orl.com	○	○	耳鼻咽喉科	宮崎日出海	
1150	松庵内科クリニック	167-0054	東京都杉並区松庵2-7-11櫻井ビル1階	03-5370-2006	http://shouannaika.jp/	○	○	内科	千葉琢哉	松庵内科クリニック（東京都杉並区松庵2-7-11櫻井ビル１階）
1151	白い鳥医院	168-0062	東京都杉並区方南２－２３－９モナークマンション方南町１F-A	03-5307-8733	https://shiroitori-iin.jp		○	耳鼻科<br>内科	堀　夏樹<br>堀みどり	立正佼成会更正病院（東京都杉並区）
1152	杉並すだクリニック	167-0021	東京都杉並区井草1-16-4	03-3395-1192	http://suginami-suda.com	○	○	内科<br>循環器内科<br>心臓血管外科<br>外科	須田　優司	荻窪病院（東京都杉並区今川3-1-24）<br>河北総合病院（東京都杉並区阿佐谷北1-7-3）<br>順天堂大学練馬病院（東京都練馬区高野台3-1-10）<br>東京医科大学病院（東京都新宿区西新宿6-7-1）

電話や情報通信機器を用いて診療を実施する医療機関の一覧（東京都）

	基本情報					事務連絡に基づく対応について				
	施設名	郵便番号	住所（都道府県から記載）	電話番号	ウェブサイトURL	初診の電話等を用いた診療の実施の有無	再診の電話等を用いた診療の実施の有無	対応診療科	担当医師名	対面診療が必要と判断した場合に連携する医療機関名（複数ある場合は複数、住所も併せて記載）
1153	杉並区夜間急病診療所	167-0051	東京都杉並区荻窪5－20－1	03-3391-1599		○	○	小児科　内科<br>外科　耳鼻科	当番医	
1154	杉並堀ノ内クリニック	166-0013	東京都杉並区堀ノ内2-29-14ライオンズマンション新高円寺1階	03-5929-1611	https://www.sugihori.com/		○	内科・小児科・皮膚科・整形外科・耳鼻科・泌尿器科		
1155	すずかわ循環器内科	168-0063	東京都杉並区和泉3-46-6	03-5300-2200	https://suzukawa-clinic.jp/	○	○	内科　循環器内科　糖尿病・脂質代謝内科	鈴川　満雄	
1156	せいきょう診療所	166-0014	東京都杉並区松ノ木3-23-8	03-3313-7365	http://www.seikyou-clinic.com	○	○	内科<br>小児科	湯浅　潤子	河北総合病院（東京都杉並区阿佐谷北1-7-3）
1157	高井戸こどもクリニック	168-0071	東京都杉並区高井戸西１－２７－２２	03-3331-6644		○	○	内科<br>小児科	柳垣　繁<br>柳垣　優江	河北総合病院（東京都杉並区阿佐谷北1-7-3）
1158	滝澤医院	168-0064	東京都杉並区永福2-12-10	03-3328-5316	eifuku-takizawa.cl@fb4.so-net.ne.jp		○	内科<br>小児科	滝澤　誠	
1159	多田小児クリニック	166-0012	東京都杉並区和田1－19－5 新村ビル１階	03-3380-0415	http://tada-kids.jp		○	小児科	多田 光	
1160	東京ビジョンアイクリニック阿佐ヶ谷	166-0004	東京都杉並区阿佐谷南3-58-1　ビーンズ阿佐ヶ谷１Ｆ	03-6383-0238	https://tokyo2020vision.com/asagaya/		○	眼科	井手武	河北総合病院<br>（東京都杉並区阿佐谷北1-7-3）
1161	なかがわ内科クリニック	167-0021	東京都杉並区井草1-2-5ワイズスクエア101	03-5311-5577	http://www/nakagawanaika.com		○	内科	中川靖章	荻窪病院（東京都杉並区今川3-1-24）<br>なかがわ内科クリニック（東京都杉並区井草1-2-5ワイズスクエア101）

電話や情報通信機器を用いて診療を実施する医療機関の一覧（東京都）

	基本情報					事務連絡に基づく対応について				
	施設名	郵便番号	住所（都道府県から記載）	電話番号	ウェブサイトURL	初診の電話等を用いた診療の実施の有無	再診の電話等を用いた診療の実施の有無	対応診療科	担当医師名	対面診療が必要と判断した場合に連携する医療機関名（複数ある場合は複数、住所も併せて記載）
1162	長沼内科	166-0015	東京都杉並区成田東3-36-8	03-3311-1803			○	内科<br>小児科	長沼裕一郎	河北総合病院(東京都杉並区阿佐谷北)
1163	西荻窪診療所	167-0053	東京都杉並区西荻南4-2-7	03-3333-5434	http://www.kenyu-kai.or.jp		○	内科	澤田　玲	
1164	ニューハート・ワタナベ国際病院	168-0065	東京都杉並区浜田山3-19-11	03-3311-1119	http://newheart.jp	○	○	心臓血管外科<br>循環器内科	渡邊　剛<br>石川　紀彦<br>その他各科医師	
1165	はっとり整形外科・リウマチ科クリニック	167-0021	東京都杉並区井草5-6-3	03-4285-9807	https://hattori-seikei.com/	○	○	整形外科<br>リウマチ科	服部宏行	
1166	浜田山病院	168-0065	東京都杉並区浜田山4-1-8	03-3311-1195	http://www.hamadayama.or.jp		○	整形外科<br>内科	小瀬　忠男<br>井上　啓造	
1167	人見クリニック	168-0072	東京都杉並区高井戸東3-16-25	03-5370-2166	https://www.hitomiclinic.com/	○	○	内科/循環器内科/小児科/脳神経外科	橋木 治<br>橋木 優哉<br>小児科担当医	
1168	渕之上クリニック	167-0033	東京都杉並区清水1ー15ー12ローカス四面道１F	03-5382-0876		○	○	内科<br>小児科	渕之上　眞澄	荻窪病院(東京都杉並区今川３丁目１-24)河北総合病院(東京都杉並区阿佐ヶ谷北1丁目7-3)東京衛生アドベンチスト病院(東京都杉並区天沼3-17-３)
1169	方南町胃腸内科・内視鏡クリニック	168-0062	東京都杉並区方南2-19-2　鈴力第三ビル３階	03-3317-0311	https://www.honancho-ge-clinic.com/	○	○	内科<br>胃腸内科	辻　昌孝	
1170	方南町さくらクリニック	168-0062	東京都杉並区方南2ー20ー5鈴力第2ビル1階	03-3315-1315	http://honancho-clinic.com/	○	○	内科	辻　綾子	

電話や情報通信機器を用いて診療を実施する医療機関の一覧（東京都）

	基本情報					事務連絡に基づく対応について				
	施設名	郵便番号	住所（都道府県から記載）	電話番号	ウェブサイトURL	初診の電話等を用いた診療の実施の有無	再診の電話等を用いた診療の実施の有無	対応診療科	担当医師名	対面診療が必要と判断した場合に連携する医療機関名（複数ある場合は複数、住所も併せて記載）
1171	マキ皮膚科クリニック	166-0015	東京都杉並区成田東1-49-7-201	03-3314-7775	http://www.maki-derma.com		○	皮膚科	川名　万季	
1172	宮下小児科医院	166-0016	東京都杉並区成田西3-20-3	03-3392-3855			○	小児科	佐々木　礼子	
1173	むらい浜田山クリニック	168-0072	東京都杉並区高井戸東４—２７－１７	03-5370-2222	https://murai.clinic/	○	○	内科	村井　謙治	
1174	山本クリニック	167-0021	東京都杉並区井草1-8-9アセッツ下井草100号室	03-3301-7856	https://www.yamamoto-cli.jp/	○	○	内科	山本クリニック	河北総合病院（東京都杉並区阿佐谷北1-7-3）<br>荻窪病院（東京都杉並区今川3-1-24）<br>東京警察病院（東京都中野区中野4-22-1）
1175	よしだ内科	166-0003	東京都杉並区高円寺南4-19-1山長ビル１F	03-3311-3293	http://yoshida-naika.net/	○	○	内科<br>小児科	芳田　工	河北総合病院
1176	和田クリニック	166-0012	東京都杉並区和田3-15-15	03-3381-5411	http://www.sgn.tokyo.med.or.jp/search/detail.php?code=8&no=277	○	○	内科<br>脳神経外科	下山一郎	佼成病院（東京都杉並区和田2-25-1）<br>東京警察病院（東京都中野区中野4-22-1）<br>河北総合病院（東京都杉並区阿佐ヶ谷1-7-3）
1177	ファインクリニック西荻南	167-0053	東京都杉並区西荻南1-1-1-101	03-5941-3916	https://fine-clinic-nishiogiminami.com	○	○	内科、呼吸器内科、循環器内科、消化器内科、泌尿器科	園田唯<br>宮部彰<br>佐藤達也<br>斎木寛	
1178	アイビー大腸肛門クリニック	170-0002	東京都豊島区巣鴨1-18-10　4F	03-5940-5811	https://ivyclinic.jp/		○	肛門科、胃腸科	山田麻子、寺田俊明　他	
1179	あずま通りクリニック	171-0022	東京都豊島区南池袋3-18-34-101	03-3982-7203		○	○	内科<br>小児科	久保	東京都立大塚病院（東京都豊島区南大塚2-8-1）

電話や情報通信機器を用いて診療を実施する医療機関の一覧（東京都）

	基本情報					事務連絡に基づく対応について				
	施設名	郵便番号	住所（都道府県から記載）	電話番号	ウェブサイトURL	初診の電話等を用いた診療の実施の有無	再診の電話等を用いた診療の実施の有無	対応診療科	担当医師名	対面診療が必要と判断した場合に連携する医療機関名（複数ある場合は複数、住所も併せて記載）
1180	吾妻医院	171-0051	東京都豊島区長崎6-2-12	03-3957-5502	http://www.azuma-clinic.jp		○	内科<br>麻酔科	遠藤　正之	
1181	荒木医院	171-0031	東京都豊島区目白３－６－４	03-3953-4006		○	○	内科	荒木　崇	
1182	アルパカ小児科耳鼻科クリニック	171-0052	東京都豊島区南長崎4ー5ー20<br>アイテラス落合南長崎3階	03－3565－6639	https://alpaca-kodomo.com/	○	○	小児科<br>耳鼻科<br>アレルギー科	西野　多聞<br>吉田　智恵<br>藤原　摩耶	
1183	あんこうメディカルクリニック	171-0021	東京都豊島区西池袋1-17-10　エキニア池袋７階	03-3971-9941	http://ankoh.jp		○	内科<br>消化器内科科	安康　晴博	
1184	池袋大谷クリニック	171-0021	東京都豊島区西池袋1-39-4	03-3986-0337	https://otani-clinic.com	○	○	呼吸器内科	大谷義夫	
1185	池袋オリーブメンタルクリニック	1700013	東京都豊島区東池袋１－４４－１５プランドール２階	03-6914-0709	https://iomc.jp/		○	精神科<br>心療内科	松島幸恵	
1186	池袋こころのクリニック	171-0022	東京都豊島区南池袋２－２７－２<br>マルグリットビル４F	03-5985-7781	http://www.ikebukuro-kokoro.com		○	精神科<br>心療内科	横山　雄二	
1187	池袋さゆり眼科	170-0013	東京都豊島区東池袋1-10-1住友池袋駅前ビル２階	03-5962-0656	https://www.sayuri-eyeclinic.com/	○	○	眼科	藤井さゆり	
1188	池袋サンシャイン通り眼科診療所	170-0013	東京都豊島区東池袋1-5-6　アイケアビル5階	03-3981-6363	http://www.shec.jp/		○	眼科	堀　好子<br>本間　慶	日本大学医学部附属板橋病院（東京都板橋区大谷口上町30-1）<br>東京都立大塚病院眼科（東京都豊島区南大塚2-8-1）

電話や情報通信機器を用いて診療を実施する医療機関の一覧（東京都）

	基本情報					事務連絡に基づく対応について				
	施設名	郵便番号	住所（都道府県から記載）	電話番号	ウェブサイトURL	初診の電話等を用いた診療の実施の有無	再診の電話等を用いた診療の実施の有無	対応診療科	担当医師名	対面診療が必要と判断した場合に連携する医療機関名（複数ある場合は複数、住所も併せて記載）
1189	池袋スリープケアクリニック	171-0022	東京都豊島区南池袋3-14-11　中町ビル1階	03-5956-3838	https://www.sleep-care.jp		○	呼吸器内科（当院CPAP処方患者の定期受診に限る）	金子　泰之	
1190	池袋東口まめクリニック	170-0013	東京都豊島区東池袋1-42-12ステーションサイドビル3階	03-6915-2472	https://mame-clinic.jp/	○	○	内科	石川　雅俊	JCHO東京新宿メディカルセンター/東京通信病院/日本大学病院/順天堂大学医学部附属順天堂医院/三井記念病院等
1191	池袋メトロポリタンクリニック	171-0021	東京都豊島区西池袋1-11-1　メトロポリタンプラザビル(ルミネ) 10F	03-5954-1266	http://www.metropolitan-clinic.com		○	内科<br>婦人科	沼田　明<br>玉崗　有告	
1192	医療法人社団櫻和会櫻和メンタルクリニック	170-0002	東京都豊島区巣鴨1-19-12八木下ビル2階・3階	03-5319-1908	http://www.ohwa-mental.jp		○	精神科・児童精神科	山野かおる　里村あゆみ　松本倫子　鈴木美央　涌水良晃　鈴木淑子	
1193	医）求林会　クリニック西川	170-0005	東京都豊島区南大塚２－４５－９<br>ヤマナカヤビル１階	03-5395-0721	https://clinic-nishikawa.com		○	精神科<br>心療内科	西川嘉伸	
1194	医療法人社団求林会<br>池袋クリスタル眼科	171-8557	東京都豊島区南大塚１－２８－２<br>池袋パルコ７階	03-3988-2388	https://www.ikebukuro-crystal-ganka.jp		○	眼科	一戸　唱<br>西川啓子	
1195	医療法人社団　明善会<br>榎本クリニック	171-0021	東京都豊島区西池袋1-2-5	03-3982-5321	http://www.enomoto-clinic.jp/		○	精神科	深間内　文彦	
1196	医療法人社団　明善会<br>新大塚榎本クリニック	170-0005	東京都豊島区南大塚3-11-9	03-6907-8061	http://wwwshinotsuka-enomoto-clinic.jp		○	精神科	松田　隆夫	
1197	医療法人社団　環桜会<br>巣鴨さくらなみき皮膚科	170-0002	東京都豊島区巣鴨2-5-12　真野ビル3F	03-3917-1241	https://sakuranamiki-hifuka.jp/		○	皮膚科<br>小児皮膚科<br>アレルギー科<br>美容皮膚科	鈴木　さやか	

電話や情報通信機器を用いて診療を実施する医療機関の一覧（東京都）

	基本情報					事務連絡に基づく対応について				
	施設名	郵便番号	住所（都道府県から記載）	電話番号	ウェブサイトURL	初診の電話等を用いた診療の実施の有無	再診の電話等を用いた診療の実施の有無	対応診療科	担当医師名	対面診療が必要と判断した場合に連携する医療機関名（複数ある場合は複数、住所も併せて記載）
1198	医療法人社団康英会池袋ユナイテッドクリニック	171-0021	東京都豊島区西池袋1-28-7ニイミビル7階	03-6907-1942	https://united-clinic.jp/		○	内科<br>自由診療<br>（ED・AGA）	細田　淳英	横浜ユナイテッドクリニック<br>221-0835神奈川県横浜市神奈川区鶴屋町2-22-4<br>第七浅川ビル4階A室<br>ユナイテッドクリニック新宿西口院<br>160-0023東京都新宿区西新宿7-1-10守矢ビル8階<br>ユナイテッドクリニック渋谷駅前院<br>150-0043東京都渋谷区道玄坂2-30-4玉久ビル4階
1199	医療法人社団さかもと会くにやクリニック	170-0005	東京都豊島区南大塚2-37-11　ハイムセクオイア101	03-5319-0873	https://kuniya.org	○	○	内科 アレルギー科	小泉久仁弥	
1200	医療法人社団朱紡会巣鴨レディースクリニック	112-0001	東京都豊島区巣鴨２－５－１２真野ビル２階	03-3916-2262	https://sugamo-lc.jp/		○	産婦人科	花田　佐知子	
1201	医療法人社団尚香会なかい耳鼻咽喉科	171-0021	東京都豊島区西池袋5ー5ー21ザ・タワーグランディア2階池袋クリニックモール	03-5960-4133	http://www.clinic-mall.jp/ikebukuro/nakai.html		○	耳鼻咽喉科	中井孝尚、中井百香	
1202	医療法人社団昭日会　後藤クリニック	170-0013	東京都豊島区東池袋2-45-4　メロス学園ビル１階	03-5928-0510	http://www.gotoh-clinic.jp/	○	○	内科<br>小児科<br>皮膚科	後藤昭子<br>後藤伊織	
1203	医療法人社団昭日会　山下医院	170-0013	東京都豊島区東池袋2-11-8　１階	03-3971-4994	http://www.yamashita-iin.or.jp/	○	○	内科<br>小児科<br>皮膚科	後藤伊織<br>後藤昭子	
1204	医療法人社団仁泉会としま昭和病院	171-0052	東京都豊島区南長崎5丁目17番9号	03-3953-5555			○	内科	大部　雅英	
1205	医療法人社団瑞幸会千川篠田整形外科	171-0043	東京都豊島区要町3-13-8	03-3959-4114			○	整形外科	篠田　瑞生	
1206	医療法人社団のぞみ会スガモ駅前整形外科	170-0002	東京都豊島区巣鴨1-14-3　藤ビル2階	03-6912-0318	https://www.sugamo-ekimae-cl.com/		○	整形外科	近見　仁	

電話や情報通信機器を用いて診療を実施する医療機関の一覧（東京都）

	基本情報					事務連絡に基づく対応について				
	施設名	郵便番号	住所（都道府県から記載）	電話番号	ウェブサイトURL	初診の電話等を用いた診療の実施の有無	再診の電話等を用いた診療の実施の有無	対応診療科	担当医師名	対面診療が必要と判断した場合に連携する医療機関名（複数ある場合は複数、住所も併せて記載）
1207	鵜木耳鼻咽喉科医院	171-0051	東京都豊島区長崎5-33-10	03-3957-4720	https://www.unoki-jibiinkouka.com	○	○	耳鼻咽喉科<br>小児耳鼻咽喉科	新井　美帆	
1208	江戸橋診療所	170-0002	東京都豊島区巣鴨４－４－８	03-3918-1768		○	○	内科<br>小児科	山崎泰行	
1209	及川醫院	170-0002	東京都豊島区巣鴨1-40-5-2階	03-3945-9270	https://www.oikawaiin.info/		○	内科<br>小児科	及川	
1210	大沢眼科	170-6007	東京都豊島区東池袋３－１－１サンシャイン60ビル7階	03-3988-3333	http://www.osawaganka.jp/	○	○	眼科	大澤　彰	大沢眼科（東京都豊島区東池袋３－１－１サンシャイン60ビル
1211	大塚・栄一クリニック	170-0005	東京都豊島区南大塚3-45-5　サンユースビル１Ｆ	03-3987-8110	http://www.otsukaeiichiclinic.jp	○	○	心療内科、内科、歯科	内田　栄一	東京都立大塚病院（東京都豊島区南大塚2-8-1）
1212	大塚北口眼科	170-0004	東京都豊島区北大塚2-6-12 KSK大塚医療ビル4F	03-3949-1195	https://www.keiseikai-group.com/ootsuka_eyeclinic/	○	○	眼科	杉本　聡子	
1213	大塚北口診療所	170-0004	東京都豊島区北大塚2-6-12 KSK大塚医療ビル1F	03-3949-1141	https://www.keiseikai-group.com/ootsuka_kitaguchi/	○	○	消化器科<br>内科	河　一京<br>小島　陽子	
1214	岡崎耳鼻咽喉科医院	171-0033	東京都豊島区高田1－40－12岡崎ビル２Ｆ	03-3981-0763		○	○	耳鼻咽喉科<br>アレルギー科	岡崎健二	
1215	北池袋こころのクリニック	170-0011	東京都豊島区池袋本町1-38-14ナゴヤビル1階	03-5924-6851	http://kitaike-cocoro.com/		○	心療内科・精神科	春日　悠貴	

電話や情報通信機器を用いて診療を実施する医療機関の一覧（東京都）

	基本情報					事務連絡に基づく対応について				
	施設名	郵便番号	住所（都道府県から記載）	電話番号	ウェブサイトURL	初診の電話等を用いた診療の実施の有無	再診の電話等を用いた診療の実施の有無	対応診療科	担当医師名	対面診療が必要と判断した場合に連携する医療機関名（複数ある場合は複数、住所も併せて記載）
1216	木村整形外科	1710022	東京都豊島区南池袋2-29-4	03-3984-3306	http://www.kimura-seikei.com		○	整形外科	木村　元	
1217	こがクリニック	170-0004	東京都豊島区北大塚１－２０－９OSKビル４０１	03-5972-1465			○	内科<br>循環器内科	古賀　規貴	
1218	新大塚こどもクリニック	170-0005	東京都豊島区南大塚2-15-7	03-3944-0808	http://eri-kids.com/	○	○	小児科	杉田　依里	東京都立大塚病院（東京都豊島区南大塚二丁目８番１号）
1219	すがも駅前耳鼻咽喉科クリニック	170-0002	東京都豊島区巣鴨1-14-5　第一松岡ビル4階	03-3947-1050	http://sugamo.sho-jin.com/	○	○	耳鼻科	高野澤　美奈子	
1220	すがも北口内科クリニック	170-0002	東京都豊島区巣鴨2-3-10<br>森川第1ビル5階	03-5980-9225	http://kaiseikai-net.or.jp/group/sugamo-naika		○	内科<br>呼吸器内科	山本　義孝	
1221	巣鴨こし石クリニック	170-0002	東京都豊島区巣鴨3-30-3	03-3918-6566	https://www.ogata-iin.com/		○	内科　小児科<br>呼吸器内科　アレルギー科	輿石　義彦	
1222	すがも小林皮フ科	170-0002	東京都豊島区巣鴨2-1-2　ブラウンタワー3階	03-5972-1825	http://www.s-Kobayashi-derma.com	○	○	皮膚科<br>美容皮膚科	小林道子	
1223	巣鴨千石皮ふ科	170-0002	東京都豊島区巣鴨1-20-10宝生第１ビル５F	03-3941-1241	https://sugamo-sengoku-hifu.jp/	○	○	皮膚科<br>アレルギー科	小西　真絢	
1224	巣鴨泌尿器科内科クリニック	170-0002	東京都豊島区巣鴨1-30-2ハイツサンアベニュー2F	03-6912-0767	http://sugamo-urology.com/	○	○	泌尿器科等	篠原充	

電話や情報通信機器を用いて診療を実施する医療機関の一覧（東京都）

	基本情報					事務連絡に基づく対応について				
	施設名	郵便番号	住所（都道府県から記載）	電話番号	ウェブサイトURL	初診の電話等を用いた診療の実施の有無	再診の電話等を用いた診療の実施の有無	対応診療科	担当医師名	対面診療が必要と判断した場合に連携する医療機関名（複数ある場合は複数、住所も併せて記載）
1225	巣鴨山口内科・糖尿病内科クリニック	170-0002	東京都豊島区巣鴨1-20-10　宝生第一ビル3階A	03-5395-5700	https://yamaguchi-dm.com/	○	○	内科<br>糖尿病内科	山口賢	
1226	セイントクリニック池袋駅前	171-0021	東京都豊島区西池袋1-16-10第2三笠ビル3階	03-6812-1902	http://www.syntcl.com/	○	○	内科<br>耳鼻咽喉科	深井　有美<br>盛田　惠	
1227	せんかわ耳鼻咽喉科	171-0043	東京都豊島区要町3-39-5	03-5926-8077	http://kjibi.net/sjibi/		○	耳鼻咽喉科	野本　剛輝	
1228	雑司が谷赤ちゃん・こどもクリニック	171-0032	東京都豊島区雑司が谷3-1-20 S.Zビル201	03-6907-0037	http://www.baby-kids-clinic.com/	○	○	小児科　内科	青柳裕之　中務秀嗣　波多野恵	都立大塚病院　東京女子医大病院　日本大学医学部付属病院　速やかに対面診療も可能です
1229	たいようクリニック池袋院	171-0014	東京都豊島区池袋2-41-2 葉山ビル5階	03-5985-4721	https://home.sun-cl.jp/	○	○	泌尿器科	赤桐　一弘	
1230	高橋クリニック	170-0003	東京都豊島区駒込2-12-11	03-5907-7311			○	内科<br>外科	高橋　望	花と森の東京病院
1231	田村医院	170-0011	東京都豊島区池袋本町1-45-16	03-3971-4922	http://www.tamura-cl.jp/		○	内科<br>小児科	田村　仁<br>田村　奈渚<br>田中　朝志	
1232	TOMクリニック池袋院	170-0013	東京都豊島区東池袋1-15-3　近代ビルNo.12　7F	03-5927-7531	https://www.tom-clinic.com/		○	自由診療<br>(AGA)	大屋久晴	
1233	東京美容医療クリニック	171-0022	東京都豊島区南池袋１－２３－１１　池袋ブロンズビル１０階	03-6709-2656	http://tokyo-biyo.com/	○	○	美容皮膚科・皮膚科	高　尚威	Wi clinic銀座院（東京都中央区銀座8－9－16長崎センタービル９階）

電話や情報通信機器を用いて診療を実施する医療機関の一覧（東京都）

	基本情報					事務連絡に基づく対応について				
	施設名	郵便番号	住所（都道府県から記載）	電話番号	ウェブサイトURL	初診の電話等を用いた診療の実施の有無	再診の電話等を用いた診療の実施の有無	対応診療科	担当医師名	対面診療が必要と判断した場合に連携する医療機関名（複数ある場合は複数、住所も併せて記載）
1234	豊島中央病院	170-0012	東京都豊島区上池袋2-42-21	03-3916-7211	https://www.toshima-chuo-hospital.jp/		○	内科	澤田　登起彦	豊島中央病院(東京都豊島区上池袋2-42-21)
1235	ノガキクリニック	171-0021	東京都豊島区西池袋3-22-13　丸栄ビル２Ｆ	03-3980-6150	http://www.nogaki-cl.com		○	泌尿器科<br>内科,外科	野垣　譲二	東京都保健医療公社豊島病院（東京都板橋区栄町）<br>日本大学医学部板橋病院（東京都板橋区大谷口）
1236	野田さくらハートクリニック	170-0003	東京都豊島区駒込4-15-19	03-3915-5500	http://noda-sakuraheart-clinic.com	○	○	内科	野田誠	
1237	林胃腸科外科クリニック	170-6007	東京都豊島区東池袋3-1-1サンシャイン60ビル7階	03-3988-5700	https://www.hayashi-clinic.org/	○	○	内科	林　竜平	都立大塚病院（豊島区南大塚2-8-1）
1238	東長崎駅前内科クリニック	171-0051	東京都豊島区長崎4-7-11　マスターズ東長崎1階	03-5926-9664	https://umeoka-cl.com/higashinagasaki/	○	○	内科消化器科	吉良文孝	
1239	ファミリークリニックしいなまち	171-0052	東京都豊島区南長崎1-25-26ファーマシティⅡ2A	03-6908-2234	http://fcls.jp/		○	内科 小児科	保坂辰樹	
1240	町のクリニック目白	171-0033	東京都豊島区高田1-19-21　1階	03-3971-5500	https://lifeworkbalance.jp/family-medicine/	○	○	内科<br>小児科<br>外科	重島祐介	
1241	南池袋パークサイドクリニック	171-0022	東京都豊島区南池袋2-27-17　いちご南池袋ビル3階	03-5957-3443	http://www.mipc.jp		○	内分泌外科<br>内分泌内科	飯原雅季<br>川真田明子<br>鈴木留美	
1242	南大塚クリニック	170-0005	東京都豊島区南大塚2－41－9　坂本ビル1階	03-3943-7277	https://www.m-otsuka-clinic.com/		○	内科、リウマチ科、アレルギー科、泌尿器科、皮膚科	岡本　完	

電話や情報通信機器を用いて診療を実施する医療機関の一覧（東京都）

	基本情報					事務連絡に基づく対応について				
	施設名	郵便番号	住所（都道府県から記載）	電話番号	ウェブサイトURL	初診の電話等を用いた診療の実施の有無	再診の電話等を用いた診療の実施の有無	対応診療科	担当医師名	対面診療が必要と判断した場合に連携する医療機関名（複数ある場合は複数、住所も併せて記載）
1243	目白駅前クリニック	171-0031	東京都豊島区目白三丁目４番１１号ヒューリック目白ビル３階	03-5906-5806	https://mejiroekimae.com/	○	○	内科<br>循環器内科	明石英之<br>岸川正大<br>青山麻織<br>渡部創<br>岡部崇志	
1244	目白すずきクリニック	171-0031	東京都豊島区目白3丁目12-11	050-3187-6569	http://mejiro3.jp/	○	○	内科	鈴木市三	豊島病院　（東京都江東区亀戸８丁目８−８）<br>日本大学医学部附属板橋病院（東京都板橋区大谷口上町３０−１）<br>東京警察病院（東京都中野区中野４丁目２２−１）<br>練馬総合病院（東京都練馬区旭丘１丁目２４−１）<br>大久保病院（ 東京都新宿区歌舞伎町２丁目４４−１）<br>聖母病院（東京都新宿区中落合２丁目５−１）<br>総合東京病院（東京都中野区江古田３丁目１５−２）
1245	目白整形外科内科	171-0031	東京都豊島区目白2-38-2	03-5960-7800	https://www.mejiro-seikei.com/	○	○	整形外科　内科	東京都新宿区	目白病院（東京都新宿区下落合3-22-23）　聖母病院（東京都新宿区中落合2-5-1）
1246	目白乳腺クリニック	171-0031	東京都豊島区目白3-4-11　ヒューリック目白3階	03-6908-1026	https://www.mejiro-breast.jp		○	乳腺外科	緒方晴樹	東京逓信病院（千代田区富士見2-4-17）
1247	目白メンタルクリニック	171-0031	東京都豊島区目白3-4-11-3階	03-5906-5863	http://www.mejiro-mental.com		○	精神科<br>心療内科	須田　賢太	
1248	めじろ安田眼科	171-0031	東京都豊島区目白3-4-11ヒューリック目白2階	03-3565-6536	https://yasuda-eye.com/		○	眼科	安田　明弘	
1249	ﾓﾓ･ﾒﾃﾞｨｶﾙ･ｸﾘﾆｯｸ	170-0005	東京都豊島区南大塚1-51-1	03-3945-3344	https://www.momo-mc.com/		○	内科	百瀬隆二	
1250	医療法人社団法山会　山下診療所大塚	170-0004	東京都豊島区北大塚2-13-1　ba07　5F	03-3910-6711	http://hozankai.com/	○	○	内科、小児科、耳鼻科、循環器内科、呼吸器内科、アレルギー科	山下　巖、成本治、稲葉　敦、竹下　久美子、野村　由香、大島　華倫、谷田けい、宮本　智史、石井　裕子、軽部　昇	
1251	ゆみのハートクリニック	171-0033	東京都豊島区高田3-14-29KDX高田馬場ビル１F	03-5956-8010	https://www.yumino-clinic.com/		○	内科<br>循環器内科<br>呼吸器内科	弓野　大<br>臼井　靖博	国立国際医療研究センター病院（東京都新宿区戸山1-21-1）

電話や情報通信機器を用いて診療を実施する医療機関の一覧（東京都）

	基本情報					事務連絡に基づく対応について				
	施設名	郵便番号	住所（都道府県から記載）	電話番号	ウェブサイトURL	初診の電話等を用いた診療の実施の有無	再診の電話等を用いた診療の実施の有無	対応診療科	担当医師名	対面診療が必要と判断した場合に連携する医療機関名（複数ある場合は複数、住所も併せて記載）
1252	吉田クリニック	170-0002	東京都豊島区巣鴨4-30-12	03-5907-3553	http://www.y-clinic2007.com	○	○	内科<br>消化器科<br>外科<br>肛門科<br>リハ科	吉田竜介	＊当院に来院するか、不可能であれば、一心病院（豊島区北大塚1-18-7）
1253	立教通り皮フ科形成外科	171-0021	東京都豊島区西池袋3-29-9	03-5954-3051	https://rikkyo-dps.com	○	○	皮膚科、アレルギー科	須澤　由希子	
1254	和こころクリニック	171-0014	東京都豊島区池袋2-48-9 恩京ｱﾑｰﾙﾋﾞﾙ2階	03-6907-2520	https://wacocoro-clinic.jp		○	精神科<br>心療内科	中山　寿一	
1255	池袋心療内科メディカルオーククリニック	171-0021	東京都豊島区西池袋1-39-4第一大谷ビル5階	03-5391-9602	http://medical-oak.com		○	心療内科<br>精神科	小川原　純子<br>山岡　昌之	成増厚生病院(東京都板橋区三園)
1256	北池袋よしだ内科クリニック	170-0012	東京都豊島区上池袋４－２９－９北池テラス4階	03-3916-8010	https://kitaike-clinic.com		○	内科<br>循環器内科<br>消化器内科	吉田　裕志<br>吉田　裕志<br>吉田　見紀	
1257	青木内科クリニック	114-0024	東京都北区西ヶ原1-46-17	03-5961-1855	http://www.aoki-naika-clinic.com/	○	○	内科<br>小児科	青木　薫	都立大塚病院（東京都豊島区南大塚2-8-1）
1258	赤羽胃腸肛門クリニック	115-0055	東京都北区赤羽西1-40-5　3F	03-5924-1500	https://akabaneichoukoumon.com/		○	大腸・肛門外科、胃腸内科、内科	山村芳弘、葛岡健太郎　他	
1259	赤羽駅前女性クリニック	115-0045	東京都北区赤羽1-13-1ミドリヤビル４階	03-6903-9090	https://akabane-ekimaecl.com	○	○	産婦人科	深沢　瞳子	
1260	赤羽すずらん眼科	115-0045	東京都北区赤羽2-9-10　グリーンハイム赤羽101	03-3903-6336	http://suzuraneye.byoinnavi.jp/pc/		○	眼科	大須賀方子	

電話や情報通信機器を用いて診療を実施する医療機関の一覧（東京都）

	基本情報					事務連絡に基づく対応について				
	施設名	郵便番号	住所（都道府県から記載）	電話番号	ウェブサイトURL	初診の電話等を用いた診療の実施の有無	再診の電話等を用いた診療の実施の有無	対応診療科	担当医師名	対面診療が必要と判断した場合に連携する医療機関名（複数ある場合は複数、住所も併せて記載）
1261	赤羽中央総合病院	115-0044	東京都北区赤羽南2-5-12	03-3902-0348	http://www.hakueikai.or.jp		○	内科	外来担当医	
1262	赤羽病院	115-0045	東京都北区赤羽2-2-1	03-3902-3261	https://www.akabane-hp.com		○	内科<br>整形外科<br>外科	古川　清憲他	
1263	いさ内科クリニック	114-0002	東京都北区王子1-25-10アルカディア津多屋１F	03-3927-1105		○	○	内科<br>小児科<br>神経内科	伊佐　文子	
1264	板橋眼科山田医院	114-0023	東京都北区滝野川7-11-10	03-3916-5885			○	眼科	山田　眞	
1265	いずみ整形外科クリニック	115-0055	東京都北区赤羽西1-4-15　グランデール赤羽	03-5948-2911	https://www.izumi-seikei.com/		○	整形外科	泉　正隆	
1266	いとう王子神谷内科外科クリニック	114-0002	東京都北区王子5-5-3 ｼｰﾒｿﾞﾝ王子神谷3階	03-5959-7705	https://itokc.jp/	○	○	内科<br>小児科<br>外科<br>乳腺外科<br>呼吸器内科<br>消化器内科<br>内視鏡内科<br>皮膚科	伊藤　博道<br>伊藤　美帆	基本的に当院で対応する。重症例・治療抵抗例・数的に困難な場合は、東京北医療センター・明理会中央総合病院・帝京大学病院へ紹介しております。
1267	医療法人社団　五十嵐医院	115-0045	東京都北区赤羽1-49-9	03-3901-3131	http://www.igarashi-iin.com	○	○	皮膚科	五十嵐　勝	
1268	医療法人社団AT<br>たむら内科クリニック	115-0043	東京都北区神谷２－２５－４	03-3598-0888	https://tamura-naikaclinic.com		○	内科	田村　彰教	
1269	医療法人社団　おかだ皮フ科クリニック	115-0045	東京都北区赤羽1-2-2モン・マレー202	03-3595-1165	http://www.okada-hifuka.com		○	皮膚科　アレルギー科	岡田裕之	

電話や情報通信機器を用いて診療を実施する医療機関の一覧（東京都）

	基本情報					事務連絡に基づく対応について				
	施設名	郵便番号	住所（都道府県から記載）	電話番号	ウェブサイトURL	初診の電話等を用いた診療の実施の有無	再診の電話等を用いた診療の実施の有無	対応診療科	担当医師名	対面診療が必要と判断した場合に連携する医療機関名（複数ある場合は複数、住所も併せて記載）
1270	医療法人社団啓仁会　平川胃腸クリニック	115-0055	東京都北区赤羽西１丁目６－１ ビビオ3F	03-3900-2232	http://hirakawa-icho.med.gr.jp/	○	○	内科<br>消化器内科<br>放射線科	平川　賢	東京北医療センター（東京都北区赤羽台4-17-56）
1271	医療法人社団康優会染谷メンタルクリニック	114-0002	東京都北区王子2-12-12　松尾ビル１F	03-5959-8511	http://www.someyamental.com/	○	○	精神科<br>心療内科	染谷康宏<br>松野泰彦<br>竹内崇	東京医科歯科大学医学部附属病院（東京都文京区湯島1-5-45）
1272	医療法人社団　彩祥会　中島皮フ科	115-0045	東京都北区赤羽2－23－2　1.2階	03-3598-2823	https://nakashima-hifuka.com/	○	○	皮膚科/美容皮膚科/形成外科/アレルギー科	中島　知賀子	
1273	医療法人社団　爽健会　中田医院	114-0015	東京都北区中里２－２０－１５－２０２	03-3940-0504			○	内科	中田　一也	都立駒込病院　日本医科大学付属病院　花と森の東京病院
1274	医療法人社団慈広会　王子神谷齋藤クリニック	114-0003	東京都北区豊島8-24-4	03-3911-5433	http://www.saito-clinic.or.jp	○	○	内科 循環器科 消化器科 小児科	齋藤広重　齋藤奈々子	
1275	医療法人社団寿廣記念会岸メディカルクリニック	114-0034	東京都北区上十条2-21-1	03-3908-2103	http://www.kishi-hospital.or.jp	○	○	内科<br>整形外科	岸ひさ子・鈴木伸明・中島利菜・鈴木崇史・芳野晃	
1276	医療法人財団　富士病院	114-0024	東京都北区西ケ原３－３３－１１	03-3910-6661	http://www.fujihospital.com		○	精神科	安部 千晶	
1277	うえやま眼科	114-0002	東京都北区王子5-1-40 サミットストア王子桜田通り店2階	03-3914-3300	https://ueyama-ganka.com/		○	眼科	上山　数弘<br>上山　さやか	
1278	浮間小児科クリニック	115-0051	東京都北区浮間4－1－12	03-3558-8777	https://www.ukima-cl.com/	○	○	小児科	有坂玲子	

電話や情報通信機器を用いて診療を実施する医療機関の一覧（東京都）

	基本情報					事務連絡に基づく対応について				
	施設名	郵便番号	住所（都道府県から記載）	電話番号	ウェブサイトURL	初診の電話等を用いた診療の実施の有無	再診の電話等を用いた診療の実施の有無	対応診療科	担当医師名	対面診療が必要と判断した場合に連携する医療機関名（複数ある場合は複数、住所も併せて記載）
1279	おうじキッズクリニック	112-0002	東京都北区王子5-1-40ｻﾐｯﾄｽﾄｱ王子桜田通り店2階	03-3914-1511	http://oji-kids.com/		○	小児科	若林太一<br>米沢文恵	東京北医療センター（東京都北区赤羽台4-17-56）<br>帝京大学医学部付属病院（東京都板橋区加賀　2-11-1）
1280	王子生協病院	114-0003	東京都北区豊島3-4-15	03-3912-2201	http://oujiseikyo-hp.jp/		○	内科<br>整形<br>泌尿器	各医師	
1281	おおたけ整形外科･内科	114-0002	東京都北区王子1-8-1　王子パークビル6F	03-3914-1177	https://ojiotake-clinic.com	○	○	整形外科<br>内科	大武　修一郎<br>大武　幸子	東京北医療センター（東京都北区赤羽台3-17-56）<br>東京女子医科大学病院（東京都新宿区河田町8-1）
1282	大西医院	114-0002	東京都北区王子4-17-2	03-3911-3387		○	○	耳鼻咽喉科	長井　由紀	
1283	大橋病院	115-0054	東京都北区桐ヶ丘1-22-1	03-3907-1222	https://www.o-hp.or.jp		○	内科・神経内科<br>整形外科・外科<br>婦人科	外来担当医	
1284	おひさまクリニック	114-0034	東京都北区上十条1-19-8	03-6454-3511	https://ohisamakodomo.com/	○	○	小児科<br>耳鼻咽喉科<br>アレルギー科	金高太一<br>金高清佳	東京北医療センター（東京都北区）<br>帝京大学医学部付属病院（東京都板橋区）など
1285	おりはた乳腺胃腸パラスクリニック	115-0045	東京都北区赤羽2－13－3サトウビル2F	03-3092-3123	https://paras.jp/	○	○	乳腺外科　内科<br>消化器内科	織畑剛太郎	
1286	加治クリニック	114-0023	東京都北区滝野川7-2-7藤和板橋コープ	03-5394-1925		○	○	内科<br>小児科	加治　正弘	
1287	上中里醫院	114-0016	東京都北区上中里2-32-12	03-3911-3740	http://kaminakazato-cl.com/		○	内科	青木久幸	

電話や情報通信機器を用いて診療を実施する医療機関の一覧（東京都）

	基本情報					事務連絡に基づく対応について				
	施設名	郵便番号	住所（都道府県から記載）	電話番号	ウェブサイトURL	初診の電話等を用いた診療の実施の有無	再診の電話等を用いた診療の実施の有無	対応診療科	担当医師名	対面診療が必要と判断した場合に連携する医療機関名（複数ある場合は複数、住所も併せて記載）
1288	河村内科	115-0053	東京都北区赤羽台3丁目25番19号	03-5993-8333		○	○	内科・小児科	河村 雅明	
1289	北赤羽さきやま皮膚科	115-0052	東京都北区赤羽北2-24-24バンイードビル101	03-3905-4112	https://kitaaka-hifuka.com/		○	皮膚科	崎山真幸<br>崎山とも	
1290	桐ヶ丘団地診療所	115-0053	東京都北区赤羽台3丁目17番地	03-3907-2029	http://www.kenbun.or.jp		○	内科	高野　勝匡<br>小田　実<br>川杉　和夫<br>岩野　知世<br>岩間　昌大<br>石川　徹<br>松崎　寛崇<br>鹿戸　福子	
1291	桑畑医院	114-0003	東京都北区豊島2-6-1	03-3919-0700			○	小児科	桑畑圭子	
1292	小出医院	114-0014	東京都北区田端3-4-7	03-3821-1668		○	○	内科<br>小児科	小出研爾	都立駒込病院<br>都立大塚病院<br>日本医大
1293	公益社団法人発達協会王子クリニック	115-0044	東京都北区赤羽南2-10-20	03-3903-3311	ojiclinic/jp		○	精神科	石崎朝世<br>洲鎌倫子<br>竹内紀子<br>大谷良子<br>伊東ゆたか	
1294	河野診療所	115-0055	東京都北区赤羽西1-1-4	03-3900-4385		○	○	内科	安田寛治郎	
1295	ごとう内科	114-0002	東京都北区王子5-1-40サミットストア王子桜田通り店2階	03-3914-2510	http://gotonaika.jp/		○	内科<br>消化器内科	後藤　英晃	
1296	こまごめ内科・循環器内科<br>クリニック	114-0014	東京都北区田端4-3-2　山積ビル1F	03-5832-9600	https://komagome-naika.com/	○	○	内科<br>循環器内科	西城　由之	

電話や情報通信機器を用いて診療を実施する医療機関の一覧（東京都）

	基本情報					事務連絡に基づく対応について				
	施設名	郵便番号	住所（都道府県から記載）	電話番号	ウェブサイトURL	初診の電話等を用いた診療の実施の有無	再診の電話等を用いた診療の実施の有無	対応診療科	担当医師名	対面診療が必要と判断した場合に連携する医療機関名（複数ある場合は複数、住所も併せて記載）
1297	こやの整形外科内科	114-0002	東京都北区王子４－２２－１－１０１	03-5959-5831	http://www.koyano.or.jp/		○	整形外科<br>内科	小谷野誠司	
1298	さくらクリニック	114-0021	東京都北区岸町1-3-1 伯清王子ビル3F	03-3908-0001	https://sakura.clinic		○	泌尿器科、内科	砂倉瑞明	
1299	十条霞医院	114-0034	東京都北区上十条4-6-13	03-5948-8536	http://jujokasumiiin.jp	○	○	婦人科<br>内科	史　周霞	
1300	城北診療所	115-0052	東京都北区赤羽北二丁目19番地1号	03-3907-1523			○	内科<br>小児科<br>精神科	汐月　信仁<br>汐月　治実	板橋中央総合病院(東京都板橋区小豆沢2-12-7)<br>帝京大学医学部附属病院(東京都板橋区加賀2-11-1)
1301	須賀田医院	114-0024	東京都北区西ヶ原1-53-1エリーワールド１F	03-3918-2816			○	内科	須賀田　元彦	
1302	すこやかこどもクリニック浮間	115-0053	東京都北区浮間3-1-40	03-5918-9421	https://ukima.sukoyaka-kodomo.clinic	○	○	小児科	金井 慎一	
1303	すずきクリニック	115-0045	東京都北区赤羽2-69-4-1F	03-3598-3310	http://www.akabane-suzukiclinic.jp/	○	○	内科<br>脳神経内科	鈴木　敏文<br>鈴木　健太郎	
1304	生協浮間診療所	115-0051	東京都北区浮間3-22-1	03-3558-8361	http://www.t-hokuto.coop/clinic/ukima/		○	内科	藤沼　康樹	
1305	生協診療所	114-0001	東京都北区東十条2-8-5	03-3913-5271	http://hokuto-kita-clinic.jp/		○	内科<br>整形外科	大場　俊英<br>餌取　博<br>秋山　潤<br>渡会　公治	

電話や情報通信機器を用いて診療を実施する医療機関の一覧（東京都）

	基本情報					事務連絡に基づく対応について				
	施設名	郵便番号	住所（都道府県から記載）	電話番号	ウェブサイトURL	初診の電話等を用いた診療の実施の有無	再診の電話等を用いた診療の実施の有無	対応診療科	担当医師名	対面診療が必要と判断した場合に連携する医療機関名（複数ある場合は複数、住所も併せて記載）
1306	たかねクリニック	114-0015	東京都北区中里3-17-1	03-3949-0442		○	○	内科	高根　一郎	
1307	田中医院	114-0016	東京都北区上中里３－６－16	03-3913-6627			○	内科 小児科	田中孝幸	
1308	中島クリニック	114-0001	東京都北区東十条３－１－１４	03-6915-4700	http://www.nakajima-clinic.tokyo		○	内科	中島　剛	
1309	東十条さかい糖尿病・内科クリニック	114-0001	東京都北区東十条4丁目5-16 HJ フレール101	03-3914-2000	https://www.hjsakai-dmc.com	○	○	内科	堺　弘治	
1310	明理会中央総合病院	114-0001	東京都北区東十条3-2-11	03-5902-1199	https://www.ims.gr.jp/meirikaichuo/		○	内科<br>心臓外科<br>血管外科<br>整形外科<br>神経内科<br>眼科<br>外科<br>脳神経外科	主治医	
1311	メンタルクリニック赤とんぼ	114-0034	東京都北区上十条3-29-6 ビルヴァンテアン201	03-6454-3877	http://akatombo-mc.org/		○	心療内科	髙橋　えみ子	
1312	横山医院	114-0032	東京都北区中十条2-22-16	03-3908-3452	http://www.kenjukai.or.jp/	○	○	内科<br>小児科	横山　健一	
1313	渡辺医院	114-0024	東京都北区西ヶ原４－４８－４	03-3910-0380		○	○	内科	渡邉壽和	
1314	医療法人社団隆樹会<br>木村クリニック	115-0043	東京都北区神谷1-15-9	03-3911-1220	https://kimurahomeclinic.jp/	○	○	内科<br>小児科	木村隆雄<br>木村さら<br>宮本哲也<br>生津圭祐<br>渡辺勇<br>藤森丈広	明理会中央総合病院（東京都北区東十条3丁目2番11号）<br>東京北医療センター（東京都北区赤羽台4-17-56）<br>帝京大学医学部附属病院（東京都板橋区加賀2-11-1）<br>東京大学医学部附属病院（東京都文京区本郷7-3-1）<br>東京医科大学病院（東京都新宿区西新宿6-7-1）<br>日本医科大学附属板橋病院（東京都板橋区大谷口上町30-1）<br>東京都健康長寿医療センター（東京都板橋区栄町35番2号）

電話や情報通信機器を用いて診療を実施する医療機関の一覧（東京都）

	基本情報					事務連絡に基づく対応について				
	施設名	郵便番号	住所（都道府県から記載）	電話番号	ウェブサイトURL	初診の電話等を用いた診療の実施の有無	再診の電話等を用いた診療の実施の有無	対応診療科	担当医師名	対面診療が必要と判断した場合に連携する医療機関名（複数ある場合は複数、住所も併せて記載）
1315	たかの内科クリニック	114-0002	東京都北区王子1-10-17 ヒューリック王子ビル5F	03-3912-8544	https://oji-takano-clinic.com/	○	○	内科	野村 和至	
1316	水野医院	114-0014	東京都北区田端6-3-20 1階	03-3821-3305	https://mizuno-iin.com/		○	内科	水野 雅之	
1317	医療法人社団慈広会<br>西巣鴨こどもクリニック	114-0023	東京都北区滝野川6-14-9-301	03-6903-5025	http://www.nishisugamo-child.jp	○	○	小児科	大久保隆志	東京北医療センター(東京都北区赤羽台4丁目17-56)<br>東京都立大塚病院(東京都豊島区南大塚2丁目8-1)<br>東京都保健医療公社 豊島病院(東京都板橋区栄町33-1)
1318	あべクリニック	116-0014	東京都荒川区東日暮里6-60-10 日暮里駅前中央ビル5F	03-5810-7808	https://abeclinic.com	○	○	精神科	阿部哲夫	
1319	荒川生協診療所	116-0002	東京都荒川区荒川4-54-5	03-3802-2601	http://arakawaseikyo.com/		○	内科	田邉 康一<br>伊沢 庸之	
1320	医療法人社団いなばキッズクリニック	116-0003	東京都荒川区南千住４－７－１ BiVi南千住3階	03-5604-1710	http://www.inabakids.com	○	○	小児科	稲葉八興 稲葉利佳子	
1321	医療法人社団しんけん会<br>東尾久耳鼻咽喉科・アレルギー科	116-0012	東京都荒川区東尾久5-23-5インペリアルトパーズ1F	03-6240-8741	https://higashiogu-ent.com	○	○	耳鼻咽喉科	小山 京子	
1322	うえだ眼科クリニック	116-0011	東京都荒川区西尾久1-12-5 ロジュマン西尾久101	03-5692-3100		○	○	眼科	上田 裕子	
1323	尾久駅前内科クリニック	116-0011	東京都荒川区西尾久７－２５－３	03-5855-0979	www.okumed.com/	○	○	内科<br>循環器科<br>神経内科	冨永 誠一<br>大久保 誠二	

電話や情報通信機器を用いて診療を実施する医療機関の一覧（東京都）

	基本情報					事務連絡に基づく対応について				
	施設名	郵便番号	住所（都道府県から記載）	電話番号	ウェブサイトURL	初診の電話等を用いた診療の実施の有無	再診の電話等を用いた診療の実施の有無	対応診療科	担当医師名	対面診療が必要と判断した場合に連携する医療機関名（複数ある場合は複数、住所も併せて記載）
1324	古賀整形外科	116-0012	東京都荒川区東尾久3-30-4	03-3892-8814	http://kogaseikei.jp/index.html		○	整形外科	古賀三郎、渋谷一行、眞々田一浩	都立駒込病院、三井記念病院、東京逓信病院等
1325	社会医療法人社団一成会<br>木村病院	116-0001	東京都荒川区町屋2-3-7	03-3892-3161	http://www.kimura-hp.or.jp	○<br>症状によってお受けできない場合もあります	○<br>症状によってお受けできない場合もあります	内科<br>外科	各医師	
1326	上智クリニック	116-0001	東京都荒川区町屋4-9-10	03-3892-4514			○	内科	銭谷　平<br>田尾　まゆ子	
1327	鈴木こどもクリニック	116-0011	東京都荒川区西尾久3丁目21-5　AYビル1F	03-5855-3030		○	○	小児科	北爪　勉	
1328	東京女子医科大学東医療センター	116-8567	東京都荒川区西尾久2-1-10	03-3810-1111	https://twmu-mce.jp/index.html		○	全診療科	全医師	
1329	はたの耳鼻咽喉科	116-0003	東京都荒川区南千住7-1-1アクレスティ南千住304	03-6806-8733	https://hatano-jibika.jp/	○	○	耳鼻咽喉科	波多野　吟哉	
1330	まる福ホームクリニック	116-0011	東京都荒川区西尾久４－２７－３	03-6807-9810	https://www.marufuku-hc.com	○	○	内科<br>小児科	菅野　哲也	東京女子医科大学東医療センター（東京都荒川区）
1331	宮の前診療所	116-0011	東京都荒川区西尾久2-3-2	03-3800-7111		○		内科	山本　藍	
1332	上野小児科医院	116-0003	東京都荒川区南千住1-13-22	03-3806-4970	http://uenokids.byoinnavi.jp/pc/index.html		○	小児科	上野　正浩	東京女子医大東医療センター<br>（東京都荒川区西尾久２丁目１－１０）

電話や情報通信機器を用いて診療を実施する医療機関の一覧（東京都）

	基本情報					事務連絡に基づく対応について				
	施設名	郵便番号	住所（都道府県から記載）	電話番号	ウェブサイトURL	初診の電話等を用いた診療の実施の有無	再診の電話等を用いた診療の実施の有無	対応診療科	担当医師名	対面診療が必要と判断した場合に連携する医療機関名（複数ある場合は複数、住所も併せて記載）
1333	青山クリニック	174-0064	東京都板橋区中台3ー27 サンシティA101・103	03-3931-8888	https://www.aocli.jp/		○	内科<br>小児科	青山 友則	板橋中央総合病院<br>板橋区医師会病院
1334	赤塚眼科はやし医院	175-0093	東京都板橋区赤塚新町1-24-5	03-3938-8900	http://www.akatsuka-eye-clinic.com/		○	眼科	林　殿宣	
1335	赤塚新町クリニック	175-0093	東京都板橋区赤塚新町2-5-16ヴィルヌーブ赤塚1F	03-6915-6617	https://www.a-h-clinic.com/	○	○	内科	塩原康正	板橋区医師会病院<br>（東京都板橋区高島平3-12-6）
1336	小豆沢病院付属高島平診療所	175-0082	東京都板橋区高島平8-1-1	03-3932-3394			○<br>定期受診患者の定期処方のみ医師との電話再診可能	内科	佐藤　達郎、桑名　慶和その他各曜日の担当医師	
1337	天木診療所	174-0053	東京都板橋区清水町47-7	03-3961-3913			○	内科<br>小児科	天木聡	
1338	飯沼病院	174-8680	東京都板橋区常盤台2-33-15	03-3960-0091	http://www.iinuma-hp.com		○	内科<br>心療内科<br>精神科	山路朋久<br>大野裕治<br>飯沼久美子<br>齋藤令子<br>湊口龍<br>阿部察貴男<br>小林勉<br>五十嵐紀之	
1339	石澤内科クリニック	174-0056	東京都板橋区志村2-10-9	03-3966-1438	http://ishizawa-clinic.net/		○	内科	石澤　将	板橋区医師会病院（東京都板橋区高島平3丁目12-6）<br>板橋中央総合病院（東京都板橋区小豆沢2-12-7）
1340	いたばし・ハートクリニック	173-0004	東京都板橋区板橋1-21-7　聖楽ビル1階	03-5248-0880	http://www.itabashi-heart.com		○	内科<br>循環器内科	原田　忠宜	
1341	板橋区役所前診療所	173-0013	東京都板橋区氷川町1-12　コスモディエース３階	03-5375-9031	http://ita-shinryojo.jp/		○	内科	島田　潔<br>鈴木陽一<br>浅海　直<br>安井宏仁<br>三寺隆之<br>御子柴路朗<br>糸山　智	平成ホームクリニック（東京都板橋区向原三丁目7-7-2階）<br>平成ゆうわクリニック（埼玉県戸田市新曽南4-2-35-1階）

電話や情報通信機器を用いて診療を実施する医療機関の一覧（東京都）

	基本情報					事務連絡に基づく対応について				
	施設名	郵便番号	住所（都道府県から記載）	電話番号	ウェブサイトURL	初診の電話等を用いた診療の実施の有無	再診の電話等を用いた診療の実施の有無	対応診療科	担当医師名	対面診療が必要と判断した場合に連携する医療機関名（複数ある場合は複数、住所も併せて記載）
1342	板橋すばる眼科	173-0016	東京都板橋区中板橋20-5-302	03-6905-3511	http://www.subaru-eye.com	○	○	眼科	菅波　絵理	板橋区医師会病院（東京都板橋区高島平3丁目12-6）
1343	板橋セントラルクリニック	174-0051	東京都板橋区小豆沢2-16-15	03-3967-1268	http://www.ims.gr.jp/itabashi.hp/		○	皮膚科<br>眼科<br>小児科	問わず	
1344	板橋中央総合病院	174-0051	東京都板橋区小豆沢1-12-7	03-3967-1181	http://www.ims.gr.jp/itabashi.hp/		○	内科<br>腎臓内科<br>血液内科<br>リウマチ科<br>糖尿病内科<br>脳神経内科<br>呼吸器内科<br>循環器内科	問わず	
1345	イムス板橋健診クリニック	174-0051	東京都板橋区小豆沢2-23-15	03-3967-1515	https://www.ims.gr.jp/kenshin/	○	○	内科・循環器内科	平　久美子	
1346	イムス記念病院	174-0071	東京都板橋区常盤台4-25-5	03-3932-9181	http://www.ims.gr.jp/ims-kinen/	○	○	全診療科	主治医	
1347	医療法人社団かのう会かのう耳鼻咽喉科クリニック	173-0004	東京都板橋区板橋1-53-10-2F2B	03-6905-3387	www.kano-jibi.jp		○	耳鼻科<br>小児耳鼻科	加納　章子<br>梓澤　陽子<br>蕨　希欧伊<br>北見　エミ	
1348	医療法人社団　ごんだクリニック	175-0082	東京都板橋区高島平2-33-1 ピーコックストア高島平店2階	03-5922-5377	https://gonda-cl.com		○	内科<br>消化器外科<br>肛門外科	権田　剛	
1349	医療法人社団慈誠会東武練馬中央病院	175-0083	東京都板橋区徳丸3-19-1	03-3934-1611	http://www.tobunerima-hospital.jp		○	内科	森<br>小澤<br>小池<br>吉居	
1350	医療法人社団仁恕会メンタルクリニックいたばし	173-0004	東京都板橋区板橋1−21−5　1,2F	03-3961-9603	http://www.mental-clinic.net/	○	○	精神科	高橋　彰久<br>関口　緑	

電話や情報通信機器を用いて診療を実施する医療機関の一覧（東京都）

	基本情報					事務連絡に基づく対応について				
	施設名	郵便番号	住所（都道府県から記載）	電話番号	ウェブサイトURL	初診の電話等を用いた診療の実施の有無	再診の電話等を用いた診療の実施の有無	対応診療科	担当医師名	対面診療が必要と判断した場合に連携する医療機関名（複数ある場合は複数、住所も併せて記載）
1351	医療法人社団多比良医院	175-0094	東京都板橋区成増4-13-2-1階	03-3975-8139	https://tairaiin.jp		○	内科<br>小児科	多比良 清	板橋区医師会病院（東京都板橋区高島平3-12-6）
1352	医療法人財団　同潤会<br>富士見病院	173-0012	東京都板橋区大和町１４番１６号	03-3962-2431	https://www.fujimi-hp.or.jp/gairai.html		○	内科<br>外科胃腸科<br>泌尿器科	各外来担当医	
1353	医療法人社団　ふるた小児クリニック	175-0083	東京都板橋区徳丸２−２１−８	03-3932-7800			○	小児科	古田俊哉	
1354	医療法人社団翠会<br>みどりの杜クリニック	175-0094	東京都板橋区成増5-6-3	03-5967-2770	http://www.mhcg.or.jp/midorinomori/	○	○	精神科	森川すいめい<br>北　麻希子	
1355	おおの内科クリニック	173-0004	東京都板橋区板橋1-36-1　TYビル3F	03-3963-5521	http://www.ohnomc.tokyo.jp/	○	○	内科	大野　安実<br>大野　美穂<br>佐伯　仁	
1356	大森メディカルクリニック	174-0065	東京都板橋区若木1-17-17	03-5921-0755			○	内科	大森　千春	板橋区医師会病院（東京都板橋区高島平3丁目12-6 ）
1357	大山東方クリニック	173-0014	東京都板橋区大山東町60-9	03-3964-1830		○	○	内科<br>外科	小出　雅彦	東京都長寿医療センター
1358	金谷整形外科<br>せぼね・骨粗しょう症クリニック	174-0056	東京都板橋区志村二丁目10番1号<br>PLAZA　NIKI　1階	03-3965-5252	https://kanaya-seikei.com		○	整形外科	金谷　幸一	東京都健康長寿医療センター<br>住所；東京都板橋区栄町35-2
1359	亀井消化器内科クリニック	175-0094	東京都板橋区成増1-28-15林屋ビル2F	03-3976-4180			○	内科<br>消化器内科	亀井　明	東京都健康長寿医療センター<br>（東京都板橋区栄町３５−２）<br>板橋区医師会病院<br>（東京都板橋区高島平−１２−１６）

電話や情報通信機器を用いて診療を実施する医療機関の一覧（東京都）

	基本情報					事務連絡に基づく対応について				
	施設名	郵便番号	住所（都道府県から記載）	電話番号	ウェブサイトURL	初診の電話等を用いた診療の実施の有無	再診の電話等を用いた診療の実施の有無	対応診療科	担当医師名	対面診療が必要と判断した場合に連携する医療機関名（複数ある場合は複数、住所も併せて記載）
1360	亀田整形外科	175-0083	東京都板橋区徳丸2-3-10久美ビル１F	03-3933-9988	https://www.kameda-seikei.com/	○	○	整形外科	吉田　弘範	
1361	共助会医院	174-0051	東京都板橋区小豆沢2-26-8	03-5924-0266	http://kyoujyokai.com/	○	○	内科<br>小児科	堀内	豊島病院<br>日大病院<br>健康長寿医療センター<br>帝京病院
1362	楠医院	175-0092	東京都板橋区赤塚6-23-14	03-3939-0096	https://www.kusunoki-clinic.jp/		○	内科<br>産婦人科<br>泌尿器科	板倉宏尚<br>板倉伊吹	
1363	公益財団法人　愛世会<br>愛誠病院	173-8588	東京都板橋区加賀1-3-1	03-3961-5351	http://www.aisei-byouin.or.jp/		○	内科<br>外科<br>整形外科<br>循環器内科<br>糖尿病内科<br>神経内科<br>漢方内科<br>皮膚科	松原Ｄｒ<br>川上Ｄｒ<br>玉山Ｄｒ<br>島田Ｄｒ<br>その他担当Ｄｒ	
1364	公益財団法人東京都保健医療公社　豊島病院	173-0015	東京都板橋区栄町33-1	03-5375-1234	http://www.toshima-hp.jp		○<br>当院通院中の患者で定期的受診であって、医師が電話診療可能と判断した患者さんのみを対象	全科	外来担当医師	
1365	幸南クリニック	173-0034	東京都板橋区幸町23-7コモディイイダ幸町店別館１F	03-5917-3900	http://konanc.jp/	○<br>初診は受診歴のある方のみ	○	内科	加藤富嗣<br>伊藤嘉恭<br>植田文博	
1366	こうのファミリーケア・クリニック	174-0056	東京都板橋区志村3-20-26ー1階	03-3965-1649	http://www013.upp.so-net.ne.jp/Kouno-clinic/	○	○	内科<br>小児科	河野　嘉英	豊島病院（東京都板橋区栄町３３－１）
1367	さいしょ小児科	174-0074	東京都板橋区東新町２－５６－９SOB春原ビル１０１	03-5986-1271	http://www5e.biglobe.ne.jp/~saisho/	○	○	小児科<br>内科	税所純敬	
1368	サンシティ耳鼻咽喉科	174-0064	東京都板橋区中台3-27-7-2F	03-5922-4187	http://suncity-ent.com	○	○	耳鼻咽喉科	鈴川　佳吾	

電話や情報通信機器を用いて診療を実施する医療機関の一覧（東京都）

	基本情報					事務連絡に基づく対応について				
	施設名	郵便番号	住所（都道府県から記載）	電話番号	ウェブサイトURL	初診の電話等を用いた診療の実施の有無	再診の電話等を用いた診療の実施の有無	対応診療科	担当医師名	対面診療が必要と判断した場合に連携する医療機関名（複数ある場合は複数、住所も併せて記載）
1369	慈誠会前野病院	174-0063	東京都板橋区前野町6-38-3	03-3969-1511	http://maeno-hospital.jp/		○	内科	内科すべての医師	
1370	志村坂上あおき眼科	174-0051	東京都板橋区小豆沢2-15-11	03-3967-1146	https://aoki-eyeclinic.com/		○	眼科	青木　彩	
1371	下赤塚診療所	175-0092	東京都板橋区赤塚2-9-4	03-3979-6361	http://www.kenbun.or.jp/		○	内科	鹿戸　福子	東京都健康長寿医療センター（板橋区栄町36-2）<br>日本大学医学部附属板橋病院（板橋区大谷口上町30-1）<br>帝京大学付属病院（板橋区加賀2-11-1）
1372	荘病院	173-0004	東京都板橋区板橋1-41-14	03-3963-0551	http://www.sho-hospital.jp/usr/pc/index.php		○	婦人科	荘　隆一郎	
1373	心身障害児総合医療療育センター	173-0037	東京都板橋区小茂根1-1-10	03-3974-2146	https://www.ryouiku-net.com/		○	整形外科<br>小児科<br>精神科	伊藤　順一<br>中谷　勝利<br>米山　明	
1374	すがやこどもクリニック	174-0051	東京都板橋区小豆沢2ー36ー13マツエクリニックビル3階	03-5914-3777	http://www.sugaya-clinic.com/	○	○	小児科	菅谷明則	
1375	杉内医院	175-0094	東京都板橋区成増3-17-16	03-3930-2331		○	○	小児科<br>内科	杉内　孝謙	板橋区医師会病院（東京都板橋区高島平3-12-6）
1376	誠志会病院	174-0043	東京都板橋区坂下1-40-2	03-3968-2621	http://www.seisikai.or.jp	○	○	内科	内野啓子<br>福井容子	
1377	高島平中央総合病院	175-0082	東京都板橋区高島平1-73-1	03-3936-7451	https://takasimadaira-hospital.jp		○	内科、消化器科、整形外科、脳神経外科、乳腺外科、形成外科、小児科、耳鼻咽喉科、皮膚科、眼科	各担当医	

電話や情報通信機器を用いて診療を実施する医療機関の一覧（東京都）

	基本情報					事務連絡に基づく対応について				
	施設名	郵便番号	住所（都道府県から記載）	電話番号	ウェブサイトURL	初診の電話等を用いた診療の実施の有無	再診の電話等を用いた診療の実施の有無	対応診療科	担当医師名	対面診療が必要と判断した場合に連携する医療機関名（複数ある場合は複数、住所も併せて記載）
1378	高島平2丁目整形外科	175-0082	東京都板橋区高島平2-22-2	03-5399-1231			○	整形外科	鈴木英弘<br>長田知也<br>川上順子	
1379	田島クリニック	175-0094	東京都板橋区成増5-18-4	03-5383-6622		○	○	内科	林　潤洙	
1380	田幡医院	175-0083	東京都板橋区徳丸3-13-9	03-3933-1627	http://www.tabataiin.com…	○	○	内科	田幡　雅彦	板橋区医師会病院（東京都板橋区高島平３－１２－６）
1381	帝京大学医学部附属病院	173-8606	東京都板橋区加賀2-11-1	03-3964-1211	http://www.teikyo-hospital.jp/		○	全診療科	全担当医	
1382	東京シティクリニック大山	173-0023	東京都板橋区大山町9-6　大山EMPビル2F	03-3955-2200	https://tokyocityclinic-ooyama.com/	○	○	内科<br>小児科	菱木　三佳乃	
1383	東京腎泌尿器センター大和病院	173-0001	東京都板橋区本町36-3	03-5943-2411	https://www.ims.gr.jp/yamato/		○	全診療科	担当医	
1384	東京都健康長寿医療センター	173-0015	東京都板橋区栄町３５番２号	03-3964-1141	https://www.tmghig.jp/hospital/		○	全科	各診療科担当医	
1385	常盤台外科病院	174-0071	東京都板橋区常盤台2-25-20	03-3960-7211	http://tokiwadai-geka.jp/		○	整形外科<br>脳神経外科<br>形成外科<br>外科<br>内科<br>皮膚科<br>泌尿器科	[illegible]<br>大澤 俊雄<br>村上 宏史<br>洪 理江<br>瀬下 崇<br>望月 龍二<br>沼本ロバート知彦<br>[illegible]	
1386	ときわ台村中眼科	174-0071	東京都板橋区常盤台1-2-3-　新光常盤台ビル4階	03-5915-2003	https://muranaka-ganka.com/		○	眼科	村中　公正	徳丸村中眼科

電話や情報通信機器を用いて診療を実施する医療機関の一覧（東京都）

	基本情報					事務連絡に基づく対応について				
	施設名	郵便番号	住所（都道府県から記載）	電話番号	ウェブサイトURL	初診の電話等を用いた診療の実施の有無	再診の電話等を用いた診療の実施の有無	対応診療科	担当医師名	対面診療が必要と判断した場合に連携する医療機関名（複数ある場合は複数、住所も併せて記載）
1387	徳丸村中眼科	175-0083	東京都板橋区徳丸5-3-4	03-5920-2003	https://muranaka-ganka.com/		○	眼科	久保　玲子	ときわ台村中眼科
1388	徳山内科外科	107-0043	東京都板橋区坂下3-12-10	03-3966-8737			○	内科・神経内科<br>外科・整形外科	徳山　仲根<br>徳山　承明	
1389	中小路整形リハビリクリニック	174-0043	東京都板橋区坂下2-15-5	03-5393-2477	http://www.nakakohjircl.com		○	整形外科	中小路拓	板橋区医師会病院
1390	なかむらクリニック	174-0056	東京都板橋区志村1-12-6	03-5948-7378			○	内科	中村　直也	
1391	成増眼科医院	175-0094	東京都板橋区成増2-17-30	03-5997-9421			○	眼科	清水　敬子	板橋区医師会病院（東京都板橋区高島平3-12-6）
1392	成増高橋眼科	175-0094	東京都板橋区成増1-13-6　田中ビル2F	03-39979-6726			○	眼科	高橋賢一郎<br>高橋恵実	
1393	日本大学医学部附属板橋病院	173-8610	東京都板橋区大谷口上町30番１号	03-3972-8111	http://www.med.nihon-u.ac.jp/hospital/itabashi/		○<br>対面診療が必要となる場合あり	総合科、血液・膠原病内科、腎臓・高血圧・内分泌内科、糖尿病・代謝内科、循環器内科、呼吸器内科、消化器・肝臓内科、心療内科、脳神経内科、小児科・新生児科、[illegible]	各担当医	
1394	ねぎし整形外科クリニック	175-0082	東京都板橋区高島平2-33-1　ピーコックストア２Ｆ	03-6906-6226			○	整形外科<br>リウマチ科<br>ペインクリニック外科	根岸　慎一	
1395	ねや内科クリニック	175-0083	東京都板橋区徳丸5-3-22	03-5922-5100	http://www.neya-naika.jp		○	内科<br>循環器内科	禰屋和雄	板橋区医師会病院<br>（東京都板橋区高島平３－１２－６）

電話や情報通信機器を用いて診療を実施する医療機関の一覧（東京都）

	基本情報					事務連絡に基づく対応について				
	施設名	郵便番号	住所（都道府県から記載）	電話番号	ウェブサイトURL	初診の電話等を用いた診療の実施の有無	再診の電話等を用いた診療の実施の有無	対応診療科	担当医師名	対面診療が必要と判断した場合に連携する医療機関名（複数ある場合は複数、住所も併せて記載）
1396	野村医院	173-0004	東京都板橋区板橋2-65-10-2F	03-3964-8544	http://nomura-clinic.com/	○	○	内科	野村　和至	
1397	野村循環器科内科クリニック	173-0014	東京都板橋区大山東町18-4　北岡ビル2階	03-5375-3711	http://www.nomuraclinic.com		○	循環器内科<br>内科	野村　周三	
1398	はせがわ眼科	175-0094	東京都板橋区成増2-14-5　EH第二ビル2階A室	03-5998-1222	https://www.narimasuekimae-hasegawa.com		○	眼科	長谷川　裕基	
1399	早川医院	173-0026	東京都板橋区中丸町9-1	03-3972-6162		○	○	内科<br>小児科	早川　洋	日本大学医学部附属板橋病院<br>帝京大学医学部附属病院<br>健康長寿医療センター<br>東京都保健医療公社　豊島病院
1400	林クリニック	173-0037	東京都板橋区小茂根4-28-14	03-3956-2090	hayashi-medical.com	○	○	内科<br>小児科	林滋<br>林毅	板橋区医師会病院（東京都高島平3-12-6）
1401	光が丘クリニック	175-0093	東京都板橋区赤塚新町3-32-12-204	03-5968-3030	https://hikarigaokaclinic.com/	○	○	内科	徳安　良紀	
1402	平沼クリニック	173-0004	東京都板橋区板橋2-16-6	03-6784-1190	https://www.hiranuma-clinic.net/		○	内科	平沼　孝之	
1403	平山医院	174-0064	東京都板橋区中台1-46-3	03-3932-3598		○	○	小児科<br>内科	平山貴度	板橋区医師会病院
1404	藤田医院	174-0055	東京都板橋区泉町23-8-1Ｆ	03-3960-2822	http://www.fujitaiin.org/		○	内科<br>小児科	藤田雅巳	日本大学医学部附属病院（東京都板橋区大谷口上町30-1）<br>東京都保健医療公社　豊島病院（東京都板橋区栄町33-1）<br>東京都健康長寿医療センター（東京都板橋区栄町35-2）

電話や情報通信機器を用いて診療を実施する医療機関の一覧（東京都）

	基本情報					事務連絡に基づく対応について				
	施設名	郵便番号	住所（都道府県から記載）	電話番号	ウェブサイトURL	初診の電話等を用いた診療の実施の有無	再診の電話等を用いた診療の実施の有無	対応診療科	担当医師名	対面診療が必要と判断した場合に連携する医療機関名（複数ある場合は複数、住所も併せて記載）
1405	双葉町クリニック	173-0011	東京都板橋区双葉町12-17	03-3961-8060	http://www.futabacho-cl.com/		○	内科<br>アレルギー科小児科<br>皮膚科	清水園子	板橋区医師会病院（東京都板橋区高島平３丁目１２－６）
1406	平成ホームクリニック	173-0036	東京都板橋区向原三丁目7-7　ｺｰｼｬﾊｲﾑ向原7号棟2階	03-5926-4491			○	内科	藤井秀樹<br>伊藤　直<br>長谷川大輔	平成ゆうわクリニック（埼玉県戸田市新曽南4-2-35-1階）<br>板橋区役所前診療所（東京都板橋区氷川町1-12-3階）
1407	まえだファミリークリニック	175-0092	東京都板橋区赤塚3-9-1リバティカイト1F	03-3979-0901	https://www.maeda-family.jp/	○	○	内科<br>小児科<br>心療内科<br>精神科	前田　修司	
1408	まつもとクリニック	174-0056	東京都板橋区志村2-10-1-1F	03-3558-7117	http://matsu-cl.jp		○	内科	松本浩次	板橋中央総合病院
1409	水野医院	175-0093	東京都板橋区赤塚新町一丁目17-1	03-3559-2111			○	内科<br>脳神経外科	水野　重樹	板橋区医師会病院<br>（東京都板橋区高島平３－１２－６）
1410	みなくちクリニック	175-0083	東京都板橋区徳丸2-14-8	03-6785-3791			○	内科<br>外科	水口博之	板橋区医師会病院
1411	宮田医院	175-0082	東京都板橋区高島平8-12-9	03-3559-8480	https://www.itb.tokyo.med.or.jp/kikan/byoin/kobetsu_base.html?id=270		○	内科<br>小児科	宮田隆<br>宮田久裕	板橋区医師会病院（東京都板橋区高島平3-2-6）
1412	やごうクリニック	174-0071	東京都板橋区常盤台3-24-13 ホワイトクロス5 1階	03-5915-5500	https://www.itb.tokyo.med.or.jp/kikan/byoin/kobetsu_base.html?id=169		○	内科<br>消化器内科	矢郷　祐三	
1413	山倉医院	173-0031	東京都板橋区大谷口北町51-2	03-3956-2626			○	内科小児科	徳永誠／由子	日大板橋病院　東京都健康長寿医療センター

電話や情報通信機器を用いて診療を実施する医療機関の一覧（東京都）

	基本情報					事務連絡に基づく対応について				
	施設名	郵便番号	住所（都道府県から記載）	電話番号	ウェブサイトURL	初診の電話等を用いた診療の実施の有無	再診の電話等を用いた診療の実施の有無	対応診療科	担当医師名	対面診療が必要と判断した場合に連携する医療機関名（複数ある場合は複数、住所も併せて記載）
1414	山櫻診療所	174-0076	東京都板橋区上板橋3-15-5	03-3933-8739			○	内科小児科	山田由紀	
1415	よつくら医院	174-0063	東京都板橋区前野町2-3-17	03-3969-0250			○	泌尿器科	四倉正己	板橋区医師会病院
1416	渡辺医院	173-0016	東京都板橋区中板橋26-6	03-3964-0831	https://www.wwatanabe-iin.com/		○	整形外科<br>内科<br>皮膚科	渡辺　仁	板橋区医師会病院（東京都板橋区高島平3-12-6）
1417	渡辺産婦人科医院	175-0082	東京都板橋区高島平2-3-14	03-5399-3008	http://www.watanabe-sanfu-clinic.com/	○	○	産婦人科<br>小児科	渡邉　哲也	板橋区医師会病院<br>（東京都板橋区高島平３－１２－６）
1418	板橋幸町クリニック	173-0034	東京都板橋区幸町21-13プラザさいわい1Ｆ	03-5966-5801		○	○	内科	横山卓司	日本大学医学部付属板橋病院（板橋区大谷口上町30-1）<br>豊島病院（板橋区栄町33-1）<br>健康長寿医療センター（板橋区栄町35-2）
1419	中川医院	174-0071	東京都板橋区常盤台１－１２－１	03-3969-9421		○	○	眼科	中川　哲郎	
1420	医療法人社団ナイズ キャップスクリニック板橋	175-0083	東京都板橋区徳丸二丁目6番1号イオン板橋ショッピングセンター4階	03-6906-6397	https://caps-clinic.jp/itabashi/	○	○	小児科	白岡亮平	練馬光が丘病院(東京都練馬区光が丘2-11-1)<br>帝京大学医学部附属(東京都板橋区加賀２丁目11-1)<br>河北総合病院(東京都杉並区阿谷北 1-7-3)
1421	おおやま耳鼻咽喉科・アレルギー科	173-0023	東京都板橋区大山町9-6　大山EMPビル3F	03-3972-3387	https://ooyama-ent.com/	○	○	耳鼻咽喉科<br>アレルギー科	菊地　仁	
1422	いくこ皮フ科クリニック	177-0044	東京都練馬区上石神井1-14-4エソール2F-B	03-5927-5200	https://ikuko-hifuka.com/	○	○	皮膚科	末原郁子	

電話や情報通信機器を用いて診療を実施する医療機関の一覧（東京都）

	基本情報					事務連絡に基づく対応について				
	施設名	郵便番号	住所（都道府県から記載）	電話番号	ウェブサイトURL	初診の電話等を用いた診療の実施の有無	再診の電話等を用いた診療の実施の有無	対応診療科	担当医師名	対面診療が必要と判断した場合に連携する医療機関名（複数ある場合は複数、住所も併せて記載）
1423	石黒内科クリニック	176-0004	東京都練馬区小竹町２－８１－８	03-5917-6100	http://www.ishiguro-clinic.com/	○	○	内科	石黒久貴	
1424	医療法人財団厚生協会<br>大泉病院	178-0061	東京都練馬区大泉学園町6-9-1	03-3924-2111	https://www.oizumibyoin.jp/		○	精神科	主治医	
1425	医療法人財団　秀行会<br>阿部クリニック	176-0002	東京都練馬区桜台二丁目１番７号	03-3992-1103	http://www.syukoukai.or.jp/group/abe_top.html		○	内科<br>小児科	中村　哲郎	
1426	医療法人社団アップル会<br>藤澤皮膚科	178-0063	東京都練馬区東大泉1-37-14-2階	03-3925-8947	http://fujisawahifuka.com/	○	○	皮膚科	藤澤重樹<br>藤澤大輔	日大板橋病院（東京都板橋区大谷口上町30-1）<br>順天堂練馬病院（東京都練馬区高野台3-1-10）
1427	医療法人社団上石神井サン・クリニック	177-0044	東京都練馬区上石神井3-6-34	03-5910-3888	https://www.kamishakujii-sun-clinic.com/		○	内科	小西　正樹	
1428	医療法人社団弘順会大井手クリニック	178-0063	東京都練馬区東大泉5-34-10	03-5905-2625	http://www.oide-clinic.com/		○	内科<br>小児科	大井手弘純	順天堂練馬病院（東京都練馬区高野台3-1-10）
1429	医療法人社団晃仁会内田内科胃腸クリニック	176-0012	東京都練馬区豊玉北5-7-15	03-5912-1241	https://uchida-cl.jp/		○	内科	内田康仁	
1430	医療法人社団光葉会　佐川クリニック	179-0083	東京都練馬区平和台2-11-14	03-6915-7233	https://www.sagawaclinic.com/houmon	○	○	内科	佐川　直彦<br>佐川　絵理	
1431	医療法人社団浩陽会 はやみ医院	178-0061	東京都練馬区大泉学園町5-7-21	03-3922-3956		○	○	内科<br>小児科	早水　順子	

電話や情報通信機器を用いて診療を実施する医療機関の一覧（東京都）

	基本情報					事務連絡に基づく対応について				
	施設名	郵便番号	住所（都道府県から記載）	電話番号	ウェブサイトURL	初診の電話等を用いた診療の実施の有無	再診の電話等を用いた診療の実施の有無	対応診療科	担当医師名	対面診療が必要と判断した場合に連携する医療機関名（複数ある場合は複数、住所も併せて記載）
1432	医療法人社団弘和会歌橋医院	176-0021	東京都練馬区貫井4－25－31	03-3999-6767	http://www.utahashi-clinic.com/	○	○	内科<br>小児科	歌橋和哉	順天堂練馬病院（東京都練馬区高野台3-1-10）
1433	医療法人社団康和会<br>田中医院	177-0032	東京都練馬区谷原６－９－８	03-3996-5700	http://www.tanakaiin.net	○	○	内科<br>小児科	田中　康之	
1434	医療法人社団咲良会さくまクリニック	179-0072	東京都練馬区光が丘3-9-2 IMA南館2F	03-6904-2656	https://sakuma-cl.jp/		○	泌尿器科<br>皮膚科	咲間　隆裕	
1435	医療法人社団三世会 ねぎしクリニック	176-0012	東京都練馬区豊玉北4-19-6	03-3991-0328	http://negishiclinic.com		○	内科<br>小児科	佐藤 秀一	
1436	医療法人三芽会原田クリニック	177-0041	東京都練馬区石神井町4-1-14繁田ビル2階東号	03-6766-3022	http://www.haradaclinic.com	○	○	内科消化器内科	原田　昇	
1437	医療法人社団順洋会<br>武蔵野総合クリニック練馬	176-0001	東京都練馬区練馬1-26-1	03-3993-7015	http://www.6340-group.jp/clinic-n/	○	○	総合診療科	小林医師	
1438	医療法人社団祥和会<br>中村内科クリニック	179-0071	東京都練馬区旭町2-9-13	03-3977-3673	https://www.nakamura-naikaclinic.jp/	○	○	内科	出口　祥子	
1439	医療法人社団清栄会加藤医院	179-0076	東京都練馬区土支田1-6-6	03-3922-5861	http://www.katou-iin.jp	○	○	内科<br>小児科<br>呼吸器科	加藤　博一	
1440	医療法人社団世仁会すずき耳鼻咽喉科	176-0021	東京都練馬区貫井３－８－４	03-3577-9560	http://suzuki-ent.jp	○	○	耳鼻科	鈴木伸弘	

電話や情報通信機器を用いて診療を実施する医療機関の一覧（東京都）

	基本情報					事務連絡に基づく対応について				
	施設名	郵便番号	住所（都道府県から記載）	電話番号	ウェブサイトURL	初診の電話等を用いた診療の実施の有無	再診の電話等を用いた診療の実施の有無	対応診療科	担当医師名	対面診療が必要と判断した場合に連携する医療機関名（複数ある場合は複数、住所も併せて記載）
1441	医療法人社団ヅーフハルマ中村医院	177-0032	東京都練馬区谷原2-11-5	03-3997-0250		○	○	内科	中村　光彦	坪田和光病院（埼玉県和光市白子2-12-15）
1442	医療法人社団　徳枝会佐藤小児科皮膚科クリニック	177-0051	東京都練馬区関町北1－22－10－201	03-3928-2767	http://www.hifu-codomo.com		○	小児科<br>皮膚科	佐藤　徳枝<br>海老原亜貴子<br>倉繁公美子	順天堂練馬病院等
1443	医療法人社団中山クリニック	179-0073	東京都練馬区田柄2-45-8	03-3939-0361	https://nakayama-nerima.clinic/	○	○	内科・消化器内科・外科	中山　昇	
1444	医療法人社団柔和会藤澤こどもクリニック	176-0012	東京都練馬区豊玉北6－6－6	03-3557-7950	http://fujisawa-kodomo.com	○	○	小児科　内科<br>アレルギー科<br>皮膚科	藤澤　孝人<br>浦邉　智美	
1445	医療法人社団のと小児科クリニック	179-0083	東京都練馬区平和台4-12-6	03-5945-9855	https://www.noto-clinic.com/heiwadai/	○	○	内科、小児科	能登　信孝<br>藤田　ひろ子	日大板橋病院（東京都板橋区大谷口上町30-1）
1446	医療法人社団平真会　薬師堂診療所	177-0035	東京都練馬区南田中3-26-3	03-3997-2657	http://www.yakushido.jp/	○	○	内科	平良　眞一郎	
1447	医療法人社団平成会たいら整形外科クリニック	179-0076	東京都練馬区土支田2-29-19	03-5947-3699	https://taira-seikeigeka.jp/	○	○	整形外科	平良　勝成	
1448	医療法人社団倫佑会富士見台眼科	176-0021	東京都練馬区貫井3-11-14　1F	03-5987-5311			○	眼科	浅野　由香	
1449	医療法人社団和泰会　平和台クリニック	179-0083	東京都練馬区平和台4-26-8ザ・グランディール平和台Ⅰ	03-5922-1241	http://www.d-disk.net/heiwadai/	○	○	内科	千葉井基泰	安田病院（東京都板橋区成増1-13-9）

電話や情報通信機器を用いて診療を実施する医療機関の一覧（東京都）

	基本情報					事務連絡に基づく対応について				
	施設名	郵便番号	住所（都道府県から記載）	電話番号	ウェブサイトURL	初診の電話等を用いた診療の実施の有無	再診の電話等を用いた診療の実施の有無	対応診療科	担当医師名	対面診療が必要と判断した場合に連携する医療機関名（複数ある場合は複数、住所も併せて記載）
1450	上原医院	179-0081	東京都練馬区北町８丁目１２−１０	03-3935-2226		○	○	内科、小児科	上原　毅	
1451	江古田みずのクリニック	176-0006	東京都練馬区栄町３２－１３スワースモアＮ１Ｆ	03-6914-6475	https://www.mizuno-cl.net		○	精神科、心療内科	水野智之	
1452	大泉アカデミアクリニック	178-0063	東京都練馬区東大泉1-30-4第7三幸ﾋﾞﾙ3F	03-5933-2522	www.med-academia.com		○	内科　外科	脇田　進一	
1453	大泉学園こども・思春期クリニック	178-0063	東京都練馬区東大泉6-47-18ドクターズポート大泉学園	03-5935-7861	oizumi-kodomo.com/		○	小児科	齋藤　正博	
1454	大泉学園桜クリニック	178-0061	東京都練馬区大泉学園町7 - 24 - 15	03-5933-1075		○	○	内科<br>外科	北島　圭浩	
1455	大泉生協病院	178-0063	東京都練馬区東大泉6-3-3	03-5387-3111	http://www.ooizumi.net		○	内科	各科かかりつけ医	
1456	大泉中央クリニック	178-0061	東京都練馬区大泉学園町６−２７−２	03-3923-5678	https://www.oizumi-clinic.com	○	○	内科<br>外科	砂村　眞琴	国立埼玉病院（埼玉県和光市諏訪2-1）<br>順天堂大学練馬病院（東京都練馬区高野台3-1-10）
1457	おおぐちこどもクリニック	1790074	東京都練馬区春日町5-33-30-1F	359719009	https://www.kodomoclinic.info	○	○	小児科	大口展生	自院で可能ですが、入院が必要そうなら順天堂練馬病院に紹介します。
1458	大角医院	177-0044	東京都練馬区上石神井4-3-23	03-3920-0966	https://oosumi-cl.net/	○	○	内科・外科・小児科・泌尿器科・呼吸器科	中島・伊藤・毛利・紀伊	

電話や情報通信機器を用いて診療を実施する医療機関の一覧（東京都）

	基本情報					事務連絡に基づく対応について				
	施設名	郵便番号	住所（都道府県から記載）	電話番号	ウェブサイトURL	初診の電話等を用いた診療の実施の有無	再診の電話等を用いた診療の実施の有無	対応診療科	担当医師名	対面診療が必要と判断した場合に連携する医療機関名（複数ある場合は複数、住所も併せて記載）
1459	大野医院	176-0005	東京都練馬区旭丘1-55-5	03-3951-8182	http://ohno-cli.com/	○	○	内科<br>小児科	大野大二	
1460	おなかの富士見台クリニック	176-0021	東京都練馬区貫井3-3-10富士屋ビル3階	03-5971-2421	http://www.onaka-fujimidai.com/		○	内科<br>肛門科	服部　正一	河北総合病院（東京都杉並区阿佐谷北1-7-3）
1461	かくたに内視鏡消化器内科クリニック	176-0041	東京都練馬区石神井町3-21-9第三島光ビル３階	03-3997-7149	https://www.nerima-naisikyo.com/	○	○	内視鏡内科<br>消化器内科<br>内科	角谷　宏	東京慈恵会医科大学（東京都港区新橋3-19-18）<br>練馬光が丘病院（東京都練馬区光が丘2-11-1）
1462	笠井内科循環器クリニック	176-0021	東京都練馬区貫井3-11-12ジョイントヒルズ３F	03-3577-8071	http://www.kasai-naika.com	○	○	内科<br>循環器内科	笠井　建彰	
1463	加藤クリニック	177-0051	東京都練馬区関町北２－２７－１１－５F	03-3920-3920	www2.gol.com/users/katoc/		○	内科	加藤久良	東京医科歯科大学医学部付属病院（東京都文京区湯島１－５－４５）
1464	金田医院	177-0045	東京都練馬区石神井台７－２－１０	03-3920-1065	http://kanedaiinn.com		○	内科消化器内科	金田伸章	
1465	かわはらメンタルクリニック	179-0075	東京都練馬区高松5-18-4　ｻﾝﾌﾗﾜｰ大門光が丘1F	03-6913-1523	https://kawahara-cl.byoinnavi.jp/		○	精神科<br>心療内科	河原　聖之	当院（病状に応じ、対面診察・来院を依頼することあり）
1466	くどうクローバークリニック	177-0051	東京都練馬区関町北２丁目２６－１７	03-5903-5070	https://www.kudocloverclinic.jp/	○	○	内科	工藤學	順天堂練馬病院（東京都練馬区高野台３－１－１０）
1467	こくぶん医院	179-0081	東京都練馬区北町１－３６－１８	03-3933-5550			○	内科小児科	國分裕司<br>國分百合子	

電話や情報通信機器を用いて診療を実施する医療機関の一覧（東京都）

	基本情報					事務連絡に基づく対応について				
	施設名	郵便番号	住所（都道府県から記載）	電話番号	ウェブサイトURL	初診の電話等を用いた診療の実施の有無	再診の電話等を用いた診療の実施の有無	対応診療科	担当医師名	対面診療が必要と判断した場合に連携する医療機関名（複数ある場合は複数、住所も併せて記載）
1468	権藤醫院	178-0063	東京都練馬区東大泉１－２７－２５	03-3923-5515		○	○	内科	権藤守男	国立東京病院(東京都清瀬市竹丘３－１－１)<br>国立埼玉病院(埼玉県和光市諏訪２－１)
1469	桜台診療所	1760002	東京都練馬区桜台1-7-5	03-3993-0305	http://www.myclinic.ne.jp		○	精神科<br>心療内科	辰野剛	
1470	さとう内科医院	177-0045	東京都練馬区石神井台4-7-3石神井台クリニックモール1階	03-5903-9613	http://satoh-naikaiin.jp/	○	○	内科・循環器内科	佐藤　元	順天堂練馬病院（東京都練馬区高野台3-1-10）荻窪病院（東京都杉並区今川3-1-24）
1471	慈雲堂病院	177-0053	東京都練馬区関町南四丁目１４番５号	03-3928-6511	http://www.jiundo.or.jp		○	内科<br>精神科	有賀正恵<br>田邉英一ほか多数	
1472	しまむらファミリークリニック	179-0074	東京都練馬区春日町3-29-8 2Ｆ	03-5848-6477	https://shimamura-fc.com/		○	内科	島村　元章	
1473	下島メディカルクリニック	170-0081	東京都練馬区北町2-36-6ソレム守屋１Ｆ	03-5945-3266	http://www.shimojima-clinic.com	○	○	内科　小児科	下島ひろみ	
1474	順天堂大学医学部附属練馬病院	177-8521	東京都練馬区高野台3-1-10	03-5923-3111	https://www.juntendo.ac.jp/hospital_nerima/		○	総合診療・性差科<br>（外来）<br>総合診療・性差科<br>循環器内科<br>消化器内科<br>呼吸器内科<br>腎・高血圧内科<br>膠原病・リウマチ内科<br>[illegible]	各医師	
1475	新桜台 内科・外科クリニック	176-0002	東京都練馬区桜台1ー28ー8　シースリーモール新桜台2階	03-3993-9090	https://sakuradai-clinic.jp/		○	内科<br>外科	橋本　大樹	練馬総合病院（東京都練馬区旭丘1-24-1）<br>順天堂大学練馬病院（東京都練馬区高野台3-1-10）<br>日大板橋病院（東京都板橋区大谷口上町30番1号）
1476	新桜台中村ファミリークリニック	176-0003	東京都練馬区羽沢2-2-14	03-3991-7131	http://www.s-nfc.com		○	内科<br>小児科<br>皮膚科	中村　聡美	

電話や情報通信機器を用いて診療を実施する医療機関の一覧（東京都）

	基本情報					事務連絡に基づく対応について				
	施設名	郵便番号	住所（都道府県から記載）	電話番号	ウェブサイトURL	初診の電話等を用いた診療の実施の有無	再診の電話等を用いた診療の実施の有無	対応診療科	担当医師名	対面診療が必要と判断した場合に連携する医療機関名（複数ある場合は複数、住所も併せて記載）
1477	すみ医院	176-0021	東京都練馬区貫井1-5-9	03-3999-7321			○	精神科	鷲見　すみ江	
1478	すみれホームケアクリニック	177-0031	東京都練馬区三原台3-26-14 ポレール石神井403	03-6904-5828	https://sumire-clinic.jp	○	○	内科	市場　保	大泉生協病院（東京都練馬区東大泉6-3-3）
1479	関町こどもクリニック	168-0072	東京都練馬区関町北５－１５－２６　２F	03-5991-0888	http://sekimachi-kodomo.com/	○	○	小児科<br>アレルギー科	伊藤　真樹	
1480	関町内科クリニック	177-0051	東京都練馬区関町北5-6-1	03-5903-3881		○	○	内科<br>小児科	申　偉秀	国立国際医療研究センター<br>豊島病院
1481	妹尾眼科医院	178-0063	東京都練馬区東大泉５－３６－６	03-3922-5341		○	○	眼科	妹尾一恵	
1482	竹下医院	178-0064	東京都練馬区南大泉5-36-9	03-3922-3341	https://www.takeshita-clinic.jp/		○	内科・外科	安藤　由美子	
1483	築根クリニック	177-0052	東京都練馬区関町東1-22-11	03-3928-7868			○	内科	築根　吉彦<br>石橋みゆき	
1484	つかさクリニック	178-0063	東京都練馬区東大泉１－２８－６モンセーヌ大泉3階	03-5947-5800	http://www.tsukasa-c.com	○	○	内科<br>小児科	下田　司	
1485	つちやハートクリニック	176-0024	東京都練馬区中村3-17-6内田ビル１F	03-5933-9914	http://t-heartclinic.com		○	内科<br>循環器内科	土屋　洋人	順天堂大学医学部附属練馬病院（〒177-8521 東京都練馬区高野台3-1-10）

電話や情報通信機器を用いて診療を実施する医療機関の一覧（東京都）

	基本情報					事務連絡に基づく対応について				
	施設名	郵便番号	住所（都道府県から記載）	電話番号	ウェブサイトURL	初診の電話等を用いた診療の実施の有無	再診の電話等を用いた診療の実施の有無	対応診療科	担当医師名	対面診療が必要と判断した場合に連携する医療機関名（複数ある場合は複数、住所も併せて記載）
1486	とうみょう内科クリニック	177-0035	東京都練馬区南田中1-13-13プレミールコート1F	03-5923-6262	http://www.tomyo-clinic.jp	○	○	内科<br>リウマチ科	東名　正幸	
1487	富田いきいきクリニック	177-0051	東京都練馬区関町北4-4-17	03-3928-2781	http://www.tomida-ikiiki.com	○	○	内科、外科、脳神経外科	富田　修一先生	
1488	内科 吉沢医院	179-0071	東京都練馬区旭町3-25-21-1F	03-6423-0707	http://naika-yoshizawaiin.jp/	○	○	内科	吉澤亜人	
1489	中村内科クリニック	178-0061	東京都練馬区大泉学園町4-22-13	03-3867-1711			○	内科　小児科<br>循環器科	中村　尚己	
1490	中村橋駅前内科クリニック	176-0023	東京都練馬区中村北3-23-5<br>シュウカワグチビル1階	03-5848-7830	https://nakamurabashi-naika.com	○	○	内科	篠原　明成	
1491	沼口整形外科・小児科	179-0072	東京都練馬区光が丘5-2-5-102	03-3976-0131	http://numaguchi.clinic/	○	○	小児科	沼口俊介<br>沼口俊平	
1492	ねりま駅前キッズクリニック	176-0001	東京都練馬区練馬1-17-1　４F	03-6914-8821	https://nerima-kids.com/	○	○	小児科	成高中之<br>徳竹忠臣	順天堂大学附属練馬病院（練馬区高野台3-1-10）<br>練馬光が丘病院（練馬区光が丘2-11-1）
1493	練馬駅前 内視鏡・乳腺クリニック	176-0001	東京都練馬区練馬1-4-1　ユニティフォーラム９階	03-5912-1230	http://www.nbec.jp		○	乳腺外科　内科	佐貫潤一　佐貫千秋	
1494	練馬総合病院	176-8530	東京都練馬区旭丘１－２４－１	03-5988-2200	https://nerima-hosp.or.jp/		○	全科	各医師	

電話や情報通信機器を用いて診療を実施する医療機関の一覧（東京都）

	基本情報					事務連絡に基づく対応について				
	施設名	郵便番号	住所（都道府県から記載）	電話番号	ウェブサイトURL	初診の電話等を用いた診療の実施の有無	再診の電話等を用いた診療の実施の有無	対応診療科	担当医師名	対面診療が必要と判断した場合に連携する医療機関名（複数ある場合は複数、住所も併せて記載）
1495	練馬第二診療所	176-0001	東京都練馬区練馬1-6-16	03-3991-4670	http://www.kenbun.or.jp/		○	内科	奈倉　志のぶ	
1496	野口医院	176-0001	東京都練馬区豊玉上１－１３－６	03-3994-5018	http://www.shinseikai-cc.jp	○	○	内科	野口　眞利	
1497	野崎クリニック	177-0054	東京都練馬区立野町14-15	03-3920-4066		○	○	内科	野崎　基	
1498	氷川台のと小児科クリニック	179-0084	東京都練馬区氷川台3-40-6　1階3階	03-5946-9950	https://www.noto-clinic.com/hikawadai/		○	小児科	能登　孝昇	
1499	富士見堂クリニック	176-0021	東京都練馬区貫井3-14-6	03-5848-5987	http://www.fujimido-clinic.com	○	○	内科	榎本信行	
1500	二葉医院	177-0031	東京都練馬区三原台１－１－１７	03-3922-1466	http://www.futaba-iin.com		○	内科<br>小児科	野上　眞	
1501	間遠医院	179-0073	東京都練馬区田柄4-27-23	03-3975-1550	https://sites.google.com/site/madoclinic/	○	○	内科<br>消化器科<br>循環器科<br>肛門科	間遠　一成<br>須郷　亜紀子<br>間遠　陽子	
1502	丸山整形外科	178-0063	東京都練馬区東大泉4-27-40-2F	03-3978-8555	http://www.maruyama-seikei.jp/	○	○	整形外科	丸山徹雄	順天堂大学付属練馬病院
1503	水谷内科呼吸器科クリニック	178-0063	東京都練馬区東大泉6-51-4TKマンション1階	03-3867-8141	www.mizutani-clinic.com	○	○	内科	水谷清二	

電話や情報通信機器を用いて診療を実施する医療機関の一覧（東京都）

	基本情報					事務連絡に基づく対応について				
	施設名	郵便番号	住所（都道府県から記載）	電話番号	ウェブサイトURL	初診の電話等を用いた診療の実施の有無	再診の電話等を用いた診療の実施の有無	対応診療科	担当医師名	対面診療が必要と判断した場合に連携する医療機関名（複数ある場合は複数、住所も併せて記載）
1504	宮園内科クリニック	179-0075	東京都練馬区高松１－４２－２０	03-3825-1001	http://www.miyazono-clinic.or.jp		○	内科	宮園　裕子	練馬光が丘病院（東京都練馬区光が丘2-11-1）<br>順天堂練馬病院（東京都練馬区高野台3-1-10）
1505	宮本外科内科	179-0082	東京都練馬区錦2-8-5	03-3933-5656		○	○	内科	宮本　成基	豊島病院（東京都豊島区）
1506	山川クリニック	178-0063	東京都練馬区東大泉７－３８－５	03-3867-1885	https://yamakawa-clinic.biz	○	○	内・外科・脳外・神経内科・整形外科	山川健太	
1507	わだファミリークリニック	177-0035	東京都練馬区南田中３－７－２９	03-6915-9156	http://www.wada-family-clinic.jp/		○	神経内科　内科<br>小児科	和田　圭一郎<br>和田　万里子	
1508	医療法人社団こいし会<br>関町ゆいクリニック	177-0051	東京都練馬区関町北2-26-11	03-6766-0808	https://www.sekimachiyuiclinic.com/	○	○	内科	吉田　聡	
1509	医療法人社団ナイズ<br>キャップスクリニック光が丘	179-0072	東京都練馬区光が丘2丁目10番2号<br>光が丘IMA東館　2階	03-6904-0330	https://caps-clinic.jp/hikarigaoka/	○	○	小児科	吉田　聡	
1510	テル皮膚科	176-0023	東京都練馬区中村北1－10－12	03-3577-5100	https://teru-hifuka.com/	○	○	皮膚科	東芝　輝臣	
1511	医療法人社団育陽会<br>練馬さくら病院	179-0081	東京都練馬区北町3-7-19	03-3931-1101	http://www.t-sakurahosp.com/		○	内科<br>精神科	三橋　将人<br>髙野　徹<br>在宅担当医	
1512	旭医院	120-0026	東京都足立区旭町９-15	03-3888-3508	http://www.asahi-iin.jp/	○	○	内科・胃腸内科・外科	青木　哲	

電話や情報通信機器を用いて診療を実施する医療機関の一覧（東京都）

	基本情報					事務連絡に基づく対応について				
	施設名	郵便番号	住所（都道府県から記載）	電話番号	ウェブサイトURL	初診の電話等を用いた診療の実施の有無	再診の電話等を用いた診療の実施の有無	対応診療科	担当医師名	対面診療が必要と判断した場合に連携する医療機関名（複数ある場合は複数、住所も併せて記載）
1513	あだち共生病院	120-0855	東京都足立区本木南町27-6	03-3880-2777			○	内科	梶原・井關	
1514	足立慶友眼科	120-0015	東京都足立区足立1-12-12足立メディカルモール2F	03-5845-8200	https://keiyu-ganka.com/	○	○	眼科	清水　努	
1515	足立慶友整形外科	120-0015	東京都足立区足立1-12-12　足立メディカルモール3F	03-5845-8900	https://clinic.adachikeiyu.com/		○	整形外科<br>リウマチ科	北城　雅照	
1516	井上てるクリニック	121-0813	東京都足立区竹ノ塚5-7-10森屋ビル2F	03-5851-7666	https://inoueteru.jp/	○	○			
1517	医療法人社団　綾瀬病院	120-0005	東京都足立区綾瀬6丁目3番1号	03-3605-3319	http://ayasehp.la.coocan.jp/		○	内科・精神科	担当医師	
1518	医療法人社団叡美会　えのもとファミリークリニック	120-0038	東京都足立区千住橋戸町1-13　ポンテポルタ千住4F	03-6806-2471	https://enomoto-fcl.jp/	○	○	内科<br>小児科	榎本あき矢	東京女子医科大学東医療センター<br>（東京都荒川区西尾久2-1-10）<br>東京慈恵会医科大学葛飾医療センター<br>（東京都葛飾区青戸6-41-2）<br>東部地域病院（東京都葛飾区西亀有5-14-1）
1519	医療法人社団三奉会井上クリニック	121-0813	東京都足立区竹の塚5丁目11番8号	03-3850-5771	http://www.inouehsp.or.jp		○	内科	早川貴美子	
1520	医療法人社団　俊和会寺田病院	123-0873	東京都足立区扇1-20-12	03-3898-5231	https://terada-hospital.or.jp/		○	内科、外科、胃腸内科、大腸・肛門外科、消化器内科（内視鏡）、整形外科、婦人科、泌尿器科	寺田俊明、澤井廣量、葛岡健太郎、堀孝吏、田中良明、最上恭至、高石祐子、神山剛一、竹本俊二、中村浩他	
1521	医療法人社団仁幸会扇大橋病院	123-0873	東京都足立区扇1-55-28	03-3890-1171	https://ougioohashi-hp.com/		○	内科	金光　裕仁	

電話や情報通信機器を用いて診療を実施する医療機関の一覧（東京都）

	基本情報					事務連絡に基づく対応について				
	施設名	郵便番号	住所（都道府県から記載）	電話番号	ウェブサイトURL	初診の電話等を用いた診療の実施の有無	再診の電話等を用いた診療の実施の有無	対応診療科	担当医師名	対面診療が必要と判断した場合に連携する医療機関名（複数ある場合は複数、住所も併せて記載）
1522	医療法人社団心和会足立共済病院	120-0022	東京都足立区柳原１－３６－８	03-3881-6116	http://www.adachi-kyosai.com/		○	内科、外科	山本　学、水谷　央	
1523	医療法人社団中興会ミルディス内科胃腸科クリニック	120-0034	東京都足立区千住3-98千住ミルディスⅡ番館	03-3888-1871	http://www.milledix.com	○	○	内科<br>消化器内科	中村治道<br>吉汲　祐加子	
1524	医療法人社団なずな会池上レディースクリニック	121-0823	東京都足立区伊興５－６－８	03-5838-0228	http://www.ikegami-lady.com		○	婦人科	池上　芳美	
1525	医療法人社団　日岩会下井病院	120-0005	東京都足立区綾瀬３－２８－８	03-3620-8811	http://www.shimoi.or.jp/	○	○	整形外科<br>内科	金沢　輝久<br>下井　優一<br>下井　謙吾	
1526	医療法人社団大和会平成扇病院	123-0873	東京都足立区扇3-26-5	03-3855-5500	https://ougihp.jp/		○	精神科	日野 哲耶<br>山縣　文<br>松井　敏史<br>宮川　熱志	医療法人社団大和会　大内病院
1527	医療法人社団六扇会西新井江北クリニック	123-0845	東京都足立区西新井本町2-27-13・1階	03-5838-0833	https://rokusenkai.jp/		○	内科	前田　義智	
1528	大内病院	123-0841	東京都足立区西新井5-41-1	03-3890-1306	https://oouchihp.net/		○	精神科	各医師	
1529	おおつぼ小児科	123-0851	東京都足立区梅田6-31-11	03-3840-7773	http://otsubo-peds.com		○	小児科<br>アレルギー科	大坪　主税	
1530	おかのクリニック	123-0841	東京都足立区西新井1-11-4	03-3898-4983	https://www.okanoclinic.com/	○	○	ペインクリニック<br>内科	岡野隆利<br>岡野千恵美	

電話や情報通信機器を用いて診療を実施する医療機関の一覧（東京都）

	基本情報					事務連絡に基づく対応について				
	施設名	郵便番号	住所（都道府県から記載）	電話番号	ウェブサイトURL	初診の電話等を用いた診療の実施の有無	再診の電話等を用いた診療の実施の有無	対応診療科	担当医師名	対面診療が必要と判断した場合に連携する医療機関名（複数ある場合は複数、住所も併せて記載）
1531	北足立生協診療所	121-0836	東京都足立区入谷3-1-5	03-3896-9971	http://kitaadachi-clinic.info/		○	内科<br>小児科	渡邉 隆将	
1532	北綾瀬駅前せたｸﾘﾆｯｸ	120-0006	東京都足立区谷中2-5-10ｸｲｰﾝﾊｲﾂ浅伊2Ｆ	03-3628-8181	https://seta.clinic/		○	消化器内科<br>内科<br>内視鏡内科	瀬田真祐	東和病院(東京都足立区東和4-7-10)
1533	北川医院	120-0037	東京都足立区千住河原町22-6	03-3881-0364		○	○	内科<br>小児科	北川泰久<br>石川貴子<br>金山勇吾<br>竹川幸男	
1534	江北生協診療所	123-0872	東京都足立区江北2-24-1	03-3857-6636	https://www.kouhoku-clinic.com/		○	内科	渡邉　郁夫	
1535	江北ファミリークリニック	123-0872	東京都足立区江北5-1-1	03-3897-3030	http://www.kohoku-fc.com/		○	内科、小児科	杉村久理	
1536	小宮耳鼻咽喉科医院	120-0034	東京都足立区千住1-13-10	03-3881-0981		○	○	耳鼻咽喉科	小宮　尚	
1537	シオノアイクリニック	120-0006	東京都足立区谷中2-5-10	03-6802-6575	https://shiono-eye-clinic.com/	○	○	眼科	全医師	
1538	鹿浜診療所	123-0865	東京都足立区新田2-4-15	03-3912-8491	http://cl-shikahama.com/		○	内科<br>小児科	平山陽子	
1539	島袋内科	120-0026	東京都足立区千住旭町43-11	03-3882-9083	http://www7b.biglobe.ne.jp/shimabukuronaika/	○	○	内科	島袋　嘉修	

電話や情報通信機器を用いて診療を実施する医療機関の一覧（東京都）

	基本情報					事務連絡に基づく対応について				
	施設名	郵便番号	住所（都道府県から記載）	電話番号	ウェブサイトURL	初診の電話等を用いた診療の実施の有無	再診の電話等を用いた診療の実施の有無	対応診療科	担当医師名	対面診療が必要と判断した場合に連携する医療機関名（複数ある場合は複数、住所も併せて記載）
1540	成仁医院	120-0002	東京都足立区中川4-29-12	050-3734-5021	http://www.seijin.org/		○	精神科	担当曜日による	
1541	成仁病院	121-8515	東京都足立区島根3-2-1	050-3734-5401	http://www.seijin.org/		○	精神科	担当曜日による	
1542	椿１丁目内科クリニック	123-0871	東京都足立区椿１－６－１３	03-3890-1860	http://tsubakinaika.com		○	内科	大森　淳	
1543	東京ふれあい医療生活協同組合<br>ふれあいファミリークリニック	120-0047	東京都足立区宮城１－３３－２０	03-6908-4330	https://www.fureaico-op.biz/	○	○	内科<br>小児科	渡邊仁<br>角允博<br>倉信均<br>平原佐斗司<br>渡邉章	梶原診療所（東京都北区堀船3-29-9）<br>明理会中央総合病院（東京都北区東十条3-22-11）<br>王子生協病院（東京都北区豊島3-4-15
1544	特定医療法人社団潤恵会<br>敬仁病院	123-0865	東京都足立区新田二丁目18番6号	03-3913-3106	https://keijin-hospital.jp		○	内科・胃腸内科・外科・整形外科・腎臓内科･循環器内科	柳沼　道郎<br>柳沼　行宏<br>柳沼　征人<br>大樋　信之<br>柳沼　樹宏<br>柳沼　憲志	
1545	とねり耳鼻いんこう科・アレルギー科	121-0831	東京都足立区舎人１-１１-１６	03-3897-3387	https://toneri.jp/		○	耳鼻いんこう科、小児耳鼻いんこう科、アレルギー科	國井　直樹	
1546	とねり内科	121-0831	東京都足立区舎人1-11-10	03-6803-1168	https://toneri-naika.com/	○	○	内科<br>小児科	中田　亮	
1547	内科・脳神経外科<br>西原クリニック	123-0851	東京都足立区梅田5-25-33ロイヤルパークス梅島1Ｆ	03-5888-5288	http://www.nishihara-clinic.jp	○	○	内科<br>脳神経外科	西原哲浩	
1548	なかじま整形外科小児科クリニック	123-0862	東京都足立区皿沼1-16-5	03-5837-8070	https://www.nakajima-clinic-adachi.com/		○	小児科	中島　由佳	

電話や情報通信機器を用いて診療を実施する医療機関の一覧（東京都）

	基本情報					事務連絡に基づく対応について				
	施設名	郵便番号	住所（都道府県から記載）	電話番号	ウェブサイトURL	初診の電話等を用いた診療の実施の有無	再診の電話等を用いた診療の実施の有無	対応診療科	担当医師名	対面診療が必要と判断した場合に連携する医療機関名（複数ある場合は複数、住所も併せて記載）
1549	西新井駅前さくら参道内科クリニック	123-0843	東京都足立区西新井栄町２－３－３　２Ｆ	03-5888-6635	http://www.sakurasando-cl.jp		○	内科、腎臓内科、循環器科、リウマチ科	齋藤　督芸<br>齋藤　穎	
1550	野原耳鼻咽喉科医院	120-0026	東京都足立区千住旭町39-11	03-3870-2022	https://www.nohara-ent.com/	○	○	耳鼻咽喉科	野原　修	東京慈恵会医科大学附属病院（東京都港区西新橋3-19-18）
1551	深見医院	121-1301	東京都足立区伊興3-2-1	03-3897-1301	https://fukami-clinic.com	○	○	内科	深見　公一	
1552	ほつかクリニック	121-0072	東京都足立区保塚町18-15	03-3858-3822	https://hotsuka-cl.jp/	○	○	内科/糖尿病内科/循環器内科/消化器内科/皮膚科	各担当医師	
1553	ほつかこどもクリニック	121-0072	東京都足立区保塚町8-16　2階	03-5856-6930	https://hotsukakodomo.jp/	○	○	小児科	各担当医師	
1554	まちどりクリニック	120-0005	東京都足立区綾瀬3-6-2 ボナール16ビル4F	03-5673-0555	http://machidori-cl.com/		○	心療内科<br>精神科	待鳥　浩司<br>藤巻　純　青木淳一	
1555	山一ビル内科クリニック	121-0815	東京都足立区島根3-8-1　山一ﾋﾞﾙ島根Ⅱ1階	03-3884-8888	http://www.yamaichi-bldg-mc.com	○	○	内科<br>小児科	有野　亨<br>佐藤　敬太<br>土屋　昌史	
1556	山田耳鼻咽喉科医院	120-0003	東京都足立区東和2-24-4	03-3620-8880		○	○	耳鼻咽喉科	山田　一仁	
1557	横川レディースクリニック	121-0064	東京都足立区保木間１－２２－１５	03-3884-1241	https://www.yklc.jp		○	小児科	佐藤 御也子	横川レディースクリニック（東京都足立区1-22-15）

電話や情報通信機器を用いて診療を実施する医療機関の一覧（東京都）

	基本情報					事務連絡に基づく対応について				
	施設名	郵便番号	住所（都道府県から記載）	電話番号	ウェブサイトURL	初診の電話等を用いた診療の実施の有無	再診の電話等を用いた診療の実施の有無	対応診療科	担当医師名	対面診療が必要と判断した場合に連携する医療機関名（複数ある場合は複数、住所も併せて記載）
1558	渡辺整形外科	120-0013	東京都足立区弘道1-3-27	03-3840-1551	https://www.watanabe-clinic.or.jp/		○	整形外科<br>脳神経外科	渡邊寧<br>渡邊熙<br>田地野和宏	
1559	和田小児科医院	121-0812	東京都足立区西保木間2-15-23	03-3884-2301		○	○	内科<br>小児科	和田　紀之<br>和田　美穂	東京慈恵会医科大学付属病院（東京都港区西新橋3-9-18）<br>東京慈恵会医科大学葛飾医療センター（東京都葛飾区青戸6-41-2）
1560	医社）福寿会　慈英会病院	121-0843	東京都足立区西新井栄町2-8-6	03-3852-1111		○	○	内科	多井　晃	
1561	江北メンタルクリニック	123-0872	東京都足立区江北1-33-23ロイヤルＫＪ1階	03-5838-0797	https://www.kohoku-mental.com/		○	精神科	斎藤広生・中村馨	
1562	あさみファミリークリニック	125-0051	東京都葛飾区新宿3-15-2	03-3608-5577	https://asami-familyclinic.jp		○	内科<br>小児科	浅見育広	
1563	アリオ大島眼科	125-0061	東京都葛飾区亀有3-49-3アリオ亀有アリオモール2階	03-3602-2223	http://www.ario-oshima.com/		○	眼科	大島　真	
1564	医）いつき会ハートクリニック	124-0001	東京都小菅4-14-5レインボーヒルズ１Ｆ	03-3602-1810	httpps://www.heartclinic-kazuki.jp/online	○	○	循環器内科<br>内科<br>小児循環器<br>心臓血管外科	佐藤一樹	東部地域病院（東京都葛飾区西亀有5-14-1）<br>東京慈恵医科大学葛飾医療センター（東京都葛飾区青戸6-14-2）<br>平成立石病院（東京都葛飾区立石5-1-9）
1565	医療法人社団 一秀会 葛飾橋病院	125-0041	東京都葛飾区東金町7-33-1	03-3607-0891	http://www.katsushikabashi.jp/		○	精神科	尾内<br>柳田<br>杉山<br>桑本	
1566	医療法人社団　高志会<br>よつぎホームクリニック	124-0011	東京都葛飾区四つ木1-47-12　ハルメディカルビル3階	03-5698-7715	http://yotsugi-homeclinic.com/	○	○	内科<br>循環器内科<br>消化器内科<br>皮膚科<br>整形外科	丸山　晴久	日本医科大学付属病院（東京都文京区千駄ヶ谷1-1-5）

電話や情報通信機器を用いて診療を実施する医療機関の一覧（東京都）

	基本情報					事務連絡に基づく対応について				
	施設名	郵便番号	住所（都道府県から記載）	電話番号	ウェブサイトURL	初診の電話等を用いた診療の実施の有無	再診の電話等を用いた診療の実施の有無	対応診療科	担当医師名	対面診療が必要と判断した場合に連携する医療機関名（複数ある場合は複数、住所も併せて記載）
1567	医療法人社団さくら耳鼻咽喉科クリニック	124-0013	東京都葛飾区東立石3-25-12-101	03-5654-3387	http://sakurajibi.com/	○	○	耳鼻咽喉科	加賀田博子	
1568	医療法人社団讃紀会　オシダ眼科クリニック	124-0012	東京都葛飾区立石８丁目１－２　グレイスカメリア３０１号	03-3694-7123	http://www.oshida-eye-clinic.com	○	○	眼科	忍田　太紀	
1569	医療法人社団十有会中山耳鼻咽喉科	125-0061	東京都葛飾区亀有3ー7ー7サンセリテ鞠子301	03-3838-8733	www.nakajibi.or.jp	○	○	耳鼻咽喉科	中山一美<br>中山尚樹	
1570	医療法人社団十有会中山耳鼻咽喉科青戸医院	125-0062	東京都葛飾区青戸3-37-６holy.com3階	03ー5629ー8733	www.nakajibi.or.jp	○	○	耳鼻咽喉科	中山尚樹<br>中山一美	
1571	医療法人社団祥孝会柴又さくらクリニック	125-0052	東京都葛飾区柴又4-25-16 山本ビル1階	03-5693-1538	http://www.shohkohkai-clinic.com/shibamata/		○<br>電話のみ。条件あり。	皮膚科	溝口　雅子	
1572	医療法人社団双泉会いずみホームケアクリニック	125-0062	東京都葛飾区青戸5-30-4	03-3603-1717	http://www.sosenkai.or.jp	○	○	内科	豊田　千純子<br>堀元　寛美	
1573	医療法人社団智成会東金町内科クリニック	125-0041	東京都葛飾区東金町７－５－８　ロイヤルクレセント１階	03-5648-5715	http://www2s.biglobe.ne.jp/~ishigaki/HKC.htm	○	○	内科<br>小児科<br>消化器科	石垣　宏	イムス葛飾総合病院（東京都葛飾区西新小岩4丁目18-1）<br>東部地域病院（東京都葛飾区亀有5丁目14-1）
1574	医療法人社団藤福会　小沼医院	125-0033	東京都葛飾区東水元2-10-2　1F	03-3607-4008	http://www.onumaclnc.com	○	○	内科<br>消化器科<br>乳腺外科	加藤　孝男<br>加藤　満利子	東部地域病院（東京都葛飾区亀有五丁目14-1）<br>日本医科大学病院（東京都文京区千駄木1-1-5）
1575	医療法人社団直和会平成立石ペンギンクリニック	124-0012	東京都葛飾区立石5-7-3	03-3692-3131	http://www.heisei-tateishi.net/		○	内科<br>外科<br>整形外科<br>泌尿器科<br>脳神経外科<br>皮膚科	岩村　太郎<br>塩井　由美子	医療法人社団直和会　平成立石病院（東京都葛飾区立石5-1-9）

電話や情報通信機器を用いて診療を実施する医療機関の一覧（東京都）

	基本情報					事務連絡に基づく対応について				
	施設名	郵便番号	住所（都道府県から記載）	電話番号	ウェブサイトURL	初診の電話等を用いた診療の実施の有無	再診の電話等を用いた診療の実施の有無	対応診療科	担当医師名	対面診療が必要と判断した場合に連携する医療機関名（複数ある場合は複数、住所も併せて記載）
1576	医療法人社団ナイズ キャップスクリニック亀有	125-0061	東京都葛飾区亀有3-26-1 リリオ館6階	03-4579-2996	https://www.caps-clinic.jp/kameari	○	○	内科<br>小児科	和田　知博	
1577	医療法人社団博雅会大江医院	124-0004	東京都葛飾区東堀切2-5-15	03-3602-2096	https://www.ooe-iin.com	○	○	内科<br>小児科<br>消化器科	大江　毅	
1578	医療法人社団美昭会　たけなか眼科	125-0061	東京都葛飾区亀有5-20-11　Y.Sビルド1F	03-5613-1235			○	眼科	竹中　千昭<br>湊原　幸恵	
1579	医療法人社団裕栄会 高砂診療所	125-0054	東京都葛飾区高砂３－６－９－１０１	03-3659-3107	http://www.takasagocl.com		○	内科<br>小児科	浦田　栄吉	
1580	永寿堂医院	125-0061	東京都葛飾区亀有３－４３－５	03-3604-2101		○	○	小児科　内科	松永　貞一	
1581	えがおホームクリニック	125-0052	東京都葛飾区柴又5-33-2リバーサイドマンション401	03-6657-8658			○	内科<br>泌尿器科	中野　好<br>中野　透	
1582	おぎわら耳鼻咽喉科クリニック	125-0052	東京都葛飾区柴又1-4-10	03-3627-3341	https://www.ogiwara-ent-cl.com/		○	耳鼻咽喉科<br>小児科<br>気管食道科	荻原一郎	
1583	小澤病院	124-0022	東京都葛飾区奥戸2-31-3	03-3692-7610		○	○	内科	小澤	
1584	お花茶屋こどもクリニック	124-0005	東京都葛飾区宝町2-34-13-115	03-5654-9462	https://www.ohanajyaya-kodomo.jp/		○	小児科	永友　祥子	

電話や情報通信機器を用いて診療を実施する医療機関の一覧（東京都）

	基本情報					事務連絡に基づく対応について				
	施設名	郵便番号	住所（都道府県から記載）	電話番号	ウェブサイトURL	初診の電話等を用いた診療の実施の有無	再診の電話等を用いた診療の実施の有無	対応診療科	担当医師名	対面診療が必要と判断した場合に連携する医療機関名（複数ある場合は複数、住所も併せて記載）
1585	おんだ耳鼻咽喉科クリニック	125-0063	東京都葛飾区白鳥4-10-17コープみらい葛飾白鳥店2階	03-3690-6590	https://www.onchan-clinic.com/		○	耳鼻咽喉科<br>小児耳鼻咽喉科	恩田　信人	
1586	かつしか江戸川病院	125-0054	東京都葛飾区高砂3-27-13	03-3672-1046	http://katsushika-hp.com/		○	全診療科	各担当医	
1587	かつしか野村クリニック	125-0032	東京都葛飾区水元2-13-6福招会医療ビル1F	03-3826-5723	http://www.nomura-clinic.jp	○	○	内科<br>消化器科<br>小児科	野村　哲也	
1588	金町駅前脳神経内科<br>(名称変更手続き中)	125-0042	東京都葛飾区金町6-4-3　金町メディカルモール401	03-5660-5211	https://www.knoc.jp/		○	脳神経内科	内野勝行	新東京病院(千葉県松戸市和名ヶ谷1271)
1589	かなまち慈優クリニック	125-0041	東京都葛飾区東金町1-41-3 第5ウバノビル2階	03-3609-0133	http://www.jiyu-clinic.jp	○	○	内科<br>消化器内科	髙山　哲朗	
1590	金町診療所	125-0041	東京都葛飾区東金町1-15-5	03-3607-5124			○	内科	鈴木瑞史	
1591	金町成人病クリニック	125-0041	東京都葛飾区東金町２－13－４	03-3826-6171	https://www.kanja.jp/004183.html		○	内科	金哲宇	あさひ病院（東京都足立区平野１－２－３）
1592	かめありこどもクリニック	125-0061	東京都葛飾区亀有3-14-9　プリムロウズ島田２F	03-3602-3206	https://www.kanja.jp/017342html	○	○	小児科	角田　由理	
1593	亀有メディカルクリニック	125-0061	東京都葛飾区亀有3-26-1 リリオ館6階	03-6231-2905			○	内科<br>婦人科	塚田　健次	

電話や情報通信機器を用いて診療を実施する医療機関の一覧（東京都）

	基本情報					事務連絡に基づく対応について				
	施設名	郵便番号	住所（都道府県から記載）	電話番号	ウェブサイトURL	初診の電話等を用いた診療の実施の有無	再診の電話等を用いた診療の実施の有無	対応診療科	担当医師名	対面診療が必要と判断した場合に連携する医療機関名（複数ある場合は複数、住所も併せて記載）
1594	黒木整形外科内科クリニック	124-0021	東京都葛飾区細田3-29-15	03-3672-9611	http://www.kuroki-seikei.jp/	○	○	整形外科<br>内科	黒木　啓之<br>山田　直樹<br>谷　英明	
1595	小泉胃腸肛門クリニック	125-0042	東京都葛飾区金町6-4-3金町メディカルモール内102	03-3627-1300	https://koizumi-gip-clinic.com/	○	○	胃腸内科<br>肛門外科<br>乳腺科	小泉和也	
1596	佐久間医院	124-0023	東京都葛飾区東新小岩1-7-10	03-3694-3811		○	○	内科<br>耳鼻科	中原　國廣<br>岡村　洋沖	
1597	新葛飾ロイヤルクリニック	124-0006	東京都葛飾区堀切2-66-17	03-3697-8333	http://www.ims.gr.jp/katsushika-clinic/		○	内科<br>心臓血管外科<br>整形外科<br>脳神経外科<br>泌尿器科	鷲原　規喜	
1598	新小岩駅前総合クリニック	124-0024	東京都葛飾区新小岩2-1-1 リーフコンフォート新小岩3階	03-5678-5616	https://shinkoiwa.towakai.com/		○	内科	谷川　太志	
1599	新小岩北口診療所	129-0025	東京都葛飾区西新小岩1-8-4山口ビル１Ｆ	03-5670-2826			○	外科<br>内科	拝殿　清名	
1600	新小岩すばるクリニック	124-0024	東京都葛飾区新小岩2-1-1 リーフコンフォート新小岩2階	03-5678-6550	https://shinkoiwa-subaru.towakai.com/		○	耳鼻咽喉科	古沢　純	
1601	新小岩ゆり医院	124-0024	東京都葛飾区新小岩2-9-14　長尾第２ビル2Ｆ	03-5662-3301		○	○	耳鼻科	岡村　洋沖	
1602	新小岩わんぱくクリニック	124-0024	東京都葛飾区新小岩2-1-1 リーフコンフォート新小岩3階	03-6231-5512	https://shinkoiwa-wanpaku.towakai.com/		○	小児科	安井　孝二郎	

電話や情報通信機器を用いて診療を実施する医療機関の一覧（東京都）

	基本情報					事務連絡に基づく対応について				
	施設名	郵便番号	住所（都道府県から記載）	電話番号	ウェブサイトURL	初診の電話等を用いた診療の実施の有無	再診の電話等を用いた診療の実施の有無	対応診療科	担当医師名	対面診療が必要と判断した場合に連携する医療機関名（複数ある場合は複数、住所も併せて記載）
1603	鈴木内科・糖尿病クリニック	124-0025	東京都葛飾区西新小岩1-2-8第一鈴亀ビル4階	03-3694-1071	https://www.suzuki-naika.org	○	○	内科<br>糖尿病内科	鈴木一成<br>二見章子<br>橋本雅夫	東京臨海病院（東京都江戸川区臨海町）
1604	セリざわ医院	124-0003	東京都葛飾区お花茶屋2－15－5	03-3601-3361	https://www.serizawa-cl.com/	○	○	内科小児科	芹澤直紀	
1605	高木皮膚科医院	125-0061	東京都葛飾区亀有３－３３－４	03-3601-2218			○	皮膚科	高木道生・奈緒	
1606	高砂駅前消化器内科・外科クリニック	125-0054	東京都葛飾区高砂2-40-5-高砂駅前クリニックモール101	03-3657-0123	https://takasago.clinic/	○	○	内科・消化器	松浦芳文	
1607	ツルヤ外科内科クリニック	125-0061	東京都葛飾区亀有3-18-1鶴屋ビル2階	03-5876-6722	https://www.tsuruya-clinic.com/	○	○	内科	川村　純	東部地域病院（東京都葛飾区亀有五丁目14-1）
1608	東京慈恵会医科大学葛飾医療センター	125-8506	東京都葛飾区青戸6-41-2	03-3603-2111	http://www.jikei.ac.jp/hospital/katsushika/		○	外来診療を行う全ての診療科	左記診療科の外来担当医	
1609	中沢内科胃腸科医院	125-0042	東京都葛飾区金町6-9-2	03-3608-4976	https://www.nakazawa-naika.com		○	内科<br>胃腸科<br>小児科<br>皮膚科	中澤　幸弘<br>中澤　幸史	
1610	中村内科	125-0062	東京都葛飾区青戸3-39-15	03-3603-5501			○	内科<br>消化器科<br>循環器科	中村　正明	（公財）東京都保健医療公社　東部地域病院<br>（東京都葛飾区亀有5-14-1）
1611	新宿診療所	125-0052	東京都葛飾区柴又１－３７－５	03-3607-2850		○	○	内科<br>外科<br>整形外科	深澤　正樹	

電話や情報通信機器を用いて診療を実施する医療機関の一覧（東京都）

	基本情報					事務連絡に基づく対応について				
	施設名	郵便番号	住所（都道府県から記載）	電話番号	ウェブサイトURL	初診の電話等を用いた診療の実施の有無	再診の電話等を用いた診療の実施の有無	対応診療科	担当医師名	対面診療が必要と判断した場合に連携する医療機関名（複数ある場合は複数、住所も併せて記載）
1612	平木こどもクリニック	124-0014	東京都葛飾区東四つ木1-20-2	03-5671-0577	http://www.hiraki-kodomo-cl.com		○	小児科<br>内科	平木　洋子<br>平木　達朗	
1613	翠皮フ科・アレルギー科	125-0061	東京都葛飾区亀有3-37-17	03-6662-4232	http://midorihifuka.jp		○	皮膚科<br>アレルギー科	飯塚　仁	
1614	山口医院	125-0052	東京都葛飾区柴又４－１６－２	03-3657-3601	http:/yamaguchiiin.web.fc2.com/		○	内科	山口和彦	
1615	立石こころクリニック	124-0012	東京都葛飾区立石４－２６－９	03-3697-6488	https://tateishi-cocoro.com		○	精神科	熊倉荘一	
1616	かつしか心身総合クリニック	125-0041	東京都葛飾区東金町1-41-1桜井ビル2階	03-3627-0233	https://famille.or.jp/		○	内科<br>心療内科	駒形　清則<br>大川　昭宏<br>永井　斐子	
1617	医)東京育明会よつば皮膚科	132-0035	東京都江戸川区4-11-5平井イスズビル2階	03-3637-1112	http://www.ikumeikai.tokyo/yotsuba		○	皮膚科	三井　洋美	
1618	一之江けいゆう整形外科クリニック	132-0024	東京都江戸川区　一之江7-35-22　一富ビルディング3F	03-6905-9666	http://ku-seikei.com/	○	○	整形外科	酒井　均	
1619	医療法人社団あさかぜ会北小岩胃腸科クリニック	133-0051	東京都江戸川区北小岩4-8-3	03-3672-2865		○	○	内科	猪又　雄一	
1620	医療法人社団茜遥会<br>目々澤醫院	133-0051	東京都江戸川区北小岩4-5-8	03-3657-5470	http://www.memezawa.com/med	○	○	内科<br>神経内科	目々澤　肇	

電話や情報通信機器を用いて診療を実施する医療機関の一覧（東京都）

	基本情報					事務連絡に基づく対応について				
	施設名	郵便番号	住所（都道府県から記載）	電話番号	ウェブサイトURL	初診の電話等を用いた診療の実施の有無	再診の電話等を用いた診療の実施の有無	対応診療科	担当医師名	対面診療が必要と判断した場合に連携する医療機関名（複数ある場合は複数、住所も併せて記載）
1621	医療法人親和会　英診療所	132-0021	東京都江戸川区中央３－２０－１０	03-5661-5888	http://www.houei.or.jp	○	○	全診療科	各担当医師	
1622	医療法人社団つむぎ会守島医院	133-0057	東京都江戸川区西小岩1-16-6	03-3671-3711	https://www.morishima-iin.net/	○	○	内科	守島　亜季	
1623	医療法人社団津端会<br>京葉病院	132-0025	東京都江戸川区松江2-43-12	03-3654-8211	http://www.keiyo-hp.jp/	○	○	外科<br>整形外科<br>内科	津端　徹<br>津端　仁<br>平井　倫世<br>佐藤　利知子	東京臨海病院（東京都江戸川区臨海町1-4-2）<br>社会福祉法人仁生社江戸川病院（東京都江戸川区東小岩2-24-18）<br>東京ベイ浦安・市川医療センター（千葉県浦安市当代島3-4-32）
1624	医療法人社団同愛会病院	132-0031	東京都江戸川区松島1-42-21	0570-08-3311	https://www.douaikai.jp		○<br>薬の処方対応のみ	内科<br>外科<br>整形外科<br>眼科<br>泌尿器科<br>皮膚科	各担当医師	
1625	医療法人社団ナイズ<br>キャップスクリニック北葛西	134-0081	東京都江戸川区北葛西5-15-2	03-4579-0040	https://www.caps-clinic.jp/kitakasai	○	○	内科　小児科	森　博子	順天堂大学医学部附属 浦安病院（千葉県浦安市富岡２丁目１−１）<br>東京ベイ浦安市川医療センター（千葉県浦安市当代島３丁目４−３２）<br>東京臨海病院（東京都江戸川区臨海町１丁目４−２）<br>森山記念病院（東京都江戸川区北葛西４丁目３−１）<br>東京都立墨東病院（東京都墨田区江東橋４丁目２３−１５）
1626	医療法人社団ナイズ<br>キャップスクリニック西葛西	134-0088	東京都江戸川区西葛西6-12-1　関寅ビル第二	03-4579-9217	https://www.caps-clinic.jp/nishikasai	○	○	小児科	伊東　真隆	
1627	医療法人社団向日葵会<br>まつしま病院	132-0031	東京都江戸川区松島1-41-29	03-3653-5541	http://www.matsushima-wh.or.jp/		○	小児科<br>心療内科	益原千加<br>古池織恵	
1628	医療法人社団　結草会<br>みやのこどもクリニック	134-0085	東京都江戸川区南葛西2-18-27	03-3869-4133	https://www.miyano-kids.jp	○	○	小児科皮膚科	宮野孝一	
1629	葛西昌医会病院	134-8678	東京都江戸川区東葛西6-30-3	03-5696-1611	http://www.shoikai.com		○	外来診療科全て	各担当医師	

電話や情報通信機器を用いて診療を実施する医療機関の一覧（東京都）

	基本情報					事務連絡に基づく対応について				
	施設名	郵便番号	住所（都道府県から記載）	電話番号	ウェブサイトURL	初診の電話等を用いた診療の実施の有無	再診の電話等を用いた診療の実施の有無	対応診療科	担当医師名	対面診療が必要と判断した場合に連携する医療機関名（複数ある場合は複数、住所も併せて記載）
1630	くれもとクリニック	134-0081	東京都江戸川区北葛西4ー1ー45-102	03-5658-8777		○	○	小児科<br>内科	呉本　慶子	東京臨海病院（東京都江戸川区臨海町1-4-2）
1631	小岩すばるクリニック	133-0052	東京都江戸川区東小岩4-5-4 金本ビル2階	03-5693-3349	https://koiwa-subaru.towakai.com/		○	耳鼻咽喉科	國方　竜太郎	
1632	酒井内科・神経内科クリニック	146-0085	東京都江戸川区西葛西6-15-20 アイビーハイツ2F	03-6808-2807	https://sakai-cl.jp	○	○	内科<br>脳神経内科	酒井　宏一郎	
1633	さくらライフ江戸川クリニック	132-0021	東京都江戸川区中央4-11-8アルカディア親水公園ビル1F	03-6868-7851	http://www.slclinic.com/aboutus/edogawaclinic/	○		内科、精神科、心療内科	茂呂勝美<br>山口順嗣<br>渡会昌広<br>古坂隆幸	
1634	篠崎駅前クリニック	133-0061	東京都江戸川区篠崎町2-7-1	03-5666-1331	https://shinozaki-clinic.tums.jp/		○	内科	岡田　吉弘	
1635	篠崎駅前わんぱくクリニック	133-0061	東京都江戸川区篠崎町2-7-15　エスタシオン篠崎1階	03-5879-8871	https://shinozakiwanpaku-clinic.tums.jp/		○	小児科	川島　陽介	
1636	社会福祉法人仁生社　江戸川病院	133-0052	東京都江戸川区東小岩2-24-18	03-3673-1221	https://www.edogawa.or.jp/		○	全科	全医師	
1637	社会福祉法人仁生社　メディカルプラザ江戸川	133-0052	東京都江戸川区東小岩2-6-1	03-3673-4892	https://www.edogawa.or.jp/		○	全科	全医師	
1638	社会福祉法人仁生社<br>江戸川メディケア病院	133-0071	東京都江戸川区東松本2-14-12	03-3657-1181	https://edogawa-medicare.jp/		○	全外来診療科	各担当医師	

電話や情報通信機器を用いて診療を実施する医療機関の一覧（東京都）

	基本情報					事務連絡に基づく対応について				
	施設名	郵便番号	住所（都道府県から記載）	電話番号	ウェブサイトURL	初診の電話等を用いた診療の実施の有無	再診の電話等を用いた診療の実施の有無	対応診療科	担当医師名	対面診療が必要と判断した場合に連携する医療機関名（複数ある場合は複数、住所も併せて記載）
1639	社会医療法人社団森山医会　森山記念病院	134-0081	東京都江戸川区北葛西4-3-1	03-5679-1211	http://mk.moriyamaikai.or.jp/		○	脳神経外科<br>糖尿・内分泌内科<br>内科<br>大腸・肛門外科<br>整形外科<br>外科<br>循環器内科	対応診療科の常勤医と一部の非常勤医	自院にて対応
1640	社会医療法人社団森山医会森山脳神経センター病院	134-0088	東京都江戸川区西葛西7-12-7	03-3675-1211	http://mr.moriyamaikai.or.jp/		○	内科<br>脳神経外科<br>循環器内科<br>神経内科<br>内分泌科<br>泌尿器科<br>大腸肛門科	眼科を除くすべての外来診療担当医師	社会医療法人社団森山医会　森山記念病院（東京都江戸川区北葛西4-3-1）
1641	親和ハートフルクリニック	132-0035	東京都江戸川区平井2-15-15　アポロンビル2F	03-3684-7150	http://www.ikumeikai.tokyo/shinwa	○	○	内科	篠崎美樹子	
1642	東京みらいクリニック葛西	134-0083	東京都江戸川区中葛西5-34-8須賀ビル1F	03-3680-5560	https://tokyomiraiclinic-kasai.com/		○	小児科<br>内科	古屋　彩夏	
1643	にいほりクリニック	132-0001	東京都江戸川区新堀1-38-11	03-5243-4141	https://niihori-cl.jp/	○	○	内科/循環器内科/皮膚科	各担当医師	
1644	西葛西駅前総合クリニック	134-0088	東京都江戸川区西葛西3-15-13 第一江の本ビル3階	03-5679-7851	https://nishikasai.towakai.com/		○	内科	波多野　良二	
1645	西葛西わんぱくクリニック	134-0088	東京都江戸川区西葛西3-15-13 第一江の本ビル2階	03-5679-7851	https://nishikasai-wanpaku.towakai.com/		○	小児科	山田　浩之	
1646	東小岩げんきクリニック	133-0052	東京都江戸川区東小岩4-5-2 神奈川ビル2階	03-5612-3702	https://higashikoiwa-genki.towakai.com/		○	内科	山下　智子	
1647	東小岩わんぱくクリニック	133-0052	東京都江戸川区東小岩4-5-2 神奈川ビル1階	03-5612-3754	https://higasikoiwa-wanpaku.towakai.com/		○	小児科	小島　博之	

電話や情報通信機器を用いて診療を実施する医療機関の一覧（東京都）

	基本情報					事務連絡に基づく対応について				
	施設名	郵便番号	住所（都道府県から記載）	電話番号	ウェブサイトURL	初診の電話等を用いた診療の実施の有無	再診の電話等を用いた診療の実施の有無	対応診療科	担当医師名	対面診療が必要と判断した場合に連携する医療機関名（複数ある場合は複数、住所も併せて記載）
1648	平井駅前総合クリニック	132-0035	東京都江戸川区平井3-23-18 光井グランドハイツ2階	03-5875-3377	https://hirai.towakai.com/		○	内科	豊田　紀夫	
1649	平井すばるクリニック	132-0035	東京都江戸川区平井3-23-18 光井グランドハイツ1階	03-5875-1161	https://hirai-subaru.towakai.com/		○	耳鼻咽喉科	小島　慎平	
1650	船堀ゆり医院	134-0091	東京都江戸川区船堀1-4-13　ｼｬﾝｻｰﾙ船堀２Ｆ	03-5605-3301		○	○	耳鼻科	岡村　洋沖	
1651	松江病院	132-0025	東京都江戸川区松江2-6-15	03-3652-3121	https://matue.or.jp/		○	内科<br>外科<br>脳神経外科<br>胃腸外科<br>整形外科	山田　徹<br>宮本　竜之<br>宮崎　紀樹等	
1652	まなべファミリークリニック	134-0085	東京都江戸川区南葛西6-12-7	03-3869-1525	https://www.manabe-medical.com/	○	○	小児科　内科	眞鍋周太郎	
1653	瑞江すばるクリニック	133-0065	東京都江戸川区南篠崎町3-1-2 渡辺ビル1階	03-5636-1061	https://mizue-subaru.towakai.com/		○	耳鼻咽喉科	上村　隆一郎	
1654	瑞江総合クリニック	133-0065	東京都江戸川区南篠崎町3-1-2 渡辺ビル2階	03-5664-6588	https://mizue.towakai.com/		○	内科	松原　顕次	
1655	瑞江わんぱくクリニック	133-0065	東京都江戸川区南篠崎町3-1-2 渡辺ビル1階	03-3698-6531	https://mizue-wanpaku.towakai.com/		○	小児科	室伏　航	
1656	葛西こころのクリニック	134-0084	東京都江戸川区東葛西6-1-17-702	03-5878-0526	https://kasai-kokoro.com/		○	精神科、心療内科		

電話や情報通信機器を用いて診療を実施する医療機関の一覧（東京都）

	基本情報					事務連絡に基づく対応について				
	施設名	郵便番号	住所（都道府県から記載）	電話番号	ウェブサイトURL	初診の電話等を用いた診療の実施の有無	再診の電話等を用いた診療の実施の有無	対応診療科	担当医師名	対面診療が必要と判断した場合に連携する医療機関名（複数ある場合は複数、住所も併せて記載）
1657	西葛西メディカルクリニック	134-0088	東京都江戸川区西葛西6-18-3	03-3686-3637	http://nishikasai-cl.com	○	○	内科<br>循環器内科<br>糖尿病内科	田寺　長	
1658	イーアス高尾眼科	193-0834	東京都八王子市東浅川町550-1　イーアス高尾2F	042-673-5111	https://iiastakao.navi-clinic.jp/		○	眼科	宮本　桂一	
1659	市川内科クリニック	192-0072	東京都八王子市南町3-5シュゼル八王子1Ｆ	042-620-2272	http://ai-cli.jp/	○	○	内科	市川和人	
1660	医）岳　内科・循環器内科　クリニック髙田	192-0363	東京都八王子市別所2-2-1　1F	042-670-2039	http://www.clinic-takata.com/		○	内科のみ	髙田　博之	多摩南部地域病院（東京都多摩市）
1661	医）慈聖会　鈴木診療所	192-0904	東京都八王子市子安町2-10-14	042-642-3923	http://www.suzushin3923.jp/		○	内科<br>小児科	笹本和男<br>笹本優佳	
1662	（医社）古谷医院	192-0916	東京都八王子市みなみ野1-2-1アクロスモール八王子みなみ野2階	042-632-6866	https://furuya-iin.com/		○	内科<br>小児科	古谷　利通	東京医科大学八王子医療センター（東京都八王子市館町1163番地）
1663	いしづか内科クリニック	193-0832	東京都八王子市散田町3-13-6	042-668-0841			○	内科	石塚　太一	
1664	いずみクリニック	192-0904	東京都八王子市子安町1-11-9	042-649-4032	http://www.izumiclinic.jp/	○	○	内科<br>脳神経内科<br>小児科	堀内泉	
1665	医療法人社団松濤会　伊藤内科クリニック	192-0046	東京都八王子市明神町4-2-7 秀和第一八王子レジデンス１０２	042-644-2770	http://netsite.co.jp/dr_itoh/		○	内科	伊藤　秀二	

電話や情報通信機器を用いて診療を実施する医療機関の一覧（東京都）

	基本情報					事務連絡に基づく対応について				
	施設名	郵便番号	住所（都道府県から記載）	電話番号	ウェブサイトURL	初診の電話等を用いた診療の実施の有無	再診の電話等を用いた診療の実施の有無	対応診療科	担当医師名	対面診療が必要と判断した場合に連携する医療機関名（複数ある場合は複数、住所も併せて記載）
1666	医療法人社団　玉栄会<br>東京天使病院附属駅前クリニック	192-0046	東京都八王子市明神町4-6-2<br>シャンボール京王八王子１階	042-656-0075	https://angelcourt.or.jp/clinic/		○	内科	中村　昌平<br>今西　愿<br>高崎　雄司<br>坂巻　文雄<br>近藤　祐介<br>星川　賀織<br>岡部　多加志	
1667	医療法人社団おなか会おなかクリニック	192-0083	東京都八王子市旭町１２－１２	042-644-1127	https://www.m-onaka.com	○	○	内科<br>外科<br>肛門外科	村井 隆三<br>羽田 文紀<br>小幡 和彦	
1668	医療法人社団ＫＮＩ<br>北原リハビリテーション病院	192-0012	東京都八王子市左入町461	042-692-3332			○	リハビリテーション科	西谷　和敏<br>檀　充	北原国際病院<br>北原ライフサポートクリニック
1669	医療法人社団KNI北原国際病院	192-0045	東京都八王子市大和田町一丁目7番23号	042-645-1356	https://kokusai.kitaharahosp.com/		○	脳神経外科<br>循環器内科<br>神経内科<br>精神科<br>消化器内科	各診療科担当医	
1670	医療法人社団KNI北原ライフサポートクリニック	192-0904	東京都八王子市子安町4-7-1サザンスカイタワー八王子1階	042-655-6665	https://ls.kitaharahosp.com/		○	脳神経外科<br>総合診療科<br>小児科<br>神経内科<br>消化器外科<br>心臓血管外科<br>精神科<br>整形外科	各診療科担当医	
1671	医療法人社団青雲会<br>北野台病院	192-0911	東京都八王子市打越町1068番地	042-637-1001	http://www.kitanodai.jp/		○	精神科	市川<br>曽根<br>荻<br>川本<br>加茂<br>大谷	
1672	医療法人社団清仙会<br>松本クリニック	193-0835	東京都八王子市千人町2-20-2クローバービル3階303	042-673-4366	http://www.seisenkai.or.jp/	○	○	内科<br>整形外科<br>精神科	松本清彦他	ほりごめクリニック（八王子市千人町2-20-2-301）
1673	医療法人社団斗南堂<br>八王子クリニック	192-0081	東京都八王子市横山町11-5斗南堂ビル	042-643-3717	https://hachicli.or.jp/outpatient/hachicli.html	○	○	内科<br>肛門科<br>皮膚科	井藤　尚文<br>髙橋　克之	東京医科大学八王子医療センター（東京都八王子市館町1163）<br>東海大学医学部付属八王子病院（東京都八王子市石川町1838）
1674	医療法人社団斗南堂<br>八王子クリニック	192-0065	東京都八王子市新町7-10シルバーヒルズ八王子1F,2F	042-643-1321	https://hachicli.or.jp/outpatient/hachicli-shincho.html	○	○	内科<br>脳神経内科<br>循環器内科	新井　謙<br>中村　高浩<br>瀬戸口　雅彦	東京医科大学八王子医療センター（東京都八王子市館町1163）<br>東海大学医学部付属八王子病院（東京都八王子市石川町1838）

電話や情報通信機器を用いて診療を実施する医療機関の一覧（東京都）

	基本情報					事務連絡に基づく対応について				
	施設名	郵便番号	住所（都道府県から記載）	電話番号	ウェブサイトURL	初診の電話等を用いた診療の実施の有無	再診の電話等を用いた診療の実施の有無	対応診療科	担当医師名	対面診療が必要と判断した場合に連携する医療機関名（複数ある場合は複数、住所も併せて記載）
1675	医療法人社団ニューロアソシエイツ長池脳神経内科	192-0363	東京都八王子市別所1-75-3	042-678-7360	https://neuroassociates.jp		○	脳神経内科	丹羽 直樹	
1676	医療法人社団八王子中央診療所	192-0053	東京都八王子市八幡町5-11	042-626-5591		○	○	内科<br>小児科	山田　真	
1677	医療法人社団 朋樹会<br>御殿山クリニック	192-0375	東京都八王子市鑓水428番地160	042-677-1500	http://www.gotenyama-cl.com/		○	内科	工藤　樹彦	
1678	医療法人社団　明和会<br>キタクリニック	192-0046	東京都八王子市明神町3-22-10　守屋ビル3Ｆ	042-645-9000	http://www.ims-site.jp/kita_clinic/		○	精神科<br>心療内科	高橋	
1679	医療法人社団明和会<br>こころのクリニック<br>イムスハ王子	192-0082	東京都八王子市東町1-10 グランデハイツ八王子101	042-649-8221	https://www.ims.gr.jp/kokocli8/		○	精神科<br>心療内科	山田　西元<br>星加　青木	
1680	医療法人社団　明和会<br>西八王子病院	192-0151	東京都八王子市上川町2150番地	042-654-4551	http://www.ims.gr.jp/nishihachi-hp/		○	精神科	三根<br>高島<br>水尾<br>平木<br>宮坂<br>西元	
1681	医療法人社団めぐみ会<br>南大沢メディカルプラザ	192-0364	東京都八王子市南大沢2-25　フォレストモール3階	042-670-2460	https://www.m-medicalplaza.com/		○	内科		
1682	医療法人社団めぐみ会<br>南大沢メディカルプラザ２	192-0364	東京都八王子市南大沢2-25　フォレストモール2階	042-670-5922	https://www.m-medicalplaza.com/mp2/		○<br>電話のみ（条件付き）	整形外科<br>小児科	全医師	
1683	医療法人隆雅会伊藤内科消化器医院	192-0045	東京都八王子市大和田町4-15-14	042-642-6734	https://www.ito-iin.jp/		○	内科	伊藤　均	

電話や情報通信機器を用いて診療を実施する医療機関の一覧（東京都）

	基本情報					事務連絡に基づく対応について				
	施設名	郵便番号	住所（都道府県から記載）	電話番号	ウェブサイトURL	初診の電話等を用いた診療の実施の有無	再診の電話等を用いた診療の実施の有無	対応診療科	担当医師名	対面診療が必要と判断した場合に連携する医療機関名（複数ある場合は複数、住所も併せて記載）
1684	岩本脳神経クリニック	192-0911	東京都八王子市打越町1197-1はけしたビルF棟4階	03-3923-5515	042-632-5585		○	脳神経外科<br>神経内科<br>内科	岩本邦憲	対面診療不可
1685	永生クリニック	193-0942	東京都八王子市椚田町588-17	042-661-7780	eisei.or.jp		○	内科	金子　弥樹	
1686	大島医院	192-0904	東京都八王子市子安町3-5-9	042-642-1932		○	○	内科<br>小児科	大島一太	東京医大八王子医療センター・東海大学八王子病院
1687	加藤醫院	192-0919	東京都八王子市七国4-9-3	042-632-7950	http://www.katoiin.com/	○	○	小児科　内科<br>アレルギー科	加藤　直樹<br>齊木　裕香	
1688	かとう耳鼻科クリニック	192-0045	東京都八王子市大和田町5-13-15	042-642-7744			○	耳鼻咽喉科	加藤　晴弘	東海大学医学部付属八王子病院(東京都八王子市石川町１８３８)<br>東京医科大学八王子医療センター(東京都八王子市館町１１６３)
1689	金子内科クリニック	192-0083	東京都八王子市旭町6-6<br>ピオスビル4階	042-621-5240			○	内科	金子　健蔵	
1690	亀谷診療所	192-0362	東京都八王子市松木48-10　グランドゥール1階	042-689-5959			○	内科	亀谷　学	
1691	かわだ眼科	192-0364	東京都八王子市南大沢２－２パオレビル５階	042-689-6037	kawada-ganka.jp/		○	眼科	河田英一郎	
1692	北野小児科	192-0906	東京都八王子市北野町545-3北野タウンビル6階	042-645-8715	http://kitasyou.starfree.jp	○	○	小児科<br>内科	廣田保蔵	北野小児科

電話や情報通信機器を用いて診療を実施する医療機関の一覧（東京都）

	基本情報					事務連絡に基づく対応について				
	施設名	郵便番号	住所（都道府県から記載）	電話番号	ウェブサイトURL	初診の電話等を用いた診療の実施の有無	再診の電話等を用いた診療の実施の有無	対応診療科	担当医師名	対面診療が必要と判断した場合に連携する医療機関名（複数ある場合は複数、住所も併せて記載）
1693	クリニックゼロ	193-0943	東京都八王子市寺田町490	042-666-1556	https://www.eisei.or.jp/		○	内科	石塚　英夫	
1694	小松整形外科	192-0045	東京都八王子市大和田町5-30-28	042-642-4017			○	整形外科、内科	小松　隆	
1695	小宮メディカルクリニック	192-0031	東京都八王子市小宮町1165-2東亜建設第3ビル1階	042-639-0666	http://www.komiya-mc.com		○	内科	牛川憲司	
1696	坂本クリニック	193-0836	東京都八王子市日吉町4-20	042-622-2601	www.sakamoto-clinic.net	○	○	内科<br>小児科外科	坂本直隆<br>宮薗光	東京医科大学八王子医療センター（東京都八王子市館町1163）<br>南多摩病院（東京都八王子市散田町3-10-1）
1697	ささき医院	192-0352	東京都八王子市大塚496	090-1439-7766	http://minamiosawa.xsrv.jp/	○	○	内科<br>小児科	佐々木　哲三	ささき医院（東京都八王子市大塚496）、南大沢クリニック（東京都八王子市南大沢5-14-4-1）
1698	三愛病院	192-0005	東京都八王子市宮下町377番地	042-691-4111	http://www.sanai-hosp.jp/		○	内科	各医師	
1699	島田療育センターはちおうじ	193-0931	東京都八王子市台町4丁目33番地の13	042-634-8511	https://www.shimada-ryoiku.or.jp/shima8/		○	療育<br>小児科	小沢浩<br>小沢愉理<br>河野千佳<br>井之上寿美<br>河野芳美<br>塩田睦記<br>中村由紀子<br>松岡雄一郎	
1700	しんや内科	1;93-0835	東京都八王子市千人町4丁目13-2	042-673-3033	https://www.shinya-naika.com		○	内科	新谷　英滋	南多摩病院（東京都八王子市散田町3-10-1）<br>東海大学医学部付属八王子病院（東京都八王子市石川町1838）<br>東京医科大学八王子医療センター（東京都八王子市館町1163）
1701	仁和会総合病院	192-0046	東京都八王子市明神町4-8-1	042-644-3711	http://www.jinwakai.jp/		○	全科	全科	

電話や情報通信機器を用いて診療を実施する医療機関の一覧（東京都）

	基本情報					事務連絡に基づく対応について				
	施設名	郵便番号	住所（都道府県から記載）	電話番号	ウェブサイトURL	初診の電話等を用いた診療の実施の有無	再診の電話等を用いた診療の実施の有無	対応診療科	担当医師名	対面診療が必要と判断した場合に連携する医療機関名（複数ある場合は複数、住所も併せて記載）
1702	菅原脳神経外科クリニック	192-0903	東京都八王子市万町175-1　D棟	042-622-3000	https://sugawaraclinic.jp/	○	○	内科<br>脳神経外科	菅原道仁<br>伊藤たえ	
1703	スマイルこどもクリニック	193-0832	東京都八王子市散田町5-4-20	042-661-5529	https://smile-child-jesus.com	○	○	小児科	三輪久美子	
1704	田島医院	192-0023	東京都八王子市久保山町2-43-2	042-691-7550	作成中	○	○	内科・整形外科	窪田信行	
1705	田中医院	193-0942	東京都八王子市椚田町249-1	042-665-0221		○	○	内科<br>神経内科	田中伸幸<br>田中幸男	
1706	田中内科医院	192-0046	東京都八王子市明神町2-11-1	042-645-6144		○	○	内科	田中　浩三	東京医科大学八王子医療センター（八王子市館町1163）<br>東海大学医学部付属八王子病院（八王子市石川町1838）
1707	知野整形外科医院	192-0081	東京都八王子市横山町20-15ノモス八王子101	042-643-0073	http://www014.upp.so-net.ne.jp/chino/		○	整形外科	知野　公明	南多摩病院
1708	辻野クリニック	193-0832	東京都八王子市散田町3-8-24茂和4階	042-666-7064	https://www.hospita.jp/detail/86/		○	泌尿器科	辻野　進	なし
1709	てんじん内科外科クリニック	193-0934	東京都八王子市小比企480-1	042-632-8751	http://wwwtenjinn…–mcjp		○	内科外科	天神　敏博	
1710	中濵クリニック	192-0375	東京都八王子市鑓水2-175-9	042-676-1234	https://www.nakahama-clinic.com/	○	○	内科<br>小児科	中濵　昌夫	

電話や情報通信機器を用いて診療を実施する医療機関の一覧（東京都）

	基本情報					事務連絡に基づく対応について				
	施設名	郵便番号	住所（都道府県から記載）	電話番号	ウェブサイトURL	初診の電話等を用いた診療の実施の有無	再診の電話等を用いた診療の実施の有無	対応診療科	担当医師名	対面診療が必要と判断した場合に連携する医療機関名（複数ある場合は複数、住所も併せて記載）
1711	ながふさ共立診療所	193-0824	東京都八王子市長房町1462-5	042-664-1005	http://kita.kyoritsu-clinic.jp		○	内科<br>小児科<br>整形外科	松田文子<br>高柳　新<br>井出勝久<br>安達美菜<br>寺田　穣<br>西川洋平	
1712	西てらかた医院	192-0153	東京都八王子市西寺方町383-1	042-650-5055	http://www.zuishoukai.or.jp/nishiterakata/?gclid=Cj0KCQjws_r0BRCwARIsAMxfDRh7KoHh0uX6kHC5Qq35u9FlwPWvQ3ao0eDE_sBtsUKAyatS9up4XxMaAseEEALw_wcB	○	○	内科<br>整形外科<br>眼科	向井田　智之	
1713	弐番街メディカルクリニック	192-0373	東京都八王子市上柚木3-6-1	042-670-7288	http://www.2ndamc.or.jp	○	○	内科<br>外科<br>小児科<br>整形外科	板岡　俊成	弐番街メディカルクリニック(東京都八王子市上柚木3-6-1)
1714	沼沢医院	192-0904	東京都八王子市子安町4-20-9	042-622-2369			○	内科	沼沢　良樹	
1715	八王子山王病院	192-0042	東京都八王子市中野山王2-15-16	042-626-1144	https://hachioji-sannou.or.jp/		○	標榜科全て	各担当医	
1716	八王子消化器病院	192-0903	東京都八王子市万町177-3	042-626-5111	http://www.hachiojisyokaki.com/		○	消化器内科<br>消化器外科<br>糖尿病内科<br>リウマチ科	小池　伸定	
1717	八王子糖尿病内科クリニック	192-0083	東京都八王子市旭町11-5 CP6ビル3F	042-631-1024	http://hachioji-dm.com		○	糖尿病内科	山本　直之	
1718	原内科医院	193-0931	東京都八王子市台町2-22-7-102	042-625-7133			○	内科	原　歩	東京医大八王子医療センター（東京都八王子市館町1163）
1719	福原内科クリニック	192-0071	東京都八王子市八日町4-16	042-623-3238	https://www.fukuhara-naika.com/	○	○	内科<br>小児科	今西　亮	

電話や情報通信機器を用いて診療を実施する医療機関の一覧（東京都）

	基本情報					事務連絡に基づく対応について				
	施設名	郵便番号	住所（都道府県から記載）	電話番号	ウェブサイトURL	初診の電話等を用いた診療の実施の有無	再診の電話等を用いた診療の実施の有無	対応診療科	担当医師名	対面診療が必要と判断した場合に連携する医療機関名（複数ある場合は複数、住所も併せて記載）
1720	冨士森内科クリニック	193-0931	東京都八王子市台町2-14-20	042-621-0300	http://www.fujimori.sms.gr.jp/	○	○	内科<br>消化器内科<br>内視鏡内科<br>循環器内科　リウマチ科	清川重人<br>清川智史<br>清川博史<br>髙嶋志在<br>小笠原倫大<br>服部伸洋<br>坂本直人<br>山崎隼人	南多摩病院（東京都八王子市散田町3-10-1）<br>東海大学医学部付属八王子病院（東京都八王子市石川町1838）<br>東京医科大学八王子医療センター（東京都八王子市館町1163）
1721	冨士森内科みなみのクリニック	192-0917	東京都八王子市西片倉3-1-21第1みなみ野クリニックセンター2F	042-635-6711	http://fujimori-minamino.com	○	○	内科	髙嶋志在<br>清川重人<br>箕輪健太郎	
1722	ふれあいつつじヶ丘診療所	193-0823	東京都八王子市横川町668-69	042-626-0311			○	内科	原　政晴	
1723	ほりごめクリニック	193-0835	東京都八王子市千人町2-20-2クローバービル3階301	042-673-7004		○	○	内科	堀米政利他	医療法人社団清仙会松本(八王子市千人町2-20-2-303)
1724	まつもと小児ｱﾚﾙｷﾞｰｸﾘﾆｯｸ	192-0364	東京都八王子市南大沢2　パオレ5F	042-679-6051	http://ssc2.doctorqube.com/macc/		○	小児科<br>アレルギー科	松本勉	東海大学医学部付属八王子病院<br>東京医科大学八王子医療センター<br>日本医科大学多摩永山病院<br>多摩南部地域病院
1725	真宮病院	192-0075	東京都八王子市南新町23	042-625-0648	http://www.mamiya-hp.jp/		○	内科<br>整形外科科	真宮一<br>浜野昭彦	
1726	右田病院	192-0043	東京都八王子市暁町１丁目４８番１８号	042-622-5155	http://www.migitahosp.or.jp/		○	外科<br>乳腺外科<br>整形外科<br>内科	右田　隆之<br>鴨　宣之<br>森島　満<br>蒔苗　永	
1727	みなみ野眼科クリニック	192-0917	東京都八王子市西片倉3-1-21　サザンクロス2-2	042-632-5888	https://www.minamino-cc.com/		○	眼科	藤田　哲	
1728	みなみ野こどもクリニック	192-0917	東京都八王子市西片倉3-1-4 第2みなみ野クリニックセンター3階	042-637-5151	http://minamino-kids.clinic	○	○	小児科<br>アレルギー科	檜垣　博嗣	

電話や情報通信機器を用いて診療を実施する医療機関の一覧（東京都）

	基本情報					事務連絡に基づく対応について				
	施設名	郵便番号	住所（都道府県から記載）	電話番号	ウェブサイトURL	初診の電話等を用いた診療の実施の有無	再診の電話等を用いた診療の実施の有無	対応診療科	担当医師名	対面診療が必要と判断した場合に連携する医療機関名（複数ある場合は複数、住所も併せて記載）
1729	みなみ野循環器病院	192-0918	東京都八王子市兵衛1-25-1	042-637-8101	https://mjhospital.tokyo/		○	循環器内科	幡　芳樹	
1730	山田皮フ科クリニック	192-0917	東京都八王子市西片倉3-1-21サザンクロス1階	042-635-2551	http://www.y-hifuka.com/		○	皮膚科	山田　晴義	
1731	よしこ眼科クリニック	192-0918	東京都八王子市兵衛1-3-1ミクリスシティ4階	042-632-8008	https://www.yoshiko-eye.jp/		○	眼科	赤坂佳子	
1732	義澤皮膚科内科クリニック	192-0914	東京都八王子市片倉町342-3　K's片倉ビル2F	042-697-9923			○	内科	義澤　成美	
1733	わかばやし内科クリニック	192-0051	東京都八王子市元本郷町2-5-1	042-622-0550	https://wakabayashinaika.wixsite.com/clinic		○	内科<br>呼吸器内科<br>アレルギー科	若林徹	
1734	医療法人社団幸悠会　内野クリニック	190-0012	東京都立川市曙町　2丁目34番地6　小杉ビル6階	042-521-4139	http://www.kouyukai1968.or.jp		○	精神科	各医師	
1735	医療法人社団 敏和会 西砂川病院	190-0031	東京都立川市砂川町8-2-3	042-535-8811	http://www.nishisuna-hp.jp/		○	内科<br>耳鼻咽喉科	各医師	国家公務員共済組合連合会 立川病院<br>（東京都立川市錦町4-2-22）<br>独立行政法人国立病院機構災害医療センター<br>（東京都立川市緑町3256）
1736	医療法人社団颯由会今井皮フ形成外科クリニック	190-0012	東京都立川市曙町２－１３－３立川三菱ビル６F	0120-50-1103	https://www.imai-clinic.jp/	○	○	皮膚科<br>形成外科	今井　由典<br>大津　孝枝<br>朝山　祥子<br>山本　佐織	
1737	うちだ内科医院	190-0002	東京都立川市幸町5－95－4	042-535-3974	http://www.tamagawa-josui.com/	○	○	内科<br>小児科<br>皮膚科	鎌形　博展	

電話や情報通信機器を用いて診療を実施する医療機関の一覧（東京都）

	基本情報					事務連絡に基づく対応について				
	施設名	郵便番号	住所（都道府県から記載）	電話番号	ウェブサイトURL	初診の電話等を用いた診療の実施の有無	再診の電話等を用いた診療の実施の有無	対応診療科	担当医師名	対面診療が必要と判断した場合に連携する医療機関名（複数ある場合は複数、住所も併せて記載）
1738	おおたか脳神経外科・内科	190-0004	東京都立川市柏町4-56-1　ｸﾞﾛｰﾊﾞﾙﾋﾞﾙ1FA	042-535-1177	https://otaka-nouge.com/		○	脳神経外科・内科<br>内科	大高　弘稔	独立行政法人災害医療センター（東京都立川市緑町3256）<br>立川相互病院（東京都立川市緑町4-1）
1739	クリニックみらい立川	190-0023	東京都立川市柴崎町6-19-20<br>メディカル・フォレストたちかわ	042-525-7550	https://www.tama-mirai.com/tachikawa/index.php		○	内科	金重勝博	
1740	立川駅南泌尿器科皮膚科クリニック	190-0022	東京都立川市錦町2-1-33立川南口HMビル3階A号	042-548-8802	http://www.tachikawa-clinic.jp		○	泌尿器科<br>皮膚科<br>内科	湯澤　政行	
1741	立川相互病院	190-8578	東京都立川市緑町4-1	042-525-2585	https://www.t-kenseikai.jp/tachisou/		○	産婦人科<br>眼科<br>脳神経外科	佐藤　典子<br>長坂　康子<br>張　暁慧<br>池田　麗<br>菅原　知美<br>大久保　俊之<br>安部　友康<br>佐々木　正史	
1742	立川南口耳鼻咽喉科	190-0023	東京都立川市柴崎町3-10-5　大雅ビル1階	042-540-2917	http://tachikawa.sho-jin.com/	○	○	耳鼻科	庄司　育央	
1743	西立川おとなとこどものクリニック	190-0013	東京都立川市富士見町1-31-18<br>西立川KIビル２F	042-521-3333	https://nishitachikawa-otona-kodomo.jp/	○	○	内科	高橋　永子	
1744	まつもとクリニック	190-0022	東京都立川市錦町２－１－３３<br>立川南口HMビル２F	042-540-6766	https://www.dr-matsumotoclinic.com		○	内科<br>乳腺外科<br>外科<br>消化器内科<br>肛門外科	松本　匡浩	
1745	南眼科	190-0012	東京都立川市曙町2-13-3　立川三菱ビル5階	042-525-4981	http://www.minamiganka.jp/	○	○	眼科	南　修一郎	
1746	吉祥寺もろほし耳鼻咽喉科	180-0004	東京都武蔵野市吉祥寺本町一丁目７番４号<br>丸二清水ビル３階	0422-20-4133	http://www.morohosi-jibika.jp/jibika/kichijoji/	○	○	耳鼻咽喉科<br>アレルギー科	満山知恵子	

電話や情報通信機器を用いて診療を実施する医療機関の一覧（東京都）

	基本情報					事務連絡に基づく対応について				
	施設名	郵便番号	住所（都道府県から記載）	電話番号	ウェブサイトURL	初診の電話等を用いた診療の実施の有無	再診の電話等を用いた診療の実施の有無	対応診療科	担当医師名	対面診療が必要と判断した場合に連携する医療機関名（複数ある場合は複数、住所も併せて記載）
1747	あべ整形外科クリニック	180-0003	東京都武蔵野市吉祥寺南町１-6-18 ルネ吉祥寺B201 -A	0422-46-7251	http://www.abeseikei.com		○	整形外科　リウマチ科　リハビリテーション科	阿部　康裕	
1748	池下レディースクリニック吉祥寺	180-0004	東京都武蔵野市吉祥寺本町1-23-1 KS23ビル6・7F	0422-27-2965	http://www.ikeshita-clinic.com/kichijoji		○	産婦人科	矢野　直美	
1749	石澤敦クリニック	180-0004	東京都武蔵野市吉祥寺本町1-21-2-206	0422-20-0755	http://www.ishizawa-clinic.jp/	○	○	脳神経外科	石澤　敦	
1750	いちむら内科クリニック	180-0023	東京都武蔵野市境南町1-30-16	0422-39-4123			○	内科　呼吸器内科　　アレルギー科	市村　浩一	
1751	いるかこどもクリニック	180-0021	東京都武蔵野市桜堤2-7-27 メディシャトル桜堤102号	0422-52-1650	http://iruka-kodomo.jp/	○	○	小児科　アレルギー科	佐藤　大	
1752	おおやクリニック	180-0022	東京都武蔵野市境2-8-3	0422-51-3001	① http://www.clinics.medley.life/clinics/58ef45bdad12b80009a8fdef ② https://www.ooya-clinic.com/	○	○	内科　婦人科　小児科	田中　正司	
1753	かえで内科クリニック	180-0003	東京都武蔵野市吉祥寺南町2-17-2-102	0422-24-7065			○	内科	瀬浪　克彦	
1754	かたおか医院	180-0013	東京都武蔵野市西久保3-2-22 イノグリーンフォレスト1階	0422-50-5311	https://www.kataoka-iin.com/		○	内科	片岡　直之	
1755	かみやま内科クリニック	180-0022	東京都武蔵野市境1-9-9	0422-60-3188	http://www.kamiyama-cl.com		○	内科	神山　俊典	

電話や情報通信機器を用いて診療を実施する医療機関の一覧（東京都）

	基本情報					事務連絡に基づく対応について				
	施設名	郵便番号	住所（都道府県から記載）	電話番号	ウェブサイトURL	初診の電話等を用いた診療の実施の有無	再診の電話等を用いた診療の実施の有無	対応診療科	担当医師名	対面診療が必要と判断した場合に連携する医療機関名（複数ある場合は複数、住所も併せて記載）
1756	河原クリニック	180-0013	東京都武蔵野市西久保2-14-5	0422-55-7500	http://www.kawaharaclinic.jp/	○	○	整形外科・リウマチ科・皮膚科	河原　徹<br>伊藤　算昭<br>水谷　三記子	
1757	北町診療所	180-0001	東京都武蔵野市吉祥寺北町1-1-3	0422-22-8151	http://www.kichijoji-kitamachi-clinic.com	○	○	内科	藤川　恭浩	
1758	吉祥寺・藤田クリニック	180-0003	東京都武蔵野市吉祥寺南町１-1-10<br>MAビル7F	0422-40-5177	http://www.fujita-cl.jp/		○	内科　糖尿病内科　循環器内科　内分泌　甲状腺内科　腎臓内科	藤田　進彦<br>藤田　恵美子	
1759	吉祥寺あさひ病院	180-0004	東京都武蔵野市吉祥寺本町1-30-12	0422-22-1120	https://www.zenjinkai-group.jp//tojinkai/hospital/kichijoji/		○	腎臓内科　糖尿病内科　リウマチ科　循環器内科　泌尿器科　内科	各担当医師	
1760	吉祥寺クローバークリニック	180-0004	東京都武蔵野市吉祥寺本町2-25-7<br>吉祥寺プラザ3階	0422-28-7377	http://www.kichijoji-clover-clinic.jp		○	精神科	備瀬　哲弘	
1761	吉祥寺こころの診療所	180-0004	東京都武蔵野市吉祥寺本町1-18-2<br>M-9ビル7階	0422-27-1282	http://www.kj-kokoro.com		○	精神科	那須　一郎	
1762	吉祥寺榊原クリニック	180-0003	東京都武蔵野市吉祥寺南町１-8-6<br>カーサカルム吉祥寺2階	0422-48-6311		○	○	循環器内科<br>消化器内科　内科	酒井　吉郎<br>春木　京子	
1763	吉祥寺たかのメンタルクリニック	180-0004	東京都武蔵野市吉祥寺本町2-13-4<br>井野ビル301	0422-27-1077	https://takano-cl.jp		○	心療内科　精神科	高野　佳也	
1764	吉祥寺東方医院	180-0002	東京都武蔵野市吉祥寺東町1-2-2<br>オリエントプラザ丹野201号室	0422-21-7211			○	内科　皮膚科<br>アレルギー科	三浦　於菟	

電話や情報通信機器を用いて診療を実施する医療機関の一覧（東京都）

	基本情報					事務連絡に基づく対応について				
	施設名	郵便番号	住所（都道府県から記載）	電話番号	ウェブサイトURL	初診の電話等を用いた診療の実施の有無	再診の電話等を用いた診療の実施の有無	対応診療科	担当医師名	対面診療が必要と判断した場合に連携する医療機関名（複数ある場合は複数、住所も併せて記載）
1765	吉祥寺南病院	180-0003	東京都武蔵野市吉祥寺南町3-14-4	0422-45-2161	https://www.kichijoujiminami-hp.jp/		○	内科<br>外科<br>整形外科<br>脳神経外科	佐藤　俊一<br>阿南　匡<br>山下　重雄<br>藤井　正道<br>齋藤　良彦<br>高草木　宏之	
1766	吉祥寺南町眼科	180-0003	東京都武蔵野市吉祥寺南町2-4-12	0422-43-0055			○	眼科	瀧川　かおり	
1767	吉祥寺メディカルクリニック	180-0004	東京都武蔵野市吉祥寺本町2-13-4<br>井野ビル202号室	0422-23-2820	http://www.kichijoji-clinic.com		○	内科　消化器内科	高橋　正彦	
1768	吉祥寺レディースクリニック	180-0003	東京都武蔵野市吉祥寺南町2-6-10<br>富士パームビル5F	0422-40-1770	http://www.kichijyoji-ladies.com/		○	産婦人科	成川　希	
1769	木下循環器呼吸器内科クリニック	180-0004	東京都武蔵野市吉祥寺本町1-33-6	0422-22-8300	http://www5a.biglobe.ne.jp/~k-clinic/index.html		○	内科　呼吸器内科　循環器内科	木下　陽	
1770	甲賀クリニック	180-0023	東京都武蔵野市境南町3-12-2	0422-30-7707	http://www.kougaclinic.com	○	○	内科　小児科　皮膚科　アレルギー科　整形外科　リハビリテーション科　心療内科　漢方内科	甲賀　文晴	
1771	ごとう内科・循環器クリニック	180-0023	東京都武蔵野市境南町2-20-6	0422-32-5048	http://www.gotou-clinic.jp/		○	内科・循環器内科	後藤　依里	
1772	小林眼科医院	180-0004	東京都武蔵野市吉祥寺本町2-9-1<br>KYビル３F	0422-22-3507			○	眼科	小林　康紀	
1773	小森病院	180-0014	東京都武蔵野市関前3-3-15	0422-55-8311		○（診療録が残っている方のみ）	○	内科　小児科		

電話や情報通信機器を用いて診療を実施する医療機関の一覧（東京都）

	基本情報					事務連絡に基づく対応について				
	施設名	郵便番号	住所（都道府県から記載）	電話番号	ウェブサイトURL	初診の電話等を用いた診療の実施の有無	再診の電話等を用いた診療の実施の有無	対応診療科	担当医師名	対面診療が必要と判断した場合に連携する医療機関名（複数ある場合は複数、住所も併せて記載）
1774	境南クリニック	180-0023	東京都武蔵野市境南町2-7-19 境南スカイビル3,4,5F	0422-33-8411			○	内科	井上　宙哉	
1775	境南耳鼻咽喉科診療所	180-0023	東京都武蔵野市境南町2-7-13	0422-31-1435	http://www.mmkonan.com		○	耳鼻咽喉科	長野　澄雄	
1776	桜堤ファミリークリニック	180-0021	東京都武蔵野市桜堤2-8-55	0422-53-1111	http://www.sakurazutsumi-clinic.com	○	○	内科　外科 消化器内科　内視鏡内科	諸井　隆一	
1777	佐々木産婦人科	180-0022	東京都武蔵野市境4-10-8	0422-54-9311	http://www.sasakisanfujinka.com		○	産婦人科　内科 小児科	佐々木　重胤	
1778	椎名医院	180-0003	東京都武蔵野市吉祥寺南町１-18-6	0422-44-3478			○	精神科　心療内科	椎名　健一	
1779	耳鼻咽喉科青木クリニック	180-0022	東京都武蔵野市境2-14-1 スイングビル402	0422-50-3321	http://www.aoki-clinic.com		○	耳鼻咽喉科	青木　和博	
1780	清水眼科	180-0022	東京都武蔵野市境2-14-1 スイング3F	0422-55-8377	http://www.shimizu.gr.jp/		○	眼科	清水　裕子	
1781	スイング・ビル野崎クリニック	180-0022	東京都武蔵野市境2-14-1 スイング803号	0422-36-3534	http://www.swing-nozakiclinic.com	○	○	内科	野崎　稔	
1782	髙橋医院	180-0006	東京都武蔵野市中町2-13-25	0422-52-3149		○	○	内科　小児科 精神科　外科 婦人科	高橋　猛	武蔵野赤十字病院（東京都武蔵野市境南町1-26-1）杏林大学病院（東京都三鷹市新川6-20-2）東京医科大学病院（東京都新宿区西新宿6-7-1）

電話や情報通信機器を用いて診療を実施する医療機関の一覧（東京都）

	基本情報					事務連絡に基づく対応について				
	施設名	郵便番号	住所（都道府県から記載）	電話番号	ウェブサイトURL	初診の電話等を用いた診療の実施の有無	再診の電話等を用いた診療の実施の有無	対応診療科	担当医師名	対面診療が必要と判断した場合に連携する医療機関名（複数ある場合は複数、住所も併せて記載）
1783	たけうち診療所	180-0006	東京都武蔵野市中町1-10-6<br>三鷹北口共同ビル202	050-3161-2876	http://www.cabinet-takeuchi.org		○	精神科	竹内　光夫	
1784	武居小児科医院	180-0023	東京都武蔵野市境南町2-8-17-2F	0422-32-4152			○　但し慢性疾患のみ	小児科	武居　正郎	
1785	タワーズ内科クリニック	180-0006	東京都武蔵野市中町1-12-10<br>武蔵野タワーズスカイゲートタワー5F・B号	0422-50-1331	http://www.musashino-towers-clm.com/internal.htm		○	内科	西村　徹	
1786	時計台メディカルクリニック	180-0004	東京都武蔵野市吉祥寺本町3-10-14	0422-20-1151	http://www.ichikawa.org		○	内科　外科<br>消化器内科	市川　直哉	
1787	友利院	180-0014	東京都武蔵野市関前2-5-14	0422-51-7365		○	○	内科　小児科<br>外科　胃腸内科	友利　千之	
1788	内科　いしだクリニック	180-0006	東京都武蔵野市中町1-17-2<br>ステラ武蔵野2F	0422-53-1115			○	内科	石田　雄二	
1789	長沼整形外科・内科医院	180-0022	東京都武蔵野市境2-13-6	0422-59-1300	http://www.naganuma-clinic.jp/		○	整形外科　内科	長沼　浩二	
1790	中田内科クリニック	180-0001	東京都武蔵野市吉祥寺北町2-1-14	0422-28-4600			○	内科	中田　哲也	
1791	中町内科医院	180-0006	東京都武蔵野市中町2-31-12<br>メゾンドマーティー1階	0422-55-1203		○	○	内科	久木田　雅弘	

電話や情報通信機器を用いて診療を実施する医療機関の一覧（東京都）

	基本情報					事務連絡に基づく対応について				
	施設名	郵便番号	住所（都道府県から記載）	電話番号	ウェブサイトURL	初診の電話等を用いた診療の実施の有無	再診の電話等を用いた診療の実施の有無	対応診療科	担当医師名	対面診療が必要と判断した場合に連携する医療機関名（複数ある場合は複数、住所も併せて記載）
1792	野の花メンタルクリニック	180-0004	東京都武蔵野市吉祥寺本町1-21-2 グローリオ吉祥寺本町203	0422-28-1587	https://www.br4.fiberbit.net/nonohana0452		○	精神科　心療内科	野田　恭平	
1793	長谷川小児科医院	180-0001	東京都武蔵野市吉祥寺北町2-8-5	0422-22-6804		○	○	小児科	長谷川　正子<br>長谷川　慶	
1794	はせがわ内科	180-0022	東京都武蔵野市境4-4-1	0422-53-0001			○	内科　小児科	長谷川　ひとみ	
1795	春木医院	180-0022	東京都武蔵野市境1-5-4	0422-51-4567			○	内科　小児科 東洋医学　糖尿病内科	春木　武徳	
1796	ひぐち眼科	180-0004	東京都武蔵野市吉祥寺本町1-8-3 ダイヤガイヤビル4F	0422-23-8552	http://www.higuchiganka.com		○	眼科	樋口　裕彦	
1797	樋口クリニック	180-0006	東京都武蔵野市中町1-13-1 駅前田辺ビル2F	0422-56-3588			○	精神科　心療内科　内科　皮膚科	樋口　祥一	
1798	富士見台医院	180-0023	東京都武蔵野市境南町5-11-2	0422-31-8050			○	内科	八島　理	
1799	前澤クリニック	180-0023	東京都武蔵野市境南町3-15-21	0422-30-2861	https://maezawa-clinic.jp/		○	内科　小児科	前澤　良彦<br>前澤　浩美	
1800	松井クリニック	180-0002	東京都武蔵野市吉祥寺東町2-17-27	0422-20-1001	http://www.matsui-clinic.org		○	内科　胃腸内科 アレルギー科 リウマチ科　小児科	松井　秀樹	

電話や情報通信機器を用いて診療を実施する医療機関の一覧（東京都）

	基本情報					事務連絡に基づく対応について				
	施設名	郵便番号	住所（都道府県から記載）	電話番号	ウェブサイトURL	初診の電話等を用いた診療の実施の有無	再診の電話等を用いた診療の実施の有無	対応診療科	担当医師名	対面診療が必要と判断した場合に連携する医療機関名（複数ある場合は複数、住所も併せて記載）
1801	松本医院	180-0002	東京都武蔵野市吉祥寺東町１-23-3	0422-22-5755			○	内科　小児科	三室　知子	
1802	まつもとファミリークリニック	180-0023	東京都武蔵野市境南町2-9-3<br>大矢ビル2F	0422-39-5315	http://www.m-family-clinic.com		○	内科　消化器内科　外科	松本　春美	
1803	美夏クリニック	180-0004	東京都武蔵野市吉祥寺本町1-8-3<br>三松ビル5F	0422-28-2033	http://www.mika-clinic.com		○	皮膚科　形成外科　内科	石井　美夏	
1804	三鷹もろほし耳鼻咽喉科	180-0006	東京都武蔵野市中町一丁目１２番１０号武蔵野タワーズ<br>スカイゲートタワー・タワーズモール内５階・Ｃ号	0422-37-3341	http://www.morohosi-jibika.jp/mitaka/	○	○	耳鼻咽喉科<br>アレルギー科	星川治子	
1805	みどり町眼科	180-0012	東京都武蔵野市緑町1-12-14<br>よつわビル1F	0422-56-8201	http://www.midoricho.sakura.ne.jp		○	眼科	伊藤　寿賀子	
1806	武蔵境皮フ科クリニック	180-0022	東京都武蔵野市境1-3-1<br>ネオック中央ビル2F	0422-56-0121			○	皮膚科	羽田　啓次	
1807	武蔵境眼科医院	180-0023	東京都武蔵野市境南町1-8-1<br>武蔵野STビル2F	0422-34-2117			○	眼科	村松　隆次	
1808	武蔵境病院	180-0022	東京都武蔵野市境1-18-6	0422-51-0301	https://tenseikai.jp/sakai-hosp/	○	○	内科	天野　英介	
1809	武蔵境病院付属あんずクリニック	180-0023	東京都武蔵野市境南町4-1-15	0422-50-9770	https://www.tenseikai.jp/anzu-clinic/	○	○	整形外科　内科<br>皮膚科　小児科<br>リハビリテーション科	天野　秀介<br>十河　まゆみ	

電話や情報通信機器を用いて診療を実施する医療機関の一覧（東京都）

	基本情報					事務連絡に基づく対応について				
	施設名	郵便番号	住所（都道府県から記載）	電話番号	ウェブサイトURL	初診の電話等を用いた診療の実施の有無	再診の電話等を用いた診療の実施の有無	対応診療科	担当医師名	対面診療が必要と判断した場合に連携する医療機関名（複数ある場合は複数、住所も併せて記載）
1810	武蔵野アトラスターズ整形外科　スポーツクリニック	180-0006	東京都武蔵野市中町1-24-15<br>メディパーク中町２F	0422-38-8015	http://www.musashinoasc.com		○	整形外科<br>リハビリテーション科	丸野　秀人	
1811	むさしの共立診療所	180-0013	東京都武蔵野市西久保2-17-11	0422-52-2512	http://www.kyoritsuclinic.org/		○	内科	村田　嘉彦	
1812	むさしの糖尿病・甲状腺クリニック	180-0022	東京都武蔵野市境1-15-10<br>イストワール1F101号	0422-56-1510	https://musashino-dm-thyroid.com/		○	内科　内分泌内科　　糖尿病内科	田口　学	
1813	むさしのメンタルクリニック	180-0022	東京都武蔵野市境2-14-8<br>アドニス・モリヤ1F	0422-53-5155			○	心療内科　精神科	原　仁美	
1814	むらくにクリニック	180-0003	東京都武蔵野市吉祥寺南町1-5-12	0422-76-8036			○	内科（外科、肛門外科、乳腺外科等は対面診療要）	村國　均	
1815	メディカルクリニック武蔵境	180-0023	東京都武蔵野市境南町2-8-19	0422-39-5654	http://wafukai.or.jp		○	脳神経外科　内科	藤井　芳樹	
1816	もとはし内科	180-0001	東京都武蔵野市吉祥寺北町2-19-9	0422-23-6886	http://www.motohashinaika.com		○	内科　消化器内科　　代謝内科　腎臓内科	本橋　茂	
1817	安岡整形外科脳外科クリニック	180-0004	東京都武蔵野市吉祥寺本町2-14-8	0422-20-2010	http://www.yasuoka-clinic.com/sp/	○	○	整形外科　脳外科　　リハビリテーション科	安岡　正蔵	
1818	湯川リウマチ内科クリニック	180-0023	東京都武蔵野市境南町3-14-6-3F	0422-31-1155	http://www.yukawa-clinic.jp/	○	○	内科<br>リウマチ科	湯川宗之助	武蔵野赤十字病院(東京都武蔵野市境南町1-26-1)

電話や情報通信機器を用いて診療を実施する医療機関の一覧（東京都）

	基本情報					事務連絡に基づく対応について				
	施設名	郵便番号	住所（都道府県から記載）	電話番号	ウェブサイトURL	初診の電話等を用いた診療の実施の有無	再診の電話等を用いた診療の実施の有無	対応診療科	担当医師名	対面診療が必要と判断した場合に連携する医療機関名（複数ある場合は複数、住所も併せて記載）
1819	吉方病院	180-0006	東京都武蔵野市中町2-2-4	0422-52-4371	http://valuelab.co.jp/yoshikata/index.html	○	○	整形外科	花岡　央屋	
1820	よしかた内科	180-0011	東京都武蔵野市八幡町3-1-19	0422-50-0351			○	内科	吉方　久雄	
1821	らいおんハート武蔵野脳神経外科内科クリニック	180-0011	東京都武蔵野市八幡町3-1-25	0422-52-6600			○	脳神経外科　内科	仲川　和彦	
1822	良寛こどもファミリークリニック	180-0006	東京都武蔵野市中町2-4-5　1階	0422-27-8621	http://www.ryoukanclinic.com		○	内科　小児科	林　良寛<br>林　知子	
1823	わかばひふ科クリニック	180-0002	東京都武蔵野市吉祥寺東町2-11-2-1F	0422-22-1232	http://www.fc@wakaba-hifuka.com		○	皮膚科	野崎　誠	
1824	渡辺医院	180-0004	東京都武蔵野市吉祥寺本町4-7-9	0422-22-8937		○	○	内科	渡辺　滋	
1825	吉祥寺まいにちクリニック	180-0004	東京都武蔵野市吉祥寺本町1-9-10<br>レインボービル2階	0422-29-3092	https://kichijojiclinic.com/	○	○	内科、泌尿器科、皮膚科	柳澤薫	
1826	１７３総合内科クリニック	181-0013	東京都三鷹市下連雀6-8-50	0422-26-5173	https://173clinic.jp/	○	○	内科<br>泌尿器科	稲見　光春<br>峰村　広<br>中野　由梨	杏林大学病院　東京都三鷹市新川６丁目２０−２　日本赤十字社・武蔵野赤十字病院<br>東京都武蔵野市境南町１丁目２６−１
1827	医療法人社団MYCサウスポイントMYクリニック	181-0013	東京都三鷹市下連雀3-43-23サウスポイント三鷹2･3F	0422-46-7851	https://www.sp-myclinic.com/		○	内科、整形外科	宮坂芳郎　徳永尊彦	

電話や情報通信機器を用いて診療を実施する医療機関の一覧（東京都）

	基本情報					事務連絡に基づく対応について				
	施設名	郵便番号	住所（都道府県から記載）	電話番号	ウェブサイトURL	初診の電話等を用いた診療の実施の有無	再診の電話等を用いた診療の実施の有無	対応診療科	担当医師名	対面診療が必要と判断した場合に連携する医療機関名（複数ある場合は複数、住所も併せて記載）
1828	医療法人社団うちはら内科クリニック	181-0012	東京都三鷹市上連雀7丁目１１番７号石井ビル１階	0422-40-6180	http://www.uchihara-clinic.com	○	○	内科	内原正勝	
1829	医療法人社団　片平内科クリニック	181-00013	東京都三鷹市下連雀3-27-12-4F	0422-79-0271	http://www001.upp.so-net.ne.jp/katahira_medcli/		○	内科	片平　宏	
1830	医）　桜一会　かんの内科	181-0013	東京都三鷹市下連雀3丁目44-17エルヴェ三鷹203	0422-40-5022	info@kanno-naika.com	○	○	内科<br>糖尿病<br>内分泌代謝科	菅野　一男	
1831	医療法人財団慈生会<br>野村病院	181-8503	東京都三鷹市下連雀8-3-6	0422-47-4848	https://www.nomura.or.jp/		○	一般内科<br>消化器内科<br>神経内科<br>糖尿病内科<br>循環器内科<br>呼吸器内科<br>血液内科<br>消化器外科	各担当医	
1832	医療法人社団かえでこどもクリニック	181-0011	東京都三鷹市井口3-6-16アップルかえで通りビル1F	0422-39-3306	https://kaede-kodomo.jp/	○	○	小児科	小口　薫	杏林大学医学部付属病院（東京都三鷹市新川６丁目２０－２）<br>武蔵野赤十字病院（東京都武蔵野市境南町１丁目２６－１）
1833	医療法人社団佳仁会<br>三鷹第一クリニック	181-0013	東京都三鷹市下連雀８－９－２１	0422-46-4141	http://keijin-kai.or.jp/daiichi_hp/		○	内科	宇井　義典	杏林大学医学部付属病院（東京都三鷹市新川6-20-2）<br>武蔵野赤十字病院（東京都武蔵野市境南町1-26-1）
1834	医療法人社団CVIC<br>三鷹心臓クリニック	181-0013	東京都三鷹市下連雀3-26-9<br>サンシロービル4F	03-4500-1670	https://www.mitakaclinic.com/		○	循環器内科<br>内科	寺島　正浩<br>高桑　蓉子	
1835	医療法人社団千実会あきやま子どもクリニック	181-0012	東京都三鷹市上連雀4-3-3川口ビル1階	0422-70-5777	https://www.akiyamakodomo.com/	○	○	小児科	秋山千枝子<br>宗像可枝	
1836	医療法人社団理恵会<br>新川クリニック	181-0004	東京都三鷹市新川5-6-21	0422-43-0123	http://park18.wakwak.com/~shinkawa-clinic/	○	○	内科<br>小児科<br>皮膚科	新後閑　周二<br>新後閑　弘章	

電話や情報通信機器を用いて診療を実施する医療機関の一覧（東京都）

	基本情報					事務連絡に基づく対応について				
	施設名	郵便番号	住所（都道府県から記載）	電話番号	ウェブサイトURL	初診の電話等を用いた診療の実施の有無	再診の電話等を用いた診療の実施の有無	対応診療科	担当医師名	対面診療が必要と判断した場合に連携する医療機関名（複数ある場合は複数、住所も併せて記載）
1837	杏林大学医学部付属病院	181-8611	東京都三鷹市新川6丁目20番2号	0422-47-5511	http://www.kyorin-u.ac.jp/hospital/		○<br>対面診療が必要となる場合あり	内科、循環器科、小児科、皮膚科、精神科、外科、脳神経外科、整形外科、心臓血管外科、形成外科、小児外科、泌尿器	各医師	
1838	公益財団法人　井之頭病院	181-8531	東京都三鷹市上連雀4-14-1	0422-44-5521	http://www.inokashira-hp.or.jp		○<br>病状が安定した患者のみ	精神科	精神科医師全員	
1839	第一医院	181-0013	東京都三鷹市下連雀4-17-30	0422-43-8636			○	内科	谷口　亮一	医療法人慈誠会野村病院（東京都三鷹市下連雀8丁目3番6号）
1840	高松メディカルクリニック	181-0013	東京都三鷹市下連雀4-16-12三鷹第一ビル2F	0422-70-1035	https://www.takamatsu-medical.com/		○	内科	髙松　慶太	野村病院（東京都三鷹市下連雀8-3-6）
1841	東京国際大堀病院	181-0013	東京都三鷹市下連雀4-8-40	0422-47-1000	https://ohori-hosp.jp/		○	泌尿器科	大堀・権藤・鏑木・豊永・竹内・夏山・山下	
1842	のぞみメモリークリニック	181-0013	東京都三鷹市下連雀4-2-8	0422-70-3880	http://www.nozomi-mem.jp/index.html		○	精神科<br>神経内科	木之下　徹	
1843	長谷川病院	181-8586	東京都三鷹市大沢2-20-36	0422-31-8600	hasegawa-hp.or.jp		○	精神科	当日の当番医	
1844	花楯こころ・からだのクリニック	181-0013	東京都三鷹市下連3-34-15<br>アトラス三鷹レジデンス２階	0422-72-0022	http://wwww.hanadate-clinic.com		○	心療内科<br>精神科	加藤　王康	
1845	三鷹駅前クリニック耳鼻咽喉科	181-0013	東京都三鷹市下連雀3丁目36-1　トリコナ3F	0422-26-9810	http://mitaka-jibiinkouka.com	○	○	耳鼻咽喉科	池谷　淳	河北総合病院（東京都杉並区阿佐谷北1-7-3）

電話や情報通信機器を用いて診療を実施する医療機関の一覧（東京都）

	基本情報					事務連絡に基づく対応について				
	施設名	郵便番号	住所（都道府県から記載）	電話番号	ウェブサイトURL	初診の電話等を用いた診療の実施の有無	再診の電話等を用いた診療の実施の有無	対応診療科	担当医師名	対面診療が必要と判断した場合に連携する医療機関名（複数ある場合は複数、住所も併せて記載）
1846	三鷹駅前内科・糖尿病クリニック	180-0013	東京都三鷹市下連雀3-36-1 トリコナ3階	0422-24-8010	https://mitaka-dm.com/	○	○	内科<br>糖尿病内科	田中 祐希<br>国東　拓也<br>玉寄 皓大<br>高岡由梨子	杏林大学医学部付属病院（東京都三鷹市新川6-20-2）<br>武蔵野赤十字病院（東京都武蔵野市境南町1-26-1）<br>河北総合病院（東京都杉並区阿佐ヶ谷北1-7-3）
1847	みたかクリニック	181-0004	東京都三鷹市新川3-21-12	0422-26-5351	http://mitaka.edaclinic.jp/		○	整形外科	沼崎　伸	杏林大学病院(東京都三鷹市)
1848	三鷹さくらクリニック	181-0002	東京都三鷹市牟礼2-11-5	0422-43-6410	http://mitaka-sakura.com/	○	○	内科<br>小児科	古川秋生	
1849	みたか循環器内科	181-0012	三鷹市上連雀2-4-8	0422-45-1210	https://mitakacardio.com/		○	循環器内科<br>内科	宮本貴庸	
1850	三鷹東クリニック	181-0003	東京都三鷹市北野４－８－４０－1階	03-6909-0565	http://houmonshinryou.com/clinic/jukei-03/		○	内科<br>外科<br>老年内科	山城　理仁	
1851	たなべ耳鼻咽喉科	181-0012	東京都三鷹市上連雀9-15-24<br>メゾンドール1階	0422-70-3387	https://www.tanabe-orl.jp		○	耳鼻咽喉科	田鍋　志保	
1852	三鷹駅こころえがおクリック	181-0012	東京都三鷹市上連雀2-1-10 KBビル301	0422-71-3556	https://kokoro-egao.net/		○	精神科<br>心療内科	山田佳幸	
1853	青梅耳鼻咽喉科	198-0024	東京都青梅市新町2-16-2	0428-34-9833	https://omejibika.com/		○	耳鼻いんこう科	坂本　恵	青梅市立総合病院（東京都青梅市東青梅4-16-5）
1854	医療法人社団幸悠会　鈴木慈光病院	198-0052	東京都青梅市長渕五丁目1086番地	0428-22-3126	http://www.kouyukai1968.or.jp		○	精神科	各医師	

電話や情報通信機器を用いて診療を実施する医療機関の一覧（東京都）

	基本情報					事務連絡に基づく対応について				
	施設名	郵便番号	住所（都道府県から記載）	電話番号	ウェブサイトURL	初診の電話等を用いた診療の実施の有無	再診の電話等を用いた診療の実施の有無	対応診療科	担当医師名	対面診療が必要と判断した場合に連携する医療機関名<br>（複数ある場合は複数、住所も併せて記載）
1855	医療法人社団　新町クリニック	198-0024	東京都青梅市新町３－５３－５	0428-31-5377	http://www.shinmachi-cl.com		○	内科	神應　知道	高木病院（東京都青梅市今寺５－１８－１９）
1856	下奥多摩医院	198-0052	東京都青梅市長淵4-376-1	0428-22-2580	https://www.shimookutamaclinic.com/		○	内科	古味　隆子	
1857	友田クリニック	357-0023	東京都青梅市友田町3-136-1	0428-25-1173	http://ntcc.or.jp		○	内科	小沼　裕寿	青梅市立総合病院（東京都青梅市東青梅4-16-5）<br>公立福生病院（東京都福生市加美平1-6-1）
1858	東青梅診療所	198-0042	東京都青梅市東青梅1-7-5	0428-25-8651	http://ome-clinic.com	○	○	内科<br>小児科<br>外科<br>泌尿器科<br>眼科	武信　康弘<br>武信　敦里<br>川口　有紀子<br>小沼　憲祥<br>樋野　忠司<br>清河　宏倫	青梅市立総合病院（東京都青梅市東青梅4-16-5）
1859	医療法人社団大慈会慈秀病院	183-0011	東京都府中市白糸台三丁目16番地の5	042-369-1511	http://www.jishu-hp.jp/		○	内科<br>腎臓内科	稲垣　雄一朗<br>他	多摩総合医療センター（東京都府中市武蔵台２丁目８－２９）
1860	おき医院	183-0011	東京都府中市白糸台5-24-1フジビル102	042-354-1277	https://www.okinopio.jp	○	○	小児科<br>アレルギー科	隠岐直紀<br>川原亜由美	武蔵野赤十字病院(東京都武蔵野市境南町1-26-1)<br>多摩総合医療センター(東京都府中市武蔵台2-8-29)<br>小児総合医療センター(東京都府中市武蔵台2-8-29)
1861	榊原記念病院	183-0003	東京都府中市朝日町3-16-1	042-314-3111	http://www.hp.heart.or.jp/	○	○	循環器内科	各医師にて対応	
1862	崎山小児科	183-0042	東京都府中市武蔵台３－２－２	042-322-7859	http://www.sakiyama.or.jp	○	○	小児科	崎山　弘	
1863	多摩総合医療センター	183-8524	東京都府中市武蔵台2丁目8番29号	042-323-5111	https://www.fuchu-hp.fuchu.tokyo.jp/		○	整形外科<br>リウマチ膠原病科<br>外科・乳腺外科<br>消化器内科<br>脳神経内科<br>血液内科<br>呼吸器内科	整形外科：伊賀　徹、その他診療科各担当<br>リウマチ膠原病科：島田　浩太　その他診療科各担当<br>外科・乳腺外科：森田　泰弘　その他診療科各担当<br>消化器内科：並木　伸　その他診療科各担当<br>脳神経内科：川田　明広　その他診療科各担当<br>血液内科：香西　康司　その他診療科各担当<br>呼吸器内科：高森　幹雄　そ[illegible]	

電話や情報通信機器を用いて診療を実施する医療機関の一覧（東京都）

	基本情報					事務連絡に基づく対応について				
	施設名	郵便番号	住所（都道府県から記載）	電話番号	ウェブサイトURL	初診の電話等を用いた診療の実施の有無	再診の電話等を用いた診療の実施の有無	対応診療科	担当医師名	対面診療が必要と判断した場合に連携する医療機関名（複数ある場合は複数、住所も併せて記載）
1864	東京都立小児総合医療センター	183-8561	東京都府中市武蔵台2-8-29	042-300-5111	https://www.byouin.metro.tokyo.lg.jp/shouni/		○	予約センター（電話042-312-8200）へ平日9:00～16:00の間にお問い合わせください。	外来担当医	
1865	東府中病院	183-0005	東京都府中市若松町2-7-20	042-364-0151	https://www.higashifuchu-hp.jp/	○	○	内科<br>小児科<br>産婦人科	西弘示<br>川端利信<br>秋林雅也<br>繁友憲郎<br>里見操緒<br>細野真沙子<br>十藏寺晶子	
1866	府中駅前クリニック	183-0023	東京都府中市宮町1-100 ル・シーニュ4階	042-319-8383	https://fuchu.towakai.com/		○	内科	金澤　寛之	
1867	府中恵仁会病院	183-8507	東京都府中市住吉町5-21-1	042-365-1211	https://www.fuchu-keijinkai.or.jp/		○	内科<br>外科<br>脳神経外科	上林　拓男<br>小林　泉<br>伊奈　秀高<br>畑田　英彦<br>倉田　勇<br>久保　佳史<br>福地　孝明<br>名倉　正利	
1868	あさの循環器内科クリニック	183-0056	東京都府中市寿町１－４－２１<br>第３福井ビル２F	042-366-5555	http://www.asanoclinic.jp/		○	内科<br>循環器内科	浅野竜太	
1869	宮西クリニック	183-0022	東京都府中市宮西町2－4－3<br>アイスバーグビル2F	042-362-5833		○	○	内科<br>小児科	大谷哲士	
1870	寿町クリニック	183-0056	東京都府中市寿町1-3-10<br>藤和府中コープ101	042-354-3111	http://kc-clinic.jp/	○	○	脳神経外科	賣野智之	都立多摩総合医療センター<br>（東京都府中市武蔵野台2-9-2）
1871	愛和診療所	183-0051	東京都府中市栄町3-12-1<br>ﾒﾃﾞｨｶﾙﾋﾞﾙ・ｸﾞﾚｲｽ1Ｆ	042-314-1551	http://www.aiwa-cl.net		○	内科　小児科<br>精神科　漢方内科　疼痛緩和内科	柴田　光太郎	
1872	医療法人社団均禮会<br>府中の森土屋産婦人科	183-0055	東京都府中市府中町3-10-9	042-351-3748	http://wwwtsuchiyaclinic.com		○	産婦人科	土屋　清志	

電話や情報通信機器を用いて診療を実施する医療機関の一覧（東京都）

	基本情報					事務連絡に基づく対応について				
	施設名	郵便番号	住所（都道府県から記載）	電話番号	ウェブサイトURL	初診の電話等を用いた診療の実施の有無	再診の電話等を用いた診療の実施の有無	対応診療科	担当医師名	対面診療が必要と判断した場合に連携する医療機関名（複数ある場合は複数、住所も併せて記載）
1873	昭島リウマチ膠原病内科	196-0024	東京都昭島市宮沢町４９５－３０	042-546-0011	https://akishima-rheumatologist.jp/		○	内科<br>リウマチ科<br>アレルギー科	吉岡拓也	
1874	うしお病院	196-0021	東京都昭島市武蔵野2-7-12	042-541-5423	http://www.usio-hp.com/	○	○	内科<br>外科、胃腸科<br>整形外科	加藤　真帆人<br>杉本　大<br>森川　五竜<br>万本　潤<br>渡部　泰幸	
1875	麻生こどもクリニック	182-0026	東京都調布市小島町1－5－6	042-480-7810	https://www.aso-kodomo.jp/		○	小児科	麻生　泰二	
1876	医療法人社団青山会<br>青木病院	182-0035	東京都調布市上石原３丁目３３番地の１７	042-483-1355	http://www.aoki-hospital.jp/		○	内科<br>精神科	各医師	
1877	医療法人社団あすなろの会　ぬきいこどもクリニック	182-0022	東京都調布市国領町4-8-1　プラザパレット2階	042-440-0215	http://www.anssam.com/yoyaku/nkcymb/rsv_top.asp	○	○	小児科　アレルギー科	貫井清孝	
1878	医療法人社団CVIC<br>仙川心臓クリニック	182-0002	東京都調布市仙川町2-12-6<br>アヴェニール仙川1階	03-4500-1310	https://www.cviclinic-sengawa.com/		○	循環器内科<br>内科	寺島　正浩<br>濵田　佳江	
1879	医療法人社団スリープクリニック<br>スリープクリニック調布	182-0026	東京都調布市小島町２－５３－１	042-490-0700	https://www.sleepmedicine-tokyo.com/		○	精神科<br>心療内科	当日の担当医	
1880	医療法人社団東山会<br>調布東山病院	182-0026	東京都調布市小島町2-32-17	042-481-5511	https://www.touzan.or.jp/		○	各科	担当医	
1881	医療法人社団大和会<br>多摩川病院	182-0022	東京都調布市国領町5-31-1	042-483-4114	https://tamagawahp.jp/	○	○	内科<br>循環器内科<br>整形外科	後藤　紀史<br>天野　茂夫<br>齋藤　正之<br>駒形　正志<br>太田　晃一<br>前園　知宏<br>高亀　則博<br>清水　孝夫	

電話や情報通信機器を用いて診療を実施する医療機関の一覧（東京都）

	基本情報					事務連絡に基づく対応について				
	施設名	郵便番号	住所（都道府県から記載）	電話番号	ウェブサイトURL	初診の電話等を用いた診療の実施の有無	再診の電話等を用いた診療の実施の有無	対応診療科	担当医師名	対面診療が必要と判断した場合に連携する医療機関名（複数ある場合は複数、住所も併せて記載）
1882	医療法人社団ラサンテ くにさわクリニック	182-0033	東京都調布市富士見町2-14-19三ツ木富士見町マンション1階	080-5529-8117	https://www.e-kuni.jp	○	○	内科、呼吸器科	國澤晃、金子和代、川述剛士、濱中和嘉子	杏林大学病院（東京都三鷹市新川6-20-2）
1883	永研会クリニック	182-0002	東京都調布市仙川町1-11-8　2F	03-5315-1181	http://eikencl.jp/	○	○	内科<br>整形外科	羽田　谷川<br>菊地　西迫	
1884	くろかわクリニック	182-0022	東京都調布市国領町5-70-2サン・ルーシー２階	042-444-4880	http://kurokawa.byoinnavi.jp/		○	内科	黒川富貴子	
1885	国領駅前耳鼻科	182-0022	東京都調布市国領町3-10-24　レジェンド国領1F	042-440-1187	http://kokuryo.sho-jin.com/	○	○	耳鼻科	本田　英幸	
1886	国領内科・消化器内科クリニック	182-0022	東京都調布市国領町3-3-20 よろずやビル201号室	042-426-9509	https://kokuryou-naika.com/		○	内科	高田　康裕	
1887	国領医院	182-0022	東京都調布市国領町7-46-5	042-482-2600			○	内科<br>血液内科	薄井　紀子	東京慈恵会医科大学附属第三病院（東京都狛江）
1888	国領めいようクリニック	182-0022	東京都調布市国領町8-6-8	042-440-1515	http://www.meiyou-clinic.com/		○	内科<br>整形外科	富永　伸徳<br>菅澤　源<br>堀江　俊裕	
1889	せき循環器内科クリニック	182-0026	東京都調布市小島町1-14-3ヒューリック調布2F	042-487-1505	http://sekiclinic.net		○	循環器内科<br>内科	関　敦	
1890	仙川整形外科	182-0002	東京都調布市仙川町３−２−４ウィステリア仙川1階Ａ	03-3305-0088	https://sengawa-ortho.jp		○	整形外科	日下部　浩	

電話や情報通信機器を用いて診療を実施する医療機関の一覧（東京都）

	基本情報					事務連絡に基づく対応について				
	施設名	郵便番号	住所（都道府県から記載）	電話番号	ウェブサイトURL	初診の電話等を用いた診療の実施の有無	再診の電話等を用いた診療の実施の有無	対応診療科	担当医師名	対面診療が必要と判断した場合に連携する医療機関名（複数ある場合は複数、住所も併せて記載）
1891	仙川脳神経外科クリニック	182-0002	東京都調布市仙川町3-9-15モンヴィラージュ仙川1階	03-5969-8061	https://sncl.jp/		○	脳神経外科	山口竜一	
1892	調布駅前クリニック耳鼻咽喉科	182-0024	東京都調布市布田4-17-10 セントラルレジデンス調布3F	042-441-5161	http://chofu.sho-jin.com/	○	○	耳鼻科	髙原　恵理子	
1893	桐光会　調布病院	182-0034	東京都調布市下石原３－４５－１	042-484-2626	http://www.tokokai.or.jp/		○	内科外科整形外科産婦人科脳神経外科	全医師	
1894	飛田給内科クリニック	182-0036	東京都調布市飛田給1－28－2　1階	042-497-6380			○	内科	井上　理慧	
1895	濱中めいようクリニック	182-0022	東京都調布市国領町7-33-1	042-452-8970	https://www.hamanaka-meiyou-cl.com/		○	内科	濱中　久尚<br>佐藤　義典	
1896	ふかざわクリニック	182-0025	東京都調布市多摩川5-15-5ﾌﾟﾚｾﾝﾄM 2階	042-444-3170	https://fukazawa.clinic/		○	内科<br>小児科	深澤　一郎	
1897	ふしや内科・消化器内科クリニック	182-0021	東京都調布市調布ヶ丘3-19-12　桑田ビル2階	042-485-0248	http://www.fushiya-clinic.jp	○	○	内科　消化器内科内視鏡内科　胃腸内科　肝臓・胆のう・膵臓内科	伏谷　直	
1898	プライムクリニック	182-0004	東京都調布市入間町2-28-36 ウェリスオリーブ成城学園前１階	050-1746-1165	https://prime-clinic.jp/	○	○	総合診療、内科，循環器内科、ペイン緩和、小児科	佐藤大介、露嵜仁志、田嶋美裕	
1899	緑ヶ丘井上内科診療所	182-0001	東京都調布市緑ヶ丘2-2-1	03-3309-2202	http://www.midori-inouenaika.com	○	○	内科	井上明彦	

電話や情報通信機器を用いて診療を実施する医療機関の一覧（東京都）

	基本情報					事務連絡に基づく対応について				
	施設名	郵便番号	住所（都道府県から記載）	電話番号	ウェブサイトURL	初診の電話等を用いた診療の実施の有無	再診の電話等を用いた診療の実施の有無	対応診療科	担当医師名	対面診療が必要と判断した場合に連携する医療機関名（複数ある場合は複数、住所も併せて記載）
1900	村上こどもクリニック	182-0002	東京都調布市仙川町3-7-15	03-3300-4633	https://www.murakamikodomo-clinic.com/	○	○	小児科<br>内科<br>アレルギー科	村上　由加里	杏林大学病院（東京都三鷹市新川6-20-2）<br>慈恵医大第3病院（東京都狛江市和泉4-11-1）
1901	山田病院	182-0005	東京都調布市東つつじヶ丘2-36-1	03-3308-8281	http://www.myclinic.ne.jp/hpyamada/pc/		○	精神科	藤枝　誠<br>酒井　啓子<br>田吉　伸哉<br>松島　英介<br>山口　登<br>荒木　剛<br>白石　弘巳<br>岩瀬　利郎	
1902	予防接種のこども診療所	182-0023	東京都調布市染地２－４３－７６	042-455-1889	http://11sakura.jimdofree.com	○	○	小児科<br>内科	藤本　巌	
1903	医療法人社団梟杜会<br>西田医院	182-0014	東京都調布市柴崎1-64-13	042-483-1350	http://www.nishida-scl.com/	○	○	内科<br>小児科	西田 伸一	
1904	おぎもと内科クリニック	182-0022	東京都調布市国領町2-13-8	042-489-6658	http://www.ogimoto-naika.com/	○	○	内科	荻本剛一	
1905	しおり小児科	182-0024	東京都調布市布田2-44-1	042-440-7213	http://shioriped.com		○	小児科	根本しおり	
1906	伊藤医院	182-0006	東京都調布市西つつじヶ丘4－19－11	042-483-0127			○	内科<br>小児科	原田日女	
1907	鵜戸西クリニック	182-0017	東京都調布市深大寺元町4−31−2	042-482-8658	https://udonishi-clinic.com		○	外科内科泌尿器科	宇野能子<br>宇野正志	
1908	東つつじケ丘さくらクリニック	182-0003	東京都調布市東つつじヶ丘3−53−11	03-3300-3366	https://hopesakura.com/	○	○	内科<br>小児科	神谷　増三	杏林大学医学部附属病院<br>（〒181-8611 東京都三鷹市新川6丁目20−2）

電話や情報通信機器を用いて診療を実施する医療機関の一覧（東京都）

	基本情報					事務連絡に基づく対応について				
	施設名	郵便番号	住所（都道府県から記載）	電話番号	ウェブサイトURL	初診の電話等を用いた診療の実施の有無	再診の電話等を用いた診療の実施の有無	対応診療科	担当医師名	対面診療が必要と判断した場合に連携する医療機関名（複数ある場合は複数、住所も併せて記載）
1909	こころのクリニック調布	182-0024	東京都調布市布田2-51-6-402	042-444-8527			○	精神科<br>心療内科	伊藤郁子<br>伊藤真人	
1910	医)友歩会<br>いしいこどもクリニック	182-0021	東京都調布市調布ケ丘3-19-12桑田ビル1階B	042-483-0101	https://ishii-kodomo.com/		○	小児科	石井　敏夫	
1911	一般財団法人ひふみ会<br>まちだ丘の上病院	195-0064	東京都町田市小野路町11-1	042-735-3731	https://machida-hospital.com	○	○	内科	米倉　修司<br>小森　將史	町田市民病院<br>多摩南部地域病院<br>日本医科大学多摩永山病院
1912	稲垣耳鼻咽喉科医院	194-0013	東京都町田市原町田6-22-15	042-722-3115	http://inagaki-ent.com	○	○	耳鼻咽喉科	稲垣　康治	
1913	入倉クリニック	195-0057	東京都町田市真光寺2-37-11鶴川台メディカルヴィレッジ脳神経外科棟	042-734-3339	http://www.myclinic.ne.jp/irikura/	○	○	脳神経外科、麻酔科、整形外科	入倉克己、入倉弘子	町田市民病院（町田市旭町2-15-41）、多摩南部地域病院（多摩市中沢2-1-2）、新百合ヶ丘総合病院（川崎市麻生区古沢都古255
1914	医療法人社団おおぞら会<br>つばさクリニック	194-0035	東京都町田市忠生3-25-11　忠生ビル302号室	042-794-6811	https://www.clinic-tsubasa.com/	○	○	精神科<br>内科<br>心療内科	鈴木　智広	
1915	医療法人社団　快晴会<br>やまざき整形外科・外科	195-0074	東京都町田市山崎町2055-2　グランハート町田B-105	042-794-4960	http://kaiseikai-net.or.jp/group/yamazaki-seikei		○	整形外科<br>皮膚科	濱田一壽<br>庭山麻里絵	
1916	医療法人社団恭啓会小野寺クリニック	194-0032	東京都町田市本町田4394-9本町田メディカルプラザ内	042-732-5605	http://onodera.webcrow.jp/yi_liao_fa_ren_she_tuan_gong_qi_hui_xiao_ye_sikurinikku/yi_liao_fa_ren_she_tuangong_qi_huixiao_ye_sikurinikku.html		○	内科<br>泌尿器科	小野寺　恭忠	町田市民病院(町田市旭町2-15-41)
1917	医療法人社団　幸隆会<br>多摩丘陵病院	194-0297	東京都町田市下小山田町1491	042-797-1511	http://www.tamakyuryo.or.jp/hospital/		○	内科<br>外科<br>整形外科<br>眼科<br>脳神経外科<br>泌尿器科<br>皮膚科<br>婦人科	各医師	

電話や情報通信機器を用いて診療を実施する医療機関の一覧（東京都）

	基本情報					事務連絡に基づく対応について				
	施設名	郵便番号	住所（都道府県から記載）	電話番号	ウェブサイトURL	初診の電話等を用いた診療の実施の有無	再診の電話等を用いた診療の実施の有無	対応診療科	担当医師名	対面診療が必要と判断した場合に連携する医療機関名（複数ある場合は複数、住所も併せて記載）
1918	医療法人社団慶泉会町田慶泉病院	194-0005	東京都町田市南町田2-1-47	042-795-1668	https://www.machida-keisen.com/		○	内科系<br>外科系		
1919	医療法人社団仁悠会<br>しながわ内科・循環器クリニック	195-0074	東京都町田市山崎町333-1	042-791-1545	https://www.shinagawaclinic.com/	○	○	内科	品川　弥人<br>佐藤　弥生<br>品川　直介<br>加藤　彩美	
1920	医療法人社団藤栄会<br>ただお整形外科・内科	194-0035	東京都町田市忠生2-28-5	042-793-0201	http://tadao-clinic.com/	○	○	内科<br>整形外科	葛目　正央<br>田畑　忍	
1921	医療法人社団成瀬台耳鼻咽喉科	194-0043	東京都町田市成瀬台3-8-16	042-729-3341	http://narujibi.byoinnavi.jp/pc/	○	○	耳鼻咽喉科	井藤博之	
1922	医療法人社団<br>はやしクリニック	194-0035	東京都町田市忠生2-28-7	042-793-3055	http://hayashi-clinic.jp	○	○	小児科<br>内科	林　泉彦<br>森　俊夫	
1923	氏川眼科医院	194-0013	東京都町田市原町田６－１－１１ルミネ町田７階	042-720-0530	http://www.ujikawa-ganka.com/	○	○	眼科	氏川　真理	
1924	おだぎりクリニック	194-0021	東京都町田市中町1-3-1	042-721-1102			○	循環器内科	小田切　重遠	大和成和病院（神奈川県大和市南林間9-8-2）<br>新百合ヶ丘総合病院（神奈川県川崎市麻生区古沢都古２５５）
1925	金森かとう眼科	194-0015	東京都町田市金森東4-42-17よこたビル1階	042-788-4566	https://kanamori-katoeye.com/	○	○	眼科	加藤　雄一	
1926	佐藤威文前立腺クリニック	194-0013	東京都町田市原町田4-14-14 Lifixﾋﾞﾙ 2F	042-860-6307			○	泌尿器科	佐藤　威文	

電話や情報通信機器を用いて診療を実施する医療機関の一覧（東京都）

	基本情報					事務連絡に基づく対応について				
	施設名	郵便番号	住所（都道府県から記載）	電話番号	ウェブサイトURL	初診の電話等を用いた診療の実施の有無	再診の電話等を用いた診療の実施の有無	対応診療科	担当医師名	対面診療が必要と判断した場合に連携する医療機関名（複数ある場合は複数、住所も併せて記載）
1927	湘南内科皮フ科クリニック町田院	〒194-0013	東京都町田市原町田6-3-20 TK町田ビル5階	050-5576-1321	https://sbc-medicalcare.com/	○	○	内科　皮膚科<br>泌尿器科（男性性感染症）	石黒　由高	町田市民病院（東京都町田市旭町2-15-41）
1928	整形外科・リハビリテーション科　かもめClinic	194-0013	東京都町田市原町田6-17-6MHCLビル1F	042-850-8598			○	整形外科	工藤　敏治	
1929	園田クリニック	194-0045	東京都町田市南成瀬１－８－２１　成瀬メディカルビル３階	042-739-7322			○	内科	園田　和彦	
1930	たけいち内科クリニック	194-0003	東京都町田市小川1-14-1	042-850-6555	https://takeichi-naika.jp		○	内科	武市　康敬	
1931	たなかだて内科・呼吸器内科クリニック	194-8589	東京都町田市鶴間3-3-1　グランベリーパーク　ｽﾃｰｼｮﾝｺｰﾄ2F	042-850-5027	http://www.tanakadate-clinic.com/		○	内科	田中舘　基親	南町田病院、虎の門病院、虎の門病院分院
1932	つくしの駅前内視鏡クリニック	194-0001	東京都町田市つくし野2-26-17-1F	042-850-7140	http://tsukushino.or.jp	○	○	消化器内科<br>内視鏡内科	松本　育宏	
1933	つくし野耳鼻咽喉科	194-0003	東京都町田市小川1-2-32	042-796-8733	http://www.tsukushino-jibika.jp		○	耳鼻咽喉科<br>小児耳鼻咽喉科<br>麻酔科	泰地　秀信	
1934	鶴川さくら病院	195-0064	東京都町田市小野路町1632番地	042-735-2621	tsurukawasakura.or.jp/		○	精神科<br>内科	富正哉<br>有山襄<br>橋本智矢	
1935	鶴川記念病院	195-0054	東京都町田市三輪町1059番地1	044-987-1311	https://www.tsurukawakinen.or.jp	○	○	内科<br>小児科	舩津　到<br>小野　朋洋	

電話や情報通信機器を用いて診療を実施する医療機関の一覧（東京都）

	基本情報					事務連絡に基づく対応について				
	施設名	郵便番号	住所（都道府県から記載）	電話番号	ウェブサイトURL	初診の電話等を用いた診療の実施の有無	再診の電話等を用いた診療の実施の有無	対応診療科	担当医師名	対面診療が必要と判断した場合に連携する医療機関名（複数ある場合は複数、住所も併せて記載）
1936	鶴川レディースクリニック	195-0053	東京都町田市能ヶ谷3-1-5	042-737-7271	http://www9.plala.or.jp/tsurukawa-lds/	○	○	内科<br>産婦人科	中林　豊	
1937	豊川小児科内科医院	194-0001	東京都町田市つくし野2-18-18	042-795-4465	https://www.machida.tokyo.med.or.jp/?iryokikan=mina40	○	○	小児科<br>内科	豊川　達記	
1938	なるせクリニック	194-0046	東京都町田市西成瀬1-57-17	042-721-6686			○	内科	佐木　伸一郎	
1939	はやしクリニック分院	194-0032	東京都町田市本町田2943-1<br>サンライズヒル2F	042-720-8868	http://hayashi-clinic.jp	○	○	小児科	中山　有子<br>山下　理恵子	
1940	久田内科・呼吸器内科クリニック	194-0035	東京都町田市忠生3-20-2	042-793-4114	http://www.daiichiho.byoinnavi.jp/pc/	○	○	一般内科<br>呼吸器内科	久田淳一郎	
1941	雅クリニック	194-0013	東京都町田市原町田6-17-4芳村ビル1階	042-850-8790	http://www.masa-clinic.com		○	精神科<br>心療内科	鈴木雅博	
1942	増子クリニック	195-0053	東京都町田市能ケ谷7-11-5	042-735-1499	https://masuko-clinic.jimdo.com/	○	○	整形外科<br>皮膚科<br>内科	増子達也<br>増子真理<br>中村文昭	
1943	町田駅前内科クリニック	194-0022	東京都町田市森野１−３９−１６ 渋谷一樹ビル３F	042-722-7373	https://machida-ekimaenaika.com/	○	○	内科	伊原 玄英	
1944	町田市民病院	194-0023	東京都町田市旭町2-15-41	042-722-2230	http://machida-city-hospital-tokyo.jp/		○	内科<br>外科<br>産婦人科<br>脳神経外科<br>脳神経内科<br>整形外科<br>精神科<br>皮膚科	各医師	

電話や情報通信機器を用いて診療を実施する医療機関の一覧（東京都）

	基本情報					事務連絡に基づく対応について				
	施設名	郵便番号	住所（都道府県から記載）	電話番号	ウェブサイトURL	初診の電話等を用いた診療の実施の有無	再診の電話等を用いた診療の実施の有無	対応診療科	担当医師名	対面診療が必要と判断した場合に連携する医療機関名（複数ある場合は複数、住所も併せて記載）
1945	南町田こどもクリニック	194-0004	東京都町田市鶴間1-19-35 南町田クリニックビルI-2階　201号室	042-796-2200	http://mm-child-clinic.jp	○	○	小児科	佐藤　明弘	
1946	みるるクリニック原町田	194-0013	東京都町田市原町田1-15-18	042-710-5300	http://www.miruru-clinic.com/		○	内科<br>小児科<br>眼科	志野原　睦<br>志野原　知子	
1947	もとはしクリニック	194-0021	東京都町田市中町2-14-10	042-727-9930			○	内科	本橋　久彦	
1948	薬師台おはなぽっぽクリニック	195-0073	東京都町田市薬師台1-25-12　薬師台メディカルテラス内	042-737-7373	yakushidai-mt.com/yopc/index.html	○	○	内科<br>整形外科<br>精神科<br>循環器内科	野口　泰芳	・渕野辺総合病院<br>〒252-0206　神奈川県相模原市中央区淵野辺3丁目2-8<br>・町田市民病院<br>〒194-0023　東京都町田市旭町2-15-41
1949	山口小児クリニック	195-0063	東京都町田市野津田町１３６４	080-8747-2456	http://yama-ped.jp/	○	○	小児科<br>内科	今井文英<br>山口乙丸	
1950	若山クリニック	195-0072	東京都町田市金井2-3-18	042-736-7720	http://wakayama-cl.byoinnavi.jp/pc/index.html	○	○	内科、脳神経内科、小児科、皮膚科	若山　幸示<br>北　さくら	
1951	あとう皮膚科・形成外科クリニック	194-0037	東京都町田市木曽西2-17-26<br>木曽西医療ビル2階	042-791-1055	http://www.ato-clinic.com		○	皮膚科、形成外科	阿藤　晃一	聖マリアンナ医科大学病院<br>（神奈川県川崎市宮前区菅生2-16-1）
1952	和クリニック	195-0062	東京都 町田市 大蔵町 5002-2<br>鶴川メディカルモール1B-2	042-737-5055	http://kazu-clinic.jp/	○	○	精神科・心療内科	前田佳宏	
1953	小出クリニック	194-0036	東京都町田市木曽東1-15-40	042-721-2918	http://www.tohoyk.jp/hp/koide-cl/		○	内科<br>皮膚科<br>小児科	小出　勝彦	町田病院（東京都町田市木曽東4-21-43）

電話や情報通信機器を用いて診療を実施する医療機関の一覧（東京都）

	基本情報					事務連絡に基づく対応について				
	施設名	郵便番号	住所（都道府県から記載）	電話番号	ウェブサイトURL	初診の電話等を用いた診療の実施の有無	再診の電話等を用いた診療の実施の有無	対応診療科	担当医師名	対面診療が必要と判断した場合に連携する医療機関名（複数ある場合は複数、住所も併せて記載）
1954	アクウェルモール さくら眼科	184-0004	東京都小金井市本町6-14-28アクウェルモール2階	042-383-5558	https://aquwellmall.sakuraganka.jp/		○	眼科	小林　文貴	
1955	いせだ脳神経外科　神経内科	184-0002	東京都小金井市梶野町5-11-5　パピスプラザ1階	042-316-1518	http://www.isedans.jp		○	脳神経外科	伊勢田　努力	
1956	医療法人社団佳翔会 武蔵小金井さくら眼科	184-0004	東京都小金井市本町1-18-3-B101	042-383-0077	https://sakuraganka.jp	○	○	眼科	安田　佳守臣<br>南雲　はるか	
1957	医療法人社団大日会　小金井太陽病院	184-0004	東京都小金井市本町1-9-17	042-383-5511	http://www.koganei-taiyo.com/	○<br>電話初診は現在準備中、対象は内科のみ	○	内科<br>呼吸器内科<br>循環器内科<br>消化器内科<br>外科・消化器外科<br>整形外科	中村譲（内科）<br>開陽子（呼吸器内科）　田村俊一（循環器内科）　三浦美貴（消化器内科）<br>野内亘（外科・消化器外科）	
1958	医療法人社団つるかめ会 小金井つるかめクリニック	184-0004	東京都小金井市本町6-14-28<br>プラウドタワー武蔵小金井3階	042-386-3757	https://koganei.tsurukamekai.jp/index.html	○	○	内科	石橋　史明<br>川上　智寛<br>小林　小の実	杏林大学医学部附属病院（東京都三鷹市新川6-20-2）<br>武蔵野赤十字病院（東京都武蔵野市境南町1-26-1）<br>多摩総合医療センター（東京都府中市武蔵台2-8-29）<br>公立昭和病院（東京都小平市花小金井8-1-1）
1959	医療法人社団汎和会　さいとう医院	184-0011	東京都小金井市東町4-21-10	042-380-5510	http://www.saito-clinic.jp	○	○	内科循環器科消化器科	斎藤寛和	武蔵野赤十字病院（東京都武蔵野市境南町1-26-1）
1960	菊地脳神経外科・整形外科	184-0011	東京都小金井市東町3-12-11	0422-31-1220	https://neuro-kikuchi.or.jp/		○	脳神経外科<br>内科<br>整形外科	菊地　隆文<br>菊地　邦夫<br>菊地　令子<br>赤崎　安晴	
1961	在宅24クリニック小金井	184-0013	東京都小金井市前原町3-16-14玉野屋コーポラス102号	042-316-6746	https://www.zaitaku24.clinic/	○	○	内科<br>精神科	福岡　俊宗<br>河野　敏明	
1962	三枝耳鼻咽喉科・小児科医院	184-0004	東京都小金井市本町5-19-32三枝ビル2階	042-381-8221			○	耳鼻咽喉科<br>小児科	三枝歌子	

電話や情報通信機器を用いて診療を実施する医療機関の一覧（東京都）

	基本情報					事務連絡に基づく対応について				
	施設名	郵便番号	住所（都道府県から記載）	電話番号	ウェブサイトURL	初診の電話等を用いた診療の実施の有無	再診の電話等を用いた診療の実施の有無	対応診療科	担当医師名	対面診療が必要と判断した場合に連携する医療機関名（複数ある場合は複数、住所も併せて記載）
1963	新こがねい呼吸器内科	184-0011	東京都小金井市東町４－８－１３　登川ビル　１階	042-389-9080	https://shinkoganei-cl.com/		○	呼吸器内科<br>アレルギー科<br>内科	原崎　一浩	武蔵野赤十字病院（東京都武蔵野市境南町1-26-1）
1964	竹田内科クリニック	184-0014	東京都小金井市貫井南町5-20-13	042-381-6627	http://www.takedamc.com	○	○	内科<br>循環器内科<br>腎臓内科<br>糖尿病内科	竹田　和義<br>竹田　渓輔	武蔵野赤十字病院（東京都武蔵野市境南町1-26-1）<br>多摩総合医療センター（東京都府中市武蔵台2-8-29）
1965	東小金井クリニック	184-0002	東京都小金井市梶野町2-1-2	0422-56-8630		○	○	内科・整形外科・アレルギー科	豊田　雅樹	武蔵野日赤病院（東京都武蔵野市境南町１丁目２６－１）多摩総合医療センター（東京都府中市武蔵台２丁目８－２９）
1966	ひらた循環器クリニック	184-0004	東京都小金井市本町5-40-3	042-401-6157			○	内科<br>循環器内科	平田 二紀代	
1967	武蔵小金井クリニック	184-0004	東京都小金井市本町5-19-33	042-384-0080	http://www.kanwakai.jp	○	○	内科	金光　寛承	
1968	もろほしクリニック	184-0003	東京都小金井市 緑町 1-6-53	042-385-3341	http://www.morohosi-jibika.jp/	○	○	耳鼻咽喉科<br>アレルギー科<br>難聴外来	諸星　咲子<br>白井　杏湖	
1969	隆誠会　宮本内科医院	184-0012	東京都小金井市中町3-7-4	042-381-2219	http://www.miyamoto-mc.com/	○	○	内科	宮本　諭	
1970	医療法人社団　玉翠会　喜平橋耳鼻咽喉科	187-0044	東京都小平市喜平町1-7-26	042-332-3387	https://ssl.kihei.jp	○	○	耳鼻咽喉科<br>アレルギー科<br>気管食道科<br>小児科内科	村川哲也	公立昭和病院
1971	医療法人社団若生会　遠藤こどもクリニック	187-0001	東京都小平市大沼町1-21-8	042-343-0066	http://www.endo-kodomo-clinic.com	○	○	小児科	遠藤　隆	1.公立昭和病院（東京都小平市花小金井8-1-1）2.小児総合医療センター（府中市武蔵台2-8-29）　3.多摩北部医療センター（東村山市青葉町1-7-1）

電話や情報通信機器を用いて診療を実施する医療機関の一覧（東京都）

	基本情報					事務連絡に基づく対応について				
	施設名	郵便番号	住所（都道府県から記載）	電話番号	ウェブサイトURL	初診の電話等を用いた診療の実施の有無	再診の電話等を用いた診療の実施の有無	対応診療科	担当医師名	対面診療が必要と判断した場合に連携する医療機関名（複数ある場合は複数、住所も併せて記載）
1972	医療法人社団修恵会<br>新小平クリニック	187-0032	東京都小平市小川町2-1975-8	042-312-1185	http://www.shinkodaira-clinic.com	○	○	内科　心療内科<br>精神科　ｱﾚﾙｷﾞｰ科　漢方内科<br>皮膚科　糖尿病内科	富田　修平	公立昭和病院（東京都小平市花小金井8丁目1-1）、多摩総合医療センター（東京都府中市武蔵台2丁目8-29）、多摩北部遺留センター（東京都東村山市青葉町1丁目7-1）、東大和病院（東京都東村山市南街1-13-12）
1973	小川クリニック	187-0035	東京都小平市小川西町3-11-1	042-341-0653	http://www.ogawa-cl.net		○	内科<br>小児科	小川　哲史	
1974	かく内科整形外科	187-0032	東京都小平市小川町2-1858	042-345-7000	https://kakunaika.byoinnavi.jp/		○	内科<br>整形外科	澤田晋	公立昭和病院（東京都小平市花小金井八丁目1-1）
1975	けぶかわ医院	187-0045	東京都小平市学園西町二丁目１５番１号<br>小平サンパークビル１階	042-348-1300	http://kebukawa.jp/		○	内科<br>循環器内科	星野虎生	
1976	公立昭和病院	187-8510	東京都小平市花小金井八丁目1番1号	042-461-0052	http://www.kouritu-showa.jp/		○	呼吸器内科<br>循環器内科<br>消化器内科<br>血液内科<br>糖尿病・内分泌・代謝内科<br>腎臓内科<br>脳神経内科<br>心療内科<br>外科	再診担当医師	
1977	国立精神・神経<br>医療研究センター	187-8551	東京都小平市小川東町4-1-1	042-341-2711	https://www.ncnp.go.jp/hospital/news/2020/697.html		○	精神科<br>脳神経内科<br>小児神経科等	再診担当医師	
1978	こだいら小川町内科	187-0032	東京都小平市小川町2-1317-11	042-341-0654	https://kodaira-ogawa.jp	○	○	内科<br>小児科	小泉史明	
1979	こだいら内科・心臓内科	187-0004	東京都小平市天神町3-12-7-2F	042-341-7777	https://kodaira-naika.com/	○	○	内科<br>循環器内科	富田　和憲	
1980	佐々木クリニック<br>泌尿器科・小児泌尿器科	187-0002	東京都小平市花小金井8-11-9	042-344-3390	http://www.sasa-cli.com/	○	○	泌尿器科<br>小児泌尿器科	白柳　慶之	

電話や情報通信機器を用いて診療を実施する医療機関の一覧（東京都）

	基本情報					事務連絡に基づく対応について				
	施設名	郵便番号	住所（都道府県から記載）	電話番号	ウェブサイトURL	初診の電話等を用いた診療の実施の有無	再診の電話等を用いた診療の実施の有無	対応診療科	担当医師名	対面診療が必要と判断した場合に連携する医療機関名（複数ある場合は複数、住所も併せて記載）
1981	鈴木町クリニック	187-0011	東京都小平市鈴木町2-865-97　2階	042-401-1170	http://suzukicho-clinic.com/	○	○	内科<br>消化器内科　外科	岩瀬　尚子	
1982	たかの台こどもクリニック	187-00025	東京都小平市津田町1-3-8 シティホーム鷹の台#2 1階	042-345-2525	https://takanodai-kodomo.com/	○	○	小児科<br>アレルギー科	北村　知宏	公立昭和病院（東京都小平市花小金井８丁目１−１）　東京都立小児総合医療センター（東京都府中市武蔵台２丁目８−２９）
1983	多摩済生病院	187-0041	東京都小平市美園町3-11-1	042-341-1611	http://www.tama-saisei.or.jp		○	内科<br>外科<br>整形外科<br>精神科<br>皮膚科	藤田 信明<br>高橋 信樹<br>中谷 親弘<br>宮野 修<br>吉野 正興<br>小林 孝寛<br>須賀 潤<br>八尋 義昭	
1984	ながしま脳神経・頭痛クリニック	187-0044	東京都小平市喜平町1-7-17	042-332-0232	http://wwwnagashima-clinic.jp		○	脳神経外科	長島　正	
1985	八木メディカルクリニック	187-0002	東京都小平市花小金井6-17-5	042-460-3861		○	○	内科	八木知佳	
1986	矢口内科ｸﾘﾆｯｸ	187-0001	東京都小平市大沼町7ー3ー2	042-349-1168	http://www.yaguchi-naika.jp		○	内科	矢口　誠	
1987	緑成会整育園	187-0032	東京都小平市小川町1-741-34	042-341-3013	info@seiikuen.jp		○	小児科	山田　直人	
1988	緑成会病院	187-0035	東京都小平市小川西町2-35-1	042-341-3011	https://ryokuseikai.or.jp/		○	内科<br>整形外科<br>皮膚科	佐藤禎二	
1989	朝がおクリニック	191-0062	東京都日野市多摩平2-5-1クレヴィア豊田多摩平の森レジデンス111	042-506-9304	http://asagaocl.com	○	○	精神科・心療内科	工藤　吉尚	①日野市立病院（東京都日野市多摩平4-3-1）<br>② 杏林大学医学部付属病院（東京都三鷹市新川6-20-2）<br>③ 東京医科大学八王子医療センター（東京都八王子市館町1163番地）

電話や情報通信機器を用いて診療を実施する医療機関の一覧（東京都）

	基本情報					事務連絡に基づく対応について				
	施設名	郵便番号	住所（都道府県から記載）	電話番号	ウェブサイトURL	初診の電話等を用いた診療の実施の有無	再診の電話等を用いた診療の実施の有無	対応診療科	担当医師名	対面診療が必要と判断した場合に連携する医療機関名（複数ある場合は複数、住所も併せて記載）
1990	医療法人社団厚潤会花輪病院	191-0011	東京都日野市日野本町3丁目14-15	042-582-0061	http://www.hanawa-hp.com/	○	○	内科	伊藤亮治、康雄	
1991	医療法人社団康明会康明会病院	191-0053	東京都日野市豊田2-32-1	042-584-5251	https://www.komei-mc.or.jp/komeikai-hospital/		○	内科<br>神経内科	平井	日野市立病院（東京都日野市多摩平4-3-1）
1992	医療法人社団　順松会おおしろクリニック	191-0016	東京都日野市神明3-6-16アメニティ明和館	042-589-6780	https://ohshiro-clinic.net/		○	小児科<br>小児外科<br>内科<br>外科<br>泌尿器科	大城　清彦	
1993	医療法人社団仁智会　鈴木内科クリニック	191-0031	東京都日野市高幡1009-7　TIKビル２F	042-599-7021	http://www.suzukinaika -cl.com		○	内科	鈴木　隆之	日野市立病院　（東京都日野市多摩平4-3-1）
1994	おやまクリニック	191-0031	東京都日野市高幡３２８森久保医療モール２０２	042-592-4976	http://www.oyama-uro.com		○	泌尿器科	尾山博則	仁和会総合病院（東京都八王子市明神町４－８－１
1995	佐々木クリニック豊田	191-0054	東京都日野市東平山3-1-1	042-585-8063	http://www.sasakiclinic.info		○	内科	佐々木　弘子	
1996	太陽クリニック	191-0062	東京都日野市多摩平2-5-1-110	042-843-1686	https://www.taiyo-cl.com		○	小児科・内科	舘岡　昇	
1997	高瀬内科クリニック	191-0002	東京都日野市新町１－２０－３　エスペラール１F	042-582-7228	https://www.hino-takase-naika.jp/		○	内科	高瀬雅久	
1998	中川クリニック	191-0041	東京都日野市南平7-18-11	042-594-0313		○	○	内科	中川均	

電話や情報通信機器を用いて診療を実施する医療機関の一覧（東京都）

	基本情報					事務連絡に基づく対応について				
	施設名	郵便番号	住所（都道府県から記載）	電話番号	ウェブサイトURL	初診の電話等を用いた診療の実施の有無	再診の電話等を用いた診療の実施の有無	対応診療科	担当医師名	対面診療が必要と判断した場合に連携する医療機関名（複数ある場合は複数、住所も併せて記載）
1999	麦倉眼科	191-0042	東京都日野市程久保3-37-8	042-593-9910	http://mugikura-eye.byoinnavi.jp/		○	眼科	麦倉　好江	日野市立病院（日野市多摩平4-3-1）<br>東海大学八王子病院（八王子市石川町１８３８）
2000	百草の森ふれあいクリニック	191-0033	東京都日野市百草1042-22	042-599-7068	htpp://www.mogusanomori.jp/		○	内科<br>小児科	岩田　和也	
2001	森久保クリニック	191-0031	東京都日野市高幡328	042-594-6778	http://www.morikubo-clinic.jp/		○	内科<br>小児科	森久保雅道	日野市立病院（東京都日野市多摩平4-3-1）
2002	森末クリニック	191-0062	東京都日野市多摩平1-4-19藤ビル2階	042-589-3030	https://morisue-clinic.com/	○	○	内科　外科	森末　淳	
2003	リーデンス皮フ科クリニック	191-0031	東京都日野市高幡507-4リーデンススクエア高幡不動117	042-599-5455	http://www.leadence-hifuka.com		○	皮膚科	神谷　篤<br>神谷　玲子	
2004	医療法人社団幸悠会　逸見病院	189-0012	東京都東村山市萩山町三丁目26番地16	042-391-1903	http://www.kouyukai1968.or.jp		○	精神科	各医師	
2005	医療法人社団SAK さかき皮フ科・外科クリニック	189-0013	東京都東村山市栄町1-4-26クメガワメディカルビル502	042-398-7677	https://www.sakaki-cl.com		○	皮膚科	榊 聖樹	公立昭和病院（東京都小平市花小金井8-1-1）<br>一橋病院（東京都小平市学園西町1-2-25）
2006	医療法人社団新新会<br>多摩あおば病院	189-0002	東京都東村山市青葉町2-27-1	042-393-2881	http://www.sinsinkai.jp		○	精神科	富田三樹生<br>中島　直<br>他医師全員	
2007	医療法人社団正樹会<br>オーク・クリニック	189-0013	東京都東村山市栄町2-7-15	042-395-1550	無		○	内科<br>小児科、皮膚科	小川重樹	多摩北部医療センター（東京都東村山市青葉町1-7-1）

電話や情報通信機器を用いて診療を実施する医療機関の一覧（東京都）

	基本情報					事務連絡に基づく対応について				
	施設名	郵便番号	住所（都道府県から記載）	電話番号	ウェブサイトURL	初診の電話等を用いた診療の実施の有無	再診の電話等を用いた診療の実施の有無	対応診療科	担当医師名	対面診療が必要と判断した場合に連携する医療機関名（複数ある場合は複数、住所も併せて記載）
2008	医療法人社団誠優会東村山けやきクリニック	189-0024	東京都東村山市野口町2-4-35	042-409-0884	http://keyakiclinic.com/		○	小児科、内科、外科	大橋　忍	多摩北部医療センター（東京都東村山市青葉町1-7-1）<br>東京都立小児総合医療センター（東京都府中市武蔵台2-8-29）
2009	うしき婦人科クリニック	189-0014	東京都東村山市本町２−３−７９　ミネルバビル５Ｆ	042-390-8822	http://www.u-clinic.jp		○	婦人科	牛木信之	
2010	北多摩生協診療所	189-0014	東京都東村山市本町4-2-32ドルチェ久米川１階	042-393-5903	http://saito.coop/clinic/#a05	○	○	内科	坂本　匡一	
2011	公益財団法人東京都保健医療公社<br>多摩北部医療センター	189-8511	東京都東村山市青葉町一丁目７番地１	042-396-3811	http://www.tamahoku-hp.jp		○	全診療科	全医師	当院医師が電話等にて診療が可能と判断した場合のみ対応
2012	緑風荘病院	189-0012	東京都東村山市萩山町３丁目３１番地１	042-392-1101	http://ryokufuusou.com/		○	内科、外科<br>整形外科<br>小児科	内 北村竜一 他17名<br>外 玉崎良久 他9名<br>整 相原大和 他7名<br>小 宮嶋伸一郎 他3名	
2013	国分寺すずかけ心療クリニック	185-0011	東京都国分寺市本多2-3-3国分寺市商工会館５階	042-312-4406	https://kokubunjisuzukake.jp/		○	精神科	藤田　英親<br>中嶋　愛一郎<br>小石　慎子<br>斉藤　幹郎	
2014	小林内科医院	185-0012	東京都国分寺市本町１－７－３	042-300-2072	http://knaika.com	○	○	内科	小林　典雄	
2015	中島内科クリニック	185-0012	東京都国分寺市本町３－１３－１	042-327-3888	http://www.nakajima-med-cl.jp/		○	内科<br>糖尿病・内分泌内科	中島　泰	
2016	のむらクリニックスクエア	185-0011	東京都国分寺市本多1-8-3	042-325-0087	https://www.nomuracl-square.com/	○	○	内科・眼科	野村 敦宣/圭子	

電話や情報通信機器を用いて診療を実施する医療機関の一覧（東京都）

	基本情報					事務連絡に基づく対応について				
	施設名	郵便番号	住所（都道府県から記載）	電話番号	ウェブサイトURL	初診の電話等を用いた診療の実施の有無	再診の電話等を用いた診療の実施の有無	対応診療科	担当医師名	対面診療が必要と判断した場合に連携する医療機関名（複数ある場合は複数、住所も併せて記載）
2017	武蔵国分寺公園クリニック	185-0023	東京都国分寺市西元町2-16-34　ザ・ﾗｲｵﾝｽﾞ武蔵国分寺公園127	042-320-4970	http://ebm-clinic.com/	○	○	内科<br>小児科	名郷　直樹<br>福士元春<br>五十嵐　博<br>片山　淳仁<br>進谷　憲亮<br>渡邊　彩子<br>平沼　仁美	
2018	山口医院	185-0003	東京都国分寺市戸倉2-11-55	042-322-0121		○	○	内科<br>小児科皮膚科	山口　美穂	
2019	くにたち南口診療所	186-0004	東京都国立市中1-16-25シャトレクインテス国立1階	042-577-8953	http://www.santamairyou.com		○	内科<br>小児科<br>外科<br>泌尿器科<br>肛門科	淺倉　禮治<br>小島　美保	
2020	たにの耳鼻咽喉科	186-0004	東京都国立市中１－８－３６	042-571-8741			○	耳鼻咽喉科	谷野徹	
2021	クリニックみらい国立	186-0002	東京都国立市東1-6-9	042-505-7560	https://kunitachi.tama-mirai.com/		○	内科<br>皮膚科	野川　深雪<br>渥美　令子	
2022	ヒバリこどもクリニック	186-0003	東京都国立市富士見台3-1-15-2F	042-505-6017	https://hibari-kodomo.net/		○	小児科	青木　奈穂	
2023	ひろみこどもクリニック	186-0004	東京都国立市中2-20-9	042-501-0088	https://www.hiromi-kodomo-clinic.com/	○	○	小児科	七条　裕美	東京都立小児総合医療センター（東京都府中市武蔵台2ー8ー29）
2024	松田内科クリニック	186-0003	東京都国立市富士見台4-41-1グランソシエ国立ウエトウイング139号A	042-573-0010	http://matsuda-naika.clinic	○	○	内科	松田　昭彦	
2025	医療法人社団杏邦会西村医院	197-0003	東京都福生市熊川927	042-553-0182			○	内科<br>消化器内科<br>呼吸器内科	西村曜<br>西村理<br>小林英樹	

電話や情報通信機器を用いて診療を実施する医療機関の一覧（東京都）

	基本情報					事務連絡に基づく対応について				
	施設名	郵便番号	住所（都道府県から記載）	電話番号	ウェブサイトURL	初診の電話等を用いた診療の実施の有無	再診の電話等を用いた診療の実施の有無	対応診療科	担当医師名	対面診療が必要と判断した場合に連携する医療機関名（複数ある場合は複数、住所も併せて記載）
2026	医療法人社団福朗会　津田クリニック	197-0014	東京都福生市福生二宮2461	042-513-3656	http://www.clinic-tsuda.jp		○	内科<br>外科<br>肛門外科	津田　倫樹	
2027	公立福生病院	197-8511	東京都福生市加美平1-6-1	042-551-1111	http://www.fussahp.jp/		○	内科<br>精神科<br>循環器内科<br>腎臓内科<br>小児科<br>外科<br>整形外科<br>脳神経外科	各診療科担当医師	
2028	高村内科クリニック	197-0011	東京都福生市福生1044　STハウス	042-530-2710	http://www.takamuranaika.com/	○	○	糖尿病内科<br>内科	高村　宏	公立福生病院（東京都福生市加美平1-6-1）
2029	福生クリニック	197-0012	東京都福生市加美平3-35-13	042-551-2312	http://www.kanjinkai.net/		○	内科	玉木　一弘	
2030	石戸谷小児科	201-0012	東京都狛江市中和泉1-1-1狛江YSビル4階	03-3430-1070			○	小児科	石戸谷尚子	
2031	かじわらハートクリニック	201-0012	東京都狛江市中和泉5－17－27　グラウクス1階	03-5761-9091	kajiwara-heart.com		○	内科	梶原　秀俊	東京慈恵会医科大学附属第三病院（東京都狛江市和泉本町4－11－1）<br>杏林大学医学部付属病院（東京都三鷹市新川6-20-2）<br>東京都立多摩総合医療センター（東京都府中市武蔵台2－8－29）
2032	東京慈恵会医科大学附属第三病院	201-8601	東京都狛江市和泉本町４丁目１１番１号	03-3480-1151	http://www.jikei.ac.jp/hospital/daisan/		○	全診療科	各科担当医師	【備考】慢性疾患を有する再診患者が対象で医師が必要と判断した場合
2033	東京多摩病院	201-0005	東京都狛江市岩戸南2-2-3	03-3489-3191	https://tokyotama.or.jp/		○	内科	金子博	東京慈恵第三病院
2034	社会医療法人財団大和会東大和病院	207-0014	東京都東大和市南街1-13-12	042-562-1411	http://www.yamatokai.or.jp/higasiyamato/		○	脳神経外科<br>呼吸器科<br>腎臓内科<br>循環器科<br>消化器科<br>外科<br>糖尿病・内分泌科	小林　郁夫<br>並木　義夫<br>白矢　勝子<br>加藤　隆一<br>寺井　潔<br>木庭　雄至<br>犬飼　浩一<br>下山　直実	社会医療法人財団大和会<br>東大和病院附属セントラルクリニック<br>（東京都東大和市南街2-3-1）

電話や情報通信機器を用いて診療を実施する医療機関の一覧（東京都）

	基本情報					事務連絡に基づく対応について				
	施設名	郵便番号	住所（都道府県から記載）	電話番号	ウェブサイトURL	初診の電話等を用いた診療の実施の有無	再診の電話等を用いた診療の実施の有無	対応診療科	担当医師名	対面診療が必要と判断した場合に連携する医療機関名（複数ある場合は複数、住所も併せて記載）
2035	東大和病院附属セントラルクリニック	207-0014	東京都東大和市南街2-3-1	042-562-5511	tp://www.yamatokai.or.jp/higasiyamat	○	○	循環器内科	加藤　隆一<br>石野　光則	
2036	医療法人社団大塚耳鼻咽喉科医院	204-0022	東京都清瀬市松山1-40-21	042-492-0287	http://ootsukajibika.main.jp/	○	○	耳鼻咽喉科	大塚健司	
2037	医療法人社団順洋会<br>武蔵野総合クリニック	204-0021	東京都清瀬市元町1-8-30	042-496-7015	http://www.6340-group.jp/clinic/	○	○	総合診療科<br>小児科	守山医師<br>杉井医師	
2038	医療法人社団秀清会 清瀬ささき眼科クリニック	204-0022	東京都清瀬市松山1-2-23 セントラルプラザ清瀬3階	042-497-8522	http://www.kiyosesasakiganka.com/		○	眼科	佐々木　秀次	
2039	清瀬市元町しいのクリニック	204-0021	東京都清瀬市元町1-8-19　アーク清瀬１F	042-493-9880	http://www.shiino-clinic.com	○	○	内科　外科<br>整形外科	椎野　豊	
2040	清瀬診療所	204-0021	東京都清瀬市元町1-13-27	042-493-2727	http://www.saito.coop		○	内科<br>整形外科	各担当医	
2041	はたの耳鼻咽喉科	204-0021	東京都清瀬市元町１丁目８番アーク清瀬３階	042-491-1133	https://musashino-ent.jp	○	○	耳鼻咽喉科	小野　貴之	
2042	医療法人社団　鹿島医院	203-0023	東京都東久留米市南沢4-3-2	042-461-2967			○	内科<br>小児科<br>皮膚科	鹿島　研司<br>鹿島　正安<br>鹿島　真人	
2043	医療法人社団好仁会　滝山病院	203-0033	東京都東久留米市滝山4-1-18	042-473-3311	https://koujin-kai.jp/	○	○	内科	小笠原　芳宏<br>坂巻　周二	

電話や情報通信機器を用いて診療を実施する医療機関の一覧（東京都）

	基本情報					事務連絡に基づく対応について				
	施設名	郵便番号	住所（都道府県から記載）	電話番号	ウェブサイトURL	初診の電話等を用いた診療の実施の有無	再診の電話等を用いた診療の実施の有無	対応診療科	担当医師名	対面診療が必要と判断した場合に連携する医療機関名（複数ある場合は複数、住所も併せて記載）
2044	おかの内科クリニック	203-0014	東京都東久留米市東本町6-15　平和ビル2F	042-477-0055			○	内科<br>呼吸器内科	岡野　良	
2045	たきぐち内科クリニック	203-0032	東京都東久留米市前沢4-31-4	042-470-9118	http://takiguchi.byoinnavi.jp/pc/		○	内科	滝口邦彦	
2046	東久留米つぼい眼科	203-0054	東京都東久留米市中央町5-9-38	042-420-4100	https://tsuboi-eye.com/	○	○	眼科	坪井　隆政	
2047	東久留米なごみ内科診療所	203-0052	東京都東久留米市幸町3-11-14	042-470-7530	http://www.nagomi-naika.com		○	内科<br>循環器内科	榎本　光信<br>高世　秀仁	
2048	富士見通り診療所	203-0053	東京都東久留米市本町3-3-23	042-417-2291	http://saito.coop/clinic/#a01		○	内科	橋爪　洋一	立川相互病院（東京都立川市緑町4-1）<br>複十字病院（東京都清瀬市松山3-1-24）
2049	水野胃腸クリニック	203-0053	東京都東久留米市本町3丁目8-19セレブリオ本町3丁目A	042-420-6527	http://mizuno-icho.com	○	○	内科<br>消化器内科<br>肛門内科<br>アレルギー科	水野　滋章<br>水野　真理<br>川島　志布子	
2050	福山内科クリニック	203-0013	東京都東久留米市新川町1-9-22	042-470-9177		○	○	内科	福山　中	
2051	医療法人社団もかほ会武蔵村山さいとうクリニック	208-0013	東京都武蔵村山市大南3-68-3	042-590-2266	http://www.msaito.jp/clinic//	○	○	全科	齊藤　直人<br>島　秀樹	
2052	東京小児療育病院	208-0011	東京都武蔵村山市学園4-10-1	042-561-2521	https://www.kakufuh.com/tokyo_s/index.html		○	小児科<br>精神科	常勤医	

電話や情報通信機器を用いて診療を実施する医療機関の一覧（東京都）

	基本情報					事務連絡に基づく対応について				
	施設名	郵便番号	住所（都道府県から記載）	電話番号	ウェブサイトURL	初診の電話等を用いた診療の実施の有無	再診の電話等を用いた診療の実施の有無	対応診療科	担当医師名	対面診療が必要と判断した場合に連携する医療機関名（複数ある場合は複数、住所も併せて記載）
2053	独立行政法人国立病院機構村山医療センター	208-0011	東京都武蔵村山市学園２－３７－１	042-561-1221	http://www.murayama-hosp.jp/		○	内科<br>外科<br>整形外科<br>リハビリ科		
2054	武蔵村山病院	208-0022	東京都武蔵村山市榎1-1-5	042-566-3111	http://yamatokai.or.jp/musasimurayama/		○	各科	担当医	
2055	あいクリニック	206-0035	東京都多摩市中沢2-5-3<br>ゆいま～る中沢A棟1階	042-311-2820	https://kawakita.or.jp/aisafetynet/aiclinic-nakazawa/		○	内科	明石　のぞみ	多摩南部地域病院（東京都多摩市中沢2-1-2）<br>日本医科大学多摩永山病院（東京都多摩市永山1-7-1）<br>町田市民病院（町田市旭町2-15-41）
2056	あべ多摩センター内科	201-0034	東京都多摩市鶴牧1-22-2 多摩メディカルビルディング2階	042-373-7755	info@abetama-naika.com		○	内科<br>循環内科	安部　剛	
2057	井上内科クリニック	206-0034	東京都多摩市鶴牧2-24-12	042-372-3660	http://inoue-naikaclinic.jp		○	内科	井上　智雄	
2058	医療法人財団医親会<br>多摩海上ビル診療所	206-0034	東京都多摩市鶴牧2-1-1　多摩東京海上日動ビルディング地下1階	042-356-2222	https://www.tmkbclinic.com/		○	内科	木幡　義彰<br>田口　誠一郎<br>飯島　敏彦	
2059	医療法人財団共立医療会<br>多摩みなみクリニック	206-0025	東京都多摩市永山1-7-8	042-311-4415	http://tama.kyoritsu-clinic.jp/index.html		○	内科	濱田　隼一	
2060	医療法人社団栄友会　多摩ゆずクリニック	206-0033	東京都多摩市落合1-7-12ライティングビル6F	042-400-0467	https://yuzucl.jp/	○	○	老年内科	安田　裕亮	医療法人社団恵仁会　府中恵仁会病院（東京都府中市住吉町5-21-1）<br>医療法人社団慈敬会　府中医王病院（東京都府中市晴見町1丁目20）
2061	医療法人社団めぐみ会	215-0033	東京都多摩市落合1-44-401	042-311-1566	https://www.m-seikei.com/seikei/		○	整形外科	牧野　裕司<br>小島　崇紀<br>五味　範浩<br>佐々木　伸尚<br>中村　研太	

電話や情報通信機器を用いて診療を実施する医療機関の一覧（東京都）

	基本情報					事務連絡に基づく対応について				
	施設名	郵便番号	住所（都道府県から記載）	電話番号	ウェブサイトURL	初診の電話等を用いた診療の実施の有無	再診の電話等を用いた診療の実施の有無	対応診療科	担当医師名	対面診療が必要と判断した場合に連携する医療機関名（複数ある場合は複数、住所も併せて記載）
2062	医療法人社団めぐみ会<br>田村クリニック２	206-0033	東京都多摩市落合1-35<br>ﾗｲｵﾝｽﾞﾌﾟﾗｻﾞ多摩センター３階	042-357-3671	https://www.tamuracl2.com/		○	小児科<br>皮膚科<br>耳鼻咽喉科<br>漢方内科	HP参照	
2063	唐木田こどもクリニック	206-0035	東京都多摩市唐木田1-53-9 唐木田センタービル2C（2階）	042-355-8505	http://k-kodomo.com/		○	小児科<br>アレルギー科	飛田　正俊	
2064	桜ヶ丘記念病院	206-0021	東京都多摩市連光寺１－１－１	042-375-6311	http://www.swfsakura.or.jp/sakuragaokahp/		○	精神科	岩下　覚<br>他30名	
2065	三矢会　武島内科クリニック	206-0033	東京都多摩市落合3-11-3	042-338-5665	http://www.takeshima-naika-clinic.com	○	○	内科	武島英人	日本医科大学多摩永山病院(東京都多摩市永山1-7-1)
2066	耳鼻咽喉科<br>多摩センタークリニック	206-0033	東京都多摩市落合1-9-3　セゾン・ド・大貫　4F	042-376-8711	http://tamacenter.sho-jin.com/	○	○	耳鼻科	永屋　恵子	
2067	島田療育センター	206-0036	東京都多摩市中沢1-31-1	042-374-2071	https://www.shimada-ryoiku.or.jp/tama/	○	○	小児科<br>児童精神科	木実谷哲史他40名	
2068	（社）慶心会　多摩永山腎・内科クリニック	206-0025	東京都多摩市永山1－4グリナード永山５階	042-311-2417	https://www.keishinkai.or.jp/nagayama/		○	内科	松川　重明<br>津金澤　浩彦	日本医大多摩永山病院（東京都多摩市永山1-7-1）<br>多摩南部地域病院（東京都多摩市中沢2-1-2）
2069	たかまつ耳鼻咽喉科クリニック	206-0025	東京都多摩市永山1-14-12　キャスケード１　1F	042-371-1187	http://takamastu.sho-jin.com/	○	○	耳鼻科	髙松　俊輔	
2070	武井小児科	206-0011	東京都多摩市関戸２－６９－３	042-357-3030	http://www.takei-dr.jp	○	○	小児科	武井章人	

電話や情報通信機器を用いて診療を実施する医療機関の一覧（東京都）

	基本情報					事務連絡に基づく対応について				
	施設名	郵便番号	住所（都道府県から記載）	電話番号	ウェブサイトURL	初診の電話等を用いた診療の実施の有無	再診の電話等を用いた診療の実施の有無	対応診療科	担当医師名	対面診療が必要と判断した場合に連携する医療機関名（複数ある場合は複数、住所も併せて記載）
2071	多摩センタークリニックみらい	206-0033	東京都多摩市落合1-38マグレブパーキング1F	042-316-8570	https://www.tama-mirai.com/		○	内科<br>皮膚科	藤井　仁美<br>渥美　令子	
2072	多摩センターレディースクリニック	206-00034	東京都多摩市鶴牧1-22-2　3F	042-375-5122	https://www.tama-ladies.com/		○	産婦人科	清水　華子	
2073	東京みみ・はな・のどサージクリニック	206-0011	東京都多摩市関戸2-66	042-371-3387	https://tokyo-ent-surgi.com/	○	○	耳鼻科	渕上　輝彦	
2074	にしだこどもクリニック	206-0025	東京都多摩市永山1-2-14フローラ永山１A	042-376-1001	http://www.nishidakodomo.com	○	○	小児科<br>小児アレルギー科	西田　大祐<br>村田　岳哉	
2075	あいクリニック平尾	206-0823	東京都稲城市平尾3-7-4　コーシャハイム平尾1階	042-350-5062	https://www.kawakita.or.jp/aisafetynet/aiclinic-hirao/		○	内科	奥村　光絵	
2076	なかお内科クリニック	206-0802	東京都稲城市東長沼３１０７−４<br>京王リトナード稲城３F	042-401-5836	https://www.nakao-naika-cl.com	○	○	内科<br>消化器内科	中尾　裕	
2077	東長沼クリニック	206-0802	東京都稲城市東長沼１７２６－１６	042-379-4851		○	○	内科<br>小児科	中島一生美	
2078	医療法人社団天陽会　柳田医院	205-0014	東京都羽村市羽東1-30-20	042-555-1800	https://yanagida-clinic.or.jp/		○	内科・糖尿病内科	柳田和弘	
2079	あきる野総合クリニック	197-0802	東京都あきる野市草花1439-9	042-518-2088	https://akiruno-sogo-clinic.com	○	○	内科	小林　雅史	

電話や情報通信機器を用いて診療を実施する医療機関の一覧（東京都）

	基本情報					事務連絡に基づく対応について				
	施設名	郵便番号	住所（都道府県から記載）	電話番号	ウェブサイトURL	初診の電話等を用いた診療の実施の有無	再診の電話等を用いた診療の実施の有無	対応診療科	担当医師名	対面診療が必要と判断した場合に連携する医療機関名（複数ある場合は複数、住所も併せて記載）
2080	あきるの杜きずなクリニック	190-0164	東京都あきる野市五日市149ー1	042-596-6736	https://kizuna-cl.com	○	○	小児科<br>内科<br>外科<br>小児外科	小高　哲郎	
2081	あべクリニック	197-0803	東京都あきる野市瀬戸岡474-6	042-558-7730	https://abe-cl.net		○	内科・外科・小児科	阿部英雄	
2082	上代継診療所	197-0832	東京都あきる野市上代継84-6	042-559-2241	https://www.kakufuh.com/nishitama/index.html		○	小児科<br>精神科	常勤医	
2083	公立阿伎留医療センター	197-0834	東京都あきる野市引田78-1	042-558-0321	http://www.akiru-med.jp		○	全科	ホームページの診療担当医のとおり	当院の場合、慢性疾患等のかかりつけで再診予約患者のみが対象であり、初診は実施していない。詳細は当院ホームページ参照
2084	なかのやUクリニック	197-0804	東京都あきる野市秋川1-7-17	042-550-1156	http://www.nakanoya-clinic.com/		○	泌尿器科	仲野谷　祐嗣	公立阿伎留医療センター（東京都あきる野市引田78-1）
2085	安部医院	202-0015	東京都西東京市保谷町３－２４－２	042-461-0781	https://www.ambe-clinic.com	○	○	耳鼻咽喉科<br>小児科	安部　浩一<br>嶋田　眞人	
2086	医療法人社団慈秀会石田クリニック	188-0001	東京都西東京市谷戸町1-23-13	042-421-9905	http://www.ishidac.com/	○	○	内科外科胃腸科肛門科	石田秀世	武蔵野徳洲会病院（東京都西東京市向台町３－５－４８）
2087	医療法人社団エキップ みわ内科クリニック	202-0004	東京都西東京市下保谷4-12-2	042-438-7188	http://www.miwaclinic.net	○	○	内科<br>脳神経内科	三輪隆子、三輪英人	西東京市発熱外来・武蔵野徳洲会病院（東京都西東京市向台町3-5-48）
2088	医療法人社団斉藤小児科内科クリニック	202-0004	東京都西東京市下保谷4-2-21	042-421-7201	http://www.saitoh-clinic.com/201009/page/06%20shokai/shokai.html	○	○	小児科、内科	斉藤喜親	

電話や情報通信機器を用いて診療を実施する医療機関の一覧（東京都）

	基本情報					事務連絡に基づく対応について				
	施設名	郵便番号	住所（都道府県から記載）	電話番号	ウェブサイトURL	初診の電話等を用いた診療の実施の有無	再診の電話等を用いた診療の実施の有無	対応診療科	担当医師名	対面診療が必要と判断した場合に連携する医療機関名（複数ある場合は複数、住所も併せて記載）
2089	医療法人社団東光会　西東京中央総合病院	188-0014	東京都西東京市芝久保町2-4-19	042-464-1511	https://www.nishitokyo-chuobyoin.jp/		○	整形外科・総合診療科・内科・循環器内科・心臓血管外科・消化器科・耳鼻咽喉科・泌尿器科・眼科・脳神経外科・皮膚科・小児科	各科担当医師	
2090	医療法人社団翌檜会ひがき医院	188-0014	東京都西東京市芝久保町1-11-10	042-462-5521	http://www.higaki-cl.jp	○	○	内科<br>小児科<br>循環器内科	檜垣有司	
2091	桑原内科クリニック	202-0004	東京都西東京市下保谷4-13-23　保谷ｓｔメディカルビル２階	042-421-0707			○	内科	桑原星明	
2092	浩心会なかやま内科循環器クリニック	188-0013	東京都西東京市向台町1-19-14ノーブルハイツ向台1階	042-450-7710	https://www.nakayama-clinic.org/		○	内科	中山浩二	佐々総合病院（東京都西東京市田無町4-24-15 ）<br>西東京中央総合病院（ 東京都西東京市芝久保町2-4-19）<br>公立昭和病院（ 東京都小平市花小金井八丁目1-1）
2093	こみち内科クリニック	188-0014	東京都西東京市芝久保町1-5-8ハイネス田無弐番館104	042-450-6731	www.komichi-cl.com		○	内科	大湊佳子<br>大湊政之	
2094	しげみつファミリークリニック	188-0011	東京都西東京市田無町5-4-13　ハママンション第二１階	042-452-7411	https://sf-cl.jp/	○	○	内科<br>呼吸器内科	鬼澤　重光	
2095	耳鼻咽喉科ヒロクリニック	188-0011	東京都西東京市田無町4-29-8丸保ビル4Ｆ	042-463-8711	http://www.hirojibika.jp/	○	○	耳鼻咽喉科	石田　博義	
2096	すくすくkidsクリニック	188-0012	東京都西東京市南町5-9-17	042-41-3027	https://www.sukusukukids.tokyo/	○	○	小児科	髙田佳宜	
2097	たなしこどもクリニック	188-0014	東京都西東京市芝久保町3-4-30ケルン田無1	042-452-7101	tanashi-kids.jp		○	小児科	唐渡　孝子	

電話や情報通信機器を用いて診療を実施する医療機関の一覧（東京都）

	基本情報					事務連絡に基づく対応について				
	施設名	郵便番号	住所（都道府県から記載）	電話番号	ウェブサイトURL	初診の電話等を用いた診療の実施の有無	再診の電話等を用いた診療の実施の有無	対応診療科	担当医師名	対面診療が必要と判断した場合に連携する医療機関名（複数ある場合は複数、住所も併せて記載）
2098	田無耳鼻咽喉科クリニック	188-0012	東京都西東京市南町5-1-8　2階D区画	042-462-3387	http://tanashi-ent.jp/	○	○	耳鼻咽喉科<br>小児耳鼻咽喉科	新井千昭	
2099	つくしこどもクリニック	202-0012	東京都西東京市東町6-6-10	042-439-6827	http://tsukushi-kodomo.com	○	○	小児科	嶋田　達也	武蔵野赤十字病院（東京都武蔵野市）
2100	馬場医院	202-0015	東京都西東京市保谷町6-15-2	042-462-9177	https://www.baba-iin.com/	○	○	内科　小児科<br>循環器科	馬場　隆男	武蔵野徳洲会病院（東京都西東京市向台町３丁目５−４８）
2101	はるクリニック	188-0001	東京都西東京市谷戸町3-23-1　Kフラット1階	042-425-1178	https://www.haruclinic.com	○	○	内科<br>小児科	岩崎晴美	武蔵野徳洲会病院（東京都西東京市）<br>公立昭和病院（東京都小平市）
2102	ひばりが丘やまね小児科アレルギー科	188-0001	東京都西東京市谷戸町2-1-41グランドマストひばりが丘南103	042-469-3636	http://h-yamane.clinic/	○	○	小児科<br>アレルギー科	山根慎治<br>山根理恵	
2103	久内医院	202-0013	東京都西東京市中町2-1-17	042-421-3355			○	内科	久内　淳	複十字病院（東京都清瀬市松山3-1-24）<br>佐々総合病院（東京都西東京市田無町4-24-15）<br>西東京中央総合病院（東京都西東京市芝久保町2-4-19）<br>武蔵野赤十字病院（東京都武蔵野市境南町1-26-1）
2104	保谷内科呼吸器科クリニック	202-0005	東京都西東京市住吉町6丁目1番26号	042-458-7870		○	○	内科	保谷　功	
2105	まつばらホームクリニック	202-0012	東京都西東京市東町4-14-18　かえでビル2階Ｂ	042-439-1250	http://m-hc.jp		○	内科	松原　清二	辻内科循環器歯科クリニック<br>（東京都練馬区大泉学園町8-24-25）
2106	武蔵野ファミリークリニック	202-0023	東京都西東京市新町1-2-20	042-453-8500			○	内科・神経内科	金子秀平・金子厚	武蔵野徳洲会病院（東京都西東京市向台町3-5-48）

電話や情報通信機器を用いて診療を実施する医療機関の一覧（東京都）

	基本情報					事務連絡に基づく対応について				
	施設名	郵便番号	住所（都道府県から記載）	電話番号	ウェブサイトURL	初診の電話等を用いた診療の実施の有無	再診の電話等を用いた診療の実施の有無	対応診療科	担当医師名	対面診療が必要と判断した場合に連携する医療機関名（複数ある場合は複数、住所も併せて記載）
2107	やまぐち内科眼科クリニック	188-0011	東京都西東京市田無町7-16-30	042-462-7578	http://www.tanashi-yamaguchi-iin.jp		○	内科<br>眼科	山口康晴<br>山口靖子<br>児玉裕三	
2108	医療法人社団久遠会<br>高沢病院	190-1201	東京都西多摩郡瑞穂町二本木722-1	042-556-2311	http://www.takasawa-hospital.jp		○	内科	奥井　重徳	
2109	菜の花クリニック	190-1212	東京都西多摩郡瑞穂町殿ヶ谷454	042-557-7995	http://www.kanjinkai.net/		○	内科	宮元　周作	
2110	みずほクリニック	190-1231	東京都西多摩郡瑞穂町大字長岡長谷部31-1	042-568-0300		○	○	整形外科	川間　公雄	福生病院（東京都福生市加美平１丁目６−１）<br>青梅総合病院（東京都青梅市東青梅４丁目１６−５）
2111	奥多摩町国民健康保険<br>奥多摩病院	198-0212	東京都西多摩郡奥多摩町氷川1111番地	0428-83-2145	http://www.town.okutama.tokyo.jp/gyose/kakuka/ka/biyouin.html		○	内科<br>整形外科	井上　大輔<br>高梨　俊洋<br>小林　史典<br>小林　俊之	
2112	三宅村国民健康保険<br>直営中央診療所	100-1101	東京都三宅島<br>三宅村神着９３７番地	04994-2-0016<br>ただし予約は<br>ＩＰ：2-0159	http.//vill.miyake.tokyo.jp		○	内科、消化器科、循環器科、呼吸器科、小児科、外科、産婦人科、眼科、皮膚科、耳鼻咽喉科、精神科	水田　亮佑	
