# Supplementary figures and images for: Public Interest and Accessibility of Telehealth in Japan: Retrospective Analysis Using Google Trends and National Surveillance
Source: JMIR Form Res. 2022 Sep 14;6(9):e36525. doi: 10.2196/36525 (PMC9520390; doi:10.2196/36525)

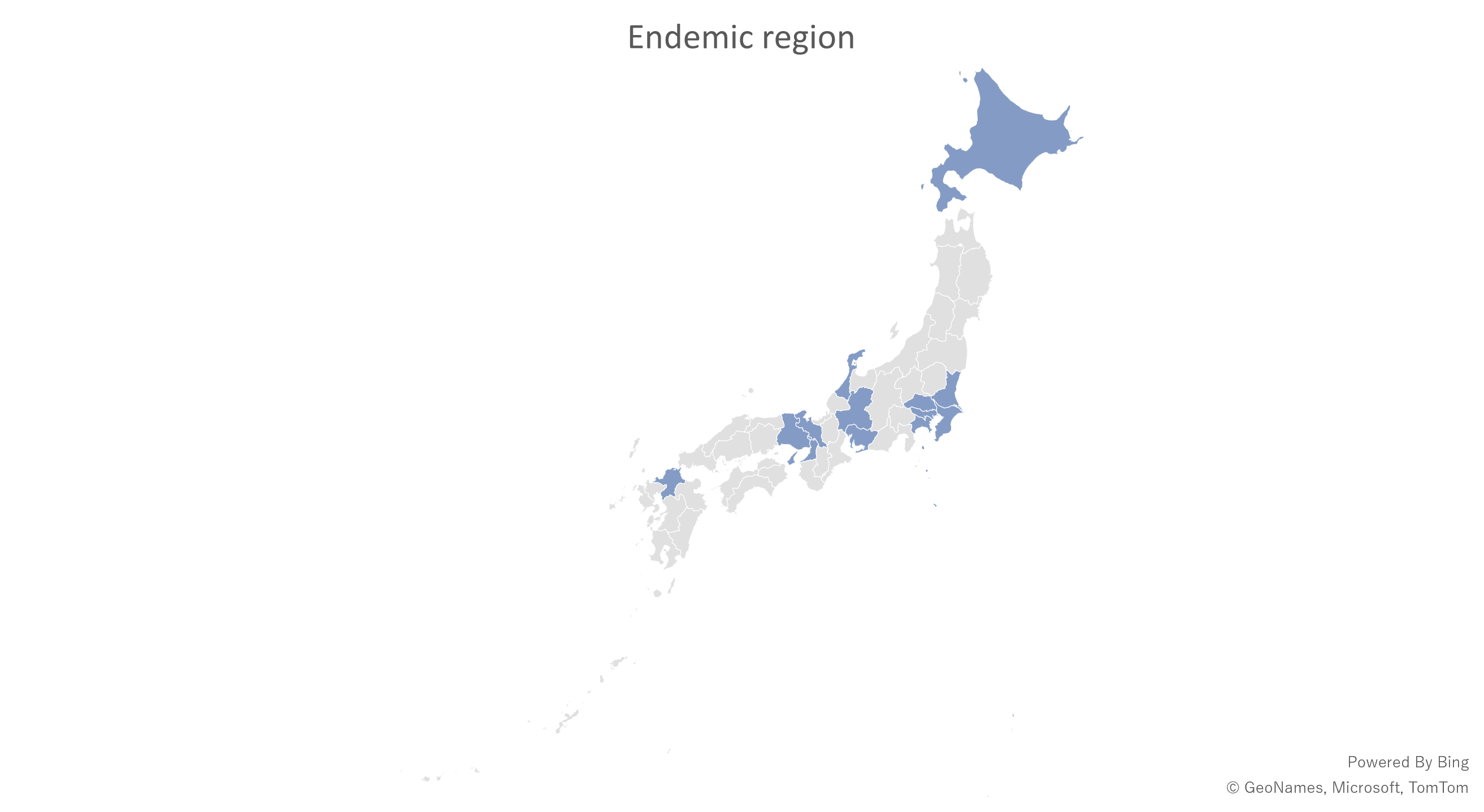

Supplement: Multimedia Appendix 4 [file formative_v6i9e36525_app4.png]
